# Developmental signs of ADHD and autism: a prospective investigation in 3623 children

**DOI:** 10.1007/s00787-022-02024-4

**Published:** 2022-06-24

**Authors:** Matti Cervin

**Affiliations:** Faculty of Medicine, Department of Clinical Sciences Lund, Child and Adolescent Psychiatry, Lund University, Sofiavägen 2D, 22241 Lund, Sweden

**Keywords:** Adhd, Autism spectrum disorder, Development, Assessment

## Abstract

Attention-deficit/hyperactivity disorder (ADHD) and autism spectrum disorder (ASD) are neurodevelopmental disorders with an early onset. Guidelines recommend a careful evaluation of developmental history when assessing the disorders, but it is unclear how children with ADHD and ASD differ from their peers growing up. In this study, physical, family, psychological, social, and educational information were examined in 3623 ethnically diverse children that were prospectively followed from birth to age 15 as part of the Fragile Families and Child Wellbeing Study. Fifteen-thousand variables were screened, and 506 variables included in the final analyses. Accuracy of the most indicative information to predict ADHD and ASD diagnoses in adolescence was evaluated. Adolescents with ADHD (*n* = 627) and ASD (*n* = 91) differed from their peers on a plethora of developmental signs, with signs closely related to the core symptoms of the disorders after age 5 being most indicative of the disorders. Predictive models correctly identified 66% of individuals with ADHD and 81% of those with ASD, but 62–88% of identified cases were false positives. The mean proportion of developmental deviations was 18.7% in the ADHD group, 20.0% in the ASD group, and 15.6% in peers; youth with both ADHD and ASD (*n* = 50) deviated on 21.8% of all developmental signs and had more pronounced deviations than those with ADHD or ASD alone. ADHD and ASD are characterized by broad and non-specific developmental deviations. Developmental information alone cannot be used to accurately predict diagnostic status in adolescence and false positives are likely if the diagnostic process relies heavily on such information. Developmental deviations are part of normal development and common in children without ADHD and ASD. Etiological heterogeneity and considerable temporal fluctuation in the core characteristics of ADHD and ASD may explain the lack of distinct developmental patterns.

**Supplementary Information:**

The online version contains supplementary material available at 10.1007/s00787-022-02024-4.

## Introduction

Attention-deficit/hyperactivity disorder (ADHD) and autism spectrum disorder (ASD) are common neurodevelopmental disorders with an early onset [1, 2]. ADHD is characterized by difficulties with inattention and/or hyperactivity and impulsivity [3] and is prevalent in children and adolescents, affecting 5–15% [4–7], with differences in prevalence rates mostly stemming from methodological characteristics of included studies [8]. ASD is characterized by enduring and impairing difficulties in social communication/interaction and restrictive and repetitive behaviors, interests, and activities, or sensory abnormalities [3] and affects 1–4% of children and adolescents [9, 10]. The two disorders commonly co-occur. About one in eight children with ADHD also meet diagnostic criteria for ASD and 40–70% of youth with ASD experience clinically significant ADHD symptoms [11]. Incidence of the disorders has increased during the last decades which appears to be the result of changes in service access, diagnostic practices, and more perceived impairment rather than true prevalence increases [10]. Causes of ADHD and ASD are unknown, but evidence suggests that their etiologies are heterogeneous, largely genetic, and that many genes with small effects are involved [2].

Early detection and diagnosis of ADHD and ASD have been highlighted as important so that affected individuals and their families can access proper information and support [12]. Early detection rests on the notion that the disorders reflect temporally stable differences in core functioning. However, increases in prevalence, less temporal stability than once assumed [13, 14], and a possible group of children onsetting with symptoms first during puberty [15] have generated discussions about the validity of the ADHD and ASD diagnoses. Concerns have been raised about possible overdiagnosis [16] and with risks of underappreciating other difficulties that may be more relevant to general functioning and health, such as intellectual functioning and learning disabilities [17].

According to the evidence-based recommendations for health care in England, practitioners assessing ADHD and ASD in children and adolescents should carefully evaluate the core symptoms of the disorders [18, 19]. The guidelines further state that the diagnostic assessment should include a full evaluation of developmental history [18]. Careful assessment of developmental history is prescribed also in several other diagnostic guidelines [20, 21]. Developmental history incorporates prenatal and perinatal information as well as development during early, middle, and late childhood. The evaluation should include but is not limited to language, speech, social communication, and motor development, and psychosocial functioning with peers, family members, and in school. There are no empirically validated measures or interviews available to guide professionals on which developmental history to evaluate in ADHD [22]. In ASD, some measures exist, but their design is based on samples with extremely high base rates of ASD, casting doubt on their accuracy in other samples and in general pediatric populations [23, 24]. The field is therefore missing adequate guidance about which developmental signs are indicative of ADHD and ASD and which developmental domains are most implicated in the disorders. With a lack of empirical guidance, overreliance on unstructured clinical judgement, which is sensitive to multiple biases, may guide the evaluation of developmental history. This can lead to poor precision in the diagnostic process, with potentially detrimental outcomes for those not receiving a diagnosis when it is warranted and those receiving a diagnosis when it is not warranted.

A broader understanding of which developmental signs are indicative of ADHD and ASD needs to be based on comprehensive and systematically collected prospective data in diverse samples of children. At current, the literature has established that a myriad of social, behavioral, biological, neural, and psychological factors are under- and overrepresented in individuals with ADHD and ASD [7]. A fairly large literature has also examined children with familial risk of ASD and their development over time [25], indicating that children with ASD show difficulties with social and communication skills already during the second year of life. In addition, there is a literature on the temporal stability of ADHD symptoms showing moderate stability and predictive validity [26–28]. However, current studies are characterized by examining one or a handful factors at the time, often in non-representative samples (e.g., treatment-seeking clinical samples or those that have received a certain diagnostic code in nationwide registries). Narrow developmental information in non-diverse samples provides little guidance about broader patterns of developmental deviances in ADHD and ASD, and thus has limited implications for clinical practice and a better understanding of the core developmental domains that are affected in the disorders.

This study synthesized and analyzed rich developmental data in a diverse and prospectively and systematically assessed sample of children to identify how children with ADHD and ASD deviate as they grow up. The first aim was to establish which developmental signs were most indicative of ADHD and ASD (exploratory analyses). The second aim was to evaluate with which accuracy this information predicted ADHD and ASD diagnoses in adolescence (confirmatory analyses). The goal was to provide empirical information about developmental deviations in ADHD and ASD to guide the evaluation of developmental history in clinical practice and inform research on behavioral phenotypes and developmental processes implicated in the disorders.

## Method

### Participants and recruitment

Prospectively collected data from the Fragile Families and Child Wellbeing Study were analyzed. The Fragile Families and Child Wellbeing Study collected developmental, family, physical, psychological, social, and educational information at six time points from birth to age 15 and has been described in detail elsewhere [29]; plenty of information about study design, collected data, and publications is available online (https://fragilefamilies.princeton.edu/). The Fragile Families and Child Wellbeing Study included children born between 1998 and 2000 and oversampled children to unmarried mothers resulting in the inclusion of many Black, Hispanic, and low-income families. The first interview was conducted at the time of birth and follow-up interviews were conducted when children were approximately 1, 3, 5, 9, and 15 years old.

### Ethical considerations

The study protocol for the Fragile Families and Child Wellbeing Study was approved by The Princeton University Institutional Review Board (IRB 13–1946). The study was also approved by appropriate review boards at affiliated universities and hospitals. Legal guardians and primary caregivers signed informed consent and adolescents provided assent. Participants received financial compensation for their time. To secure confidentiality, the data used in this study were deidentified and geographical identifiers and genetic and medical variables were not included.

### Developmental information

The essential information at birth and ages 1 and 3 was provided by caregivers, particularly mothers. At age 5, both caregivers and teachers provided information. At age 9, caregivers, teachers, and the children themselves provided information. Information about all the variables collected as part of the larger study is available online (https://fragilefamilies.princeton.edu/data-and-documentation/public-data-documentation). The online documentation alongside a screening of all variables at each wave of data collection (15,380 variables in total) resulted in the selection of 506 variables for the final analyses. All variables that tapped into the developmental domains of the child as well as family history of psychiatric disorders were included in analyses.

The 506 variables included four variables that assessed factors during pregnancy and birth (e.g., smoking, drinking, low birth weight), 20 variables for family presence of psychiatric disorders. In addition, 43 variables about broader physical development were analyzed and these included general health (e.g., asthma, allergies, seizures, diabetes, pain conditions), language and speech difficulties, height and weight development, accidents, and service use (these variables were collected from birth to age 9). With regard to caregiver-reported developmental signs, 7 variables were included at age 1 (e.g., shyness, fussiness, social reciprocity), 67 variables at age 3 (e.g., sleep problems, interaction with peers, interaction with adults, concentration, interests, mood and temperament, temper tantrums), 87 variables at age 5 (similar to age 3 but with a richer palette of functioning and behavior across social, interpersonal, educational and cognitive domains), and 113 variables at age 9 (further expanding the palette). For teacher-reported developmental information, 18 variables were included at age 5 (e.g., activity level, disabilities, performance within several school subjects) and 83 variables at age 9 (with more information about interaction with peers and teachers, cognitive skills, flexibility, schoolwork, and temperament and mood). For self-reported information, 52 variables were included at age 9 (e.g., functioning in school and at home, emotional problems, peer relations). Eight performance-based variables were included. The Peabody Picture Vocabulary Test (PPVT) was conducted at years 3, 5, and 9 during in-home visits. PPVT is a standardized test that measures the vocabulary of children. At age 5, in addition to PPVT, Leiter tests measuring attention and impulsivity were conducted. At age 9, in addition to PPVT, the forward and backward digit span (working memory) from the Wechsler Intelligence Scales for Children (WISC) were conducted and two tests from the Woodcock-Johnson Test of Cognitive Abilities: the passage comprehension test (reading and language skills) and the applied problems Test (math reasoning). Last, four variables from the Maternal Description of Child (MDoC) test were included where mothers described their child verbally and the degree of positive affect, negative affect, detachment, and amount/descriptiveness of speech was scored.

All variables that included information about developmental signs was transformed into binary variables with each variable indicating a clear deviation regarding that particular information. For example, a variable examining the child’s ability to feel guilt was coded such that a clear indication of problems with feeling guilt was indicated. Thus, ordinal items (e.g., Not at all, Not so much, Somewhat, A lot, Very Much) were binarized (deviation or not). This was done to mirror the yes/no properties of developmental questions asked during an evaluation of developmental history and the ability of caregivers to remember developmental signs retrospectively. Test scores were not binarized since they were normally distributed and can be used as part of an overall evaluation of developmental history (e.g., by reviewing documentation from school assessments). In a supplementary file, available online, the design of each variable is described alongside information about which original variables were used.

ADHD and ASD status was derived using the information at year 9 and 15 where caregivers reported whether a medical professional had diagnosed their child with either ADHD or ASD. Participants with missing information about ADHD and ASD status at ages 9 and 15 (*n* = 1275) were omitted.

### Statistical analysis

The data were split into training and test sets and final predictive models were run with the unexamined data of the test set. First, to identify for which variables youth with ADHD and ASD differed from their peers, logistic regression models to predict adolescent ADHD outcome and Firth (also referred to as penalized likelihood) logistic regression [30] to predict adolescent ASD outcome were conducted. Firth regression was used because of the unbalanced nature of the outcome variable, that is, few had ASD. The initial models were run with each separate factor as an independent variable. Sex was included as a covariate because of the known difference in sex distribution for ADHD/ASD and for many of the developmental signs that were examined. Because of the large sample, an alpha level of 0.01 was used as an indicator of statistical significance in the separate models. Risk of type I errors was accounted for by (i) analyzing selected variables in sets (see below) and (ii) running the final models on the test set (i.e., previously unanalyzed data). After running separate models for each variable, variables from the same domain (e.g., teacher rating, parent rating, family history of psychiatric disorders) and/or timepoint (e.g., birth, year 1, year 3) were analyzed in sets to determine which variables were most important to predict ADHD/ASD status during adolescence. After identifying the most salient test data variables, test data were analyzed alongside teacher-reported data from the same age since the tests assessed competencies needed in school. Complete enumeration [31] was used to find the best subset of variables and the models were run with synthesized data with an even proportion of cases and non-cases. Evenly distributed cases provide better coefficient estimates to identify true cases. When identifying the final subset of variables to determine the optimal coefficients for each variable, missing data were handled through multiple imputations with five imputations and 10 iterations using predictive mean matching. Multiple imputation was used because listwise deletion would render in the loss of a large proportion of cases when multiple variables were analyzed simultaneously. Adolescent ADHD/ASD status was not imputed. A final predictive model was selected based on findings in the training sets, and separate models were selected for ADHD and ASD. The selected model was then conducted with the test set. The accuracy of the model was evaluated by examining sensitivity (i.e., the proportion of true cases identified as cases) and the positive predictive value (PPV; i.e., the proportion of identified cases that are true cases). Accuracy was evaluated using imputed test data and probabilities of 50% and 80%, respectively. All analyses were conducted in R Studio and the statistical scripts are available in the supporting information, available online.

## Results

Table 1 shows information about participants in the full sample and subsamples. At age 15, 627 participants (17.3%) had received an ADHD diagnosis and 91 participants (2.5%) an ASD diagnosis. Of those with ADHD, 334 (57.4%) had received the diagnosis at age 9 (45 participants had missing data at this time point). Of those with ASD, 41 (50.0%) had received the diagnosis at age 9 (9 participants had missing data at this time point). Of those with ADHD, 50 (8.0%) had an ASD diagnosis. Of those with ASD, 50 (54.90%) had an ADHD diagnosis. Tables S1 and S2 in the Supplemental, available online, show all variables for which adolescents with ADHD and ASD, respectively, differed from their peers growing up. Adolescents with ADHD differed statistically significantly on 52.0% of all analyzed variables and all differences were in the direction such that those with ADHD had more prevalent problems or difficulties. Adolescents with ASD differed statistically significantly on 38.3% of all variables and for all but one variable (Watching TV more than 4 h per day; child-report at year 9), adolescents with ASD exhibited more problems or difficulties growing up.

**Table 1:** Participant characteristics

	All	ADHD	ASD	Neither ADHD nor ASD
*n*	3623	627	91	2955
Male, *n* (%)	1882 (51.9%)	451 (71.9%)	74 (81.3%)	1400 (47.4%)
Race of child^a^				
Black non-Hispanic, *n* (%)	1594 (49.0%)	245 (47.9%)	18 (29.0%)	1342 (49.4%)
White non-Hispanic, *n* (%)	588 (18.1%)	123 (24.1%)	23 (37.1%)	452 (16.7%)
Hispanic, n (%)	809 (24.9%)	100 (19.6%)	11 (17.7%)	705 (26.0%)
Other, *n* (%)	261 (8.0%)	43 (8.4%)	10 (16.2%)	215 (7.9%)
Below poverty threshold, *n* (%)	1275 (35.2%)	232 (37.0%)	24 (26.4%)	1032 (34.9%)
Age of mother at birth,* M* (*SD*)	25.15 (6.02)	24.61 (5.84)	26.13 (5.79)	25.25 (6.02)
ADHD	627 (17.3%)	627 (100%)	50 (54.9%)	0 (0%)
ASD	91 (2.5%)	50 (8.0%)	91 (100%)	0 (0%)

*ADHD* Attention-deficit/Hyperactivity disorder, *ASD *Autism Spectrum Disorder

^a^Based on information at age 15, missing data for 10.2% of participants

**Fig. 1:**
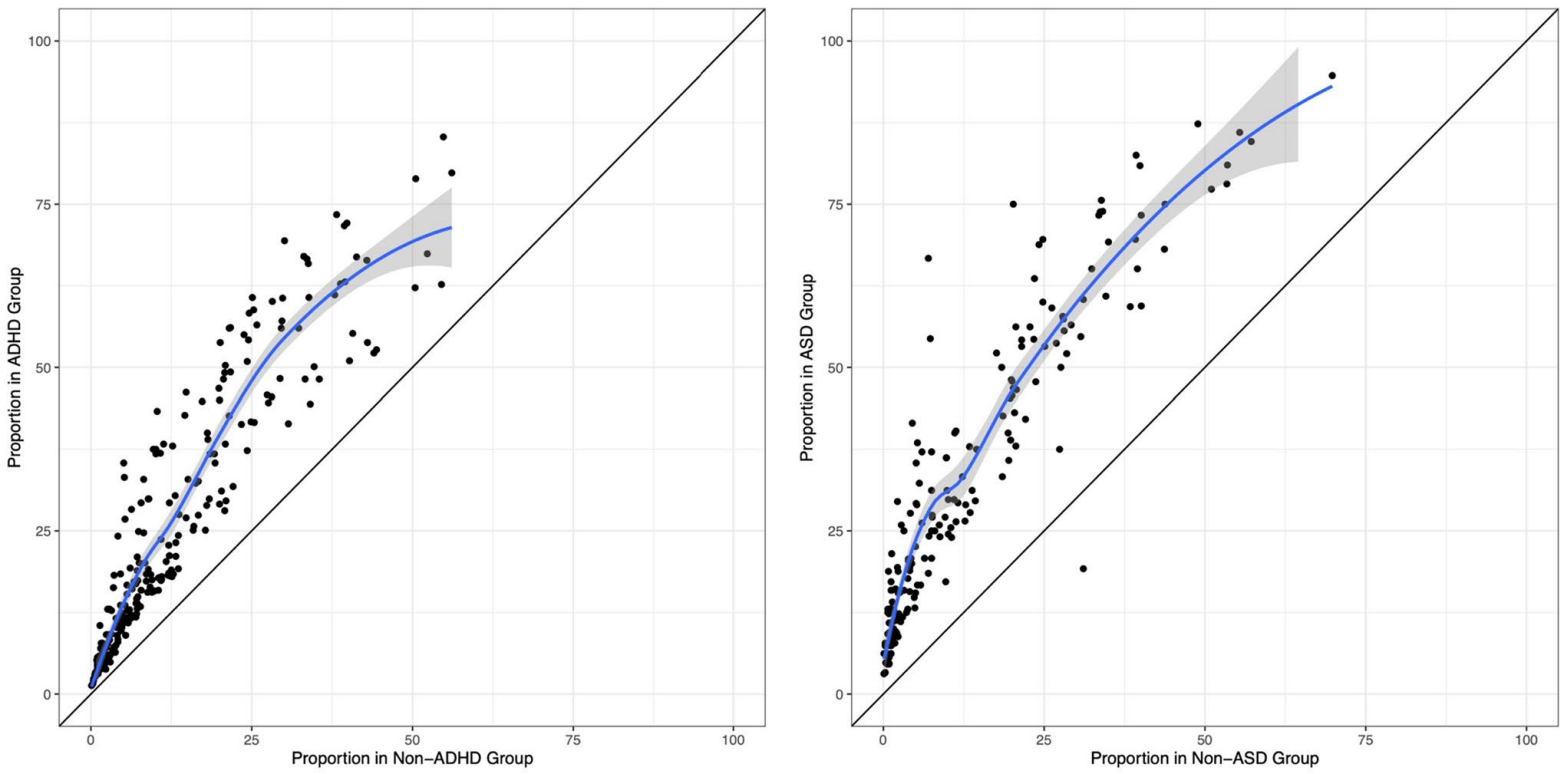
Dot plots showing the proportion in ADHD/ASD and peers of each developmental sign for which adolescents with ADHD/ASD differed from peers, with the diagonal line indicating where the proportion would be equal in both groups. The blue line is a locally fitted regression line and the shaded area represents its 95% confidence interval. *ADHD* Attention-deficit/Hyperactivity disorder, *ASD* Autism Spectrum Disorder

Figure 1 shows the proportion of each developmental sign (for which there was a statistically significant difference) in the ADHD group versus peers and in the ASD group versus peers, respectively. The figure shows that although many signs were significantly more prevalent in ADHD/ASD, the pattern of prevalence was similar across groups (i.e., signs common in ADHD/ASD were also relatively common in peers, and signs less prevalent in ADHD/ASD were also uncommon in peers).

**Fig. 2:**
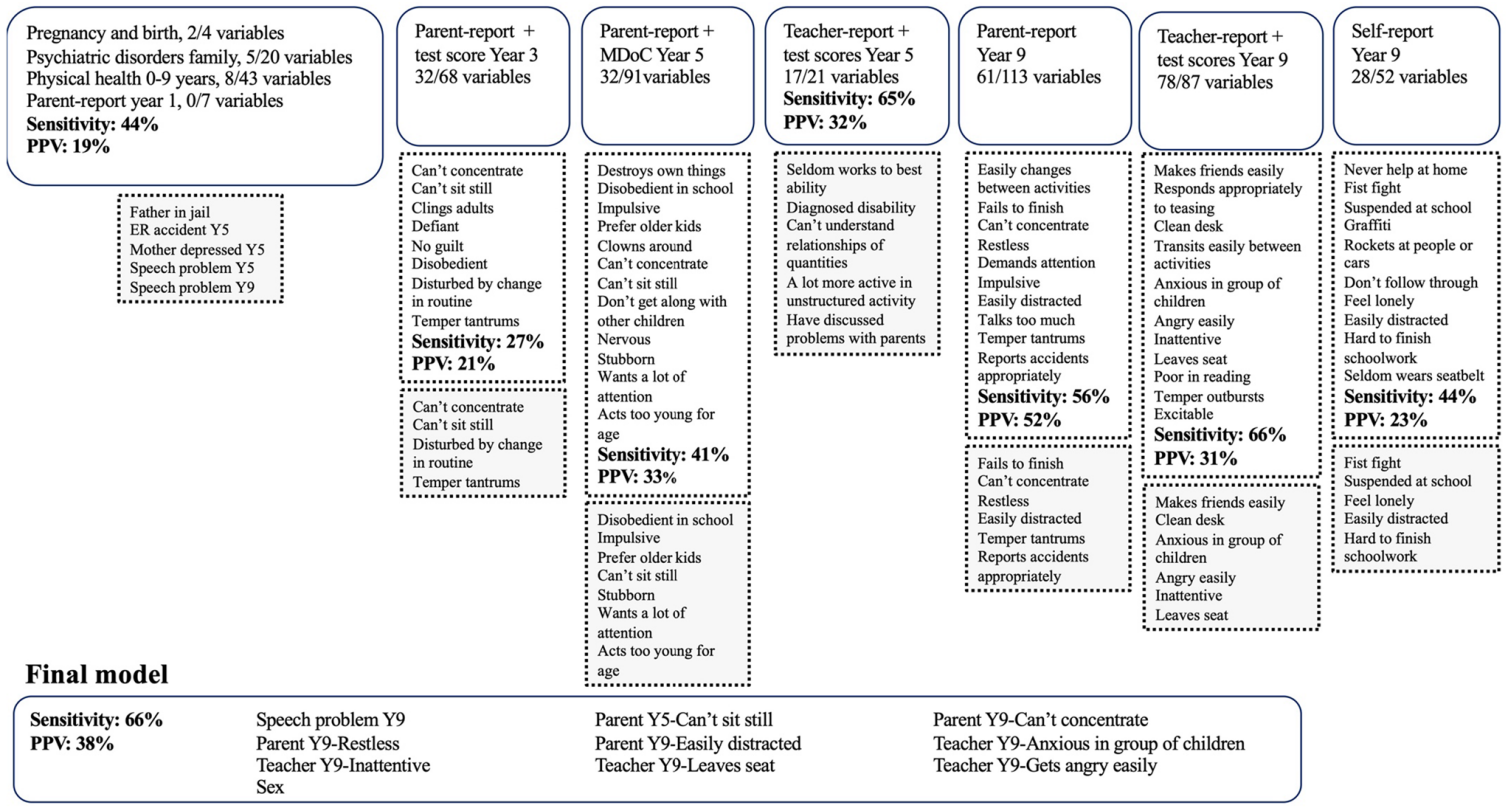
Variable selection process for predicting adolescent ADHD status, with the grey areas showing variables that were included in the final model selection. *ADHD* Attention-deficit/Hyperactivity disorder

Fig. 2 shows the full variable selection process for ADHD. The final model included nine developmental signs, of which eight (88.9%) were from the age 9 assessment. The model also included sex. Four caregiver-reported variables, four teacher-related variables, and speech problems at year 9 were included in the final model. The only variable that was not collected at age 9 was caregiver-reported difficulties with sitting still at age 5. The confirmatory model in the test set, in which individuals with a probability equal to or above 50% were classified as cases, correctly identified 66.2% of adolescents with ADHD, but 62.1% of identified cases were false positives. When a probability level of 80% was used, 23.3% of those with ADHD were identified correctly and 37.4% were false positives.

**Fig. 3:**
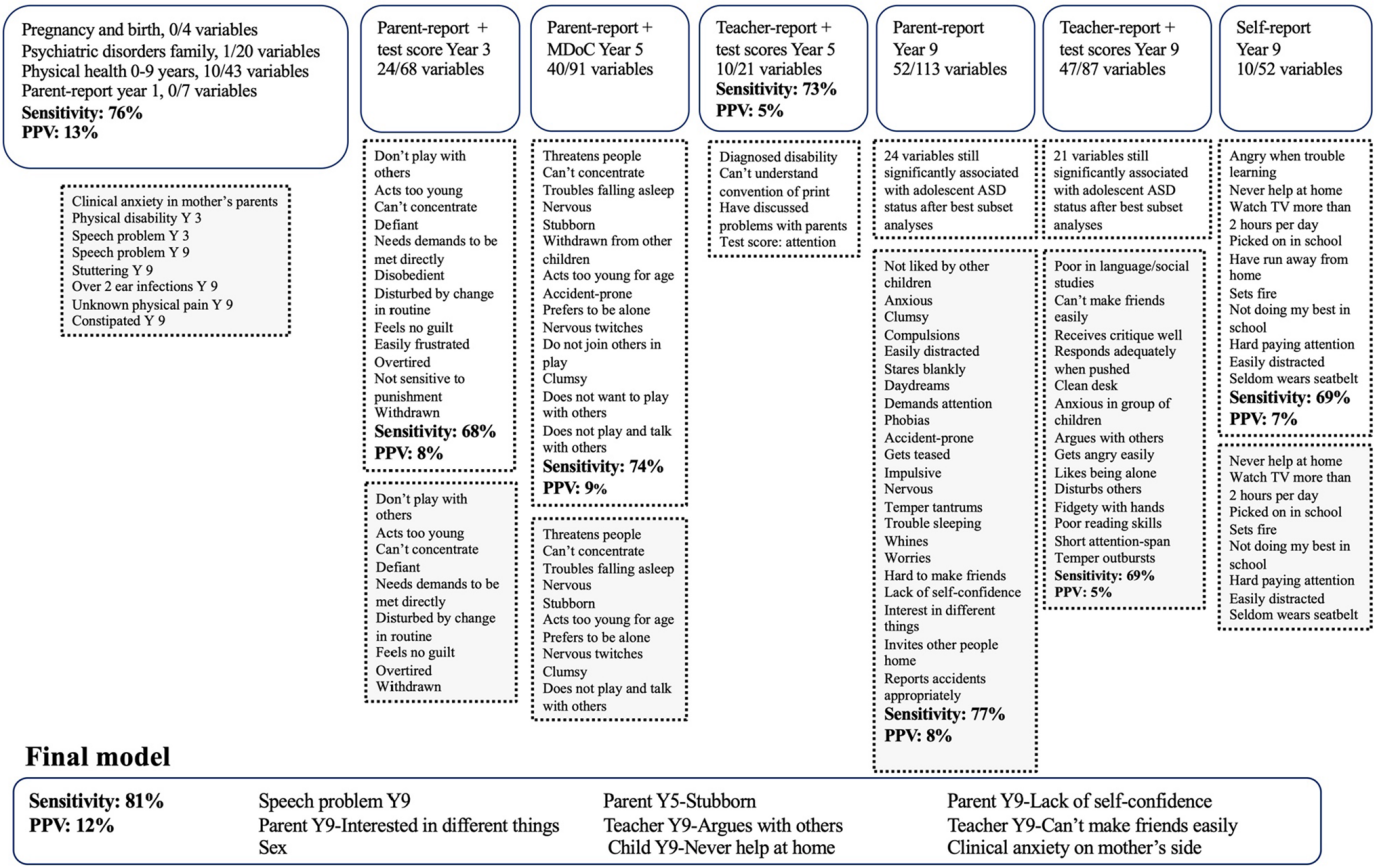
Variable selection process for predicting adolescent ASD status, with the grey areas showing variables that were included in the final model selection. *ASD* Autism Spectrum Disorder

Fig. 3 shows the full variable selection process for ASD. The final model included eight developmental signs, of which six (75.0%) were from the age 9 assessment. Three caregiver-reported variables, two teacher-reported variables, one child-reported variable, speech problems at year 9, and familiar risk of clinical anxiety on the mother’s side were included as predictors. The confirmatory model in the test set, in which individuals with a probability equal to or above 50% were classified as cases, correctly identified 81.8% of adolescents with ASD, but 87.6% of identified cases were false positives. When a probability level of 80% was used, 51.4% of those with ASD were identified correctly and 74.1% were false positives.

To further explore patterns of developmental deviations in the cohort, the number of deviations each participant exhibited during childhood was counted. The maximum number was 496 (numerical variables were excluded) and all participants that had data on 10% or more of all variables were included. Available data were used to estimate the number of deviations that each participant would exhibit whether no missing data were present. The mean number of developmental deviations was 80.11 (*SD* = 26.48, 16.2% of variables). Only one participant (0.0003%) showed zero developmental deviations and only 10.1% of all participants showed deviations on less than 10% of the variables. In those without ADHD/ASD, 11.1% showed developmental deviations on less than 10% of the variables. The difference in the mean number of developmental deviations between those with (*M* = 92.96, *SD* = 28.89, 18.7% of variables) and without ADHD (*M* = 77.24, *SD* = 24.97, 15.6% of variables) was statistically significant (*p* < 0.0001). As was the difference between those with (*M* = 99.21, *SD* = 35.40, 20.0% of variables) and without ASD (*M* = 77.26, *SD* = 24.97, 15.6% of variables; *p* for difference < 0.0001).

Post-hoc analyses were conducted to analyze whether the dually affected group (those with ADHD + ASD, *n* = 50) differed from those with ADHD or ASD alone. The mean proportion of deviations across all analyzed variables and the 17 unique variables included in the final predictive models were compared across the three groups. A one-way ANOVA with Tukey-corrected follow-up pairwise group comparisons showed that the dually affected group deviated on more variables (21.8%) than those with ADHD alone (18.4%, *p* < 0.001) or ASD alone (17.9%, *p* < 0.001). The two latter groups did not differ statistically significantly from each other (*p* = 0.89). For 7 of the 17 variables in the final predictive models, chi-square tests showed that the proportion of youth exhibiting a specific deviation was higher in the dually affected group than in the ADHD/ASD only groups. Significant differences emerged for the following variables: parent-reported stubbornness at age 5 (41.0% [ADHD + ASD] vs 15.8% [ADHD only] and 30.3% [ASD only], *p* < 0.001), clinical anxiety on the mother’s side (50.0 vs 25.2% and 30.3%, *p* < 0.001), parent-reported restlessness at age 9 (35.5 vs 12.9% and 23.5%, *p* < 0.001), parent-reported concentration problems at age 9 (50 vs 33.9% and 14.7%, *p* < 0.001), parent-reported problems with being easily distracted at age 9 (43.2 vs 24.4% and 14.7%, *p* < 0.001), teacher-reported problems with being anxious in groups of children at age 9 (78.6 vs 44.6% and 52.2%, *p* < 0.001), and teacher-reported difficulties with making friends at age 9 (87.1 vs 53.6% and 45.5%, *p* < 0.001).

## Discussion

This study addresses an important current gap in the literature on neurodevelopmental disorders, namely how children with ADHD and ASD differ from their peers growing up. Empirical investigations into this topic are important to provide practitioners with empirical guidance in their evaluation of developmental history, which is recommended as part of the overall diagnostic evaluation of ADHD and ASD [18, 19]. Clarity on this topic may also inform our understanding of behavioral phenotypes and developmental processes implicated in ADHD and ASD. The big strength of the study is the integrated analysis of comprehensive developmental information collected from birth to age 9. The access to rich and prospective material helped provide a broader picture of childhood development in ADHD and ASD. Study results are valuable additions to a field that is dominated by examining one single sign or symptom at the time, usually in non-diverse samples.

The first major result is that ADHD and ASD are characterized by a range of developmental deviations during childhood. All deviations indicate that the disorders are characterized by difficulties that may worry caregivers and others close to the child, as well as leading to distress for the child. In ADHD, signs related to attention difficulties, impulsivity, and hyperactivity were present from age 3. In fact, in the final model, six out of nine developmental signs (of which five were collected at age 9) were directly related to the diagnostic criteria of the disorder, with these signs covering all three diagnostic domains: attention, hyperactivity, and impulsivity. Speech problems at age 9 and teacher-reported age 9 problems with anger and being anxious in groups of children were also included in the final model. The latter findings imply that signs and symptoms indicative of both internalized (e.g., anxiety) and externalized emotionality (e.g., temper tantrums) are prevalent in ADHD throughout development. These results are in line with current research showing increased rates of anxiety and behavioral disorders in children with ADHD [32] and studies indicating that emotional reactivity and difficulties with emotion regulation may be as relevant to pediatric ADHD as the core symptoms of the disorder [33].

The final model correctly classified 66% of adolescents with ADHD. Importantly, 62% of all identified cases were false positives, meaning that many adolescents were identified as having ADHD when they did not. A model in which a probability of 80% was used identified fewer with ADHD (i.e., lower sensitivity), but also resulted in a lower proportion of false positives (i.e., higher PPV). Low accuracy of developmental information to identify ADHD was also shown in a previous longitudinal study where information at 6 and 18 months could not adequately predict a later ADHD diagnosis, although several variables were statistically significantly associated with later ADHD [34]. One credible explanation for the low accuracy is that developmental signs common in ADHD are common also in the general population and that the distinction between what is defined as clinical ADHD (and not) is gradual rather than fixed. This is in line with evidence suggesting that symptoms of mental and neurodevelopmental disorders fall on a continuum with differences in degree rather than in kind in the general population [35, 36]. The present study found only a small difference between the overall proportion of developmental deviations in ADHD and peers (18.7 vs 15.6%), further corroborating differences in degree rather than in kind. It is also possible that ADHD traits fluctuate considerably over time, which is supported by previous research [13]. Temporal fluctuations would result in low precision of historical data to identify current difficulties. Another plausible explanation for the low accuracy is substantial heterogeneity in ADHD symptoms and in the diagnosis of ADHD. That is, if individuals with ADHD differ from each other, it is unlikely that clear developmental patterns will be identified. Taken together, it appears as false positives are likely if the diagnostic process relies heavily on developmental information when conducting a diagnostic assessment of ADHD.

Adolescents with ASD also differed from their peers on a large number of developmental signs. Many signs were related to atypical behaviors in interaction with peers: tendencies of not joining others in play and aggressive and disturbing social behaviors. Attention, hyperactivity, and impulsivity problems were also linked to ASD, as were several signs indicating internalized symptoms such as anxiety, worry, and phobias. In contrast with ADHD, the final predictive model included a broader range of variables than signs and symptoms closely related to disorder criteria. Most of the included variables (6 out of 9) were from the age 9 assessment and these included speech problems, parent-reported difficulties with limited interests and lack of self-confidence, teacher-reported difficulties with problems making friends and arguing with others, and self-reported lack of helping with chores at home. Further, familial risk of clinical anxiety on the mother’s side and parent-reported stubbornness at age 5 were included. The model identified 82% of adolescents with ASD but 88% were false positives. An increased probability level of 80% resulted in lower sensitivity but higher PPV without changing the major interpretation of the result. Possible reasons for the low accuracy are the same as for ADHD, that is, ASD symptoms may be gradual rather than part of fixed states, heterogenous, and may fluctuate over time.

The most probable explanation for the low accuracy of developmental information to identify youth with ADHD or ASD is that the disorders are characterized by substantial etiological and phenotypic heterogeneity and temporal fluctuation in signs and symptoms. However, a competing explanation is that the validity of the diagnostic information was low. For example, parents may have reported that their children had been diagnosed even when this was not the case. This is not supported by research about parent-reported ASD diagnoses that indicates very high concordance between parent-report and register information (93–98%) [37–39] and parents have been shown to provide accurate information also in relation to ADHD [40]. This indicates high validity of the parent-reported diagnostic information in the present study.

Another explanation is that the children had received diagnoses but that these diagnoses had limited clinical validity (i.e., misdiagnosis). This is partly supported by the prevalence of ADHD in this cohort, which was higher than what has been reported in other studies with US cohorts [41]. However, the high rates of ADHD may be explained by that ADHD status was determined in mid adolescence (since the prevalence of ADHD diagnoses increases with age). The broader literature provides limited guidance about the validity of diagnoses in clinical practice, but comparisons of case notes and register-retrieved diagnoses of ASD show high validity of register-based diagnoses [42], lending further support for the validity of the diagnostic outcomes analyzed in the present study. Further, adolescents with ADHD and ASD differed from peers on a range of developmental signs, indicating that group allocation indeed had merit, but that the developmental information was not sufficient to classify ADHD/ASD status with high precision.

The only variables that were included in the final models for both ADHD and ASD were sex (being male) and speech problems at age 9. This may be surprising since ADHD and ASD often co-occur and did so also in the present sample. Thematical overlap was found for difficulties interacting/getting along with peers, which is supported by prior research showing that impaired social functioning is a shared trait in ADHD and ASD [11]. That speech problems were indicative of both disorders is not surprising since language deficits are common in ADHD [43] and communication difficulties are included in the diagnostic criteria of ASD [3]. Post hoc analyses suggested that the dually affected group (ADHD + ASD) exhibited both the most frequent and the most pronounced signs of developmental deviations. This is in line with previous research showing that youth with co-occurring ADHD and ASD are more impaired and have more difficulties with executive functioning than those with ADHD or ASD alone [11].

Several limitations merit mentioning. As mentioned above, the unknown validity of ASD and ADHD status in adolescence is a limitation of the present study and future studies that include high-quality diagnostic data can repeat the approach here and evaluate whether overall results converge. Second, very rich developmental material was analyzed but not all information that may be relevant was included; for example, more detailed information about developmental milestones would have strengthened conclusions. Third, variable selection when working with many variables is complex, and other statistical models and techniques than those used here are available (e.g., decision trees). It is out of the scope of the present paper to compare model performance, but future work may want to do this, although type of statistical model was not linked to predictive performance in relation to life outcomes at age 15 in a large collaborative study that employed the same dataset as in this study [44]. Last, few individuals had ASD, which inevitably results in a lack of information to train models and improve accuracy. Future studies should try to oversample on individuals where ASD is likely.

Adolescents with ADHD and ASD differ from their peers on a range of developmental signs growing up, but no single developmental sign or assembly of signs from birth to age 9 can be used to classify disorder status in adolescence. Etiological heterogeneity and considerable fluctuation over time in the core characteristics of the disorders may explain the lack of distinct developmental patterns in ADHD and ASD. This study also showed that developmental deviations are part of normal development with 89% of adolescents without ADHD and ASD showing deviations on at least 10% of all analyzed developmental signs. Study conclusions are limited by the uncertainty of the clinical validity of ADHD/ASD status in adolescence, but results are consistent with ADHD and ASD being gradual, heterogenous, and temporally unstable syndromes.

### Supplementary Information

Below is the link to the electronic supplementary material.

Supplementary file1 (DOCX 54 KB)

Supplementary file2 (DOCX 78 KB)

Supplementary file3 (DOCX 72 KB)
