# Supplementary material for: Developmental signs of ADHD and autism: a prospective investigation in 3623 children
Source: Eur Child Adolesc Psychiatry. 2022 Jun 24;32(10):1969–78. doi: 10.1007/s00787-022-02024-4 (PMC10533573; doi:10.1007/s00787-022-02024-4)
Supplement: Supplementary file 1 — Supplementary file1 (DOCX 54 KB) [file 787_2022_2024_MOESM1_ESM.docx]

**Table S1.** Variables in the training set for which adolescents with ADHD differed from their peers while accounting for differences in sex

|  | ADHD  *n* = 511 | Neither ADHD nor ASD  *n* = 2353 |
| --- | --- | --- |
| Boys, *n* (%) | 364 (71.2%) | 1112 (47.3%) |
| **Pregnancy and birth (4 variables in total)** | | |
| Mother smoked during pregnancy, *n* (%) | 128 (25.1%) | 418 (17.8%) |
| Father in jail at birth, *n* (%) | 32 (6.3%) | 77 (3.3%) |
| **Family presence of psychiatric disorders (20 in total)** | | |
| Clinical anxiety in mother’s parents, *n* (%) | 96 (28.1%) | 342 (20.8%) |
| Mother depressed at year 1, *n* (%) | 84 (17.6%) | 243 (11.0%) |
| Father substance use at year 3, *n* (%) | 27 (7.5%) | 67 (3.9%) |
| Mother depressed at year 3, *n* (%) | 91 (19.2%) | 293 (13.6%) |
| Mother depressed at year 5, *n* (%) | 76 (15.9%) | 229 (10.5%) |
| **Physical Health Factors (43 in total)** | | |
| Top 10% healthcare visits year 1, *n* (%) | 73 (17.5%) | 181 (9.5%) |
| Top 10% healthcare visits year 3, *n* (%) | 68 (17.4%) | 195 (10.9%) |
| Emergency room because of accident year 5, *n* (%) | 61 (15.8%) | 164 (9.9%) |
| Speech problems year 3, *n* (%) | 36 (10.2%) | 67 (4.3%) |
| Speech problems year 5, *n* (%) | 48 (11.7%) | 101 (5.4%) |
| Speech problems year 9, *n* (%) | 82 (17.2%) | 147 (6.7%) |
| Poor health year 9, *n* (%) | 25 (5.3%) | 59 (2.7%) |
| Weight percentile year 9, *M* (*SD*) | 64.12 (30.60) | 68.27 (29.45) |
| **Parent-Reported Year 1 Factors (7 in total)** | | |
| *No statistically significant differences* |  |  |
| **Parent-Reported Year 3 Factors (67 in total)** | | |
| Secure attachment, *n* (%) | 199 (68.2%) | 968 (77.9%) |
| Poor eye contact, *n* (%) | 28 (7.2%) | 64 (3.6%) |
| Can’t concentrate, *n* (%) | 54 (15.3%) | 88 (5.6%) |
| Can’t sit still, *n* (%) | 125 (35.4%) | 301 (19.3%) |
| Can’t wait, *n* (%) | 146 (41.4%) | 480 (30.7%) |
| Cling to adults, *n* (%) | 74 (19.0%) | 222 (12.5%) |
| Cries a lot, *n* (%) | 55 (15.6%) | 137 (8.8%) |
| Defiant, *n* (%) | 82 (21.2%) | 216 (12.2%) |
| Wants demands to be met directly, *n* (%) | 115 (29.6%) | 375 (21.0%) |
| Destroys own things, *n* (%) | 56 (15.9%) | 122 (7.8%) |
| Destroys things that belong to others, *n* (%) | 45 (12.8%) | 87 (5.6%) |
| Disobedient, *n* (%) | 42 (10.8%) | 89 (5.0%) |
| Disturbed by changes in routines, *n* (%) | 36 (10.2%) | 62 (4.0%) |
| Don’t get along with other children, *n* (%) | 15 (3.8%) | 41 (2.3%) |
| Feels no guilt, *n* (%) | 70 (18.0%) | 195 (11.0%) |
| Fights, *n* (%) | 28 (7.2%) | 65 (3.7%) |
| Sleep problems, *n* (%) | 41 (11.6%) | 96 (6.2%) |
| Hits others, *n* (%) | 54 (13.9%) | 125 (7.0%) |
| Nervous, *n* (%) | 25 (7.1%) | 38 (2.4%) |
| Overtired, *n* (%) | 17 (4.4%) | 38 (2.1%) |
| Physically attacks others, *n* (%) | 16 (4.6%) | 24 (1.5%) |
| Not sensitive to punishment, *n* (%) | 61 (15.6%) | 169 (9.5%) |
| Screams, *n* (%) | 72 (18.5%) | 215 (12.1%) |
| Stubborn, *n* (%) | 71 (18.2%) | 213 (12.0%) |
| Mood swings, *n* (%) | 57 (14.6%) | 126 (7.1%) |
| Temper tantrums, *n* (%) | 89 (22.8%) | 215 (12.1%) |
| Attention seeking, *n* (%) | 205 (52.7%) | 791 (44.4%) |
| Whiny, *n* (%) | 70 (18.0%) | 224 (12.6%) |
| **Parent-Reported Year 5 Factors (87 in total)** | | |
| Argues, *n* (%) | 113 (29.1%) | 333 (20.0%) |
| Confused, *n* (%) | 9 (2.3%) | 6 (0.4%) |
| Cruel to others, *n* (%) | 20 (5.2%) | 35 (2.1%) |
| Daydreams, *n* (%) | 20 (5.2%) | 42 (2.5%) |
| Destroys own things, *n* (%) | 45 (11.6%) | 82 (4.9%) |
| Disobedient at home, *n* (%) | 50 (12.9%) | 99 (6.0%) |
| Disobedient at school, *n* (%) | 30 (7.8%) | 27 (1.6%) |
| Accident-prone, *n* (%) | 35 (9.0%) | 89 (5.4%) |
| Get into fights, *n* (%) | 16 (4.1%) | 21 (1.3%) |
| Impulsive, *n* (%) | 45 (11.6%) | 65 (3.9%) |
| Prefers older kids, *n* (%) | 97 (25.1%) | 264 (15.9%) |
| Screams a lot, *n* (%) | 54 (13.9%) | 120 (7.2%) |
| Sets fires, *n* (%) | 5 (1.3%) | 2 (0.1%) |
| Clowns around, *n* (%) | 112 (28.9%) | 299 (18.0%) |
| Talks too much, *n* (%) | 172 (44.4%) | 566 (34.1%) |
| Unusually loud, *n* (%) | 94 (24.3%) | 226 (13.6%) |
| Can’t concentrate, *n* (%) | 69 (16.7%) | 106 (5.6%) |
| Can’t sit still, *n* (%) | 121 (29.3%) | 229 (12.2%) |
| Clings to adults, *n* (%) | 76 (18.4%) | 240 (12.8%) |
| Disobedient, *n* (%) | 44 (10.7%) | 83 (4.4%) |
| Don’t get along with other children, *n* (%) | 21 (5.1%) | 26 (1.4%) |
| No guilt, *n* (%) | 50 (12.1%) | 116 (6.2%) |
| Trouble falling asleep, *n* (%) | 42 (10.2%) | 90 (4.8%) |
| Nervous, *n* (%) | 25 (6.1%) | 41 (2.2%) |
| Stubborn, *n* (%) | 78 (18.9%) | 135 (7.2%) |
| Sudden mood swings, *n* (%) | 56 (13.6%) | 99 (5.3%) |
| Temper tantrums, *n* (%) | 83 (20.1%) | 140 (7.5%) |
| Anxious, *n* (%) | 28 (6.8%) | 64 (3.4%) |
| Wants a lot attention, *n* (%) | 184 (44.6%) | 518 (27.6%) |
| Acts too young for age, *n* (%) | 23 (5.6%) | 35 (1.9%) |
| High negative affect during MDoC, *n* (%) | 21 (8.0%) | 46 (4.2%) |
| **Teacher-Reported Year 5 Factors (18 in total)** | | |
| Seldom work to best ability, *n* (%) | 54 (38.0%) | 78 (12.7%) |
| Diagnosed disability, *n* (%) | 34 (26.8%) | 31 (5.3%) |
| Cannot easily name letters, *n* (%) | 26 (18.4%) | 53 (8.6%) |
| Cannot understand convention of print, *n* (%) | 55 (39.0%) | 112 (18.2%) |
| Cannot recognize differences in people, *n* (%) | 38 (27.5%) | 83 (13.7%) |
| Cannot sort & compare math materials, *n* (%) | 26 (18.3%) | 57 (9.3%) |
| Cannot understand relationships of quantities, *n* (%) | 35 (30.4%) | 80 (13.1%) |
| Do not show variety in solving math problems, *n* (%) | 46 (32.6%) | 102 (16.7%) |
| Below average in language & literacy, *n* (%) | 59 (41.3%) | 144 (23.4%) |
| Below average in science & social studies, *n* (%) | 38 (27.0%) | 91 (14.8%) |
| Below average in mathematical skills, *n* (%) | 60 (42.6%) | 131 (21.5%) |
| A lot more active in structured activity, *n* (%) | 26 (18.2%) | 22 (3.6%) |
| A lot more active in unstructured activity, *n* (%) | 26 (18.4%) | 28 (4.6%) |
| Have discussed problems with parent, *n* (%) | 82 (60.7%) | 182 (33.9%) |
| **Parent-Reported Year 9 Factors (113 in total)** | | |
| Easily changes between activities, *n* (%) | 165 (37.3%) | 925 (45.5%) |
| Can’t get mind off thoughts, *n* (%) | 57 (12.8%) | 64 (3.2%) |
| Rather alone than with others, *n* (%) | 19 (4.2%) | 25 (1.2%) |
| Destroys things that belong to others, *n* (%) | 21 (4.7%) | 21 (1.0%) |
| Sleeps less than others, *n* (%) | 20 (4.5%) | 23 (1.1%) |
| Understand feelings of others, *n* (%) | 217 (49.0%) | 1213 (59.8%) |
| Accepts input of friends in play, *n* (%) | 199 (44.8%) | 1203 (59.3%) |
| Acts too young for age, *n* (%) | 26 (5.8%) | 27 (1.3%) |
| Argues a lot, *n* (%) | 93 (21.0%) | 145 (7.2%) |
| Fails to finish, *n* (%) | 72 (16.3%) | 71 (3.5%) |
| Can’t concentrate, *n* (%) | 147 (33.2%) | 105 (5.2%) |
| Restless, *n* (%) | 157 (35.4%) | 104 (5.1%) |
| Clings to adults, *n* (%) | 40 (9.0%) | 84 (4.1%) |
| Feels lonely, *n* (%) | 19 (4.3%) | 37 (1.8%) |
| Confused, *n* (%) | 23 (5.2%) | 19 (0.9%) |
| Cries a lot, *n* (%) | 21 (4.7%) | 37 (1.8%) |
| Cruel to animals, *n* (%) | 13 (2.9%) | 17 (0.8%) |
| Cruel to other people, *n* (%) | 19 (4.3%) | 24 (1.2%) |
| Daydreams, *n* (%) | 40 (9.1%) | 48 (2.4%) |
| Demands attention, *n* (%) | 86 (19.3%) | 124 (6.1%) |
| Destroys own things, *n* (%) | 29 (6.5%) | 36 (1.8%) |
| Disobedient at home, *n* (%) | 33 (7.4%) | 45 (2.2%) |
| Disobedient in school, *n* (%) | 31 (7.0%) | 31 (1.5%) |
| Jealous, *n* (%) | 22 (4.9%) | 61 (3.0%) |
| Breaks rules, *n* (%) | 35 (7.8%) | 36 (1.8%) |
| Phobias, *n* (%) | 23 (5.1%) | 49 (2.4%) |
| Complains no one loves him/her, *n* (%) | 13 (2.9%) | 15 (0.7%) |
| Accident-prone, *n* (%) | 14 (3.1%) | 23 (1.1%) |
| Gets into many fights, *n* (%) | 15 (3.3%) | 14 (0.7%) |
| Gets teased, *n* (%) | 21 (4.7%) | 22 (1.1%) |
| Impulsive, *n* (%) | 47 (10.5%) | 28 (1.4%) |
| Lies, *n* (%) | 18 (4.0%) | 26 (1.3%) |
| Nervous, *n* (%) | 15 (3.4%) | 21 (1.0%) |
| Nervous movements, *n* (%) | 13 (2.9%) | 12 (0.6%) |
| Nightmares, *n* (%) | 9 (2.0%) | 9 (0.4%) |
| Anxious, *n* (%) | 12 (2.7%) | 15 (0.7%) |
| Poor in school, *n* (%) | 17 (3.8%) | 31 (1.5%) |
| Compulsions, *n* (%) | 17 (3.8%) | 21(1.0%) |
| Screams, *n* (%) | 23 (5.1%) | 38 (1.9%) |
| Secretive, *n* (%) | 17 (3.8%) | 22 (1.1%) |
| Self-embarrassed, *n* (%) | 27 (6.0%) | 54 (2.7%) |
| Clowns, *n* (%) | 41 (9.2%) | 65 (3.2%) |
| Easily distracted, *n* (%) | 108 (24.2%) | 86 (4.2%) |
| Hoards, *n* (%) | 26 (5.8%) | 60 (2.9%) |
| Stubborn, *n* (%) | 30 (6.7%) | 41 (2.0%) |
| Mood swings, *n* (%) | 26 (5.8%) | 27 (1.3%) |
| Talks too much, *n* (%) | 75 (16.9%) | 143 (7.0%) |
| Teases a lot, *n* (%) | 14 (3.1%) | 21 (1.0%) |
| Temper tantrums, *n* (%) | 58 (13.0%) | 59 (2.9%) |
| Troubles sleeping, *n* (%) | 25 (5.6%) | 21 (1.0%) |
| Unusually loud, *n* (%) | 24 (5.4%) | 47 (2.3%) |
| Whines, *n* (%) | 40 (9.1%) | 56 (2.7%) |
| Withdrawn, *n* (%) | 7 (1.6%) | 7 (0.3%) |
| Sympathetic to others, *n* (%) | 204 (46.2%) | 1152 (57.0%) |
| Makes friends easily, *n* (%) | 222 (49.9%) | 1329 (65.3%) |
| Self-confident, *n* (%) | 168 (37.8%) | 1008 (49.6%) |
| Interested in different things, *n* (%) | 230 (51.8%) | 1316 (64.5%) |
| Liked by others, *n* (%) | 259 (58.3%) | 1526 (75.1%) |
| Invites others’ home, *n* (%) | 213 (47.8%) | 1141 (56.0%) |
| Reports accidents to adults appropriately, *n* (%) | 231 (51.8%) | 1359 (66.7%) |
| **Teacher-Reported Year 9 Factors (83 in total)** | | |
| A lot more active in structured activity, *n* (%) | 35 (11.7%) | 57 (4.2%) |
| A lot more active in unstructured activity, *n* (%) | 41 (13.6%) | 62 (4.6%) |
| Only attention in own interests | 145 (48.2%) | 280 (20.6%) |
| Invites others in activities | 155 (51.7%) | 959 (70.6%) |
| Below average in language & literacy, *n* (%) | 52 (17.4%) | 99 (7.3%) |
| Below average in science & social studies, *n* (%) | 31 (10.4%) | 63 (4.7%) |
| Below average in mathematical skills, *n* (%) | 45 (14.9%) | 99 (7.3%) |
| Can control temper, *n* (%) | 153 (50.8%) | 1078 (79.2%) |
| Compromises in conflict, *n* (%) | 101 (33.6%) | 769 (57.1%) |
| Responds adequately to peer pressure, *n* (%) | 118 (39.9%) | 970 (71.8%) |
| Says nice things about oneself, *n* (%) | 131 (44.0%) | 919 (67.7%) |
| Uses free time appropriately, *n* (%) | 138 (45.8%) | 1027 (75.5%) |
| Finishes tasks in time, *n* (%) | 119 (39.4%) | 956 (70.2%) |
| Makes friends easily, *n* (%) | 125 (41.7%) | 1028 (75.4%) |
| Responds appropriately to teasing, *n* (%) | 84 (27.9%) | 809 (60.2%) |
| Receives critique well, *n* (%) | 100 (33.4%) | 901 (66.4%) |
| Can wait for help, *n* (%) | 80 (26.6%) | 841 (61.8%) |
| Produces correct schoolwork, *n* (%) | 132 (44.0%) | 957 (70.4%) |
| Accepts ideas from peers, *n* (%) | 135 (45.0%) | 1036 (76.2%) |
| Gives compliments, *n* (%) | 111 (36.9%) | 821 (60.5%) |
| Follows directions, *n* (%) | 150 (49.7%) | 1077 (79.1%) |
| Puts away school material adequately, *n* (%) | 160 (53.2%) | 1092 (80.1%) |
| Cooperates with peers, *n* (%) | 132 (43.9%) | 1065 (78.3%) |
| Joins groups spontaneously, *n* (%) | 129 (42.9%) | 957 (70.3%) |
| Responds adequately when pushed, *n* (%) | 102 (34.1%) | 883 (66.2%) |
| Ignores distraction from peers in class, *n* (%) | 44 (14.7%) | 615 (45.2%) |
| Clean desk, *n* (%) | 85 (28.3%) | 822 (60.6%) |
| Attends to instructions, *n* (%) | 124 (41.2%) | 1013 (74.7%) |
| Transits easily between activities, *n* (%) | 118 (39.3%) | 1018 (74.9%) |
| Gets along with different people, *n* (%) | 165 (55.0%) | 1085 (80.0%) |
| Maintains friendships, *n* (%) | 243 (91.7%) | 793 (97.1%) |
| Works independently, *n* (%) | 240 (88.6%) | 804 (94.5%) |
| Adopts well to change in routines, *n* (%) | 233 (87.6%) | 829 (95.2%) |
| Organized belongings, *n* (%) | 192 (70.1%) | 744 (81.6%) |
| Fights with others, *n* (%) | 192 (66.9%) | 551 (41.3%) |
| Low self-esteem, *n* (%) | 221 (78.9%) | 659 (50.5%) |
| Bullies others, *n* (%) | 145 (50.9%) | 322 (24.3%) |
| Appears lonely, *n* (%) | 172 (62.8%) | 515 (38.8%) |
| Anxious when in a group of children, *n* (%) | 145 (49.3%) | 293 (21.7%) |
| Argues with others, *n* (%) | 210 (79.8%) | 726 (56.1%) |
| Talks back to adults, *n* (%) | 157 (56.5%) | 337 (25.8%) |
| Gets angry easily, *n* (%) | 177 (67.0%) | 430 (33.1%) |
| Temper tantrums, *n* (%) | 124 (44.8%) | 229 (17.3%) |
| Likes being alone, *n* (%) | 190 (67.4%) | 698 (52.3%) |
| Sad, *n* (%) | 174 (61.1%) | 507 (37.9%) |
| Inattentive, *n* (%) | 209 (69.4%) | 408 (30.1%) |
| Defiant, *n* (%) | 90 (29.9%) | 122 (9.0%) |
| Restless, *n* (%) | 138 (46.2%) | 201 (14.8%) |
| Forgets what he/she has learned, *n* (%) | 114 (38.3%) | 284 (20.9%) |
| Disturbs others, *n* (%) | 128 (42.7%) | 198 (14.6%) |
| Defies adults, *n* (%) | 75 (24.9%) | 101 (7.4%) |
| Always on the go, *n* (%) | 90 (29.9%) | 121 (8.9%) |
| Poor spelling, *n* (%) | 124 (41.6%) | 345 (25.4%) |
| Cannot remain still, *n* (%) | 113 (37.5%) | 138 (10.1%) |
| Spiteful, *n* (%) | 48 (16.1%) | 87 (6.4%) |
| Leaves seat, *n* (%) | 113 (37.5%) | 132 (9.7%) |
| Fidgety with hands, *n* (%) | 114 (38.3%) | 153 (11.3%) |
| Poor in reading, *n* (%) | 137 (45.8%) | 372 (27.4%) |
| Short attention span, *n* (%) | 162 (53.8%) | 273 (20.1%) |
| Argues with adults, *n* (%) | 74 (24.7%) | 112 (8.2%) |
| Difficulties waiting his/her turn, *n* (%) | 110 (36.8%) | 137 (10.1%) |
| No interest in school, *n* (%) | 97 (32.3%) | 222 (16.3%) |
| Distractable, *n* (%) | 168 (56.0%) | 292 (21.5%) |
| Temper outbursts, *n* (%) | 85 (28.3%) | 85 (6.3%) |
| Runs/climbs around, *n* (%) | 39 (13.0%) | 36 (2.6%) |
| Poor in arithmetic, *n* (%) | 112 (37.3%) | 328 (24.3%) |
| Intrudes others, *n* (%) | 111 (36.9%) | 147 (10.8%) |
| Difficulties playing quietly, *n* (%) | 88 (29.3%) | 106 (7.8%) |
| Fails to finish tasks, *n* (%) | 120 (40.0%) | 245 (18.1%) |
| Does not follow instructions, *n* (%) | 110 (36.8%) | 251 (18.5%) |
| Excitable, *n* (%) | 130 (43.3%) | 141 (10.3%) |
| Always on the go, *n* (%) | 99 (32.9%) | 112 (8.2%) |
| Repeated grade, *n* (%) | 19 (6.4%) | 51 (3.8%) |
| **Self-Reported Year 9 Factors (52 in total)** | | |
| Don’t feel close to people at school, *n* (%) | 79 (18.1%) | 250 (12.3%) |
| Never wants to solve hard tasks, *n* (%) | 49 (11.3%) | 114 (5.6%) |
| Angry when trouble with learning, *n* (%) | 89 (20.3%) | 240 (11.7%) |
| Never helps at home, *n* (%) | 77 (17.8%) | 215 (10.5%) |
| Picked on in school, *n* (%) | 136 (31.1%) | 417 (20.3%) |
| Beaten in school, *n* (%) | 48 (10.9%) | 119 (5.8%) |
| Left out in school, *n* (%) | 51 (11.8%) | 144 (7.0%) |
| Damaged property, *n* (%) | 93 (21.1%) | 270 (13.2%) |
| Stolen something, *n* (%) | 84 (19.1%) | 183 (8.9%) |
| Gotten into fist fight, *n* (%) | 201 (45.5%) | 579 (28.1%) |
| Run away from home, *n* (%) | 23 (5.2%) | 40 (1.9%) |
| Suspended at school, *n* (%) | 145 (32.9%) | 311 (15.1%) |
| Graffiti, *n* (%) | 34 (7.7%) | 53 (2.6%) |
| Set fire, *n* (%) | 19 (4.3%) | 21 (1.0%) |
| Thrown rocks at people or cars, *n* (%) | 43 (9.7%) | 97 (4.7%) |
| Never orderly, *n* (%) | 54 (12.3%) | 145 (7.1%) |
| Not doing my best, *n* (%) | 21 (4.8%) | 35 (1.7%) |
| Don’t follow through, *n* (%) | 57 (13.1%) | 148 (7.2%) |
| Argue with others, *n* (%) | 72 (16.4%) | 188 (9.2%) |
| Hard paying attention, *n* (%) | 104 (23.7%) | 225 (10.9%) |
| Feeling lonely, *n* (%) | 76 (17.3%) | 177 (8.6%) |
| Easily distracted, *n* (%) | 162 (36.8%) | 395 (19.2%) |
| Sad, *n* (%) | 59 (13.4%) | 157 (7.7%) |
| Hard to finish schoolwork, *n* (%) | 88 (20.1%) | 172 (8.4%) |
| Worry about no one to play with, *n* (%) | 113 (25.7%) | 328 (16.0%) |
| Disturbing others, *n* (%) | 120 (27.4%) | 343 (16.7%) |
| Fighting with others, *n* (%) | 102 (23.2%) | 270 (13.2%) |
| Seldom wear seatbelt, *n* (%) | 37 (8.4%) | 87 (4.2%) |
| **Performance-based factors (8 in total)** |  |  |
| Vocabulary year 5, *M* (*SD*) | 58.58 (20.25) | 62.78 (19.05) |
| Impulsivity year 5, *M* (*SD*) | 9.22 (3.01) | 10.27 (2.73) |
| Attention year 5, *M* (*SD*) | 12.09 (3.32) | 12.99 (3.18) |
| Vocabulary year 9, *M* (*SD*) | 107.95 (20.48) | 112.11 (20.33) |
| Language year 9, *M* (*SD*) | 23.58 (6.19) | 26.00 (5.49) |
| Math skills year 9, *M* (*SD*) | 30.08 (6.28) | 32.68 (6.15) |
| Working memory year 9, *M* (*SD*) | 13.00 (3.09) | 14.06 (3.12) |

Notes. MDoC = The Maternal Description of Child.

**Table S2.** Variables in the training set for which adolescents with ASD differed from their peers while accounting for differences in sex

|  | ASD  *n* = 76 | Neither ADHD nor ASD  *n* = 3483 |
| --- | --- | --- |
| Boys, *n* (%) | 61 (80.3%) | 1123 (47.6%) |
| **Pregnancy and birth (4 variables in total)** | | |
| *No statistically significant differences* |  |  |
| **Family presence of psychiatric disorders (20 in total)** | | |
| Clinical anxiety in mother’s parents, *n* (%) | 22 (43.1%) | 340 (20.4%) |
| Mother depressed at year 1, *n* (%) | 19 (26.4%) | 251 (11.3%) |
| **Physical Health Factors (43 in total)** | | |
| Overnight stay in hospital year 1, *n* (%) | 21 (29.6%) | 318 (14.3%) |
| Physical disability year 3, *n* (%) | 9 (15.5%) | 41 (2.3%) |
| Over two ear infections year 5, *n* (%) | 10 (18.9%) | 70 (4.1%) |
| Over two ear infections year 9 *n* (%) | 6 (8.8%) | 51 (2.3%) |
| Speech problems year 3, *n* (%) | 22 (41.5%) | 72 (4.5%) |
| Speech problems year 5, *n* (%) | 23 (37.1%) | 114 (6.0%) |
| Speech problems year 9, *n* (%) | 37 (54.4%) | 162 (7.3%) |
| Stuttering year 9, *n* (%) | 8 (11.8%) | 66 (3.0%) |
| Unknown physical pains, *n* (%) | 3 (4.7%) | 18 (0.9%) |
| Constipated year 9, *n* (%) | 5 (7.8%) | 15 (0.7%) |
| Height percentile year 9, *M* (*SD*) | 51.35 (35.37) | 58.25 (29.84) |
| **Parent-Reported Year 1 Factors (7 in total)** | | |
| *No statistically significant differences* |  |  |
| **Parent-Reported Year 3 Factors (67 in total)** | | |
| Secure attachment, *n* (%) | 22 (57.9%) | 982 (77.9%) |
| Plays with others | 34 (64.2%) | 1278 (80.5%) |
| Enjoys talking with parents | 40 (75.5%) | 1430 (89.9%) |
| Acts too young for age | 10 (17.2%) | 22 (1.2%) |
| Poor eye contact | 12 (20.7%) | 70 (3.9%) |
| Can’t concentrate | 85 (5.3%) | 16 (30.2%) |
| Can’t sit still | 24 (45.3%) | 313 (19.7%) |
| Can’t wait | 29 (54.7%) | 489 (30.7%) |
| Defiant | 19 (33.3%) | 221 (12.3%) |
| Wants demands to be met directly, *n* (%) | 27 (46.6%) | 375 (20.7%) |
| Destroys own things, *n* (%) | 11 (20.8%) | 120 (7.5%) |
| Destroys things that belong to others, *n* (%) | 7 (13.2%) | 89 (4.9%) |
| Disobedient, *n* (%) | 9 (15.5%) | 89 (5.0%) |
| Disturbed by changes in routines, *n* (%) | 11 (20.8%) | 69 (4.3%) |
| Don’t respond to talk | 10 (17.2%) | 97 (9.7%) |
| Feels no guilt, *n* (%) | 17 (29.8%) | 198 (11.0%) |
| Easily frustrated, *n* (%) | 22 (37.9%) | 242 (13.4%) |
| Angry mood, *n* (%) | 14 (24.1%) | 160 (8.8%) |
| Nervous, *n* (%) | 6 (11.3%) | 34 (2.1%) |
| Overtired, *n* (%) | 7 (12.1%) | 41 (2.3%) |
| Physically attacks others, *n* (%) | 5 (9.4%) | 26 (1.6%) |
| Not sensitive to punishment, *n* (%) | 15 (25.9%) | 157 (8.7%) |
| Temper tantrums, *n* (%) | 17 (29.3%) | 210 (11.6%) |
| Withdrawn, *n* (%) | 5 (8.6%) | 28 (1.5%) |
| **Parent-Reported Year 5 Factors (87 in total)** | | |
| Argues, *n* (%) | 21 (38.9%) | 337 (19.8%) |
| Confused, *n* (%) | 4 (7.4%) | 5 (0.3%) |
| Cruel to others, *n* (%) | 5 (9.3%) | 35 (2.1%) |
| Daydreams, *n* (%) | 6 (11.1%) | 46 (2.7%) |
| Destroys own things, *n* (%) | 9 (16.7%) | 90 (5.3%) |
| Destroys things that belong to others, *n* (%) | 7 (13.0%) | 65 (3.8%) |
| Sympathy with others, *n* (%) | 22 (40.7%) | 1046 (61.6%) |
| Disobedient at home, *n* (%) | 9 (16.7%) | 99 (5.8%) |
| Disobedient at school, *n* (%) | 5 (10.0%) | 27 (1.6%) |
| Accident-prone, *n* (%) | 8 (14.8%) | 82 (4.8%) |
| Get into fights, *n* (%) | 4 (7.4%) | 21 (1.2%) |
| Impulsive, *n* (%) | 11 (20.4%) | 66 (3.9%) |
| Rather be alone, *n* (%) | 11 (25.9%) | 48 (2.8%) |
| Nervous twitches, *n* (%) | 7 (13.0%) | 12 (0.7%) |
| Joins others in play, *n* (%) | 25 (46.3%) | 1244 (73.1%) |
| Attacks people, *n* (%) | 3 (5.6%) | 15 (0.9%) |
| Clumsy, *n* (%) | 6 (11.1%) | 25 (1.5%) |
| Wants to play with others, *n* (%) | 24 (44.4%) | 1223 (71.9%) |
| Plays and talks with others, *n* (%) | 28 (51.9%) | 1364 (80.1%) |
| Threatens people, *n* (%) | 4 (7.4%) | 14 (0.8%) |
| Unusually loud, *n* (%) | 15 (27.8%) | 230 (13.5%) |
| Interested in different things, *n* (%) | 36 (66.7%) | 1385 (81.5%) |
| Enjoys talking with you, *n* (%) | 44 (81.5%) | 1583 (93.0%) |
| Can’t concentrate, *n* (%) | 20 (32.3%) | 107 (5.6%) |
| Can’t sit still, *n* (%) | 25 (40.3%) | 216 (11.3%) |
| Clings to adults, *n* (%) | 18 (29.0%) | 243 (12.8%) |
| Cries a lot, *n* (%) | 15 (24.2%) | 136 (7.1%) |
| Disobedient, *n* (%) | 12 (19.4%) | 78 (4.1%) |
| Don’t get along with other children, *n* (%) | 8 (12.9%) | 31 (1.6%) |
| Trouble falling asleep, *n* (%) | 14 (22.6%) | 96 (5.0%) |
| Nervous, *n* (%) | 12 (19.4%) | 41 (2.2%) |
| Stubborn, *n* (%) | 23 (37.1%) | 142 (7.5%) |
| Sudden mood swings, *n* (%) | 18 (29.0%) | 99 (5.2%) |
| Temper tantrums, *n* (%) | 17 (27.4%) | 145 (7.6%) |
| Anxious, *n* (%) | 11 (17.7%) | 73 (3.8%) |
| Sad, *n* (%) | 3 (4.8%) | 7 (0.4%) |
| Wants a lot of attention, *n* (%) | 31 (50.0%) | 526 (27.6%) |
| Withdrawn from other children, *n* (%) | 8 (13.1%) | 23 (1.2%) |
| Feels worthless, *n* (%) | 2 (3.3%) | 5 (0.3%) |
| Acts too young for age, *n* (%) | 18 (29.5%) | 42 (2.2%) |
| **Teacher-Reported Year 5 Factors (18 in total)** | | |
| Diagnosed disability, *n* (%) | 10 (66.7%) | 41 (7.0%) |
| Cannot understand convention of print, *n* (%) | 12 (75.0%) | 123 (20.2%) |
| Cannot sort & compare math materials, *n* (%) | 6 (37.5%) | 62 (27.4%) |
| Do not show variety in solving math problems, *n* (%) | 8 (50.0%) | 112 (18.4%) |
| Below average in language & literacy, *n* (%) | 11 (68.8%) | 148 (24.2%) |
| Below average in mathematical skills, *n* (%) | 9 (56.2%) | 138 (22.8%) |
| Have discussed problems with parents, *n* (%) | 11 (73.3%) | 178 (33.5%) |
| **Parent-Reported Year 9 Factors (113 in total)** | | |
| Easily changes between activities, *n* (%) | 14 (21.9%) | 948 (46.6%) |
| Can’t get mind off thoughts, *n* (%) | 16 (25.0%) | 66 (3.2%) |
| Rather alone than with others, *n* (%) | 10 (15.9%) | 25 (1.2%) |
| Plays with sex parts, *n* (%) | 4 (6.2%) | 2 (0.1%) |
| Sleeps less than others, *n* (%) | 8 (12.5%) | 20 (1.0%) |
| Understand feelings of others, *n* (%) | 26 (40.6%) | 1215 (59.9%) |
| Accepts input from friends in play, *n* (%) | 11 (17.5%) | 1237 (60.7%) |
| Acts too young for age, *n* (%) | 14 (21.5%) | 27 (1.3%) |
| Drinks alcohol, *n* (%) | 3 (4.7%) | 14 (0.7%) |
| Fails to finish, *n* (%) | 8 (12.5%) | 72 (3.6%) |
| Can’t concentrate, *n* (%) | 19 (29.2%) | 103 (5.1%) |
| Restless, *n* (%) | 25 (38.5%) | 107 (5.3%) |
| Clings to adults, *n* (%) | 13 (20.0%) | 90 (4.4%) |
| Feels lonely, *n* (%) | 5 (7.8%) | 37 (1.8%) |
| Confused, *n* (%) | 7 (10.9%) | 19 (0.9%) |
| Cries a lot, *n* (%) | 6 (9.2%) | 34 (1.7%) |
| Cruel to animals, *n* (%) | 3 (4.6%) | 18 (0.9%) |
| Daydreams, *n* (%) | 12 (18.8%) | 46 (2.3%) |
| Self-harm, *n* (%) | 3 (4.6%) | 13 (0.6%) |
| Demands attention, *n* (%) | 17 (26.2%) | 123 (6.0%) |
| Destroys own things, *n* (%) | 6 (9.4%) | 35 (1.7%) |
| Phobias, *n* (%) | 10 (16.1%) | 38 (1.9%) |
| Accident-prone, *n* (%) | 6 (9.4%) | 19 (0.9%) |
| Gets teased, *n* (%) | 9 (14.1%) | 28 (1.4%) |
| Impulsive, *n* (%) | 10 (15.9%) | 26 (1.3%) |
| Nervous, *n* (%) | 8 (12.5%) | 18 (0.9%) |
| Nervous movements, *n* (%) | 3 (4.7%) | 10 (0.5%) |
| Nightmares, *n* (%) | 3 (4.8%) | 9 (0.4%) |
| Not liked by other kids, *n* (%) | 5 (7.8%) | 19 (0.9%) |
| Anxious, *n* (%) | 8 (12.5%) | 15 (0.7%) |
| Attacks others physically, *n* (%) | 3 (4.7%) | 11 (0.5%) |
| Picks nose/skin, *n* (%) | 4 (6.2%) | 25 (1.2%) |
| Clumsy, *n* (%) | 6 (9.2%) | 14 (0.7%) |
| Prefers younger kids, *n* (%) | 6 (9.5%) | 38 (1.9%) |
| Compulsions, *n* (%) | 12 (18.8%) | 16 (0.8%) |
| Screams, *n* (%) | 5 (7.7%) | 31 (1.5%) |
| Easily distracted, *n* (%) | 18 (27.7%) | 85 (4.2%) |
| Stares blankly, *n* (%) | 4 (6.2%) | 13 (0.6%) |
| Strange behaviors, *n* (%) | 5 (7.8%) | 7 (0.3%) |
| Temper tantrums, *n* (%) | 8 (12.3%) | 50 (2.4%) |
| Troubles sleeping, *n* (%) | 8 (12.3%) | 19 (0.9%) |
| Vandalizes, *n* (%) | 2 (3.1%) | 3 (0.1%) |
| Whines, *n* (%) | 10 (15.6%) | 53 (2.6%) |
| Withdrawn, *n* (%) | 4 (6.2%) | 6 (0.3%) |
| Worries, *n* (%) | 10 (15.9%) | 64 (3.2%) |
| Joins groups when told so, *n* (%) | 10 (15.4%) | 871 (42.8%) |
| Makes friends easily, *n* (%) | 17 (26.2%) | 1350 (66.3%) |
| Self-confident, *n* (%) | 8 (12.7%) | 1040 (51.1%) |
| Interested in different things, *n* (%) | 20 (30.8%) | 1327 (65.0%) |
| Liked by others, *n* (%) | 26 (40.0%) | 1532 (75.2%) |
| Invites others’ home, *n* (%) | 16 (25.0%) | 1146 (56.2%) |
| Reports accidents to adults appropriately, *n* (%) | 22 (34.9%) | 1380 (67.6%) |
| **Teacher-Reported Year 9 Factors (83 in total)** | | |
| Only attention in own interests, *n* (%) | 23 47.9%) | 277 (20.0%) |
| Invites others in activities, *n* (%) | 20 (43.5%) | 980 (70.8%) |
| Below average in language & literacy, *n* (%) | 12 (25.0%) | 104 (7.5%) |
| Below average in science & social studies, *n* (%) | 17 (35.4%) | 70 (5.1%) |
| Below average in mathematical skills, *n* (%) | 15 (31.2%) | 103 (7.5%) |
| Compromises in conflict, *n* (%) | 15 (31.9%) | 775 (56.3%) |
| Responds adequately to peer pressure, *n* (%) | 19 (42.2%) | 994 (72.1%) |
| Makes friends easily, *n* (%) | 14 (30.4%) | 1044 (75.2%) |
| Responds appropriately to teasing, *n* (%) | 9 (19.1%) | 825 (60.1%) |
| Controls temper, *n* (%) | 25 (53.2%) | 1101 (79.8%) |
| Receives critique well, *n* (%) | 11 (24.4%) | 915 (66.1%) |
| Can wait for help, *n* (%) | 14 (30.4%) | 843 (60.8%) |
| Accepts ideas from peers, *n* (%) | 21 (45.7%) | 1062 (76.6%) |
| Cooperates with peers, *n* (%) | 22 (46.8%) | 1089 (78.5%) |
| Joins groups spontaneously, *n* (%) | 23 (47.9%) | 992 (71.5%) |
| Responds adequately when pushed, *n* (%) | 18 (39.1%) | 893 (65.4%) |
| Clean desk, *n* (%) | 12 (26.7%) | 829 (59.9%) |
| Transits easily between activities, *n* (%) | 22 (46.8%) | 1040 (74.9%) |
| Gets along with different people, *n* (%) | 25 (54.3%) | 1107 (80.0%) |
| Expresses own feelings, *n* (%) | 24 (52.2%) | 1057 (76.3) |
| Low self-esteem, *n* (%) | 34 (77.3%) | 676 (51.0%) |
| Appears lonely, *n* (%) | 28 (65.1%) | 534 (39.5%) |
| Anxious when in a group of children, *n* (%) | 28 (63.6%) | 324 (23.5%) |
| Argues with others, *n* (%) | 37 (86.0%) | 732 (55.4%) |
| Talks back to adults, *n* (%) | 26 (59.1%) | 348 (26.2%) |
| Gets angry easily, *n* (%) | 34 (73.9%) | 453 (34.1%) |
| Temper tantrums, *n* (%) | 24 (52.2%) | 237 (17.6%) |
| Likes being alone, *n* (%) | 34 (81.0%) | 728 (53.5%) |
| Inattentive, *n* (%) | 29 (60.4%) | 431 (31.1%) |
| Restless, *n* (%) | 18 (37.5%) | 201 (14.5%) |
| Disturbs others, *n* (%) | 15 (31.2%) | 192 (13.8%) |
| Cannot remain still, *n* (%) | 14 (29.8%) | 140 (10.1%) |
| Leaves seat, *n* (%) | 13 (27.1%) | 133 (9.6%) |
| Fidgety with hands, *n* (%) | 18 (40.0%) | 154 (11.1%) |
| Poor in reading, *n* (%) | 27 (57.4%) | 388 (28.0%) |
| Short attention span, *n* (%) | 27 (56.2%) | 286 (20.6%) |
| Difficulties waiting his/her turn, *n* (%) | 17 (36.2%) | 136 (9.8%) |
| Distractable, *n* (%) | 26 (54.2%) | 298 (21.5%) |
| Temper outbursts, *n* (%) | 10 (20.8%) | 88 (6.4%) |
| Intrudes others, *n* (%) | 15 (31.2%) | 138 (9.9%) |
| Difficulties playing quietly, *n* (%) | 13 (27.1%) | 105 (7.6%) |
| Fails to finish tasks, *n* (%) | 20 (42.6%) | 257 (18.6%) |
| Excitable, *n* (%) | 17 (36.2%) | 136 (9.8%) |
| Always on the go, *n* (%) | 12 (25.0%) | 111 (8.0%) |
| **Self-Reported Year 9 Factors (52 in total)** | | |
| Angry when trouble learning, *n* (%) | 13 (26.5%) | 261 (12.7%) |
| Never helps at home, *n* (%) | 13 (25.5%) | 216 (10.5%) |
| TV more than 4 hours per day, *n* (%) | 10 (19.2%) | 639 (31.1%) |
| Picked on in school, *n* (%) | 19 (38.0%) | 423 (20.6%) |
| Have run away from home, *n* (%) | 5 (9.6%) | 39 (1.9%) |
| Set fire, *n* (%) | 4 (7.7%) | 21 (1.0%) |
| Not doing my best, *n* (%) | 5 (9.8%) | 32 (1.6%) |
| Hard paying attention, *n* (%) | 12 (24.0%) | 218 (10.6%) |
| Easily distracted, *n* (%) | 20 (40.0%) | 399 (19.4%) |
| Seldom wear seatbelt, *n* (%) | 8 (15.7%) | 84 (4.1%) |
| **Performance-based factors (8 in total)** |  |  |
| Vocabulary year 5, *M* (*SD*) | 51.46 (22.16) | 62.58 (19.05) |
| Impulsivity year 5, *M* (*SD*) | 9.48 (3.59) | 10.16 (2.84) |
| Attention year 5, *M* (*SD*) | 10.33 (3.19) | 12.95 (3.27) |
| Vocabulary year 9, *M* (*SD*) | 104.90 (28.96) | 111.97 (20.09) |
| Language year 9, *M* (*SD*) | 22.14 (8.13) | 26.01 (5.44) |
| Math skills year 9, *M* (*SD*) | 26.86 (9.40) | 32.65 (5.92) |
| Working memory year 9, *M* (*SD*) | 11.33 (4.18) | 14.10 (3.09) |
