# Supplementary material for: Developmental signs of ADHD and autism: a prospective investigation in 3623 children
Source: Eur Child Adolesc Psychiatry. 2022 Jun 24;32(10):1969–78. doi: 10.1007/s00787-022-02024-4 (PMC10533573; doi:10.1007/s00787-022-02024-4)
Supplement: Supplementary file 2 — Supplementary file2 (DOCX 78 KB) [file 787_2022_2024_MOESM2_ESM.docx]

library(dplyr)

library(ggplot2)

library(readxl)

library(summarytools)

library(gmodels)

library(pscl)

library(dominanceanalysis)

library(caret)

library(randomForest)

library(varImp)

library(logistf)

library(devtools)

library(VariableSelection)

library(olsrr)

library(glmnet)

library(ROSE)

library(rpart)

library(dplyr)

library(tidyr)

library(mice)

library(miceadds)

library(leaps)

library(bestglm)

setwd("/Users/matticervin/Dropbox/Artiklar/Fragile Families and Child Wellbeing Study")

### Data to impute for accuracy checks

imp <- read_excel("Data/imp.xlsx")

impadhd <- subset(imp, adhdinclude==1)

impasd <- subset(imp, autisminclude==1)

impadhd <- subset(impadhd, select=c(3,5:504))

impasd <- subset(impasd, select=c(4,5:504))

## Impute data --- this is stored in external object, see below

initial1<-mice(impadhd, maxit=0, print=F, defaultMethod = "pmm")

initial1$method #PMM

initial1$predictorMatrix #Looks ok

dfimpadhd<-mice(impadhd, m=5, maxit=10, seed=1234, meth=initial1$method,

pred=initial1$predictorMatrix)

write.mice.imputation(mi.res=dfimpadhd, name="impadhd", mids2spss=F)

### And for ASD

initial1<-mice(impasd, maxit=0, print=F, defaultMethod = "pmm")

initial1$method #PMM

initial1$predictorMatrix #Looks ok

dfimpasd<-mice(impasd, m=5, maxit=10, seed=1234, meth=initial1$method,

pred=initial1$predictorMatrix)

write.mice.imputation(mi.res=dfimpasd, name="impasd", mids2spss=F)

#### #### #### #### #### #### #### #### #### #### ####

#### Data for first stages of analysis

#### #### #### #### #### #### #### #### #### #### ####

neurodev_finalclear <- read_excel("Data/neurodev_finalclear.xlsx")

df <- subset(neurodev_finalclear, adhdinclude==1)

df2 <- subset(neurodev_finalclear, autisminclude==1)

### Cases of ADHD and ASD

freq(df$adhd)

# Freq % Valid % Valid Cum. % Total % Total Cum.

#----------- ------ --------- -------------- --------- --------------

# 0 2952 82.48 82.48 81.64 81.64

# 1 627 17.52 100.00 17.34 98.98

# Tot 3579 100.00 100.00 100.00 100.00

freq(df2$autism)

# Freq % Valid % Valid Cum. % Total % Total Cum.

#----------- ------ --------- -------------- --------- --------------

#0 2954 97.01 97.01 97.01 97.01

#1 91 2.99 100.00 2.99 100.00

#Tot 3045 100.00 100.00 100.00 100.00

# Create exploratory and holdout sets

## 20% vs 80% of the sample

##ADHD

smp_size <- floor(0.2 * nrow(df))

## set the seed to make partition reproducible

set.seed(1234)

twentyperc <- sample(seq_len(nrow(df)), size = smp_size)

holdout <- df[twentyperc, ]

expl <- df[-twentyperc, ]

### ASD

smp_size2 <- floor(0.2 * nrow(df2))

## set the seed to make partition reproducible

set.seed(1234)

twentyperc2 <- sample(seq_len(nrow(df2)), size = smp_size2)

holdout2 <- df2[twentyperc2, ]

expl2 <- df2[-twentyperc2, ]

freq(expl$adhd)

# Freq % Valid % Valid Cum. % Total % Total Cum.

#----------- ------ --------- -------------- --------- --------------

#0 2353 82.16 82.16 82.16 82.16

#1 511 17.84 100.00 17.84 100.00

freq(holdout$adhd)

# Freq % Valid % Valid Cum. % Total % Total Cum.

#----------- ------ --------- -------------- --------- --------------

#0 599 83.78 83.78 83.78 83.78

#1 116 16.22 100.00 16.22 100.00

freq(expl2$autism)

# Freq % Valid % Valid Cum. % Total % Total Cum.

#----------- ------ --------- -------------- --------- --------------

#0 2360 96.88 96.88 96.88 96.88

#1 76 3.12 100.00 3.12 100.00

freq(holdout2$autism)

# Freq % Valid % Valid Cum. % Total % Total Cum.

#----------- ------ --------- -------------- --------- --------------

#0 594 97.54 97.54 97.54 97.54

#1 15 2.46 100.00 2.46 100.00

####################################

######## Comparisons

####################################

CrossTable(expl$adhd, expl$male)

### Early predictors

rpregnancy_drugs <- glm(adhd ~ male + pregnancy_drugs, data = expl, family = "binomial")

rpregnancy_drink <- glm(adhd ~ male + pregnancy_drink, data = expl, family = "binomial")

rpregnancy_smoke <- glm(adhd ~ male + pregnancy_smoke, data = expl, family = "binomial")

rbaseline_lowbirthweight <- glm(adhd ~ male + baseline_lowbirthweight, data = expl, family = "binomial")

rnotwalk14mnths <- glm(adhd ~ male + notwalk14mnths, data = expl, family = "binomial")

rbaseline_mothersage <- glm(adhd ~ male + baseline_mothersage, data = expl, family = "binomial")

rbaseline_fatherinjail <- glm(adhd ~ male + baseline_fatherinjail, data = expl, family = "binomial")

summary(rpregnancy_drugs)

summary(rpregnancy_drink)

summary(rpregnancy_smoke)#***

summary(rbaseline_lowbirthweight)#*

summary(rnotwalk14mnths)

summary(rbaseline_mothersage)

summary(rbaseline_fatherinjail)#**

CrossTable(expl$adhd, expl$pregnancy_smoke)

CrossTable(expl$adhd, expl$baseline_lowbirthweight)

CrossTable(expl$adhd, expl$baseline_fatherinjail)

### Psychiatric problems in family (grandmothers and grandfathers)

rfampsych_dep_fside <- glm(adhd ~ male + fampsych_dep_fside, data = expl, family = "binomial")

rfampsych_anx_fside <- glm(adhd ~ male + fampsych_anx_fside, data = expl, family = "binomial")

rfampsych_substance_fside <- glm(adhd ~ male + fampsych_substance_fside, data = expl, family = "binomial")

rfampsych_sucatt_fside <- glm(adhd ~ male + fampsych_sucatt_fside, data = expl, family = "binomial")

rfampsych_dep_mside <- glm(adhd ~ male + fampsych_dep_mside, data = expl, family = "binomial")

rfampsych_anx_mside <- glm(adhd ~ male + fampsych_anx_mside, data = expl, family = "binomial")

rfampsych_substance_mside <- glm(adhd ~ male + fampsych_substance_mside, data = expl, family = "binomial")

rfampsych_sucatt_mside <- glm(adhd ~ male + fampsych_sucatt_mside, data = expl, family = "binomial")

summary(rfampsych_dep_fside)

summary(rfampsych_anx_fside)

summary(rfampsych_substance_fside)

summary(rfampsych_sucatt_fside)

summary(rfampsych_dep_mside)

summary(rfampsych_anx_mside)#**

summary(rfampsych_substance_mside)

summary(rfampsych_sucatt_mside)

CrossTable(expl$adhd, expl$fampsych_anx_mside)

### Psychiatric problems in parents over time

ry1_mother_anx <- glm(adhd ~ male + y1_mother_anx, data = expl, family = "binomial")

ry1_mother_dep <- glm(adhd ~ male + y1_mother_dep, data = expl, family = "binomial")

ry1_father_anx <- glm(adhd ~ male + y1_father_anx, data = expl, family = "binomial")

ry1_father_dep <- glm(adhd ~ male + y1_father_dep, data = expl, family = "binomial")

ry3_father_substuse <- glm(adhd ~ male + y3_father_substuse, data = expl, family = "binomial")

ry3_mother_substuse <- glm(adhd ~ male + y3_mother_substuse, data = expl, family = "binomial")

ry3_father_anx <- glm(adhd ~ male + y3_father_anx, data = expl, family = "binomial")

ry3_father_dep <- glm(adhd ~ male + y3_father_dep, data = expl, family = "binomial")

ry3_mother_anx <- glm(adhd ~ male + y3_mother_anx, data = expl, family = "binomial")

ry3_mother_dep <- glm(adhd ~ male + y3_mother_dep, data = expl, family = "binomial")

ry5_mother_dep <- glm(adhd ~ male + y5_mother_dep, data = expl, family = "binomial")

ry5_father_dep <- glm(adhd ~ male + y5_father_dep, data = expl, family = "binomial")

summary(ry1_mother_anx)

summary(ry1_mother_dep)#***

summary(ry1_father_anx)

summary(ry1_father_dep)

summary(ry3_father_substuse)#**

summary(ry3_mother_substuse)

summary(ry3_father_anx)

summary(ry3_father_dep)

summary(ry3_mother_anx)

summary(ry3_mother_dep)#**

summary(ry5_mother_dep)#**

summary(ry5_father_dep)

CrossTable(expl$adhd, expl$y1_mother_dep)

CrossTable(expl$adhd, expl$y3_father_substuse)

CrossTable(expl$adhd, expl$y3_mother_dep)

CrossTable(expl$adhd, expl$y5_mother_dep)

### Physical health year 1

ry1_asthma <- glm(adhd ~ male + y1_asthma, data = expl, family = "binomial")

ry1_emergroom_accident <- glm(adhd ~ male + y1_emergroom_accident, data = expl, family = "binomial")

ry1_top10perchlthcvisits <- glm(adhd ~ male + y1_top10perchlthcvisits, data = expl, family = "binomial")

ry1_overnighthosp <- glm(adhd ~ male + y1_overnighthosp, data = expl, family = "binomial")

summary(ry1_asthma)

summary(ry1_emergroom_accident)

summary(ry1_top10perchlthcvisits)#***

summary(ry1_overnighthosp)#*

CrossTable(expl$adhd, expl$y1_top10perchlthcvisits)

### Physical health year 3

ry3_healthfairpoor <- glm(adhd ~ male + y3_healthfairpoor, data = expl, family = "binomial")

ry3_asthma <- glm(adhd ~ male + y3_asthma, data = expl, family = "binomial")

ry3_hlthcarevisitstop10perc <- glm(adhd ~ male + y3_hlthcarevisitstop10perc, data = expl, family = "binomial")

ry3_emergroom_accident <- glm(adhd ~ male + y3_emergroom_accident, data = expl, family = "binomial")

ry3_overnighthosp <- glm(adhd ~ male + y3_overnighthosp, data = expl, family = "binomial")

ry3_emergvisittop10perc2ormore <- glm(adhd ~ male + y3_emergvisittop10perc2ormore, data = expl, family = "binomial")

ry3_physdisability <- glm(adhd ~ male + y3_physdisability, data = expl, family = "binomial")

ry3_speechprblm <- glm(adhd ~ male + y3_speechprblm, data = df, family = "binomial")

ry5_speechproblem<-glm(adhd~male+y5_speechproblem, data=expl, family="binomial")

summary(ry3_healthfairpoor)#*

summary(ry3_asthma)#*

summary(ry3_hlthcarevisitstop10perc)#**

summary(ry3_emergroom_accident)

summary(ry3_overnighthosp)

summary(ry3_emergvisittop10perc2ormore)#*

summary(ry3_physdisability)

summary(ry3_speechprblm)

CrossTable(expl$adhd, expl$y3_hlthcarevisitstop10perc)

### Physical health year 5

ry5_hlthcarevisitstop10perc <- glm(adhd ~ male + y5_hlthcarevisitstop10perc, data = expl, family = "binomial")

ry5_ervisityes <- glm(adhd ~ male + y5_ervisityes, data = expl, family = "binomial")

ry5_overnightstayhospitalyes <- glm(adhd ~ male + y5_overnightstayhospitalyes, data = expl, family = "binomial")

ry5_er_accident_yes <- glm(adhd ~ male + y5_er_accident_yes, data = expl, family = "binomial")

ry5_over2earinf <- glm(adhd ~ male + y5_over2earinf, data = expl, family = "binomial")

ry5_speechproblem<-glm(adhd~male+y5_speechproblem, data=expl, family="binomial")

summary(ry5_hlthcarevisitstop10perc)#*

summary(ry5_ervisityes)

summary(ry5_overnightstayhospitalyes)

summary(ry5_er_accident_yes)#**

summary(ry5_over2earinf)

summary(ry5_speechproblem)#***

CrossTable(expl$adhd, expl$y5_er_accident_yes)

CrossTable(expl$adhd, expl$y5_speechproblem)

### Physical health year 9

ry9_phys_poorhealth <- glm(adhd ~ male + y9_phys_poorhealth, data = expl, family = "binomial")

ry9_phys_wheezingchest <- glm(adhd ~ male + y9_phys_wheezingchest, data = expl, family = "binomial")

ry9_phys_asthma <- glm(adhd ~ male + y9_phys_asthma, data = expl, family = "binomial")

ry9_phys_speechproblem <- glm(adhd ~ male + y9_phys_speechproblem, data = expl, family = "binomial")

ry9_phys_respallergy <- glm(adhd ~ male + y9_phys_respallergy, data = expl, family = "binomial")

ry9_phys_foodallergy <- glm(adhd ~ male + y9_phys_foodallergy, data = expl, family = "binomial")

ry9_phys_eczema <- glm(adhd ~ male + y9_phys_eczema, data = expl, family = "binomial")

ry9_phys_diarrhea <- glm(adhd ~ male + y9_phys_diarrhea, data = expl, family = "binomial")

ry9_phys_anemia <- glm(adhd ~ male + y9_phys_anemia, data = expl, family = "binomial")

ry9_phys_freqheadaches <- glm(adhd ~ male + y9_phys_freqheadaches, data = expl, family = "binomial")

ry9_phys_seizures <- glm(adhd ~ male + y9_phys_seizures, data = expl, family = "binomial")

ry9_phys_stuttering <- glm(adhd ~ male + y9_phys_stuttering, data = expl, family = "binomial")

ry9_phys_diabetes <- glm(adhd ~ male + y9_phys_diabetes, data = expl, family = "binomial")

ry9_over2earinf <- glm(adhd ~ male + y9_over2earinf, data = expl, family = "binomial")

ry9_parent_overeats <- glm(adhd ~ male + y9_parent_overeats, data = expl, family = "binomial")

y9_parent_overtired <- glm(adhd ~ male + y9_parent_overtired, data = expl, family = "binomial")

ry9_parent_overweight <- glm(adhd ~ male + y9_parent_overweight, data = expl, family = "binomial")

ry9_parent_unknownphys_pains <- glm(adhd ~ male + y9_parent_unknownphys_pains, data = expl, family = "binomial")

ry9_parent_unknownphys_headaches <- glm(adhd ~ male + y9_parent_unknownphys_headaches, data = expl, family = "binomial")

ry9_parent_unknownphys_nausea <- glm(adhd ~ male + y9_parent_unknownphys_nausea, data = expl, family = "binomial")

ry9_parent_unknownphys_eyes <- glm(adhd ~ male + y9_parent_unknownphys_eyes, data = expl, family = "binomial")

ry9_parent_unknownphys_skin <- glm(adhd ~ male + y9_parent_unknownphys_skin, data = expl, family = "binomial")

ry9_parent_unknownphys_vomit <- glm(adhd ~ male + y9_parent_unknownphys_vomit, data = expl, family = "binomial")

ry9_parent_unknownphys_other <- glm(adhd ~ male + y9_parent_unknownphys_other, data = expl, family = "binomial")

ry9_parent_unknownphys_cramps <- glm(adhd ~ male + y9_parent_unknownphys_cramps, data = expl, family = "binomial")

ry9_weight_percentile <- glm(adhd ~ male + y9_weight_percentile, data = expl, family = "binomial")

ry9_height_percentile <- glm(adhd ~ male + y9_height_percentile, data = expl, family = "binomial")

ry9_parent_constipated <-glm(adhd~male+y9_parent_constipated,data=expl,family="binomial")

summary(ry9_phys_poorhealth)#**

summary(ry9_phys_wheezingchest)#*

summary(ry9_phys_asthma)

summary(ry9_phys_speechproblem)#***

summary(ry9_phys_respallergy)#*

summary(ry9_phys_foodallergy)

summary(ry9_phys_eczema)

summary(ry9_phys_diarrhea)

summary(ry9_phys_anemia)

summary(ry9_phys_freqheadaches)

summary(ry9_phys_seizures)

summary(ry9_phys_stuttering)

summary(ry9_phys_diabetes)

summary(ry9_over2earinf)

summary(ry9_parent_overeats)

summary(y9_parent_overtired)

summary(ry9_parent_overweight)

summary(ry9_parent_unknownphys_pains)

summary(ry9_parent_unknownphys_headaches)

summary(ry9_parent_unknownphys_nausea)

summary(ry9_parent_unknownphys_eyes)

summary(ry9_parent_unknownphys_skin)#*

summary(ry9_parent_unknownphys_vomit)

summary(ry9_parent_unknownphys_other)

summary(ry9_parent_unknownphys_cramps)

summary(ry9_weight_percentile)#**

summary(ry9_height_percentile)

summary(ry9_parent_constipated)

CrossTable(expl$adhd, expl$y9_phys_poorhealth)

CrossTable(expl$adhd, expl$y9_phys_speechproblem)

aggregate(y9_weight_percentile ~ adhd, expl, mean)

aggregate(y9_weight_percentile ~ adhd, expl, sd)

### Year 1 Child Factors

ry1_shy <- glm(adhd ~ male + y1_shy, data = expl, family = "binomial")

ry1_fusscry <- glm(adhd ~ male + y1_fusscry, data = expl, family = "binomial")

ry1_social <- glm(adhd ~ male + y1_social, data = expl, family = "binomial")

ry1_upseteasily <- glm(adhd ~ male + y1_upseteasily, data = expl, family = "binomial")

ry1_strongreact <- glm(adhd ~ male + y1_strongreact, data = expl, family = "binomial")

ry1_shystrangers <- glm(adhd ~ male + y1_shystrangers, data = expl, family = "binomial")

summary(ry1_shy)

summary(ry1_fusscry)

summary(ry1_social)

summary(ry1_upseteasily)

summary(ry1_strongreact)

summary(ry1_shystrangers)

### Year 3 Factors

ry3_attachment_securevsnot <- glm(adhd ~ male + y3_attachment_securevsnot, data = expl, family = "binomial")

ry3_symphatothers <- glm(adhd ~ male + y3_symphatothers, data = expl, family = "binomial")

ry3_understothersfeelings <- glm(adhd ~ male + y3_understothersfeelings, data = expl, family = "binomial")

ry3_openneeds <- glm(adhd ~ male + y3_openneeds, data = expl, family = "binomial")

ry3_joinotherchildren <- glm(adhd ~ male + y3_joinotherchildren, data = expl, family = "binomial")

ry3_playswithothers <- glm(adhd ~ male + y3_playswithothers, data = expl, family = "binomial")

ry3_interestinthings <- glm(adhd ~ male + y3_interestinthings, data = expl, family = "binomial")

ry3_confidentothers <- glm(adhd ~ male + y3_confidentothers, data = expl, family = "binomial")

ry3_proud <- glm(adhd ~ male + y3_proud, data = expl, family = "binomial")

ry3_manyinterests <- glm(adhd ~ male + y3_manyinterests, data = expl, family = "binomial")

summary(ry3_attachment_securevsnot)#***

summary(ry3_symphatothers)

summary(ry3_understothersfeelings)

summary(ry3_openneeds)#*

summary(ry3_joinotherchildren)

summary(ry3_playswithothers)

summary(ry3_interestinthings)

summary(ry3_confidentothers)

summary(ry3_proud)

summary(ry3_manyinterests)

CrossTable(expl$adhd, expl$y3_attachment_securevsnot)

ry3_enjpoystalkparent <- glm(adhd ~ male + y3_enjpoystalkparent, data = expl, family = "binomial")

ry3_tooyoung <- glm(adhd ~ male + y3_tooyoung, data = expl, family = "binomial")

ry3_pooreyecont <- glm(adhd ~ male + y3_pooreyecont, data = expl, family = "binomial")

ry3_cantconcentr <- glm(adhd ~ male + y3_cantconcentr, data = expl, family = "binomial")

ry3_cantsitstill <- glm(adhd ~ male + y3_cantsitstill, data = expl, family = "binomial")

ry3_cantwait <- glm(adhd ~ male + y3_cantwait, data = expl, family = "binomial")

ry3_clingadults <- glm(adhd ~ male + y3_clingadults, data = expl, family = "binomial")

ry3_criesalot <- glm(adhd ~ male + y3_criesalot, data = expl, family = "binomial")

ry3_cruelanimals <- glm(adhd ~ male + y3_cruelanimals, data = expl, family = "binomial")

ry3_defiant <- glm(adhd ~ male + y3_defiant, data = expl, family = "binomial")

summary(ry3_enjpoystalkparent)#*

summary(ry3_tooyoung)

summary(ry3_pooreyecont)#**

summary(ry3_cantconcentr)#***

summary(ry3_cantsitstill)#***

summary(ry3_cantwait)#***

summary(ry3_clingadults)#**

summary(ry3_criesalot)#***

summary(ry3_cruelanimals)

summary(ry3_defiant)#***

CrossTable(expl$adhd, expl$y3_pooreyecont)

CrossTable(expl$adhd, expl$y3_cantconcentr)

CrossTable(expl$adhd, expl$y3_cantsitstill)

CrossTable(expl$adhd, expl$y3_cantwait)

CrossTable(expl$adhd, expl$y3_clingadults)

CrossTable(expl$adhd, expl$y3_criesalot)

CrossTable(expl$adhd, expl$y3_defiant)

ry3_demandsmetdirect <- glm(adhd ~ male + y3_demandsmetdirect, data = expl, family = "binomial")

ry3_destroyownthngs <- glm(adhd ~ male + y3_destroyownthngs, data = expl, family = "binomial")

ry3_destroyothersthngs <- glm(adhd ~ male + y3_destroyothersthngs, data = expl, family = "binomial")

ry3_disobed <- glm(adhd ~ male + y3_disobed, data = expl, family = "binomial")

ry3_distrbdchroutine <- glm(adhd ~ male + y3_distrbdchroutine, data = expl, family = "binomial")

ry3_dontrespondtalk <- glm(adhd ~ male + y3_dontrespondtalk, data = expl, family = "binomial")

ry3_notgetalongchildren <- glm(adhd ~ male + y3_notgetalongchildren, data = expl, family = "binomial")

ry3_actslikeadult <- glm(adhd ~ male + y3_actslikeadult, data = expl, family = "binomial")

ry3_noguilt <- glm(adhd ~ male + y3_noguilt, data = expl, family = "binomial")

ry3_easilyfrustr <- glm(adhd ~ male + y3_easilyfrustr, data = expl, family = "binomial")

summary(ry3_demandsmetdirect)#***

summary(ry3_destroyownthngs)#***

summary(ry3_destroyothersthngs)#***

summary(ry3_disobed)#***

summary(ry3_distrbdchroutine)#***

summary(ry3_dontrespondtalk)

summary(ry3_notgetalongchildren)

summary(ry3_actslikeadult)

summary(ry3_noguilt)#***

summary(ry3_easilyfrustr)#***

CrossTable(expl$adhd, expl$y3_demandsmetdirect)

CrossTable(expl$adhd, expl$y3_destroyownthngs)

CrossTable(expl$adhd, expl$y3_destroyothersthngs)

CrossTable(expl$adhd, expl$y3_disobed)

CrossTable(expl$adhd, expl$y3_distrbdchroutine)

CrossTable(expl$adhd, expl$y3_noguilt)

CrossTable(expl$adhd, expl$y3_easilyfrustr)

ry3_flngseasyilyhurt <- glm(adhd ~ male + y3_flngseasyilyhurt, data = expl, family = "binomial")

ry3_jealous <- glm(adhd ~ male + y3_jealous, data = expl, family = "binomial")

ry3_accidentprone <- glm(adhd ~ male + y3_accidentprone, data = expl, family = "binomial")

ry3_fights <- glm(adhd ~ male + y3_fights, data = expl, family = "binomial")

ry3_sleepproblems <- glm(adhd ~ male + y3_sleepproblems, data = expl, family = "binomial")

ry3_upsetseparation <- glm(adhd ~ male + y3_upsetseparation, data = expl, family = "binomial")

ry3_hitsothers <- glm(adhd ~ male + y3_hitsothers, data = expl, family = "binomial")

ry3_hurtothersunintent <- glm(adhd ~ male + y3_hurtothersunintent, data = expl, family = "binomial")

ry3_unhappynoreason <- glm(adhd ~ male + y3_unhappynoreason, data = expl, family = "binomial")

ry3_angrymoods <- glm(adhd ~ male + y3_angrymoods, data = expl, family = "binomial")

summary(ry3_flngseasyilyhurt)

summary(ry3_jealous)

summary(ry3_accidentprone)#*

summary(ry3_fights)#**

summary(ry3_sleepproblems)#**

summary(ry3_upsetseparation)

summary(ry3_hitsothers)#***

summary(ry3_hurtothersunintent)#*

summary(ry3_unhappynoreason)#*

summary(ry3_angrymoods)

CrossTable(expl$adhd, expl$y3_fights)

CrossTable(expl$adhd, expl$y3_sleepproblems)

CrossTable(expl$adhd, expl$y3_hitsothers)

ry3_nervous <- glm(adhd ~ male + y3_nervous, data = expl, family = "binomial")

ry3_overtired <- glm(adhd ~ male + y3_overtired, data = expl, family = "binomial")

ry3_physicallyattacks <- glm(adhd ~ male + y3_physicallyattacks, data = expl, family = "binomial")

ry3_notsenstowpunish <- glm(adhd ~ male + y3_notsenstowpunish, data = expl, family = "binomial")

ry3_shiftactivity <- glm(adhd ~ male + y3_shiftactivity, data = expl, family = "binomial")

ry3_refuseplaygames <- glm(adhd ~ male + y3_refuseplaygames, data = expl, family = "binomial")

ry3_screams <- glm(adhd ~ male + y3_screams, data = expl, family = "binomial")

ry3_unresponsaffection <- glm(adhd ~ male + y3_unresponsaffection, data = expl, family = "binomial")

ry3_easyembarassed <- glm(adhd ~ male + y3_easyembarassed, data = expl, family = "binomial")

ry3_selfish <- glm(adhd ~ male + y3_selfish, data = expl, family = "binomial")

summary(ry3_nervous)#***

summary(ry3_overtired)#**

summary(ry3_physicallyattacks)#**

summary(ry3_notsenstowpunish)#***

summary(ry3_shiftactivity)#*

summary(ry3_refuseplaygames)

summary(ry3_screams)#***

summary(ry3_unresponsaffection)

summary(ry3_easyembarassed)

summary(ry3_selfish)#*

CrossTable(expl$adhd, expl$y3_nervous)

CrossTable(expl$adhd, expl$y3_overtired)

CrossTable(expl$adhd, expl$y3_physicallyattacks)

CrossTable(expl$adhd, expl$y3_notsenstowpunish)

CrossTable(expl$adhd, expl$y3_screams)

ry3_littleaffctothers <- glm(adhd ~ male + y3_littleaffctothers, data = expl, family = "binomial")

ry3_lttleinterestthings <- glm(adhd ~ male + y3_lttleinterestthings, data = expl, family = "binomial")

ry3_shy <- glm(adhd ~ male + y3_shy, data = expl, family = "binomial")

ry3_stubborn <- glm(adhd ~ male + y3_stubborn, data = expl, family = "binomial")

ry3_moodswings <- glm(adhd ~ male + y3_moodswings, data = expl, family = "binomial")

ry3_sulks <- glm(adhd ~ male + y3_sulks, data = expl, family = "binomial")

ry3_tempertantrums <- glm(adhd ~ male + y3_tempertantrums, data = expl, family = "binomial")

ry3_tooanxious <- glm(adhd ~ male + y3_tooanxious, data = expl, family = "binomial")

ry3_uncooperative <- glm(adhd ~ male + y3_uncooperative, data = expl, family = "binomial")

ry3_underactive <- glm(adhd ~ male + y3_underactive, data = expl, family = "binomial")

ry3_sad <- glm(adhd ~ male + y3_sad, data = expl, family = "binomial")

ry3_loud <- glm(adhd ~ male + y3_loud, data = expl, family = "binomial")

ry3_attentionseeking <- glm(adhd ~ male + y3_attentionseeking, data = expl, family = "binomial")

ry3_whiny <- glm(adhd ~ male + y3_whiny, data = expl, family = "binomial")

ry3_withdrawn <- glm(adhd ~ male + y3_withdrawn, data = expl, family = "binomial")

summary(ry3_littleaffctothers)

summary(ry3_lttleinterestthings)

summary(ry3_shy)

summary(ry3_stubborn)#**

summary(ry3_moodswings)#***

summary(ry3_sulks)#*

summary(ry3_tempertantrums)#***

summary(ry3_tooanxious)#*

summary(ry3_uncooperative)#*

summary(ry3_underactive)

summary(ry3_sad)

summary(ry3_loud)

summary(ry3_attentionseeking)#**

summary(ry3_whiny)#**

summary(ry3_withdrawn)

CrossTable(expl$adhd, expl$y3_speechprblm)

CrossTable(expl$adhd, expl$y3_stubborn)

CrossTable(expl$adhd, expl$y3_moodswings)

CrossTable(expl$adhd, expl$y3_tempertantrums)

CrossTable(expl$adhd, expl$y3_attentionseeking)

CrossTable(expl$adhd, expl$y3_whiny)

### ### ### ### ### ### ### ###

### Explore which predictors of early/physical are most important + year 3 factors

### ### ### ### ### ### ### ###

## Early factors and family history of psych disorders

# Create evenly distributed synthetic data

data.rose <- ROSE(adhd ~ pregnancy_smoke+baseline_fatherinjail+

fampsych_anx_mside+y1_mother_dep+y3_father_substuse+y3_mother_dep+y5_mother_dep+

y1_top10perchlthcvisits+y3_hlthcarevisitstop10perc+y5_speechproblem+y5_er_accident_yes+y3_speechprblm+

y9_phys_poorhealth+y9_phys_speechproblem+y9_weight_percentile,

data = expl, seed = 1)$data

# Run bestglm to find best subset model. Use syntehetic data to get better coefficients to find cases

lowbwt.bglm <- data.rose[, c("pregnancy_smoke","baseline_fatherinjail","fampsych_anx_mside",

"y1_mother_dep","y3_father_substuse","y3_mother_dep","y5_mother_dep","y1_top10perchlthcvisits",

"y3_hlthcarevisitstop10perc","y5_speechproblem","y5_er_accident_yes","y3_speechprblm",

"y9_phys_poorhealth","y9_phys_speechproblem","y9_weight_percentile","adhd")]

names(lowbwt.bglm)[names(lowbwt.bglm) == "adhd"] <- "y"

lowbwt.bglm <- as.data.frame(lowbwt.bglm)

best.logit <- bestglm(lowbwt.bglm,

IC = "AIC",

family=binomial,

method = "exhaustive")

summary(best.logit$BestModel)

#baseline_fatherinjail 0.917151 0.348815 2.629 0.008555 **

#fampsych_anx_mside 0.278351 0.144714 1.923 0.054422 .

#y5_mother_dep 0.702992 0.187351 3.752 0.000175 ***

#y1_top10perchlthcvisits 0.353092 0.176906 1.996 0.045941 *

#y5_speechproblem 0.746925 0.255906 2.919 0.003514 **

#y5_er_accident_yes 0.591551 0.189004 3.130 0.001749 **

#y9_phys_poorhealth -0.626496 0.387160 -1.618 0.105623

#y9_phys_speechproblem 1.127937 0.213616 5.280 1.29e-07 ***

#y9_weight_percentile -0.005095 0.002092 -2.435 0.014889 *

### Year 3 factors (Exclude attachment because few have this data)

# Run bestglm to find best subset model. Use syntehetic data to get better coefficients to find cases. Can only use 15 predictors at a time.

# First set of 9

data.rose2 <- ROSE(adhd ~ y3_pooreyecont+y3_cantconcentr+y3_cantsitstill+y3_cantwait+y3_clingadults+y3_criesalot+

y3_defiant+y3_demandsmetdirect+y3_destroyownthngs,

data = expl, seed = 1)$data

lowbwt.bglm <- data.rose2[, c("y3_pooreyecont","y3_cantconcentr","y3_cantsitstill","y3_cantwait","y3_clingadults","y3_criesalot",

"y3_defiant","y3_demandsmetdirect","y3_destroyownthngs",

"adhd")]

names(lowbwt.bglm)[names(lowbwt.bglm) == "adhd"] <- "y"

lowbwt.bglm <- as.data.frame(lowbwt.bglm)

best.logit <- bestglm(lowbwt.bglm,

IC = "AIC",

family=binomial,

method = "exhaustive")

summary(best.logit$BestModel)

#y3_cantconcentr 0.5077 0.1459 3.48 0.00050 ***

#y3_cantsitstill 0.3861 0.1011 3.82 0.00013 ***

#y3_clingadults 0.3451 0.1191 2.90 0.00375 **

#y3_defiant 0.4216 0.1244 3.39 0.00070 ***

#y3_demandsmetdirect 0.2040 0.1056 1.93 0.05330 .

# Next set of 9

data.rose2 <- ROSE(adhd ~ y3_notgetalongchildren+y3_noguilt+y3_fights+

y3_sleepproblems+y3_hitsothers+y3_nervous+

y3_disobed+y3_distrbdchroutine+y3_destroyothersthngs,data = expl, seed = 1)$data

lowbwt.bglm <- data.rose2[, c("y3_notgetalongchildren","y3_noguilt","y3_fights",

"y3_sleepproblems","y3_hitsothers",

"y3_nervous","y3_disobed",

"y3_distrbdchroutine","y3_destroyothersthngs","adhd")]

names(lowbwt.bglm)[names(lowbwt.bglm) == "adhd"] <- "y"

lowbwt.bglm <- as.data.frame(lowbwt.bglm)

best.logit <- bestglm(lowbwt.bglm,

IC = "AIC",

family=binomial,

method = "exhaustive")

summary(best.logit$BestModel)

#y3_noguilt 0.3336 0.1230 2.71 0.00668 **

#y3_fights 0.3743 0.2105 1.78 0.07528 .

#y3_sleepproblems 0.2922 0.1477 1.98 0.04782 *

#y3_hitsothers 0.3161 0.1510 2.09 0.03634 *

#y3_disobed 0.9262 0.1726 5.37 0.0000000805 ***

#y3_distrbdchroutine 0.6650 0.1749 3.80 0.00014 ***

# Last set of 9

data.rose2 <- ROSE(adhd ~ y3_overtired+y3_physicallyattacks+y3_notsenstowpunish+y3_screams+

y3_stubborn+y3_moodswings+y3_tempertantrums+y3_attentionseeking+

y3_whiny,data = expl, seed = 1)$data

lowbwt.bglm <- data.rose2[, c("y3_overtired","y3_physicallyattacks","y3_notsenstowpunish","y3_screams",

"y3_stubborn","y3_moodswings","y3_tempertantrums","y3_attentionseeking",

"y3_whiny","adhd")]

names(lowbwt.bglm)[names(lowbwt.bglm) == "adhd"] <- "y"

lowbwt.bglm <- as.data.frame(lowbwt.bglm)

best.logit <- bestglm(lowbwt.bglm,

IC = "AIC",

family=binomial,

method = "exhaustive")

summary(best.logit$BestModel)

#y3_overtired -0.4181 0.2726 -1.53 0.125

#y3_physicallyattacks 0.5631 0.2796 2.01 0.044 *

#y3_notsenstowpunish 0.3062 0.1285 2.38 0.017 *

#y3_moodswings 0.3048 0.1466 2.08 0.038 *

#y3_tempertantrums 0.7308 0.1182 6.18 0.00000000063 ***

#y3_whiny -0.1966 0.1215 -1.62 0.106

### Combined model

data.rose2 <- ROSE(adhd ~ y3_cantconcentr+y3_cantsitstill+y3_clingadults+

y3_defiant+y3_noguilt+y3_disobed+y3_distrbdchroutine+y3_tempertantrums,

data = expl, seed = 1)$data

lowbwt.bglm <- data.rose2[, c("y3_cantconcentr","y3_cantsitstill","y3_clingadults","y3_defiant",

"y3_noguilt","y3_disobed","y3_distrbdchroutine","y3_tempertantrums","adhd")]

names(lowbwt.bglm)[names(lowbwt.bglm) == "adhd"] <- "y"

lowbwt.bglm <- as.data.frame(lowbwt.bglm)

best.logit <- bestglm(lowbwt.bglm,

IC = "AIC",

family=binomial,

method = "exhaustive")

summary(best.logit$BestModel)

#y3_cantconcentr 0.4151 0.1543 2.69 0.00713 **

#y3_cantsitstill 0.3893 0.1036 3.76 0.00017 ***

#y3_clingadults 0.2899 0.1211 2.39 0.01668 *

#y3_disobed 0.2405 0.1689 1.42 0.15454

#y3_distrbdchroutine 0.6887 0.1672 4.12 0.0000382089957 ***

#y3_tempertantrums 0.4750 0.1204 3.94 0.0000802545844 ***

# Examine accuracy in non-imputed holdout sample

glm_probs <- data.frame(probs = predict(best.logit$BestModel, newdata = holdout,type="response"))

glm_pred <- glm_probs %>% mutate(pred = ifelse(probs>.5, "1", "0"))

glm_pred <- cbind(holdout, glm_pred)

glm_pred %>% count(pred, adhd) %>% spread(adhd, n, fill = 0)

# pred 0 1

#1 0 321 61

#2 1 85 23

#3 <NA> 193 32

#Sensitivity

23/(23+61)*100 # 27.4%

#PPV

23/(85+23)*100 # 21.3%

### ### ### ### ### ### ### ###

### Year 5 factors

### ### ### ### ### ### ### ###

ry5_under7hoursofsleep <-glm(adhd~male+y5_under7hoursofsleep, data=expl, family="binomial")

ry5_sleepinparentsbed<-glm(adhd~male+y5_sleepinparentsbed, data=expl, family="binomial")

ry5_argues<-glm(adhd~male+y5_argues, data=expl, family="binomial")

ry5_brags<-glm(adhd~male+y5_brags, data=expl, family="binomial")

ry5_undstotherfeelings<-glm(adhd~male+y5_undstotherfeelings, data=expl, family="binomial")

ry5_overeats<-glm(adhd~male+y5_overeats, data=expl, family="binomial")

ry5_loneliness<-glm(adhd~male+y5_loneliness, data=expl, family="binomial")

ry5_confused<-glm(adhd~male+y5_confused, data=expl, family="binomial")

ry5_cruelothers<-glm(adhd~male+y5_cruelothers, data=expl, family="binomial")

ry5_daydreams<-glm(adhd~male+y5_daydreams, data=expl, family="binomial")

ry5_destroyownthings<-glm(adhd~male+y5_destroyownthings, data=expl, family="binomial")

ry5_destroyotherthings<-glm(adhd~male+y5_destroyotherthings, data=expl, family="binomial")

ry5_sympathyothers<-glm(adhd~male+y5_sympathyothers, data=expl, family="binomial")

ry5_disobedienthome<-glm(adhd~male+y5_disobedienthome, data=expl, family="binomial")

ry5_disobedientschool<-glm(adhd~male+y5_disobedientschool, data=expl, family="binomial")

ry5_accidentprone<-glm(adhd~male+y5_accidentprone, data=expl, family="binomial")

ry5_openwhatwant<-glm(adhd~male+y5_openwhatwant, data=expl, family="binomial")

ry5_jealous<-glm(adhd~male+y5_jealous, data=expl, family="binomial")

ry5_dothinkbadobsession<-glm(adhd~male+y5_dothinkbadobsession, data=expl, family="binomial")

ry5_hastobeperfect<-glm(adhd~male+y5_hastobeperfect, data=expl, family="binomial")

ry5_complainsnoloveshim<-glm(adhd~male+y5_complainsnoloveshim, data=expl, family="binomial")

ry5_otheroutotget<-glm(adhd~male+y5_otheroutotget, data=expl, family="binomial")

ry5_getintofights<-glm(adhd~male+y5_getintofights, data=expl, family="binomial")

ry5_getteased<-glm(adhd~male+y5_getteased, data=expl, family="binomial")

ry5_hangswotherstrouble<-glm(adhd~male+y5_hangswotherstrouble, data=expl, family="binomial")

ry5_impulsive<-glm(adhd~male+y5_impulsive, data=expl, family="binomial")

ry5_ratherbealone<-glm(adhd~male+y5_ratherbealone, data=expl, family="binomial")

ry5_liescheats<-glm(adhd~male+y5_liescheats, data=expl, family="binomial")

ry5_nervoustwitches<-glm(adhd~male+y5_nervoustwitches, data=expl, family="binomial")

ry5_notlikedbyothers<-glm(adhd~male+y5_notlikedbyothers, data=expl, family="binomial")

ry5_feelstooguilty<-glm(adhd~male+y5_feelstooguilty, data=expl, family="binomial")

ry5_joinsothersplay<-glm(adhd~male+y5_joinsothersplay, data=expl, family="binomial")

ry5_overweight<-glm(adhd~male+y5_overweight, data=expl, family="binomial")

ry5_justwatchothers<-glm(adhd~male+y5_justwatchothers, data=expl, family="binomial")

ry5_attackspeople<-glm(adhd~male+y5_attackspeople, data=expl, family="binomial")

ry5_poorschoolwork<-glm(adhd~male+y5_poorschoolwork, data=expl, family="binomial")

ry5_clumsy<-glm(adhd~male+y5_clumsy, data=expl, family="binomial")

ry5_preferolderkids<-glm(adhd~male+y5_preferolderkids, data=expl, family="binomial")

ry5_preferyoungerkids<-glm(adhd~male+y5_preferyoungerkids, data=expl, family="binomial")

ry5_refusestotalk<-glm(adhd~male+y5_refusestotalk, data=expl, family="binomial")

ry5_runsawayfromhome<-glm(adhd~male+y5_runsawayfromhome, data=expl, family="binomial")

ry5_screamsalot<-glm(adhd~male+y5_screamsalot, data=expl, family="binomial")

ry5_attentionfromothers<-glm(adhd~male+y5_attentionfromothers, data=expl, family="binomial")

ry5_secretive<-glm(adhd~male+y5_secretive, data=expl, family="binomial")

ry5_easilyembarassed<-glm(adhd~male+y5_easilyembarassed, data=expl, family="binomial")

ry5_setsfires<-glm(adhd~male+y5_setsfires, data=expl, family="binomial")

ry5_clownsaround<-glm(adhd~male+y5_clownsaround, data=expl, family="binomial")

ry5_shy<-glm(adhd~male+y5_shy, data=expl, family="binomial")

ry5_staresblankly<-glm(adhd~male+y5_staresblankly, data=expl, family="binomial")

ry5_saysthanksplease<-glm(adhd~male+y5_saysthanksplease, data=expl, family="binomial")

ry5_stealshome<-glm(adhd~male+y5_stealshome, data=expl, family="binomial")

ry5_stealsoutsidehome<-glm(adhd~male+y5_stealsoutsidehome, data=expl, family="binomial")

ry5_wanttoplayothers<-glm(adhd~male+y5_wanttoplayothers, data=expl, family="binomial")

ry5_sulks<-glm(adhd~male+y5_sulks, data=expl, family="binomial")

ry5_suspicious<-glm(adhd~male+y5_suspicious, data=expl, family="binomial")

ry5_swears<-glm(adhd~male+y5_swears, data=expl, family="binomial")

ry5_playstalkothers<-glm(adhd~male+y5_playstalkothers, data=expl, family="binomial")

ry5_talkstoomuch<-glm(adhd~male+y5_talkstoomuch, data=expl, family="binomial")

ry5_teases<-glm(adhd~male+y5_teases, data=expl, family="binomial")

ry5_confidentwithothers<-glm(adhd~male+y5_confidentwithothers, data=expl, family="binomial")

ry5_treatenspeople<-glm(adhd~male+y5_treatenspeople, data=expl, family="binomial")

ry5_proud<-glm(adhd~male+y5_proud, data=expl, family="binomial")

ry5_underactive<-glm(adhd~male+y5_underactive, data=expl, family="binomial")

ry5_unusuallyloud<-glm(adhd~male+y5_unusuallyloud, data=expl, family="binomial")

ry5_interestdiffthings<-glm(adhd~male+y5_interestdiffthings, data=expl, family="binomial")

ry5_vandalizes<-glm(adhd~male+y5_vandalizes, data=expl, family="binomial")

ry5_worries<-glm(adhd~male+y5_worries, data=expl, family="binomial")

ry5_enjoystalktoyou<-glm(adhd~male+y5_enjoystalktoyou, data=expl, family="binomial")

ry5_cantconcentrate<-glm(adhd~male+y5_cantconcentrate, data=expl, family="binomial")

ry5_cantsitstill<-glm(adhd~male+y5_cantsitstill, data=expl, family="binomial")

ry5_clingsadults<-glm(adhd~male+y5_clingsadults, data=expl, family="binomial")

ry5_criesalot<-glm(adhd~male+y5_criesalot, data=expl, family="binomial")

ry5_disobedient<-glm(adhd~male+y5_disobedient, data=expl, family="binomial")

ry5_dontgetalongotherchldrn<-glm(adhd~male+y5_dontgetalongotherchldrn, data=expl, family="binomial")

ry5_noguilt<-glm(adhd~male+y5_noguilt, data=expl, family="binomial")

ry5_troublefallingasleep<-glm(adhd~male+y5_troublefallingasleep, data=expl, family="binomial")

ry5_nervous<-glm(adhd~male+y5_nervous, data=expl, family="binomial")

ry5_stubborn<-glm(adhd~male+y5_stubborn, data=expl, family="binomial")

ry5_suddenmoodswings<-glm(adhd~male+y5_suddenmoodswings, data=expl, family="binomial")

ry5_tempertantrums<-glm(adhd~male+y5_tempertantrums, data=expl, family="binomial")

ry5_anxious<-glm(adhd~male+y5_anxious, data=expl, family="binomial")

ry5_sad<-glm(adhd~male+y5_sad, data=expl, family="binomial")

ry5_wantsalotattention<-glm(adhd~male+y5_wantsalotattention, data=expl, family="binomial")

ry5_withdrawnotherchildren<-glm(adhd~male+y5_withdrawnotherchildren, data=expl, family="binomial")

ry5_feelworthless<-glm(adhd~male+y5_feelworthless, data=expl, family="binomial")

ry5_actstooyoungforage<-glm(adhd~male+y5_actstooyoungforage, data=expl, family="binomial")

summary(ry5_under7hoursofsleep)

summary(ry5_sleepinparentsbed)

summary(ry5_argues)#***

summary(ry5_brags)

summary(ry5_undstotherfeelings)#*

summary(ry5_overeats)

summary(ry5_loneliness)

summary(ry5_confused)#**

summary(ry5_cruelothers)#**

summary(ry5_daydreams)#**

summary(ry5_destroyownthings)#**

summary(ry5_destroyotherthings)#*

summary(ry5_sympathyothers)

summary(ry5_disobedienthome)#***

summary(ry5_disobedientschool)#***

summary(ry5_accidentprone)#**

summary(ry5_openwhatwant)

summary(ry5_jealous)

summary(ry5_dothinkbadobsession)

summary(ry5_hastobeperfect)#*

summary(ry5_complainsnoloveshim)

summary(ry5_otheroutotget)

summary(ry5_getintofights)#**

summary(ry5_getteased)

summary(ry5_hangswotherstrouble)#*

summary(ry5_impulsive)#***

summary(ry5_ratherbealone)

summary(ry5_liescheats)#*

summary(ry5_nervoustwitches)

summary(ry5_notlikedbyothers)

summary(ry5_feelstooguilty)

summary(ry5_joinsothersplay)

summary(ry5_overweight)

summary(ry5_justwatchothers)

summary(ry5_attackspeople)#*

summary(ry5_poorschoolwork)

summary(ry5_clumsy)#*

summary(ry5_preferolderkids)#***

summary(ry5_preferyoungerkids)

summary(ry5_refusestotalk)

summary(ry5_runsawayfromhome)#*

summary(ry5_screamsalot)#***

summary(ry5_attentionfromothers)

summary(ry5_secretive)

summary(ry5_easilyembarassed)

summary(ry5_setsfires)#**

summary(ry5_clownsaround)#***

summary(ry5_shy)#*

summary(ry5_staresblankly)

summary(ry5_saysthanksplease)

summary(ry5_stealshome)

summary(ry5_stealsoutsidehome)

summary(ry5_wanttoplayothers)

summary(ry5_sulks)#*

summary(ry5_suspicious)

summary(ry5_swears)

summary(ry5_playstalkothers)#*

summary(ry5_talkstoomuch)#***

summary(ry5_teases)

summary(ry5_confidentwithothers)

summary(ry5_treatenspeople)#*

summary(ry5_proud)#*

summary(ry5_underactive)

summary(ry5_unusuallyloud)#***

summary(ry5_interestdiffthings)

summary(ry5_vandalizes)

summary(ry5_worries)

summary(ry5_enjoystalktoyou)#*

summary(ry5_cantconcentrate)#***

summary(ry5_cantsitstill)#***

summary(ry5_clingsadults)#**

summary(ry5_criesalot)#*

summary(ry5_disobedient)#***

summary(ry5_dontgetalongotherchldrn)#***

summary(ry5_noguilt)#***

summary(ry5_troublefallingasleep)#***

summary(ry5_nervous)#***

summary(ry5_speechproblem)#***

summary(ry5_stubborn)#***

summary(ry5_suddenmoodswings)#***

summary(ry5_tempertantrums)#***

summary(ry5_anxious)#**

summary(ry5_sad)

summary(ry5_wantsalotattention)#***

summary(ry5_withdrawnotherchildren)

summary(ry5_feelworthless)

summary(ry5_actstooyoungforage)#***

CrossTable(expl$adhd, expl$y5_argues)

CrossTable(expl$adhd, expl$y5_confused)

CrossTable(expl$adhd, expl$y5_cruelothers)

CrossTable(expl$adhd, expl$y5_daydreams)

CrossTable(expl$adhd, expl$y5_destroyownthings)

CrossTable(expl$adhd, expl$y5_disobedienthome)

CrossTable(expl$adhd, expl$y5_disobedientschool)

CrossTable(expl$adhd, expl$y5_accidentprone)

CrossTable(expl$adhd, expl$y5_getintofights)

CrossTable(expl$adhd, expl$y5_impulsive)

CrossTable(expl$adhd, expl$y5_preferolderkids)

CrossTable(expl$adhd, expl$y5_screamsalot)

CrossTable(expl$adhd, expl$y5_setsfires)

CrossTable(expl$adhd, expl$y5_clownsaround)

CrossTable(expl$adhd, expl$y5_talkstoomuch)

CrossTable(expl$adhd, expl$y5_unusuallyloud)

CrossTable(expl$adhd, expl$y5_cantconcentrate)

CrossTable(expl$adhd, expl$y5_cantsitstill)

CrossTable(expl$adhd, expl$y5_clingsadults)

CrossTable(expl$adhd, expl$y5_disobedient)

CrossTable(expl$adhd, expl$y5_dontgetalongotherchldrn)

CrossTable(expl$adhd, expl$y5_noguilt)

CrossTable(expl$adhd, expl$y5_troublefallingasleep)

CrossTable(expl$adhd, expl$y5_nervous)

CrossTable(expl$adhd, expl$y5_stubborn)

CrossTable(expl$adhd, expl$y5_suddenmoodswings)

CrossTable(expl$adhd, expl$y5_tempertantrums)

CrossTable(expl$adhd, expl$y5_anxious)

CrossTable(expl$adhd, expl$y5_wantsalotattention)

CrossTable(expl$adhd, expl$y5_actstooyoungforage)

ry5_mdoc_lowpositiveaffect<-glm(adhd~male+y5_mdoc_lowpositiveaffect, data=expl, family="binomial")

ry5_mdoc_highnegativeaffect<-glm(adhd~male+y5_mdoc_highnegativeaffect, data=expl, family="binomial")

ry5_mdoc_detached<-glm(adhd~male+y5_mdoc_detached, data=expl, family="binomial")

ry5_mdoc_notverbal<-glm(adhd~male+y5_mdoc_notverbal, data=expl, family="binomial")

summary(ry5_mdoc_lowpositiveaffect) #*

summary(ry5_mdoc_highnegativeaffect) #**

summary(ry5_mdoc_detached)

summary(ry5_mdoc_notverbal)

CrossTable(expl$adhd, expl$y5_mdoc_highnegativeaffect)

### Examine accuracy

# First 11 variables

data.rose <- ROSE(adhd ~ y5_argues+y5_confused+y5_cruelothers+y5_daydreams+y5_destroyownthings+y5_disobedienthome+y5_disobedientschool+

y5_accidentprone+y5_getintofights+y5_impulsive+y5_preferolderkids,

data = expl, seed = 1)$data

lowbwt.bglm <- data.rose[, c("y5_argues","y5_confused","y5_cruelothers","y5_daydreams","y5_destroyownthings","y5_disobedienthome","y5_disobedientschool",

"y5_accidentprone","y5_getintofights","y5_impulsive","y5_preferolderkids","adhd")]

names(lowbwt.bglm)[names(lowbwt.bglm) == "adhd"] <- "y"

lowbwt.bglm <- as.data.frame(lowbwt.bglm)

best.logit <- bestglm(lowbwt.bglm,

IC = "AIC",

family=binomial,

method = "exhaustive")

summary(best.logit$BestModel)

#y5_argues 0.19984 0.09526 2.098 0.035925 *

#y5_confused 0.98273 0.40368 2.434 0.014916 *

#y5_destroyownthings 0.46351 0.15204 3.049 0.002300 **

#y5_disobedientschool 0.69578 0.20409 3.409 0.000652 ***

#y5_getintofights 0.45691 0.27930 1.636 0.101863

#y5_impulsive 0.49135 0.15761 3.117 0.001824 **

#y5_preferolderkids 0.33460 0.09976 3.354 0.000796 ***

# Examine accuracy in non-imputed holdout sample

glm_probs <- data.frame(probs = predict(best.logit$BestModel, newdata = holdout,type="response"))

glm_pred <- glm_probs %>% mutate(pred = ifelse(probs>.5, "1", "0"))

glm_pred <- cbind(holdout, glm_pred)

glm_pred %>% count(pred, adhd) %>% spread(adhd, n, fill = 0)

# pred 0 1

#1 0 328 62

#2 1 108 26

#3 <NA> 163 28

#Sensitivity

26/(26+62)*100 # 29.5%

#PPV

26/(108+26)*100 # 19.4%

# Next 11 variables

data.rose <- ROSE(adhd ~ y5_screamsalot+y5_setsfires+y5_clownsaround+y5_talkstoomuch+

y5_unusuallyloud+y5_cantconcentrate+y5_cantsitstill+y5_clingsadults+y5_disobedient+y5_dontgetalongotherchldrn+y5_noguilt,

data = expl, seed = 1)$data

lowbwt.bglm <- data.rose[, c("y5_screamsalot","y5_setsfires","y5_clownsaround","y5_talkstoomuch",

"y5_unusuallyloud","y5_cantconcentrate","y5_cantsitstill","y5_clingsadults","y5_disobedient",

"y5_dontgetalongotherchldrn","y5_noguilt","adhd")]

names(lowbwt.bglm)[names(lowbwt.bglm) == "adhd"] <- "y"

lowbwt.bglm <- as.data.frame(lowbwt.bglm)

best.logit <- bestglm(lowbwt.bglm,

IC = "AIC",

family=binomial,

method = "exhaustive")

summary(best.logit$BestModel)

#y5_screamsalot 0.37088 0.14575 2.545 0.010939 *

#y5_clownsaround 0.34710 0.10298 3.371 0.000750 ***

#y5_talkstoomuch 0.15974 0.08933 1.788 0.073737 .

#y5_unusuallyloud 0.18282 0.11988 1.525 0.127246

#y5_cantconcentrate 0.51982 0.15149 3.431 0.000600 ***

#y5_cantsitstill 0.61161 0.11419 5.356 8.51e-08 ***

#y5_dontgetalongotherchldrn 0.82928 0.24138 3.436 0.000591 ***

#y5_noguilt 0.27518 0.14392 1.912 0.055866 .

# Examine accuracy in non-imputed holdout sample

glm_probs <- data.frame(probs = predict(best.logit$BestModel, newdata = holdout,type="response"))

glm_pred <- glm_probs %>% mutate(pred = ifelse(probs>.5, "1", "0"))

glm_pred <- cbind(holdout, glm_pred)

glm_pred %>% count(pred, adhd) %>% spread(adhd, n, fill = 0)

# pred 0 1

#1 0 345 44

#2 1 65 40

#3 <NA> 189 32

#Sensitivity

40/(40+44)*100 # 47.6%

#PPV

40/(65+40)*100 # 38.1%

# Last 9 variables

data.rose <- ROSE(adhd ~ y5_troublefallingasleep+y5_nervous+y5_stubborn+y5_suddenmoodswings+y5_tempertantrums+y5_anxious+

y5_wantsalotattention+y5_actstooyoungforage+y5_mdoc_highnegativeaffect,

data = expl, seed = 1)$data

lowbwt.bglm <- data.rose[, c("y5_troublefallingasleep","y5_nervous","y5_stubborn","y5_suddenmoodswings","y5_tempertantrums","y5_anxious",

"y5_wantsalotattention","y5_actstooyoungforage","y5_mdoc_highnegativeaffect","adhd")]

names(lowbwt.bglm)[names(lowbwt.bglm) == "adhd"] <- "y"

lowbwt.bglm <- as.data.frame(lowbwt.bglm)

best.logit <- bestglm(lowbwt.bglm,

IC = "AIC",

family=binomial,

method = "exhaustive")

summary(best.logit$BestModel)

#y5_nervous 1.01416 0.32021 3.167 0.001539 **

#y5_stubborn 0.49641 0.16618 2.987 0.002816 **

#y5_tempertantrums 0.32184 0.15338 2.098 0.035879 *

#y5_anxious -0.37915 0.22496 -1.685 0.091909 .

#y5_wantsalotattention 0.47065 0.10454 4.502 6.72e-06 ***

#y5_actstooyoungforage 1.29721 0.33768 3.842 0.000122 ***

# Examine accuracy in non-imputed holdout sample

glm_probs <- data.frame(probs = predict(best.logit$BestModel, newdata = holdout,type="response"))

glm_pred <- glm_probs %>% mutate(pred = ifelse(probs>.5, "1", "0"))

glm_pred <- cbind(holdout, glm_pred)

glm_pred %>% count(pred, adhd) %>% spread(adhd, n, fill = 0)

# pred 0 1

#1 0 362 49

#2 1 123 42

#3 <NA> 114 25

#Sensitivity

42/(42+49)*100 # 46.2%

#PPV

42/(123+42)*100 # 25.5%

### Full model

data.rose <- ROSE(adhd ~ y5_destroyownthings+y5_disobedientschool+y5_impulsive+y5_preferolderkids+y5_clownsaround+y5_cantconcentrate+

y5_cantsitstill+y5_dontgetalongotherchldrn+

y5_nervous+y5_stubborn+y5_wantsalotattention+y5_actstooyoungforage,

data = expl, seed = 1)$data

lowbwt.bglm <- data.rose[, c("y5_destroyownthings","y5_disobedientschool","y5_impulsive","y5_preferolderkids","y5_clownsaround",

"y5_cantconcentrate","y5_cantsitstill","y5_dontgetalongotherchldrn","y5_nervous","y5_stubborn",

"y5_wantsalotattention","y5_actstooyoungforage","adhd")]

names(lowbwt.bglm)[names(lowbwt.bglm) == "adhd"] <- "y"

lowbwt.bglm <- as.data.frame(lowbwt.bglm)

best.logit <- bestglm(lowbwt.bglm,

IC = "AIC",

family=binomial,

method = "exhaustive")

summary(best.logit$BestModel)

#y5_disobedientschool 1.05030 0.22903 4.586 4.52e-06 ***

#y5_impulsive 0.71499 0.18252 3.917 8.95e-05 ***

#y5_preferolderkids 0.37487 0.10722 3.496 0.000472 ***

#y5_clownsaround 0.18376 0.10141 1.812 0.069976 .

#y5_cantsitstill 0.61471 0.11051 5.562 2.66e-08 ***

#y5_dontgetalongotherchldrn 0.37660 0.23395 1.610 0.107456

#y5_stubborn 0.54206 0.13144 4.124 3.72e-05 ***

#y5_wantsalotattention 0.28042 0.09030 3.105 0.001901 **

#y5_actstooyoungforage 0.81605 0.25020 3.262 0.001108 **

# Examine accuracy in non-imputed holdout sample

glm_probs <- data.frame(probs = predict(best.logit$BestModel, newdata = holdout,type="response"))

glm_pred <- glm_probs %>% mutate(pred = ifelse(probs>.5, "1", "0"))

glm_pred <- cbind(holdout, glm_pred)

glm_pred %>% count(pred, adhd) %>% spread(adhd, n, fill = 0)

# pred 0 1

#1 0 336 48

#2 1 68 33

#3 <NA> 195 35

#Sensitivity

33/(33+48)*100 # 40.7%

#PPV

33/(68+33)*100 # 32.7%

#### Year 5 TEACHER

ry5_teacher_seldomworktobestability <- glm(adhd~male+y5_teacher_seldomworktobestability, data=expl, family="binomial")

ry5_teacher_diagnoseddisability <- glm(adhd~male+y5_teacher_diagnoseddisability, data=expl, family="binomial")

ry5_teacher_behindinschool <- glm(adhd~male+y5_teacher_behindinschool, data=expl, family="binomial")

ry5_teacher_understandstory <- glm(adhd~male+y5_teacher_understandstory, data=expl, family="binomial")

ry5_teacher_easilynameletters <- glm(adhd~male+y5_teacher_easilynameletters, data=expl, family="binomial")

ry5_teacher_readssimplebooks <- glm(adhd~male+y5_teacher_readssimplebooks, data=expl, family="binomial")

ry5_teacher_understconvofprint <- glm(adhd~male+y5_teacher_understconvofprint, data=expl, family="binomial")

ry5_teacher_recogndiffpeople <- glm(adhd~male+y5_teacher_recogndiffpeople, data=expl, family="binomial")

ry5_teacher_explaneobserv <- glm(adhd~male+y5_teacher_explaneobserv, data=expl, family="binomial")

ry5_teacher_sortscompmath <- glm(adhd~male+y5_teacher_sortscompmath, data=expl, family="binomial")

ry5_teacher_relofquantities <- glm(adhd~male+y5_teacher_relofquantities, data=expl, family="binomial")

ry5_teacher_varietyinmath <- glm(adhd~male+y5_teacher_varietyinmath, data=expl, family="binomial")

ry5_teacher_belowaverlanguage <- glm(adhd~male+y5_teacher_belowaverlanguage, data=expl, family="binomial")

ry5_teacher_belowaversocialsci <- glm(adhd~male+y5_teacher_belowaversocialsci, data=expl, family="binomial")

ry5_teacher_belowavermath <- glm(adhd~male+y5_teacher_belowavermath, data=expl, family="binomial")

ry5_teacher_activestrucutured <- glm(adhd~male+y5_teacher_activestrucutured, data=expl, family="binomial")

ry5_teacher_activeunstrucutured <- glm(adhd~male+y5_teacher_activeunstrucutured, data=expl, family="binomial")

ry5_teacher_discussproblsparents <- glm(adhd~male+y5_teacher_discussproblsparents, data=expl, family="binomial")

summary(ry5_teacher_seldomworktobestability)#***

summary(ry5_teacher_diagnoseddisability)#***

summary(ry5_teacher_behindinschool)#*

summary(ry5_teacher_understandstory)#*

summary(ry5_teacher_easilynameletters)#**

summary(ry5_teacher_readssimplebooks)#*

summary(ry5_teacher_understconvofprint)#***

summary(ry5_teacher_recogndiffpeople)#***

summary(ry5_teacher_explaneobserv)#*

summary(ry5_teacher_sortscompmath)#**

summary(ry5_teacher_relofquantities)#**

summary(ry5_teacher_varietyinmath)#***

summary(ry5_teacher_belowaverlanguage)#***

summary(ry5_teacher_belowaversocialsci)#**

summary(ry5_teacher_belowavermath)#***

summary(ry5_teacher_activestrucutured)#***

summary(ry5_teacher_activeunstrucutured)#***

summary(ry5_teacher_discussproblsparents)#***

CrossTable(expl$adhd, expl$y5_teacher_seldomworktobestability)

CrossTable(expl$adhd, expl$y5_teacher_diagnoseddisability)

CrossTable(expl$adhd, expl$y5_teacher_easilynameletters)

CrossTable(expl$adhd, expl$y5_teacher_understconvofprint)

CrossTable(expl$adhd, expl$y5_teacher_recogndiffpeople)

CrossTable(expl$adhd, expl$y5_teacher_sortscompmath)

CrossTable(expl$adhd, expl$y5_teacher_relofquantities)

CrossTable(expl$adhd, expl$y5_teacher_varietyinmath)

CrossTable(expl$adhd, expl$y5_teacher_belowaverlanguage)

CrossTable(expl$adhd, expl$y5_teacher_belowaversocialsci)

CrossTable(expl$adhd, expl$y5_teacher_belowavermath)

CrossTable(expl$adhd, expl$y5_teacher_activestrucutured)

CrossTable(expl$adhd, expl$y5_teacher_activeunstrucutured)

CrossTable(expl$adhd, expl$y5_teacher_discussproblsparents)

### Full model

data.rose <- ROSE(adhd ~ y5_teacher_seldomworktobestability+y5_teacher_diagnoseddisability+y5_teacher_easilynameletters+y5_teacher_understconvofprint+

y5_teacher_recogndiffpeople+y5_teacher_sortscompmath+y5_teacher_relofquantities+y5_teacher_varietyinmath+y5_teacher_belowaverlanguage+

y5_teacher_belowaversocialsci+y5_teacher_belowavermath+y5_teacher_activestrucutured+y5_teacher_activeunstrucutured+y5_teacher_discussproblsparents,

data = expl, seed = 1)$data

lowbwt.bglm <- data.rose[, c("y5_teacher_seldomworktobestability","y5_teacher_diagnoseddisability","y5_teacher_easilynameletters","y5_teacher_understconvofprint",

"y5_teacher_recogndiffpeople","y5_teacher_sortscompmath","y5_teacher_relofquantities","y5_teacher_varietyinmath","y5_teacher_belowaverlanguage",

"y5_teacher_belowaversocialsci","y5_teacher_belowavermath","y5_teacher_activestrucutured","y5_teacher_activeunstrucutured","y5_teacher_discussproblsparents","adhd")]

names(lowbwt.bglm)[names(lowbwt.bglm) == "adhd"] <- "y"

lowbwt.bglm <- as.data.frame(lowbwt.bglm)

best.logit <- bestglm(lowbwt.bglm,

IC = "AIC",

family=binomial,

method = "exhaustive")

summary(best.logit$BestModel)

#y5_teacher_seldomworktobestability 0.6392 0.1877 3.406 0.000659 ***

#y5_teacher_diagnoseddisability 1.1386 0.2283 4.988 6.10e-07 ***

#y5_teacher_sortscompmath 0.4546 0.2742 1.658 0.097335 .

#y5_teacher_relofquantities -0.8165 0.2763 -2.955 0.003125 **

#y5_teacher_varietyinmath 0.5472 0.2231 2.453 0.014162 *

#y5_teacher_belowaverlanguage -0.3041 0.1980 -1.536 0.124610

#y5_teacher_belowaversocialsci 0.3620 0.2163 1.673 0.094285 .

#y5_teacher_activeunstrucutured 0.7049 0.2599 2.713 0.006676 **

#y5_teacher_discussproblsparents 0.6878 0.1590 4.326 1.52e-05 ***

# Examine accuracy in non-imputed holdout sample

glm_probs <- data.frame(probs = predict(best.logit$BestModel, newdata = holdout,type="response"))

glm_pred <- glm_probs %>% mutate(pred = ifelse(probs>.5, "1", "0"))

glm_pred <- cbind(holdout, glm_pred)

glm_pred %>% count(pred, adhd) %>% spread(adhd, n, fill = 0)

# pred 0 1

#1 0 104 7

#2 1 28 13

#3 <NA> 467 96

#Sensitivity

13/(13+7)*100 # 65.0%

#PPV

13/(28+13)*100 # 31.7%

### ### ### ### ### ### ### ###

### Year 9 factors

### ### ### ### ### ### ### ###

#### Parent

ry9_parent_easychangebetweenactivities <-glm(adhd~male+y9_parent_easychangebetweenactivities,data=expl,family="binomial")

ry9_parent_cantgetmindoffthoughts <-glm(adhd~male+y9_parent_cantgetmindoffthoughts,data=expl,family="binomial")

ry9_parent_cantgetalongotherkids <-glm(adhd~male+y9_parent_cantgetalongotherkids,data=expl,family="binomial")

ry9_parent_ratheralonethanothers <-glm(adhd~male+y9_parent_ratheralonethanothers,data=expl,family="binomial")

ry9_parent_playssexpartstoomuch <-glm(adhd~male+y9_parent_playssexpartstoomuch,data=expl,family="binomial")

ry9_parent_destroysothersthings <-glm(adhd~male+y9_parent_destroysothersthings,data=expl,family="binomial")

ry9_parent_talkstoomuchaboutsex <-glm(adhd~male+y9_parent_talkstoomuchaboutsex,data=expl,family="binomial")

ry9_parent_sleepslessthanothers <-glm(adhd~male+y9_parent_sleepslessthanothers,data=expl,family="binomial")

ry9_parent_understandotherfeelings <-glm(adhd~male+y9_parent_understandotherfeelings,data=expl,family="binomial")

ry9_parent_acceptifriendsdieasinplay <-glm(adhd~male+y9_parent_acceptifriendsdieasinplay,data=expl,family="binomial")

ry9_parent_actotooyoung <-glm(adhd~male+y9_parent_actotooyoung,data=expl,family="binomial")

ry9_parent_drinksalcohol <-glm(adhd~male+y9_parent_drinksalcohol,data=expl,family="binomial")

ry9_parent_arguesalot <-glm(adhd~male+y9_parent_arguesalot,data=expl,family="binomial")

ry9_parent_failstofinish <-glm(adhd~male+y9_parent_failstofinish,data=expl,family="binomial")

ry9_parent_enjoysverylittle <-glm(adhd~male+y9_parent_enjoysverylittle,data=expl,family="binomial")

ry9_parent_brags <-glm(adhd~male+y9_parent_brags,data=expl,family="binomial")

ry9_parent_cantconcentrate <-glm(adhd~male+y9_parent_cantconcentrate,data=expl,family="binomial")

ry9_parent_restless <-glm(adhd~male+y9_parent_restless,data=expl,family="binomial")

ry9_parent_clingsadults <-glm(adhd~male+y9_parent_clingsadults,data=expl,family="binomial")

ry9_parent_loneliness <-glm(adhd~male+y9_parent_loneliness,data=expl,family="binomial")

ry9_parent_confused <-glm(adhd~male+y9_parent_confused,data=expl,family="binomial")

ry9_parent_criesalot <-glm(adhd~male+y9_parent_criesalot,data=expl,family="binomial")

ry9_parent_cruelanimals <-glm(adhd~male+y9_parent_cruelanimals,data=expl,family="binomial")

ry9_parent_cruelothers <-glm(adhd~male+y9_parent_cruelothers,data=expl,family="binomial")

ry9_parent_daydreams <-glm(adhd~male+y9_parent_daydreams,data=expl,family="binomial")

ry9_parent_harmsself <-glm(adhd~male+y9_parent_harmsself,data=expl,family="binomial")

ry9_parent_demandsattention <-glm(adhd~male+y9_parent_demandsattention,data=expl,family="binomial")

ry9_parent_destrousownthings <-glm(adhd~male+y9_parent_destrousownthings,data=expl,family="binomial")

ry9_parent_disobed_home <-glm(adhd~male+y9_parent_disobed_home,data=expl,family="binomial")

ry9_parent_disob_school <-glm(adhd~male+y9_parent_disob_school,data=expl,family="binomial")

ry9_parent_guiltyaftermisbeh <-glm(adhd~male+y9_parent_guiltyaftermisbeh,data=expl,family="binomial")

ry9_parent_jelaous <-glm(adhd~male+y9_parent_jelaous,data=expl,family="binomial")

ry9_parent_breakrules <-glm(adhd~male+y9_parent_breakrules,data=expl,family="binomial")

ry9_parent_phobias <-glm(adhd~male+y9_parent_phobias,data=expl,family="binomial")

ry9_parent_feargoingschool <-glm(adhd~male+y9_parent_feargoingschool,data=expl,family="binomial")

ry9_parent_feardosomthngbad <-glm(adhd~male+y9_parent_feardosomthngbad,data=expl,family="binomial")

ry9_parent_feelshastobeperfect <-glm(adhd~male+y9_parent_feelshastobeperfect,data=expl,family="binomial")

ry9_parent_complainsnooneloves <-glm(adhd~male+y9_parent_complainsnooneloves,data=expl,family="binomial")

ry9_parent_feelsotheroutotget <-glm(adhd~male+y9_parent_feelsotheroutotget,data=expl,family="binomial")

ry9_parent_feelworthless <-glm(adhd~male+y9_parent_feelworthless,data=expl,family="binomial")

ry9_parent_accidentprone <-glm(adhd~male+y9_parent_accidentprone,data=expl,family="binomial")

ry9_parent_getinmanyfights <-glm(adhd~male+y9_parent_getinmanyfights,data=expl,family="binomial")

ry9_parent_getteased <-glm(adhd~male+y9_parent_getteased,data=expl,family="binomial")

ry9_parent_hangsothertrouble <-glm(adhd~male+y9_parent_hangsothertrouble,data=expl,family="binomial")

ry9_parent_hearvocies <-glm(adhd~male+y9_parent_hearvocies,data=expl,family="binomial")

ry9_parent_impulsive <-glm(adhd~male+y9_parent_impulsive,data=expl,family="binomial")

ry9_parent_lies <-glm(adhd~male+y9_parent_lies,data=expl,family="binomial")

ry9_parent_nervous <-glm(adhd~male+y9_parent_nervous,data=expl,family="binomial")

ry9_parent_nervousmovements <-glm(adhd~male+y9_parent_nervousmovements,data=expl,family="binomial")

ry9_parent_nightmares <-glm(adhd~male+y9_parent_nightmares,data=expl,family="binomial")

ry9_parent_notlikedotherkids <-glm(adhd~male+y9_parent_notlikedotherkids,data=expl,family="binomial")

ry9_parent_anxious <-glm(adhd~male+y9_parent_anxious,data=expl,family="binomial")

ry9_parent_dizzy <-glm(adhd~male+y9_parent_dizzy,data=expl,family="binomial")

ry9_parent_tooguilty <-glm(adhd~male+y9_parent_tooguilty,data=expl,family="binomial")

ry9_parent_attacksphysically <-glm(adhd~male+y9_parent_attacksphysically,data=expl,family="binomial")

ry9_parent_picksnoseskin <-glm(adhd~male+y9_parent_picksnoseskin,data=expl,family="binomial")

ry9_parent_playssexpartspublic <-glm(adhd~male+y9_parent_playssexpartspublic,data=expl,family="binomial")

ry9_parent_poorinschool <-glm(adhd~male+y9_parent_poorinschool,data=expl,family="binomial")

ry9_parent_clumsy <-glm(adhd~male+y9_parent_clumsy,data=expl,family="binomial")

ry9_parent_prefersolderkids <-glm(adhd~male+y9_parent_prefersolderkids,data=expl,family="binomial")

ry9_parent_prefersyoungerkids <-glm(adhd~male+y9_parent_prefersyoungerkids,data=expl,family="binomial")

ry9_parent_refusestotalk <-glm(adhd~male+y9_parent_refusestotalk,data=expl,family="binomial")

ry9_parent_compulsions <-glm(adhd~male+y9_parent_compulsions,data=expl,family="binomial")

ry9_parent_runsawayfromhome <-glm(adhd~male+y9_parent_runsawayfromhome,data=expl,family="binomial")

ry9_parent_screams <-glm(adhd~male+y9_parent_screams,data=expl,family="binomial")

ry9_parent_secretive <-glm(adhd~male+y9_parent_secretive,data=expl,family="binomial")

ry9_parent_seesthiongsnotthere <-glm(adhd~male+y9_parent_seesthiongsnotthere,data=expl,family="binomial")

ry9_parent_selfembarassed <-glm(adhd~male+y9_parent_selfembarassed,data=expl,family="binomial")

ry9_parent_setsfires <-glm(adhd~male+y9_parent_setsfires,data=expl,family="binomial")

ry9_parent_sexualproblems <-glm(adhd~male+y9_parent_sexualproblems,data=expl,family="binomial")

ry9_parent_clowns <-glm(adhd~male+y9_parent_clowns,data=expl,family="binomial")

ry9_parent_shytimid <-glm(adhd~male+y9_parent_shytimid,data=expl,family="binomial")

ry9_parent_easydistracted <-glm(adhd~male+y9_parent_easydistracted,data=expl,family="binomial")

ry9_parent_speechproblem <-glm(adhd~male+y9_parent_speechproblem,data=expl,family="binomial")

ry9_parent_staresblankly <-glm(adhd~male+y9_parent_staresblankly,data=expl,family="binomial")

ry9_parent_stealshome <-glm(adhd~male+y9_parent_stealshome,data=expl,family="binomial")

ry9_parent_stealsoutsidehome <-glm(adhd~male+y9_parent_stealsoutsidehome,data=expl,family="binomial")

ry9_parent_hoards <-glm(adhd~male+y9_parent_hoards,data=expl,family="binomial")

ry9_parent_strangebehaviors <-glm(adhd~male+y9_parent_strangebehaviors,data=expl,family="binomial")

ry9_parent_strangeideas <-glm(adhd~male+y9_parent_strangeideas,data=expl,family="binomial")

ry9_parent_stubborn <-glm(adhd~male+y9_parent_stubborn,data=expl,family="binomial")

ry9_parent_moodswings <-glm(adhd~male+y9_parent_moodswings,data=expl,family="binomial")

ry9_parent_sulks <-glm(adhd~male+y9_parent_sulks,data=expl,family="binomial")

ry9_parent_supsicious <-glm(adhd~male+y9_parent_supsicious,data=expl,family="binomial")

ry9_parent_obsecenlanguage <-glm(adhd~male+y9_parent_obsecenlanguage,data=expl,family="binomial")

ry9_parent_talkskillingself <-glm(adhd~male+y9_parent_talkskillingself,data=expl,family="binomial")

ry9_parent_walksinsleep <-glm(adhd~male+y9_parent_walksinsleep,data=expl,family="binomial")

ry9_parent_talktoomuch <-glm(adhd~male+y9_parent_talktoomuch,data=expl,family="binomial")

ry9_parent_teasesalot <-glm(adhd~male+y9_parent_teasesalot,data=expl,family="binomial")

ry9_parent_tempertantrums <-glm(adhd~male+y9_parent_tempertantrums,data=expl,family="binomial")

ry9_parent_threatenspeople <-glm(adhd~male+y9_parent_threatenspeople,data=expl,family="binomial")

ry9_parent_smokes <-glm(adhd~male+y9_parent_smokes,data=expl,family="binomial")

ry9_parent_troublesleeping <-glm(adhd~male+y9_parent_troublesleeping,data=expl,family="binomial")

ry9_parent_skipsschool <-glm(adhd~male+y9_parent_skipsschool,data=expl,family="binomial")

ry9_parent_underactive <-glm(adhd~male+y9_parent_underactive,data=expl,family="binomial")

ry9_parent_sad <-glm(adhd~male+y9_parent_sad,data=expl,family="binomial")

ry9_parent_unusuallyloud <-glm(adhd~male+y9_parent_unusuallyloud,data=expl,family="binomial")

ry9_parent_alcohol <-glm(adhd~male+y9_parent_alcohol,data=expl,family="binomial")

ry9_parent_vandalizes <-glm(adhd~male+y9_parent_vandalizes,data=expl,family="binomial")

ry9_parent_whines <-glm(adhd~male+y9_parent_whines,data=expl,family="binomial")

ry9_parent_withdrawn <-glm(adhd~male+y9_parent_withdrawn,data=expl,family="binomial")

ry9_parent_worries <-glm(adhd~male+y9_parent_worries,data=expl,family="binomial")

ry9_parent_sympatheticothers <-glm(adhd~male+y9_parent_sympatheticothers,data=expl,family="binomial")

ry9_parent_openwhatshewants <-glm(adhd~male+y9_parent_openwhatshewants,data=expl,family="binomial")

ry9_parent_joingroupswhentoldso <-glm(adhd~male+y9_parent_joingroupswhentoldso,data=expl,family="binomial")

ry9_parent_makesfriendseasily <-glm(adhd~male+y9_parent_makesfriendseasily,data=expl,family="binomial")

ry9_parent_selfconfident <-glm(adhd~male+y9_parent_selfconfident,data=expl,family="binomial")

ry9_parent_interestindiffthings <-glm(adhd~male+y9_parent_interestindiffthings,data=expl,family="binomial")

ry9_parent_startconversations <-glm(adhd~male+y9_parent_startconversations,data=expl,family="binomial")

ry9_parent_likedbyothers <-glm(adhd~male+y9_parent_likedbyothers,data=expl,family="binomial")

ry9_parent_invitesotherstohome <-glm(adhd~male+y9_parent_invitesotherstohome,data=expl,family="binomial")

ry9_parent_reportsaccappropr <-glm(adhd~male+y9_parent_reportsaccappropr,data=expl,family="binomial")

summary(ry9_parent_easychangebetweenactivities)#**

summary(ry9_parent_cantgetmindoffthoughts)#***

summary(ry9_parent_cantgetalongotherkids)

summary(ry9_parent_ratheralonethanothers)#***

summary(ry9_parent_playssexpartstoomuch)

summary(ry9_parent_destroysothersthings)#***

summary(ry9_parent_talkstoomuchaboutsex)

summary(ry9_parent_sleepslessthanothers)#***

summary(ry9_parent_understandotherfeelings)#***

summary(ry9_parent_acceptifriendsdieasinplay)#***

summary(ry9_parent_actotooyoung)#***

summary(ry9_parent_drinksalcohol)

summary(ry9_parent_arguesalot)#***

summary(ry9_parent_failstofinish)#***

summary(ry9_parent_enjoysverylittle)

summary(ry9_parent_brags)

summary(ry9_parent_cantconcentrate)#***

summary(ry9_parent_restless)#***

summary(ry9_parent_clingsadults)#***

summary(ry9_parent_loneliness)#**

summary(ry9_parent_confused)#***

summary(ry9_parent_criesalot)#***

summary(ry9_parent_cruelanimals)#***

summary(ry9_parent_cruelothers)#***

summary(ry9_parent_daydreams)#***

summary(ry9_parent_harmsself)

summary(ry9_parent_demandsattention)#***

summary(ry9_parent_destrousownthings)#***

summary(ry9_parent_disobed_home)#***

summary(ry9_parent_disob_school)#***

summary(ry9_parent_guiltyaftermisbeh)

summary(ry9_parent_jelaous)#*

summary(ry9_parent_breakrules)#***

summary(ry9_parent_phobias)#**

summary(ry9_parent_feargoingschool)

summary(ry9_parent_feardosomthngbad)

summary(ry9_parent_feelshastobeperfect)

summary(ry9_parent_complainsnooneloves)#***

summary(ry9_parent_feelsotheroutotget)

summary(ry9_parent_feelworthless)

summary(ry9_parent_accidentprone)#**

summary(ry9_parent_getinmanyfights)#***

summary(ry9_parent_getteased)#***

summary(ry9_parent_hangsothertrouble)#*

summary(ry9_parent_hearvocies)#*

summary(ry9_parent_impulsive)#***

summary(ry9_parent_lies)#***

summary(ry9_parent_nervous)#***

summary(ry9_parent_nervousmovements)#***

summary(ry9_parent_nightmares)#**

summary(ry9_parent_notlikedotherkids)#*

summary(ry9_parent_anxious)#***

summary(ry9_parent_dizzy)#***

summary(ry9_parent_tooguilty)

summary(ry9_parent_attacksphysically)

summary(ry9_parent_picksnoseskin)#*

summary(ry9_parent_playssexpartspublic)

summary(ry9_parent_poorinschool)#**

summary(ry9_parent_clumsy)

summary(ry9_parent_prefersolderkids)

summary(ry9_parent_prefersyoungerkids)

summary(ry9_parent_refusestotalk)

summary(ry9_parent_compulsions)#***

summary(ry9_parent_runsawayfromhome)

summary(ry9_parent_screams)#***

summary(ry9_parent_secretive)#***

summary(ry9_parent_seesthiongsnotthere)

summary(ry9_parent_selfembarassed)#***

summary(ry9_parent_setsfires)

summary(ry9_parent_sexualproblems)

summary(ry9_parent_clowns)#***

summary(ry9_parent_shytimid)

summary(ry9_parent_easydistracted)#***

summary(ry9_parent_speechproblem)#***

summary(ry9_parent_staresblankly)#*

summary(ry9_parent_stealshome)

summary(ry9_parent_stealsoutsidehome)

summary(ry9_parent_hoards)#**

summary(ry9_parent_strangebehaviors)#*

summary(ry9_parent_strangeideas)

summary(ry9_parent_stubborn)#***

summary(ry9_parent_moodswings)#***

summary(ry9_parent_sulks)

summary(ry9_parent_supsicious)

summary(ry9_parent_obsecenlanguage)

summary(ry9_parent_talkskillingself)

summary(ry9_parent_walksinsleep)

summary(ry9_parent_talktoomuch)#***

summary(ry9_parent_teasesalot)#**

summary(ry9_parent_tempertantrums)#***

summary(ry9_parent_threatenspeople)

summary(ry9_parent_smokes)

summary(ry9_parent_troublesleeping)#***

summary(ry9_parent_skipsschool)

summary(ry9_parent_underactive)

summary(ry9_parent_sad)

summary(ry9_parent_unusuallyloud)#***

summary(ry9_parent_alcohol)

summary(ry9_parent_vandalizes)

summary(ry9_parent_whines)#***

summary(ry9_parent_withdrawn)#**

summary(ry9_parent_worries)#*

summary(ry9_parent_sympatheticothers)#***

summary(ry9_parent_openwhatshewants)

summary(ry9_parent_joingroupswhentoldso)

summary(ry9_parent_makesfriendseasily)#***

summary(ry9_parent_selfconfident)#***

summary(ry9_parent_interestindiffthings)#***

summary(ry9_parent_startconversations)

summary(ry9_parent_likedbyothers)#***

summary(ry9_parent_invitesotherstohome)#**

summary(ry9_parent_reportsaccappropr)#***

CrossTable(expl$adhd, expl$y9_parent_easychangebetweenactivities)

CrossTable(expl$adhd, expl$y9_parent_cantgetmindoffthoughts)

CrossTable(expl$adhd, expl$y9_parent_ratheralonethanothers)

CrossTable(expl$adhd, expl$y9_parent_destroysothersthings)

CrossTable(expl$adhd, expl$y9_parent_sleepslessthanothers)

CrossTable(expl$adhd, expl$y9_parent_understandotherfeelings)

CrossTable(expl$adhd, expl$y9_parent_acceptifriendsdieasinplay)

CrossTable(expl$adhd, expl$y9_parent_actotooyoung)

CrossTable(expl$adhd, expl$y9_parent_arguesalot)

CrossTable(expl$adhd, expl$y9_parent_failstofinish)

CrossTable(expl$adhd, expl$y9_parent_cantconcentrate)

CrossTable(expl$adhd, expl$y9_parent_restless)

CrossTable(expl$adhd, expl$y9_parent_clingsadults)

CrossTable(expl$adhd, expl$y9_parent_loneliness)

CrossTable(expl$adhd, expl$y9_parent_confused)

CrossTable(expl$adhd, expl$y9_parent_criesalot)

CrossTable(expl$adhd, expl$y9_parent_cruelanimals)

CrossTable(expl$adhd, expl$y9_parent_cruelothers)

CrossTable(expl$adhd, expl$y9_parent_daydreams)

CrossTable(expl$adhd, expl$y9_parent_demandsattention)

CrossTable(expl$adhd, expl$y9_parent_destrousownthings)

CrossTable(expl$adhd, expl$y9_parent_disobed_home)

CrossTable(expl$adhd, expl$y9_parent_disob_school)

CrossTable(expl$adhd, expl$y9_parent_jelaous)

CrossTable(expl$adhd, expl$y9_parent_breakrules)

CrossTable(expl$adhd, expl$y9_parent_phobias)

CrossTable(expl$adhd, expl$y9_parent_complainsnooneloves)

CrossTable(expl$adhd, expl$y9_parent_accidentprone)

CrossTable(expl$adhd, expl$y9_parent_getinmanyfights)

CrossTable(expl$adhd, expl$y9_parent_getteased)

CrossTable(expl$adhd, expl$y9_parent_impulsive)

CrossTable(expl$adhd, expl$y9_parent_lies)

CrossTable(expl$adhd, expl$y9_parent_nervous)

CrossTable(expl$adhd, expl$y9_parent_nervousmovements)

CrossTable(expl$adhd, expl$y9_parent_nightmares)

CrossTable(expl$adhd, expl$y9_parent_anxious)

CrossTable(expl$adhd, expl$y9_parent_dizzy) ## Too few to calculate

CrossTable(expl$adhd, expl$y9_parent_poorinschool)

CrossTable(expl$adhd, expl$y9_parent_compulsions)

CrossTable(expl$adhd, expl$y9_parent_screams)

CrossTable(expl$adhd, expl$y9_parent_secretive)

CrossTable(expl$adhd, expl$y9_parent_selfembarassed)

CrossTable(expl$adhd, expl$y9_parent_clowns)

CrossTable(expl$adhd, expl$y9_parent_easydistracted)

CrossTable(expl$adhd, expl$y9_parent_hoards)

CrossTable(expl$adhd, expl$y9_parent_stubborn)

CrossTable(expl$adhd, expl$y9_parent_moodswings)

CrossTable(expl$adhd, expl$y9_parent_talktoomuch)

CrossTable(expl$adhd, expl$y9_parent_teasesalot)

CrossTable(expl$adhd, expl$y9_parent_tempertantrums)

CrossTable(expl$adhd, expl$y9_parent_troublesleeping)

CrossTable(expl$adhd, expl$y9_parent_unusuallyloud)

CrossTable(expl$adhd, expl$y9_parent_whines)

CrossTable(expl$adhd, expl$y9_parent_withdrawn)

CrossTable(expl$adhd, expl$y9_parent_sympatheticothers)

CrossTable(expl$adhd, expl$y9_parent_makesfriendseasily)

CrossTable(expl$adhd, expl$y9_parent_selfconfident)

CrossTable(expl$adhd, expl$y9_parent_interestindiffthings)

CrossTable(expl$adhd, expl$y9_parent_likedbyothers)

CrossTable(expl$adhd, expl$y9_parent_invitesotherstohome)

CrossTable(expl$adhd, expl$y9_parent_reportsaccappropr)

### Accuracy

## First 13

data.rose <- ROSE(adhd ~ y9_parent_easychangebetweenactivities+y9_parent_cantgetmindoffthoughts+y9_parent_ratheralonethanothers+y9_parent_destroysothersthings+

y9_parent_sleepslessthanothers+y9_parent_understandotherfeelings+y9_parent_acceptifriendsdieasinplay+y9_parent_actotooyoung+y9_parent_arguesalot+

y9_parent_failstofinish+y9_parent_cantconcentrate+y9_parent_restless,

data = expl, seed = 1)$data

lowbwt.bglm <- data.rose[, c("y9_parent_easychangebetweenactivities","y9_parent_cantgetmindoffthoughts","y9_parent_ratheralonethanothers","y9_parent_destroysothersthings",

"y9_parent_sleepslessthanothers","y9_parent_understandotherfeelings","y9_parent_acceptifriendsdieasinplay","y9_parent_actotooyoung","y9_parent_arguesalot",

"y9_parent_failstofinish","y9_parent_cantconcentrate","y9_parent_restless","adhd")]

names(lowbwt.bglm)[names(lowbwt.bglm) == "adhd"] <- "y"

lowbwt.bglm <- as.data.frame(lowbwt.bglm)

best.logit <- bestglm(lowbwt.bglm,

IC = "AIC",

family=binomial,

method = "exhaustive")

summary(best.logit$BestModel)

#y9_parent_easychangebetweenactivities -0.40760 0.08343 -4.885 1.03e-06 ***

#y9_parent_cantgetmindoffthoughts 0.36044 0.18284 1.971 0.0487 *

#y9_parent_ratheralonethanothers 0.49907 0.26724 1.867 0.0618 .

#y9_parent_understandotherfeelings -0.13551 0.08037 -1.686 0.0918 .

#y9_parent_arguesalot 0.55797 0.12754 4.375 1.22e-05 ***

#y9_parent_failstofinish 0.63950 0.15843 4.036 5.43e-05 ***

#y9_parent_cantconcentrate 1.03653 0.12838 8.074 6.81e-16 ***

#y9_parent_restless 1.23021 0.12483 9.855 < 2e-16 ***

# Examine accuracy in non-imputed holdout sample

glm_probs <- data.frame(probs = predict(best.logit$BestModel, newdata = holdout,type="response"))

glm_pred <- glm_probs %>% mutate(pred = ifelse(probs>.5, "1", "0"))

glm_pred <- cbind(holdout, glm_pred)

glm_pred %>% count(pred, adhd) %>% spread(adhd, n, fill = 0)

# pred 0 1

#1 0 447 38

#2 1 52 57

#3 <NA> 100 21

#Sensitivity

57/(57+38)*100 # 60.0%

#PPV

57/(52+57)*100 # 52.3%

## Next 12

data.rose <- ROSE(adhd ~ y9_parent_clingsadults+y9_parent_loneliness+y9_parent_confused+y9_parent_criesalot+y9_parent_cruelanimals+y9_parent_cruelothers+

y9_parent_daydreams+y9_parent_demandsattention+y9_parent_destrousownthings+y9_parent_disobed_home+y9_parent_disob_school+y9_parent_jelaous,

data = expl, seed = 1)$data

lowbwt.bglm <- data.rose[, c("y9_parent_clingsadults","y9_parent_loneliness","y9_parent_confused","y9_parent_criesalot","y9_parent_cruelanimals","y9_parent_cruelothers",

"y9_parent_daydreams","y9_parent_demandsattention","y9_parent_destrousownthings","y9_parent_disobed_home","y9_parent_disob_school","y9_parent_jelaous","adhd")]

names(lowbwt.bglm)[names(lowbwt.bglm) == "adhd"] <- "y"

lowbwt.bglm <- as.data.frame(lowbwt.bglm)

best.logit <- bestglm(lowbwt.bglm,

IC = "AIC",

family=binomial,

method = "exhaustive")

summary(best.logit$BestModel)

#y9_parent_loneliness 0.34129 0.23254 1.468 0.142200

#y9_parent_confused 0.95365 0.29309 3.254 0.001139 **

#y9_parent_criesalot -0.49546 0.26442 -1.874 0.060967 .

#y9_parent_daydreams 0.87475 0.18502 4.728 2.27e-06 ***

#y9_parent_demandsattention 0.71428 0.12106 5.900 3.63e-09 ***

#y9_parent_destrousownthings 0.53837 0.22110 2.435 0.014893 *

#y9_parent_disobed_home 0.74050 0.20365 3.636 0.000277 ***

#y9_parent_disob_school 0.62636 0.20194 3.102 0.001924 **

# Examine accuracy in non-imputed holdout sample

glm_probs <- data.frame(probs = predict(best.logit$BestModel, newdata = holdout,type="response"))

glm_pred <- glm_probs %>% mutate(pred = ifelse(probs>.5, "1", "0"))

glm_pred <- cbind(holdout, glm_pred)

glm_pred %>% count(pred, adhd) %>% spread(adhd, n, fill = 0)

# pred 0 1

#1 0 480 64

#2 1 36 32

#3 <NA> 83 20

#Sensitivity

32/(32+64)*100 # 33.3%

#PPV

32/(36+32)*100 # 47.1%

## Next 12

data.rose <- ROSE(adhd ~ y9_parent_breakrules+y9_parent_phobias+y9_parent_complainsnooneloves+y9_parent_accidentprone+

y9_parent_getinmanyfights+y9_parent_getteased+y9_parent_impulsive+y9_parent_lies+

y9_parent_nervous+y9_parent_nervousmovements+y9_parent_nightmares+y9_parent_anxious,

data = expl, seed = 1)$data

lowbwt.bglm <- data.rose[, c("y9_parent_breakrules","y9_parent_phobias","y9_parent_complainsnooneloves","y9_parent_accidentprone",

"y9_parent_getinmanyfights","y9_parent_getteased","y9_parent_impulsive","y9_parent_lies",

"y9_parent_nervous","y9_parent_nervousmovements","y9_parent_nightmares","y9_parent_anxious","adhd")]

names(lowbwt.bglm)[names(lowbwt.bglm) == "adhd"] <- "y"

lowbwt.bglm <- as.data.frame(lowbwt.bglm)

best.logit <- bestglm(lowbwt.bglm,

IC = "AIC",

family=binomial,

method = "exhaustive")

summary(best.logit$BestModel)

#y9_parent_breakrules 0.46282 0.19941 2.321 0.02029 *

#y9_parent_phobias 0.49670 0.20796 2.388 0.01692 *

#y9_parent_complainsnooneloves 1.02560 0.32918 3.116 0.00184 **

#y9_parent_accidentprone 0.62482 0.32372 1.930 0.05359 .

#y9_parent_getteased 0.69382 0.26780 2.591 0.00957 **

#y9_parent_impulsive 0.91755 0.18904 4.854 1.21e-06 ***

#y9_parent_nervousmovements 0.94827 0.33454 2.835 0.00459 **

#y9_parent_anxious 0.72126 0.34769 2.074 0.03804 *

# Examine accuracy in non-imputed holdout sample

glm_probs <- data.frame(probs = predict(best.logit$BestModel, newdata = holdout,type="response"))

glm_pred <- glm_probs %>% mutate(pred = ifelse(probs>.5, "1", "0"))

glm_pred <- cbind(holdout, glm_pred)

glm_pred %>% count(pred, adhd) %>% spread(adhd, n, fill = 0)

# pred 0 1

#1 0 488 69

#2 1 24 28

#3 <NA> 87 19

#Sensitivity

28/(28+69)*100 # 28.9%

#PPV

28/(24+28)*100 # 53.8%

## Next 12

data.rose <- ROSE(adhd ~ y9_parent_poorinschool+y9_parent_compulsions+y9_parent_screams+y9_parent_secretive+y9_parent_selfembarassed+y9_parent_clowns+

y9_parent_easydistracted+y9_parent_hoards+y9_parent_stubborn+y9_parent_moodswings+y9_parent_talktoomuch+y9_parent_teasesalot,

data = expl, seed = 1)$data

lowbwt.bglm <- data.rose[, c("y9_parent_poorinschool","y9_parent_compulsions","y9_parent_screams","y9_parent_secretive","y9_parent_selfembarassed","y9_parent_clowns",

"y9_parent_easydistracted","y9_parent_hoards","y9_parent_stubborn","y9_parent_moodswings","y9_parent_talktoomuch","y9_parent_teasesalot","adhd")]

names(lowbwt.bglm)[names(lowbwt.bglm) == "adhd"] <- "y"

lowbwt.bglm <- as.data.frame(lowbwt.bglm)

best.logit <- bestglm(lowbwt.bglm,

IC = "AIC",

family=binomial,

method = "exhaustive")

summary(best.logit$BestModel)

#y9_parent_compulsions 0.53493 0.28008 1.910 0.0561 .

#y9_parent_screams 0.39749 0.22428 1.772 0.0763 .

#y9_parent_clowns 0.26183 0.17277 1.516 0.1296

#y9_parent_easydistracted 1.05759 0.12083 8.753 < 2e-16 ***

#y9_parent_stubborn 0.34584 0.21302 1.624 0.1045

#y9_parent_moodswings 1.24408 0.26414 4.710 2.48e-06 ***

#y9_parent_talktoomuch 0.54415 0.12560 4.332 1.48e-05 ***

# Examine accuracy in non-imputed holdout sample

glm_probs <- data.frame(probs = predict(best.logit$BestModel, newdata = holdout,type="response"))

glm_pred <- glm_probs %>% mutate(pred = ifelse(probs>.5, "1", "0"))

glm_pred <- cbind(holdout, glm_pred)

glm_pred %>% count(pred, adhd) %>% spread(adhd, n, fill = 0)

# pred 0 1

#1 0 462 46

#2 1 55 51

#3 <NA> 82 19

#Sensitivity

51/(51+46)*100 # 52.6%

#PPV

51/(55+51)*100 # 48.1%

## Last 12

data.rose <- ROSE(adhd ~ y9_parent_tempertantrums+y9_parent_troublesleeping+y9_parent_unusuallyloud+y9_parent_whines+y9_parent_withdrawn+y9_parent_sympatheticothers+

y9_parent_makesfriendseasily+y9_parent_selfconfident+y9_parent_interestindiffthings+y9_parent_likedbyothers+y9_parent_invitesotherstohome+y9_parent_reportsaccappropr,

data = expl, seed = 1)$data

lowbwt.bglm <- data.rose[, c("y9_parent_tempertantrums","y9_parent_troublesleeping","y9_parent_unusuallyloud","y9_parent_whines","y9_parent_withdrawn","y9_parent_sympatheticothers",

"y9_parent_makesfriendseasily","y9_parent_selfconfident","y9_parent_interestindiffthings","y9_parent_likedbyothers","y9_parent_invitesotherstohome","y9_parent_reportsaccappropr","adhd")]

names(lowbwt.bglm)[names(lowbwt.bglm) == "adhd"] <- "y"

lowbwt.bglm <- as.data.frame(lowbwt.bglm)

best.logit <- bestglm(lowbwt.bglm,

IC = "AIC",

family=binomial,

method = "exhaustive")

summary(best.logit$BestModel)

#y9_parent_tempertantrums 0.72975 0.14440 5.054 4.33e-07 ***

#y9_parent_troublesleeping 1.02590 0.23874 4.297 1.73e-05 ***

#y9_parent_whines 0.40989 0.17457 2.348 0.018875 *

#y9_parent_makesfriendseasily -0.21440 0.08428 -2.544 0.010959 *

#y9_parent_selfconfident -0.20870 0.08080 -2.583 0.009796 **

#y9_parent_likedbyothers -0.17682 0.08628 -2.049 0.040421 *

#y9_parent_reportsaccappropr -0.27355 0.08028 -3.407 0.000656 ***

# Examine accuracy in non-imputed holdout sample

glm_probs <- data.frame(probs = predict(best.logit$BestModel, newdata = holdout,type="response"))

glm_pred <- glm_probs %>% mutate(pred = ifelse(probs>.5, "1", "0"))

glm_pred <- cbind(holdout, glm_pred)

glm_pred %>% count(pred, adhd) %>% spread(adhd, n, fill = 0)

# pred 0 1

#1 0 384 46

#2 1 125 50

#3 <NA> 90 20

#Sensitivity

50/(50+46)*100 # 52.1%

#PPV

50/(125+50)*100 # 28.6%

###Final model in two parts, then merged

data.rose <- ROSE(adhd ~ y9_parent_easychangebetweenactivities+y9_parent_arguesalot+y9_parent_failstofinish+y9_parent_cantconcentrate+y9_parent_restless+

y9_parent_confused+y9_parent_daydreams+y9_parent_demandsattention+y9_parent_disobed_home+y9_parent_disob_school,

data = expl, seed = 1)$data

lowbwt.bglm <- data.rose[, c("y9_parent_easychangebetweenactivities","y9_parent_arguesalot","y9_parent_failstofinish","y9_parent_cantconcentrate","y9_parent_restless",

"y9_parent_confused","y9_parent_daydreams","y9_parent_demandsattention","y9_parent_disobed_home","y9_parent_disob_school","adhd")]

names(lowbwt.bglm)[names(lowbwt.bglm) == "adhd"] <- "y"

lowbwt.bglm <- as.data.frame(lowbwt.bglm)

best.logit <- bestglm(lowbwt.bglm,

IC = "AIC",

family=binomial,

method = "exhaustive")

summary(best.logit$BestModel)

#y9_parent_easychangebetweenactivities -0.24488 0.07951 -3.080 0.002071 **

#y9_parent_arguesalot 0.29576 0.12554 2.356 0.018477 *

#y9_parent_failstofinish 0.57022 0.17047 3.345 0.000823 ***

#y9_parent_cantconcentrate 0.90292 0.12503 7.221 5.15e-13 ***

#y9_parent_restless 1.06101 0.11696 9.072 < 2e-16 ***

#y9_parent_demandsattention 0.39441 0.12857 3.068 0.002157 **

# Examine accuracy in non-imputed holdout sample

glm_probs <- data.frame(probs = predict(best.logit$BestModel, newdata = holdout,type="response"))

glm_pred <- glm_probs %>% mutate(pred = ifelse(probs>.5, "1", "0"))

glm_pred <- cbind(holdout, glm_pred)

glm_pred %>% count(pred, adhd) %>% spread(adhd, n, fill = 0)

# pred 0 1

#1 0 466 39

#2 1 41 56

#3 <NA> 92 21

#Sensitivity

56/(56+39)*100 # 58.9%

#PPV

56/(41+56)*100 # 57.7%

data.rose <- ROSE(adhd ~ y9_parent_complainsnooneloves+y9_parent_getteased+y9_parent_impulsive+y9_parent_nervousmovements+y9_parent_easydistracted+

y9_parent_moodswings+y9_parent_talktoomuch+y9_parent_tempertantrums+y9_parent_troublesleeping+y9_parent_selfconfident+y9_parent_reportsaccappropr,

data = expl, seed = 1)$data

lowbwt.bglm <- data.rose[, c("y9_parent_complainsnooneloves","y9_parent_getteased","y9_parent_impulsive","y9_parent_nervousmovements","y9_parent_easydistracted",

"y9_parent_moodswings","y9_parent_talktoomuch","y9_parent_tempertantrums","y9_parent_troublesleeping","y9_parent_selfconfident","y9_parent_reportsaccappropr","adhd")]

names(lowbwt.bglm)[names(lowbwt.bglm) == "adhd"] <- "y"

lowbwt.bglm <- as.data.frame(lowbwt.bglm)

best.logit <- bestglm(lowbwt.bglm,

IC = "AIC",

family=binomial,

method = "exhaustive")

summary(best.logit$BestModel)

#y9_parent_impulsive 0.84106 0.21054 3.995 6.48e-05 ***

#y9_parent_nervousmovements 0.64363 0.38705 1.663 0.09633 .

#y9_parent_easydistracted 0.89576 0.12549 7.138 9.46e-13 ***

#y9_parent_moodswings 0.57577 0.23668 2.433 0.01499 *

#y9_parent_talktoomuch 0.61282 0.12355 4.960 7.04e-07 ***

#y9_parent_tempertantrums 0.50973 0.16418 3.105 0.00191 **

#y9_parent_troublesleeping 0.48229 0.24589 1.961 0.04983 *

#y9_parent_selfconfident -0.18021 0.07584 -2.376 0.01749 *

#y9_parent_reportsaccappropr -0.43319 0.07770 -5.575 2.47e-08 ***

# Examine accuracy in non-imputed holdout sample

glm_probs <- data.frame(probs = predict(best.logit$BestModel, newdata = holdout,type="response"))

glm_pred <- glm_probs %>% mutate(pred = ifelse(probs>.5, "1", "0"))

glm_pred <- cbind(holdout, glm_pred)

glm_pred %>% count(pred, adhd) %>% spread(adhd, n, fill = 0)

# pred 0 1

#1 0 466 49

#2 1 45 47

#3 <NA> 88 20

#Sensitivity

47/(47+49)*100 # 49.0%

#PPV

47/(45+47)*100 # 51.1%

### And final

data.rose <- ROSE(adhd ~ y9_parent_easychangebetweenactivities+y9_parent_failstofinish+y9_parent_cantconcentrate+y9_parent_restless+y9_parent_demandsattention+

y9_parent_impulsive+y9_parent_easydistracted+y9_parent_talktoomuch+y9_parent_tempertantrums+y9_parent_reportsaccappropr,

data = expl, seed = 1)$data

lowbwt.bglm <- data.rose[, c("y9_parent_easychangebetweenactivities","y9_parent_failstofinish","y9_parent_cantconcentrate","y9_parent_restless","y9_parent_demandsattention",

"y9_parent_impulsive","y9_parent_easydistracted","y9_parent_talktoomuch","y9_parent_tempertantrums","y9_parent_reportsaccappropr","adhd")]

names(lowbwt.bglm)[names(lowbwt.bglm) == "adhd"] <- "y"

lowbwt.bglm <- as.data.frame(lowbwt.bglm)

best.logit <- bestglm(lowbwt.bglm,

IC = "AIC",

family=binomial,

method = "exhaustive")

summary(best.logit$BestModel)

#y9_parent_easychangebetweenactivities -0.15339 0.08212 -1.868 0.061791 .

#y9_parent_failstofinish 0.65744 0.15823 4.155 3.25e-05 ***

#y9_parent_cantconcentrate 0.48581 0.13043 3.725 0.000196 ***

#y9_parent_restless 1.20493 0.12848 9.379 < 2e-16 ***

#y9_parent_demandsattention 0.33640 0.13493 2.493 0.012662 *

#y9_parent_easydistracted 0.55344 0.14162 3.908 9.31e-05 ***

#y9_parent_talktoomuch 0.21992 0.13355 1.647 0.099613 .

#y9_parent_tempertantrums 0.60032 0.16844 3.564 0.000365 ***

#y9_parent_reportsaccappropr -0.40514 0.08241 -4.916 8.82e-07 ***

# Examine accuracy in non-imputed holdout sample

glm_probs <- data.frame(probs = predict(best.logit$BestModel, newdata = holdout,type="response"))

glm_pred <- glm_probs %>% mutate(pred = ifelse(probs>.5, "1", "0"))

glm_pred <- cbind(holdout, glm_pred)

glm_pred %>% count(pred, adhd) %>% spread(adhd, n, fill = 0)

# pred 0 1

#1 0 455 42

#2 1 49 54

#3 <NA> 95 20

#Sensitivity

54/(54+42)*100 # 56.3%

#PPV

54/(49+54)*100 # 52.4%

##### Teacher-report

ry9_teacher_alotmoreactive_structuredplay <-glm(adhd~male+y9_teacher_alotmoreactive_structuredplay, data=expl, family ="binomial")

ry9_teacher_alotmoreactive_unstructuredplay <-glm(adhd~male+y9_teacher_alotmoreactive_unstructuredplay, data=expl, family ="binomial")

ry9_teacher_onlyattentionowninterests <-glm(adhd~male+y9_teacher_onlyattentionowninterests, data=expl, family ="binomial")

ry9_teacher_invitesotherinactivities <-glm(adhd~male+y9_teacher_invitesotherinactivities, data=expl, family ="binomial")

ry9_teacher_poorlanguage <-glm(adhd~male+y9_teacher_poorlanguage, data=expl, family ="binomial")

ry9_teacher_poorsocialstudies <-glm(adhd~male+y9_teacher_poorsocialstudies, data=expl, family ="binomial")

ry9_teacher_poormath <-glm(adhd~male+y9_teacher_poormath, data=expl, family ="binomial")

ry9_teacher_controltemper <-glm(adhd~male+y9_teacher_controltemper, data=expl, family ="binomial")

ry9_teacher_compromiseconflict <-glm(adhd~male+y9_teacher_compromiseconflict, data=expl, family ="binomial")

ry9_teacher_respondadeqpeerpress <-glm(adhd~male+y9_teacher_respondadeqpeerpress, data=expl, family ="binomial")

ry9_teacher_saynicethingsabself <-glm(adhd~male+y9_teacher_saynicethingsabself, data=expl, family ="binomial")

ry9_teacher_usefreetimeappropr <-glm(adhd~male+y9_teacher_usefreetimeappropr, data=expl, family ="binomial")

ry9_teacher_finishintime <-glm(adhd~male+y9_teacher_finishintime, data=expl, family ="binomial")

ry9_teacher_makesfriendseasy <-glm(adhd~male+y9_teacher_makesfriendseasy, data=expl, family ="binomial")

ry9_teacher_responapprteasing <-glm(adhd~male+y9_teacher_responapprteasing, data=expl, family ="binomial")

ry9_teacher_controlstemper <-glm(adhd~male+y9_teacher_controlstemper, data=expl, family ="binomial")

ry9_teacher_receivecritiquewell <-glm(adhd~male+y9_teacher_receivecritiquewell, data=expl, family ="binomial")

ry9_teacher_usetimeapprwaitforhelp <-glm(adhd~male+y9_teacher_usetimeapprwaitforhelp, data=expl, family ="binomial")

ry9_teacher_correctschoolwork <-glm(adhd~male+y9_teacher_correctschoolwork, data=expl, family ="binomial")

ry9_teacher_acceptpeersideas <-glm(adhd~male+y9_teacher_acceptpeersideas, data=expl, family ="binomial")

ry9_teacher_givescompliments <-glm(adhd~male+y9_teacher_givescompliments, data=expl, family ="binomial")

ry9_teacher_followsdirections <-glm(adhd~male+y9_teacher_followsdirections, data=expl, family ="binomial")

ry9_teacher_putawayschoolmaterial <-glm(adhd~male+y9_teacher_putawayschoolmaterial, data=expl, family ="binomial")

ry9_teacher_cooperatespeers <-glm(adhd~male+y9_teacher_cooperatespeers, data=expl, family ="binomial")

ry9_teacher_joingroupspontaneously <-glm(adhd~male+y9_teacher_joingroupspontaneously, data=expl, family ="binomial")

ry9_teacher_respadeqwhenpushed <-glm(adhd~male+y9_teacher_respadeqwhenpushed, data=expl, family ="binomial")

ry9_teacher_ignorepeerdistrinclass <-glm(adhd~male+y9_teacher_ignorepeerdistrinclass, data=expl, family ="binomial")

ry9_teacher_cleandesk <-glm(adhd~male+y9_teacher_cleandesk, data=expl, family ="binomial")

ry9_teacher_attendsinstruct <-glm(adhd~male+y9_teacher_attendsinstruct, data=expl, family ="binomial")

ry9_teacher_transitseasybtwactiv <-glm(adhd~male+y9_teacher_transitseasybtwactiv, data=expl, family ="binomial")

ry9_teacher_getalongdiffpeople <-glm(adhd~male+y9_teacher_getalongdiffpeople, data=expl, family ="binomial")

ry9_teacher_expressownfeelings <-glm(adhd~male+y9_teacher_expressownfeelings, data=expl, family ="binomial")

ry9_teacher_maintainsfriendships <-glm(adhd~male+y9_teacher_maintainsfriendships, data=expl, family ="binomial")

ry9_teacher_respectotherproperties <-glm(adhd~male+y9_teacher_respectotherproperties, data=expl, family ="binomial")

ry9_teacher_sensotherfeelings <-glm(adhd~male+y9_teacher_sensotherfeelings, data=expl, family ="binomial")

ry9_teacher_helpsothers <-glm(adhd~male+y9_teacher_helpsothers, data=expl, family ="binomial")

ry9_teacher_paysattention <-glm(adhd~male+y9_teacher_paysattention, data=expl, family ="binomial")

ry9_teacher_persiststasks <-glm(adhd~male+y9_teacher_persiststasks, data=expl, family ="binomial")

ry9_teacher_worksindependently <-glm(adhd~male+y9_teacher_worksindependently, data=expl, family ="binomial")

ry9_teacher_adaptschangeroutine <-glm(adhd~male+y9_teacher_adaptschangeroutine, data=expl, family ="binomial")

ry9_teacher_organizedbelongings <-glm(adhd~male+y9_teacher_organizedbelongings, data=expl, family ="binomial")

ry9_teacher_eagerlearnnewthings <-glm(adhd~male+y9_teacher_eagerlearnnewthings, data=expl, family ="binomial")

ry9_teacher_followclassroomrules <-glm(adhd~male+y9_teacher_followclassroomrules, data=expl, family ="binomial")

ry9_teacher_figthswothers <-glm(adhd~male+y9_teacher_figthswothers, data=expl, family ="binomial")

ry9_teacher_lowselfesteem <-glm(adhd~male+y9_teacher_lowselfesteem, data=expl, family ="binomial")

ry9_teacher_bulliesothers <-glm(adhd~male+y9_teacher_bulliesothers, data=expl, family ="binomial")

ry9_teacher_appearslonely <-glm(adhd~male+y9_teacher_appearslonely, data=expl, family ="binomial")

ry9_teacher_anxingroupchildren <-glm(adhd~male+y9_teacher_anxingroupchildren, data=expl, family ="binomial")

ry9_teacher_easyembarassed <-glm(adhd~male+y9_teacher_easyembarassed, data=expl, family ="binomial")

ry9_teacher_arguesothers <-glm(adhd~male+y9_teacher_arguesothers, data=expl, family ="binomial")

ry9_teacher_talkbackadults <-glm(adhd~male+y9_teacher_talkbackadults, data=expl, family ="binomial")

ry9_teacher_angryeasily <-glm(adhd~male+y9_teacher_angryeasily, data=expl, family ="binomial")

ry9_teacher_tempertantrums <-glm(adhd~male+y9_teacher_tempertantrums, data=expl, family ="binomial")

ry9_teacher_likesbeingalone <-glm(adhd~male+y9_teacher_likesbeingalone, data=expl, family ="binomial")

ry9_teacher_sad <-glm(adhd~male+y9_teacher_sad, data=expl, family ="binomial")

ry9_teacher_inattentive <-glm(adhd~male+y9_teacher_inattentive, data=expl, family ="binomial")

ry9_teacher_defiant <-glm(adhd~male+y9_teacher_defiant, data=expl, family ="binomial")

ry9_teacher_restless <-glm(adhd~male+y9_teacher_restless, data=expl, family ="binomial")

ry9_teacher_forgetwhatlearned <-glm(adhd~male+y9_teacher_forgetwhatlearned, data=expl, family ="binomial")

ry9_teacher_disturbothers <-glm(adhd~male+y9_teacher_disturbothers, data=expl, family ="binomial")

ry9_teacher_defiesadults <-glm(adhd~male+y9_teacher_defiesadults, data=expl, family ="binomial")

ry9_teacher_alwaysonthego <-glm(adhd~male+y9_teacher_alwaysonthego, data=expl, family ="binomial")

ry9_teacher_poorspelling <-glm(adhd~male+y9_teacher_poorspelling, data=expl, family ="binomial")

ry9_teacher_cannotremainstill <-glm(adhd~male+y9_teacher_cannotremainstill, data=expl, family ="binomial")

ry9_teacher_spiteful <-glm(adhd~male+y9_teacher_spiteful, data=expl, family ="binomial")

ry9_teacher_leavesseat <-glm(adhd~male+y9_teacher_leavesseat, data=expl, family ="binomial")

ry9_teacher_fidgethands <-glm(adhd~male+y9_teacher_fidgethands, data=expl, family ="binomial")

ry9_teacher_poorreading <-glm(adhd~male+y9_teacher_poorreading, data=expl, family ="binomial")

ry9_teacher_shortattentionspan <-glm(adhd~male+y9_teacher_shortattentionspan, data=expl, family ="binomial")

ry9_teacher_arguesadults <-glm(adhd~male+y9_teacher_arguesadults, data=expl, family ="binomial")

ry9_teacher_diffwaitturn <-glm(adhd~male+y9_teacher_diffwaitturn, data=expl, family ="binomial")

ry9_teacher_nointerestschool <-glm(adhd~male+y9_teacher_nointerestschool, data=expl, family ="binomial")

ry9_teacher_distractable <-glm(adhd~male+y9_teacher_distractable, data=expl, family ="binomial")

ry9_teacher_temperoutbursts <-glm(adhd~male+y9_teacher_temperoutbursts, data=expl, family ="binomial")

ry9_teacher_runsclimbs <-glm(adhd~male+y9_teacher_runsclimbs, data=expl, family ="binomial")

ry9_teacher_poorarithmetic <-glm(adhd~male+y9_teacher_poorarithmetic, data=expl, family ="binomial")

ry9_teacher_intrudesothers <-glm(adhd~male+y9_teacher_intrudesothers, data=expl, family ="binomial")

ry9_teacher_diffplayingquietly <-glm(adhd~male+y9_teacher_diffplayingquietly, data=expl, family ="binomial")

ry9_teacher_failstofinish <-glm(adhd~male+y9_teacher_failstofinish, data=expl, family ="binomial")

ry9_teacher_notfollowinstruct <-glm(adhd~male+y9_teacher_notfollowinstruct, data=expl, family ="binomial")

ry9_teacher_excitable <-glm(adhd~male+y9_teacher_excitable, data=expl, family ="binomial")

ry9_teacher_alwaysongo <-glm(adhd~male+y9_teacher_alwaysongo, data=expl, family ="binomial")

ry9_teacher_repeatedgrade <-glm(adhd~male+y9_teacher_repeatedgrade, data=expl, family ="binomial")

summary(ry9_teacher_alotmoreactive_structuredplay)#***

summary(ry9_teacher_alotmoreactive_unstructuredplay)#***

summary(ry9_teacher_onlyattentionowninterests)#***

summary(ry9_teacher_invitesotherinactivities)#***

summary(ry9_teacher_poorlanguage)#***

summary(ry9_teacher_poorsocialstudies)#**

summary(ry9_teacher_poormath)#***

summary(ry9_teacher_controltemper)#***

summary(ry9_teacher_compromiseconflict)#***

summary(ry9_teacher_respondadeqpeerpress)#***

summary(ry9_teacher_saynicethingsabself)#***

summary(ry9_teacher_usefreetimeappropr)#***

summary(ry9_teacher_finishintime)#***

summary(ry9_teacher_makesfriendseasy)#***

summary(ry9_teacher_responapprteasing)#***

summary(ry9_teacher_controlstemper)#***

summary(ry9_teacher_receivecritiquewell)#***

summary(ry9_teacher_usetimeapprwaitforhelp)#***

summary(ry9_teacher_correctschoolwork)#***

summary(ry9_teacher_acceptpeersideas)#***

summary(ry9_teacher_givescompliments)#***

summary(ry9_teacher_followsdirections)#***

summary(ry9_teacher_putawayschoolmaterial)#***

summary(ry9_teacher_cooperatespeers)#***

summary(ry9_teacher_joingroupspontaneously)#***

summary(ry9_teacher_respadeqwhenpushed)#***

summary(ry9_teacher_ignorepeerdistrinclass)#***

summary(ry9_teacher_cleandesk)#***

summary(ry9_teacher_attendsinstruct)#***

summary(ry9_teacher_transitseasybtwactiv)#***

summary(ry9_teacher_getalongdiffpeople)#***

summary(ry9_teacher_expressownfeelings)#***

summary(ry9_teacher_maintainsfriendships)#***

summary(ry9_teacher_respectotherproperties)

summary(ry9_teacher_sensotherfeelings)#*

summary(ry9_teacher_helpsothers)#*

summary(ry9_teacher_paysattention)

summary(ry9_teacher_persiststasks)#*

summary(ry9_teacher_worksindependently)#**

summary(ry9_teacher_adaptschangeroutine)#***

summary(ry9_teacher_organizedbelongings)#***

summary(ry9_teacher_eagerlearnnewthings)

summary(ry9_teacher_followclassroomrules)

summary(ry9_teacher_figthswothers)#***

summary(ry9_teacher_lowselfesteem)#***

summary(ry9_teacher_bulliesothers)#***

summary(ry9_teacher_appearslonely)#***

summary(ry9_teacher_anxingroupchildren)#***

summary(ry9_teacher_easyembarassed)

summary(ry9_teacher_arguesothers)#***

summary(ry9_teacher_talkbackadults)#***

summary(ry9_teacher_angryeasily)#***

summary(ry9_teacher_tempertantrums)#***

summary(ry9_teacher_likesbeingalone)#***

summary(ry9_teacher_sad)#***

summary(ry9_teacher_inattentive)#***

summary(ry9_teacher_defiant)#***

summary(ry9_teacher_restless)#***

summary(ry9_teacher_forgetwhatlearned)#***

summary(ry9_teacher_disturbothers)#***

summary(ry9_teacher_defiesadults)#***

summary(ry9_teacher_alwaysonthego)#***

summary(ry9_teacher_poorspelling)#***

summary(ry9_teacher_cannotremainstill)#***

summary(ry9_teacher_spiteful)#***

summary(ry9_teacher_leavesseat)#***

summary(ry9_teacher_fidgethands)#***

summary(ry9_teacher_poorreading)#***

summary(ry9_teacher_shortattentionspan)#***

summary(ry9_teacher_arguesadults)#***

summary(ry9_teacher_diffwaitturn)#***

summary(ry9_teacher_nointerestschool)#***

summary(ry9_teacher_distractable)#***

summary(ry9_teacher_temperoutbursts)#***

summary(ry9_teacher_runsclimbs)#***

summary(ry9_teacher_poorarithmetic)#***

summary(ry9_teacher_intrudesothers)#***

summary(ry9_teacher_diffplayingquietly)#***

summary(ry9_teacher_failstofinish)#***

summary(ry9_teacher_notfollowinstruct)#***

summary(ry9_teacher_excitable)#***

summary(ry9_teacher_alwaysongo)#***

summary(ry9_teacher_repeatedgrade)#***

CrossTable(expl$adhd, expl$y9_teacher_alotmoreactive_structuredplay)

CrossTable(expl$adhd, expl$y9_teacher_alotmoreactive_unstructuredplay)

CrossTable(expl$adhd, expl$y9_teacher_onlyattentionowninterests)

CrossTable(expl$adhd, expl$y9_teacher_invitesotherinactivities)

CrossTable(expl$adhd, expl$y9_teacher_poorlanguage)

CrossTable(expl$adhd, expl$y9_teacher_poorsocialstudies)

CrossTable(expl$adhd, expl$y9_teacher_poormath)

CrossTable(expl$adhd, expl$y9_teacher_controltemper)

CrossTable(expl$adhd, expl$y9_teacher_compromiseconflict)

CrossTable(expl$adhd, expl$y9_teacher_respondadeqpeerpress)

CrossTable(expl$adhd, expl$y9_teacher_saynicethingsabself)

CrossTable(expl$adhd, expl$y9_teacher_usefreetimeappropr)

CrossTable(expl$adhd, expl$y9_teacher_finishintime)

CrossTable(expl$adhd, expl$y9_teacher_makesfriendseasy)

CrossTable(expl$adhd, expl$y9_teacher_responapprteasing)

CrossTable(expl$adhd, expl$y9_teacher_receivecritiquewell)

CrossTable(expl$adhd, expl$y9_teacher_usetimeapprwaitforhelp)

CrossTable(expl$adhd, expl$y9_teacher_correctschoolwork)

CrossTable(expl$adhd, expl$y9_teacher_acceptpeersideas)

CrossTable(expl$adhd, expl$y9_teacher_givescompliments)

CrossTable(expl$adhd, expl$y9_teacher_followsdirections)

CrossTable(expl$adhd, expl$y9_teacher_putawayschoolmaterial)

CrossTable(expl$adhd, expl$y9_teacher_cooperatespeers)

CrossTable(expl$adhd, expl$y9_teacher_joingroupspontaneously)

CrossTable(expl$adhd, expl$y9_teacher_respadeqwhenpushed)

CrossTable(expl$adhd, expl$y9_teacher_ignorepeerdistrinclass)

CrossTable(expl$adhd, expl$y9_teacher_cleandesk)

CrossTable(expl$adhd, expl$y9_teacher_attendsinstruct)

CrossTable(expl$adhd, expl$y9_teacher_transitseasybtwactiv)

CrossTable(expl$adhd, expl$y9_teacher_getalongdiffpeople)

CrossTable(expl$adhd, expl$y9_teacher_expressownfeelings)

CrossTable(expl$adhd, expl$y9_teacher_maintainsfriendships)

CrossTable(expl$adhd, expl$y9_teacher_worksindependently)

CrossTable(expl$adhd, expl$y9_teacher_adaptschangeroutine)

CrossTable(expl$adhd, expl$y9_teacher_organizedbelongings)

CrossTable(expl$adhd, expl$y9_teacher_figthswothers)

CrossTable(expl$adhd, expl$y9_teacher_lowselfesteem)

CrossTable(expl$adhd, expl$y9_teacher_bulliesothers)

CrossTable(expl$adhd, expl$y9_teacher_appearslonely)

CrossTable(expl$adhd, expl$y9_teacher_anxingroupchildren)

CrossTable(expl$adhd, expl$y9_teacher_arguesothers)

CrossTable(expl$adhd, expl$y9_teacher_talkbackadults)

CrossTable(expl$adhd, expl$y9_teacher_angryeasily)

CrossTable(expl$adhd, expl$y9_teacher_tempertantrums)

CrossTable(expl$adhd, expl$y9_teacher_likesbeingalone)

CrossTable(expl$adhd, expl$y9_teacher_sad)

CrossTable(expl$adhd, expl$y9_teacher_inattentive)

CrossTable(expl$adhd, expl$y9_teacher_defiant)

CrossTable(expl$adhd, expl$y9_teacher_restless)

CrossTable(expl$adhd, expl$y9_teacher_forgetwhatlearned)

CrossTable(expl$adhd, expl$y9_teacher_disturbothers)

CrossTable(expl$adhd, expl$y9_teacher_defiesadults)

CrossTable(expl$adhd, expl$y9_teacher_alwaysonthego)

CrossTable(expl$adhd, expl$y9_teacher_poorspelling)

CrossTable(expl$adhd, expl$y9_teacher_cannotremainstill)

CrossTable(expl$adhd, expl$y9_teacher_spiteful)

CrossTable(expl$adhd, expl$y9_teacher_leavesseat)

CrossTable(expl$adhd, expl$y9_teacher_fidgethands)

CrossTable(expl$adhd, expl$y9_teacher_poorreading)

CrossTable(expl$adhd, expl$y9_teacher_shortattentionspan)

CrossTable(expl$adhd, expl$y9_teacher_arguesadults)

CrossTable(expl$adhd, expl$y9_teacher_diffwaitturn)

CrossTable(expl$adhd, expl$y9_teacher_nointerestschool)

CrossTable(expl$adhd, expl$y9_teacher_distractable)

CrossTable(expl$adhd, expl$y9_teacher_temperoutbursts)

CrossTable(expl$adhd, expl$y9_teacher_runsclimbs)

CrossTable(expl$adhd, expl$y9_teacher_poorarithmetic)

CrossTable(expl$adhd, expl$y9_teacher_intrudesothers)

CrossTable(expl$adhd, expl$y9_teacher_diffplayingquietly)

CrossTable(expl$adhd, expl$y9_teacher_failstofinish)

CrossTable(expl$adhd, expl$y9_teacher_notfollowinstruct)

CrossTable(expl$adhd, expl$y9_teacher_excitable)

CrossTable(expl$adhd, expl$y9_teacher_alwaysongo)

CrossTable(expl$adhd, expl$y9_teacher_repeatedgrade)

###Examine accuracy

data.rose <- ROSE(adhd ~ y9_teacher_alotmoreactive_structuredplay+

y9_teacher_alotmoreactive_unstructuredplay+

y9_teacher_onlyattentionowninterests+

y9_teacher_invitesotherinactivities+

y9_teacher_poorlanguage+

y9_teacher_poorsocialstudies+

y9_teacher_poormath+

y9_teacher_controltemper+

y9_teacher_compromiseconflict+

y9_teacher_respondadeqpeerpress+

y9_teacher_saynicethingsabself+

y9_teacher_usefreetimeappropr+

y9_teacher_finishintime+

y9_teacher_makesfriendseasy+

y9_teacher_responapprteasing,

data = expl, seed = 1)$data

lowbwt.bglm <- data.rose[, c("y9_teacher_alotmoreactive_structuredplay",

"y9_teacher_alotmoreactive_unstructuredplay",

"y9_teacher_onlyattentionowninterests",

"y9_teacher_invitesotherinactivities",

"y9_teacher_poorlanguage",

"y9_teacher_poorsocialstudies",

"y9_teacher_poormath",

"y9_teacher_controltemper",

"y9_teacher_compromiseconflict",

"y9_teacher_respondadeqpeerpress",

"y9_teacher_saynicethingsabself",

"y9_teacher_usefreetimeappropr",

"y9_teacher_finishintime",

"y9_teacher_makesfriendseasy",

"y9_teacher_responapprteasing","adhd")]

names(lowbwt.bglm)[names(lowbwt.bglm) == "adhd"] <- "y"

lowbwt.bglm <- as.data.frame(lowbwt.bglm)

best.logit <- bestglm(lowbwt.bglm,

IC = "AIC",

family=binomial,

method = "exhaustive")

summary(best.logit$BestModel)

#y9_teacher_alotmoreactive_unstructuredplay 0.4518 0.1697 2.663 0.00775 **

#y9_teacher_invitesotherinactivities 0.2505 0.1108 2.260 0.02384 *

#y9_teacher_controltemper -0.2690 0.1177 -2.286 0.02223 *

#y9_teacher_compromiseconflict 0.1709 0.1072 1.593 0.11107

#y9_teacher_respondadeqpeerpress -0.2956 0.1203 -2.457 0.01401 *

#y9_teacher_saynicethingsabself -0.3031 0.1035 -2.928 0.00342 **

#y9_teacher_usefreetimeappropr -0.4575 0.1081 -4.233 2.30e-05 ***

#y9_teacher_finishintime -0.3461 0.1078 -3.210 0.00133 **

#y9_teacher_makesfriendseasy -0.5089 0.1154 -4.409 1.04e-05 ***

#y9_teacher_responapprteasing -0.4790 0.1121 -4.274 1.92e-05 ***

data.rose <- ROSE(adhd ~ y9_teacher_receivecritiquewell+

y9_teacher_usetimeapprwaitforhelp+

y9_teacher_correctschoolwork+

y9_teacher_acceptpeersideas+

y9_teacher_givescompliments+

y9_teacher_followsdirections+

y9_teacher_putawayschoolmaterial+

y9_teacher_cooperatespeers+

y9_teacher_joingroupspontaneously+

y9_teacher_respadeqwhenpushed+

y9_teacher_ignorepeerdistrinclass+

y9_teacher_cleandesk+

y9_teacher_attendsinstruct+

y9_teacher_transitseasybtwactiv+

y9_teacher_getalongdiffpeople,

data = expl, seed = 1)$data

lowbwt.bglm <- data.rose[, c("y9_teacher_receivecritiquewell",

"y9_teacher_usetimeapprwaitforhelp",

"y9_teacher_correctschoolwork",

"y9_teacher_acceptpeersideas",

"y9_teacher_givescompliments",

"y9_teacher_followsdirections",

"y9_teacher_putawayschoolmaterial",

"y9_teacher_cooperatespeers",

"y9_teacher_joingroupspontaneously",

"y9_teacher_respadeqwhenpushed",

"y9_teacher_ignorepeerdistrinclass",

"y9_teacher_cleandesk",

"y9_teacher_attendsinstruct",

"y9_teacher_transitseasybtwactiv",

"y9_teacher_getalongdiffpeople", "adhd")]

names(lowbwt.bglm)[names(lowbwt.bglm) == "adhd"] <- "y"

lowbwt.bglm <- as.data.frame(lowbwt.bglm)

best.logit <- bestglm(lowbwt.bglm,

IC = "AIC",

family=binomial,

method = "exhaustive")

summary(best.logit$BestModel)

#y9_teacher_receivecritiquewell -0.3357 0.1058 -3.171 0.001517 **

#y9_teacher_usetimeapprwaitforhelp -0.2361 0.1115 -2.117 0.034259 *

#y9_teacher_followsdirections -0.2890 0.1152 -2.509 0.012096 *

#y9_teacher_respadeqwhenpushed -0.3072 0.1082 -2.839 0.004525 **

#y9_teacher_ignorepeerdistrinclass -0.2575 0.1216 -2.116 0.034304 *

#y9_teacher_cleandesk -0.4119 0.1019 -4.043 5.27e-05 ***

#y9_teacher_transitseasybtwactiv -0.4336 0.1145 -3.787 0.000152 ***

#y9_teacher_getalongdiffpeople -0.2355 0.1109 -2.124 0.033692 *

data.rose <- ROSE(adhd ~ y9_teacher_expressownfeelings+

y9_teacher_maintainsfriendships+

y9_teacher_worksindependently+

y9_teacher_adaptschangeroutine+

y9_teacher_organizedbelongings+

y9_teacher_figthswothers+

y9_teacher_lowselfesteem+

y9_teacher_bulliesothers+

y9_teacher_appearslonely+

y9_teacher_anxingroupchildren+

y9_teacher_arguesothers+

y9_teacher_talkbackadults+

y9_teacher_angryeasily+

y9_teacher_tempertantrums+

y9_teacher_likesbeingalone,

data = expl, seed = 1)$data

lowbwt.bglm <- data.rose[, c("y9_teacher_expressownfeelings",

"y9_teacher_maintainsfriendships",

"y9_teacher_worksindependently",

"y9_teacher_adaptschangeroutine",

"y9_teacher_organizedbelongings",

"y9_teacher_figthswothers",

"y9_teacher_lowselfesteem",

"y9_teacher_bulliesothers",

"y9_teacher_appearslonely",

"y9_teacher_anxingroupchildren",

"y9_teacher_arguesothers",

"y9_teacher_talkbackadults",

"y9_teacher_angryeasily",

"y9_teacher_tempertantrums",

"y9_teacher_likesbeingalone", "adhd")]

names(lowbwt.bglm)[names(lowbwt.bglm) == "adhd"] <- "y"

lowbwt.bglm <- as.data.frame(lowbwt.bglm)

best.logit <- bestglm(lowbwt.bglm,

IC = "AIC",

family=binomial,

method = "exhaustive")

summary(best.logit$BestModel)

#y9_teacher_expressownfeelings -0.5029 0.1489 -3.376 0.000734 ***

#y9_teacher_lowselfesteem 0.3191 0.1769 1.804 0.071271 .

#y9_teacher_anxingroupchildren 0.6942 0.1536 4.519 6.23e-06 ***

#y9_teacher_arguesothers 0.3500 0.2050 1.707 0.087745 .

#y9_teacher_talkbackadults 0.3244 0.1552 2.089 0.036685 *

#y9_teacher_angryeasily 0.5692 0.1639 3.473 0.000515 ***

data.rose <- ROSE(adhd ~ y9_teacher_sad+

y9_teacher_inattentive+

y9_teacher_defiant+

y9_teacher_restless+

y9_teacher_forgetwhatlearned+

y9_teacher_disturbothers+

y9_teacher_defiesadults+

y9_teacher_alwaysonthego+

y9_teacher_poorspelling+

y9_teacher_cannotremainstill+

y9_teacher_spiteful+

y9_teacher_leavesseat+

y9_teacher_fidgethands+

y9_teacher_poorreading,

data = expl, seed = 1)$data

lowbwt.bglm <- data.rose[, c("y9_teacher_sad",

"y9_teacher_inattentive",

"y9_teacher_defiant",

"y9_teacher_restless",

"y9_teacher_forgetwhatlearned",

"y9_teacher_disturbothers",

"y9_teacher_defiesadults",

"y9_teacher_alwaysonthego",

"y9_teacher_poorspelling",

"y9_teacher_cannotremainstill",

"y9_teacher_spiteful",

"y9_teacher_leavesseat",

"y9_teacher_fidgethands",

"y9_teacher_poorreading", "adhd")]

names(lowbwt.bglm)[names(lowbwt.bglm) == "adhd"] <- "y"

lowbwt.bglm <- as.data.frame(lowbwt.bglm)

best.logit <- bestglm(lowbwt.bglm,

IC = "AIC",

family=binomial,

method = "exhaustive")

summary(best.logit$BestModel)

#y9_teacher_sad 0.47799 0.09886 4.835 1.33e-06 ***

#y9_teacher_inattentive 0.49535 0.10954 4.522 6.13e-06 ***

#y9_teacher_defiant 0.26295 0.14466 1.818 0.06910 .

#y9_teacher_restless 0.29752 0.13603 2.187 0.02873 *

#y9_teacher_forgetwhatlearned 0.16980 0.11733 1.447 0.14783

#y9_teacher_disturbothers 0.24124 0.12565 1.920 0.05486 .

#y9_teacher_alwaysonthego 0.44338 0.14550 3.047 0.00231 **

#y9_teacher_cannotremainstill 0.33023 0.15340 2.153 0.03134 *

#y9_teacher_leavesseat 0.37489 0.13553 2.766 0.00567 **

#y9_teacher_fidgethands 0.25293 0.13735 1.842 0.06554 .

#y9_teacher_poorreading 0.31413 0.11105 2.829 0.00467 **

data.rose <- ROSE(adhd ~ y9_teacher_shortattentionspan+

y9_teacher_arguesadults+

y9_teacher_diffwaitturn+

y9_teacher_nointerestschool+

y9_teacher_distractable+

y9_teacher_temperoutbursts+

y9_teacher_runsclimbs+

y9_teacher_poorarithmetic+

y9_teacher_intrudesothers+

y9_teacher_diffplayingquietly+

y9_teacher_failstofinish+

y9_teacher_notfollowinstruct+

y9_teacher_excitable+

y9_teacher_alwaysongo+

y9_teacher_repeatedgrade,

data = expl, seed = 1)$data

lowbwt.bglm <- data.rose[, c("y9_teacher_shortattentionspan",

"y9_teacher_arguesadults",

"y9_teacher_diffwaitturn",

"y9_teacher_nointerestschool",

"y9_teacher_distractable",

"y9_teacher_temperoutbursts",

"y9_teacher_runsclimbs",

"y9_teacher_poorarithmetic",

"y9_teacher_intrudesothers",

"y9_teacher_diffplayingquietly",

"y9_teacher_failstofinish",

"y9_teacher_notfollowinstruct",

"y9_teacher_excitable",

"y9_teacher_alwaysongo",

"y9_teacher_repeatedgrade", "adhd")]

names(lowbwt.bglm)[names(lowbwt.bglm) == "adhd"] <- "y"

lowbwt.bglm <- as.data.frame(lowbwt.bglm)

best.logit <- bestglm(lowbwt.bglm,

IC = "AIC",

family=binomial,

method = "exhaustive")

summary(best.logit$BestModel)

#y9_teacher_shortattentionspan 0.46919 0.12723 3.688 0.000226 ***

#y9_teacher_diffwaitturn 0.45336 0.14123 3.210 0.001327 **

#y9_teacher_nointerestschool -0.28664 0.12728 -2.252 0.024317 *

#y9_teacher_distractable 0.31048 0.12223 2.540 0.011078 *

#y9_teacher_temperoutbursts 0.73145 0.15026 4.868 1.13e-06 ***

#y9_teacher_poorarithmetic 0.23857 0.10888 2.191 0.028443 *

#y9_teacher_intrudesothers 0.25889 0.12883 2.010 0.044472 *

#y9_teacher_excitable 0.53745 0.13945 3.854 0.000116 ***

#y9_teacher_alwaysongo 0.21280 0.14861 1.432 0.152176

#y9_teacher_repeatedgrade 0.49085 0.21102 2.326 0.020012 *

#### Do even more subselection

data.rose <- ROSE(adhd ~ y9_teacher_alotmoreactive_unstructuredplay+

y9_teacher_saynicethingsabself+

y9_teacher_usefreetimeappropr+

y9_teacher_finishintime+

y9_teacher_makesfriendseasy+

y9_teacher_responapprteasing+

y9_teacher_receivecritiquewell+

y9_teacher_respadeqwhenpushed+

y9_teacher_cleandesk+

y9_teacher_transitseasybtwactiv+

y9_teacher_expressownfeelings,

data = expl, seed = 1)$data

lowbwt.bglm <- data.rose[, c("y9_teacher_alotmoreactive_unstructuredplay",

"y9_teacher_saynicethingsabself",

"y9_teacher_usefreetimeappropr",

"y9_teacher_finishintime",

"y9_teacher_makesfriendseasy",

"y9_teacher_responapprteasing",

"y9_teacher_receivecritiquewell",

"y9_teacher_respadeqwhenpushed",

"y9_teacher_cleandesk",

"y9_teacher_transitseasybtwactiv",

"y9_teacher_expressownfeelings", "adhd")]

names(lowbwt.bglm)[names(lowbwt.bglm) == "adhd"] <- "y"

lowbwt.bglm <- as.data.frame(lowbwt.bglm)

best.logit <- bestglm(lowbwt.bglm,

IC = "AIC",

family=binomial,

method = "exhaustive")

summary(best.logit$BestModel)

#y9_teacher_finishintime -0.2918 0.1153 -2.530 0.01142 *

#y9_teacher_makesfriendseasy -0.4497 0.1091 -4.123 3.74e-05 ***

#y9_teacher_responapprteasing -0.3504 0.1236 -2.836 0.00457 **

#y9_teacher_receivecritiquewell -0.2067 0.1157 -1.786 0.07412 .

#y9_teacher_respadeqwhenpushed -0.1906 0.1169 -1.630 0.10305

#y9_teacher_cleandesk -0.5218 0.1046 -4.990 6.03e-07 ***

#y9_teacher_transitseasybtwactiv -0.5352 0.1168 -4.584 4.57e-06 ***

data.rose <- ROSE(adhd ~ y9_teacher_anxingroupchildren+

y9_teacher_angryeasily+

y9_teacher_sad+

y9_teacher_inattentive+

y9_teacher_alwaysonthego+

y9_teacher_leavesseat+

y9_teacher_poorreading+

y9_teacher_shortattentionspan+

y9_teacher_diffwaitturn+

y9_teacher_temperoutbursts+

y9_teacher_excitable,

data = expl, seed = 1)$data

lowbwt.bglm <- data.rose[, c("y9_teacher_anxingroupchildren",

"y9_teacher_angryeasily",

"y9_teacher_sad",

"y9_teacher_inattentive",

"y9_teacher_alwaysonthego",

"y9_teacher_leavesseat",

"y9_teacher_poorreading",

"y9_teacher_shortattentionspan",

"y9_teacher_diffwaitturn",

"y9_teacher_temperoutbursts",

"y9_teacher_excitable", "adhd")]

names(lowbwt.bglm)[names(lowbwt.bglm) == "adhd"] <- "y"

lowbwt.bglm <- as.data.frame(lowbwt.bglm)

best.logit <- bestglm(lowbwt.bglm,

IC = "AIC",

family=binomial,

method = "exhaustive")

summary(best.logit$BestModel)

#y9_teacher_anxingroupchildren 0.73200 0.11154 6.563 5.28e-11 ***

#y9_teacher_angryeasily 0.51511 0.10653 4.835 1.33e-06 ***

#y9_teacher_inattentive 0.36259 0.12469 2.908 0.003638 **

#y9_teacher_alwaysonthego 0.33739 0.15893 2.123 0.033768 *

#y9_teacher_leavesseat 0.58480 0.15385 3.801 0.000144 ***

#y9_teacher_poorreading 0.21745 0.11410 1.906 0.056670 .

#y9_teacher_shortattentionspan 0.41199 0.13494 3.053 0.002264 **

#y9_teacher_temperoutbursts 0.65587 0.19804 3.312 0.000927 ***

#y9_teacher_excitable 0.50867 0.15205 3.346 0.000821 ***

##### Final model

data.rose <- ROSE(adhd ~ y9_teacher_makesfriendseasy+

y9_teacher_responapprteasing+

y9_teacher_cleandesk+

y9_teacher_transitseasybtwactiv+

y9_teacher_anxingroupchildren+

y9_teacher_angryeasily+

y9_teacher_inattentive+

y9_teacher_leavesseat+

y9_teacher_poorreading+

y9_teacher_temperoutbursts+

y9_teacher_excitable,

data = expl, seed = 1)$data

lowbwt.bglm <- data.rose[, c("y9_teacher_makesfriendseasy",

"y9_teacher_responapprteasing",

"y9_teacher_cleandesk",

"y9_teacher_transitseasybtwactiv",

"y9_teacher_anxingroupchildren",

"y9_teacher_angryeasily",

"y9_teacher_inattentive",

"y9_teacher_leavesseat",

"y9_teacher_poorreading",

"y9_teacher_temperoutbursts",

"y9_teacher_excitable", "adhd")]

names(lowbwt.bglm)[names(lowbwt.bglm) == "adhd"] <- "y"

lowbwt.bglm <- as.data.frame(lowbwt.bglm)

best.logit <- bestglm(lowbwt.bglm,

IC = "AIC",

family=binomial,

method = "exhaustive")

summary(best.logit$BestModel)

#y9_teacher_makesfriendseasy -0.4351 0.1158 -3.755 0.000173 ***

#y9_teacher_cleandesk -0.4608 0.1150 -4.008 6.12e-05 ***

#y9_teacher_transitseasybtwactiv -0.3016 0.1178 -2.561 0.010444 *

#y9_teacher_anxingroupchildren 0.6749 0.1161 5.812 6.19e-09 ***

#y9_teacher_angryeasily 0.3851 0.1088 3.539 0.000402 ***

#y9_teacher_inattentive 0.5971 0.1158 5.155 2.54e-07 ***

#y9_teacher_leavesseat 0.7986 0.1474 5.419 5.99e-08 ***

#y9_teacher_temperoutbursts 0.3324 0.1973 1.685 0.092072 .

#y9_teacher_excitable 0.2569 0.1411 1.820 0.068722 .

View(best.logit$BestModel)

# Examine accuracy in non-imputed holdout sample

glm_probs <- data.frame(probs = predict(best.logit$BestModel, newdata = holdout,type="response"))

glm_pred <- glm_probs %>% mutate(pred = ifelse(probs>.5, "1", "0"))

glm_pred <- cbind(holdout, glm_pred)

glm_pred %>% count(pred, adhd) %>% spread(adhd, n, fill = 0)

# pred 0 1

#1 0 246 20

#2 1 85 39

#3 <NA> 268 57

#Sensitivity

39/(39+20)*100 # 66.1%

#PPV

39/(85+39)*100 # 31.45%

#### Self-reported factors

ry9_kid_extraschoolhelpmorethan2hours <- glm(adhd~male+y9_kid_extraschoolhelpmorethan2hours, data=expl, family="binomial")

ry9_kid_notfeelclosepeopleatschool <- glm(adhd~male+y9_kid_notfeelclosepeopleatschool, data=expl, family="binomial")

ry9_kid_kidstakemymoneyinschool <- glm(adhd~male+y9_kid_kidstakemymoneyinschool, data=expl, family="binomial")

ry9_kid_neverstaytaskuntilsolved <- glm(adhd~male+y9_kid_neverstaytaskuntilsolved, data=expl, family="binomial")

ry9_kid_neverwanttosolvehardtask <- glm(adhd~male+y9_kid_neverwanttosolvehardtask, data=expl, family="binomial")

ry9_kid_angrywtroublelearning <- glm(adhd~male+y9_kid_angrywtroublelearning, data=expl, family="binomial")

ry9_kid_neverhangfriends <- glm(adhd~male+y9_kid_neverhangfriends, data=expl, family="binomial")

ry9_kid_neverhelphome <- glm(adhd~male+y9_kid_neverhelphome, data=expl, family="binomial")

ry9_kid_sptimefamless30min <- glm(adhd~male+y9_kid_sptimefamless30min, data=expl, family="binomial")

ry9_kid_gamingmorethan4hours <- glm(adhd~male+y9_kid_gamingmorethan4hours, data=expl, family="binomial")

ry9_kid_tvmorethan4hours <- glm(adhd~male+y9_kid_tvmorethan4hours, data=expl, family="binomial")

ry9_kid_notfeelpartofschool <- glm(adhd~male+y9_kid_notfeelpartofschool, data=expl, family="binomial")

ry9_kid_nohappyatschool <- glm(adhd~male+y9_kid_nohappyatschool, data=expl, family="binomial")

ry9_kid_notsafeatschool <- glm(adhd~male+y9_kid_notsafeatschool, data=expl, family="binomial")

ry9_kid_pickedoninschool <- glm(adhd~male+y9_kid_pickedoninschool, data=expl, family="binomial")

ry9_kid_beateninschool <- glm(adhd~male+y9_kid_beateninschool, data=expl, family="binomial")

ry9_kid_leftoutinschool <- glm(adhd~male+y9_kid_leftoutinschool, data=expl, family="binomial")

ry9_kid_damagedproperty <- glm(adhd~male+y9_kid_damagedproperty, data=expl, family="binomial")

ry9_kid_stolensmthng <- glm(adhd~male+y9_kid_stolensmthng, data=expl, family="binomial")

ry9_kid_takenmoneyhome <- glm(adhd~male+y9_kid_takenmoneyhome, data=expl, family="binomial")

ry9_kid_cheatedschool <- glm(adhd~male+y9_kid_cheatedschool, data=expl, family="binomial")

ry9_kid_fistfight <- glm(adhd~male+y9_kid_fistfight, data=expl, family="binomial")

ry9_kid_hurtanimalpurpose <- glm(adhd~male+y9_kid_hurtanimalpurpose, data=expl, family="binomial")

ry9_kid_enteredotherprpoerty <- glm(adhd~male+y9_kid_enteredotherprpoerty, data=expl, family="binomial")

ry9_kid_runawayhome <- glm(adhd~male+y9_kid_runawayhome, data=expl, family="binomial")

ry9_kid_skippedschool <- glm(adhd~male+y9_kid_skippedschool, data=expl, family="binomial")

ry9_kid_drinking <- glm(adhd~male+y9_kid_drinking, data=expl, family="binomial")

ry9_kid_weed <- glm(adhd~male+y9_kid_weed, data=expl, family="binomial")

ry9_kid_cigarettes <- glm(adhd~male+y9_kid_cigarettes, data=expl, family="binomial")

ry9_kid_suspendedschool <- glm(adhd~male+y9_kid_suspendedschool, data=expl, family="binomial")

ry9_kid_grafitti <- glm(adhd~male+y9_kid_grafitti, data=expl, family="binomial")

ry9_kid_setfire <- glm(adhd~male+y9_kid_setfire, data=expl, family="binomial")

ry9_kid_freeriding <- glm(adhd~male+y9_kid_freeriding, data=expl, family="binomial")

ry9_kid_rockatpeopleorcars <- glm(adhd~male+y9_kid_rockatpeopleorcars, data=expl, family="binomial")

ry9_kid_neverorderly <- glm(adhd~male+y9_kid_neverorderly, data=expl, family="binomial")

ry9_kid_notdobest <- glm(adhd~male+y9_kid_notdobest, data=expl, family="binomial")

ry9_kid_notfollowthrough <- glm(adhd~male+y9_kid_notfollowthrough, data=expl, family="binomial")

ry9_kid_arguewothers <- glm(adhd~male+y9_kid_arguewothers, data=expl, family="binomial")

ry9_kid_worrytests <- glm(adhd~male+y9_kid_worrytests, data=expl, family="binomial")

ry9_kid_hardpayattention <- glm(adhd~male+y9_kid_hardpayattention, data=expl, family="binomial")

ry9_kid_feellonely <- glm(adhd~male+y9_kid_feellonely, data=expl, family="binomial")

ry9_kid_easilydistracted <- glm(adhd~male+y9_kid_easilydistracted, data=expl, family="binomial")

ry9_kid_sad <- glm(adhd~male+y9_kid_sad, data=expl, family="binomial")

ry9_kid_hardfinishschoolwork <- glm(adhd~male+y9_kid_hardfinishschoolwork, data=expl, family="binomial")

ry9_kid_worrydoingwellschool <- glm(adhd~male+y9_kid_worrydoingwellschool, data=expl, family="binomial")

ry9_kid_worryfinishwork <- glm(adhd~male+y9_kid_worryfinishwork, data=expl, family="binomial")

ry9_kid_worrynoonetoplay <- glm(adhd~male+y9_kid_worrynoonetoplay, data=expl, family="binomial")

ry9_kid_smahemistakesschool <- glm(adhd~male+y9_kid_smahemistakesschool, data=expl, family="binomial")

ry9_kid_distrubingothers <- glm(adhd~male+y9_kid_distrubingothers, data=expl, family="binomial")

ry9_kid_fightingothers <- glm(adhd~male+y9_kid_fightingothers, data=expl, family="binomial")

ry9_kid_poororfairhealth <- glm(adhd~male+y9_kid_poororfairhealth, data=expl, family="binomial")

ry9_kid_seldomseatbelt <- glm(adhd~male+y9_kid_seldomseatbelt, data=expl, family="binomial")

summary(ry9_kid_extraschoolhelpmorethan2hours)

summary(ry9_kid_notfeelclosepeopleatschool)#**

summary(ry9_kid_kidstakemymoneyinschool)#*

summary(ry9_kid_neverstaytaskuntilsolved)

summary(ry9_kid_neverwanttosolvehardtask)#***

summary(ry9_kid_angrywtroublelearning)#***

summary(ry9_kid_neverhangfriends)

summary(ry9_kid_neverhelphome)#***

summary(ry9_kid_sptimefamless30min)

summary(ry9_kid_gamingmorethan4hours)

summary(ry9_kid_tvmorethan4hours)

summary(ry9_kid_notfeelpartofschool)

summary(ry9_kid_nohappyatschool)#*

summary(ry9_kid_notsafeatschool)

summary(ry9_kid_pickedoninschool)#***

summary(ry9_kid_beateninschool)#***

summary(ry9_kid_leftoutinschool)#***

summary(ry9_kid_damagedproperty)#**

summary(ry9_kid_stolensmthng)#***

summary(ry9_kid_takenmoneyhome)#**

summary(ry9_kid_cheatedschool)#*

summary(ry9_kid_fistfight)#***

summary(ry9_kid_hurtanimalpurpose)

summary(ry9_kid_enteredotherprpoerty)

summary(ry9_kid_runawayhome)#***

summary(ry9_kid_skippedschool)

summary(ry9_kid_drinking)

summary(ry9_kid_weed)

summary(ry9_kid_cigarettes)#*

summary(ry9_kid_suspendedschool)#***

summary(ry9_kid_grafitti)#***

summary(ry9_kid_setfire)#***

summary(ry9_kid_freeriding)

summary(ry9_kid_rockatpeopleorcars)#**

summary(ry9_kid_neverorderly)#**

summary(ry9_kid_notdobest)#***

summary(ry9_kid_notfollowthrough)#***

summary(ry9_kid_arguewothers)#***

summary(ry9_kid_worrytests)

summary(ry9_kid_hardpayattention)#***

summary(ry9_kid_feellonely)#***

summary(ry9_kid_easilydistracted)#***

summary(ry9_kid_sad)#***

summary(ry9_kid_hardfinishschoolwork)#***

summary(ry9_kid_worrydoingwellschool)

summary(ry9_kid_worryfinishwork)

summary(ry9_kid_worrynoonetoplay)#***

summary(ry9_kid_smahemistakesschool)#*

summary(ry9_kid_distrubingothers)#***

summary(ry9_kid_fightingothers)#***

summary(ry9_kid_poororfairhealth)

summary(ry9_kid_seldomseatbelt)#**

CrossTable(expl$adhd, expl$y9_kid_notfeelclosepeopleatschool)

CrossTable(expl$adhd, expl$y9_kid_neverwanttosolvehardtask)

CrossTable(expl$adhd, expl$y9_kid_angrywtroublelearning)

CrossTable(expl$adhd, expl$y9_kid_neverhelphome)

CrossTable(expl$adhd, expl$y9_kid_pickedoninschool)

CrossTable(expl$adhd, expl$y9_kid_beateninschool)

CrossTable(expl$adhd, expl$y9_kid_leftoutinschool)

CrossTable(expl$adhd, expl$y9_kid_damagedproperty)

CrossTable(expl$adhd, expl$y9_kid_stolensmthng)

CrossTable(expl$adhd, expl$y9_kid_fistfight)

CrossTable(expl$adhd, expl$y9_kid_runawayhome)

CrossTable(expl$adhd, expl$y9_kid_suspendedschool)

CrossTable(expl$adhd, expl$y9_kid_grafitti)

CrossTable(expl$adhd, expl$y9_kid_setfire)

CrossTable(expl$adhd, expl$y9_kid_rockatpeopleorcars)

CrossTable(expl$adhd, expl$y9_kid_neverorderly)

CrossTable(expl$adhd, expl$y9_kid_notdobest)

CrossTable(expl$adhd, expl$y9_kid_notfollowthrough)

CrossTable(expl$adhd, expl$y9_kid_arguewothers)

CrossTable(expl$adhd, expl$y9_kid_hardpayattention)

CrossTable(expl$adhd, expl$y9_kid_feellonely)

CrossTable(expl$adhd, expl$y9_kid_easilydistracted)

CrossTable(expl$adhd, expl$y9_kid_sad)

CrossTable(expl$adhd, expl$y9_kid_hardfinishschoolwork)

CrossTable(expl$adhd, expl$y9_kid_worrynoonetoplay)

CrossTable(expl$adhd, expl$y9_kid_distrubingothers)

CrossTable(expl$adhd, expl$y9_kid_fightingothers)

CrossTable(expl$adhd, expl$y9_kid_seldomseatbelt)

## Explore accuracy

data.rose <- ROSE(adhd ~ y9_kid_notfeelclosepeopleatschool+

y9_kid_neverwanttosolvehardtask+

y9_kid_angrywtroublelearning+

y9_kid_neverhelphome+

y9_kid_pickedoninschool+

y9_kid_beateninschool+

y9_kid_leftoutinschool+

y9_kid_damagedproperty+

y9_kid_stolensmthng+

y9_kid_fistfight+

y9_kid_runawayhome+

y9_kid_suspendedschool+

y9_kid_grafitti+

y9_kid_setfire,

data = expl, seed = 1)$data

lowbwt.bglm <- data.rose[, c("y9_kid_notfeelclosepeopleatschool",

"y9_kid_neverwanttosolvehardtask",

"y9_kid_angrywtroublelearning",

"y9_kid_neverhelphome",

"y9_kid_pickedoninschool",

"y9_kid_beateninschool",

"y9_kid_leftoutinschool",

"y9_kid_damagedproperty",

"y9_kid_stolensmthng",

"y9_kid_fistfight",

"y9_kid_runawayhome",

"y9_kid_suspendedschool",

"y9_kid_grafitti",

"y9_kid_setfire", "adhd")]

names(lowbwt.bglm)[names(lowbwt.bglm) == "adhd"] <- "y"

lowbwt.bglm <- as.data.frame(lowbwt.bglm)

best.logit <- bestglm(lowbwt.bglm,

IC = "AIC",

family=binomial,

method = "exhaustive")

summary(best.logit$BestModel)

#y9_kid_angrywtroublelearning 0.16595 0.10111 1.641 0.10076

#y9_kid_neverhelphome 0.58125 0.10532 5.519 3.42e-08 ***

#y9_kid_pickedoninschool 0.15900 0.08471 1.877 0.06053 .

#y9_kid_leftoutinschool 0.25581 0.13006 1.967 0.04920 *

#y9_kid_stolensmthng 0.28282 0.11043 2.561 0.01043 *

#y9_kid_fistfight 0.29976 0.07694 3.896 9.78e-05 ***

#y9_kid_suspendedschool 0.61956 0.08935 6.934 4.09e-12 ***

#y9_kid_grafitti 0.49193 0.17321 2.840 0.00451 **

#y9_kid_setfire 0.60959 0.24325 2.506 0.01221 *

data.rose <- ROSE(adhd ~ y9_kid_rockatpeopleorcars+

y9_kid_neverorderly+

y9_kid_notdobest+

y9_kid_notfollowthrough+

y9_kid_arguewothers+

y9_kid_hardpayattention+

y9_kid_feellonely+

y9_kid_easilydistracted+

y9_kid_sad+

y9_kid_hardfinishschoolwork+

y9_kid_worrynoonetoplay+

y9_kid_distrubingothers+

y9_kid_fightingothers+

y9_kid_seldomseatbelt,

data = expl, seed = 1)$data

lowbwt.bglm <- data.rose[, c("y9_kid_rockatpeopleorcars",

"y9_kid_neverorderly",

"y9_kid_notdobest",

"y9_kid_notfollowthrough",

"y9_kid_arguewothers",

"y9_kid_hardpayattention",

"y9_kid_feellonely",

"y9_kid_easilydistracted",

"y9_kid_sad",

"y9_kid_hardfinishschoolwork",

"y9_kid_worrynoonetoplay",

"y9_kid_distrubingothers",

"y9_kid_fightingothers",

"y9_kid_seldomseatbelt", "adhd")]

names(lowbwt.bglm)[names(lowbwt.bglm) == "adhd"] <- "y"

lowbwt.bglm <- as.data.frame(lowbwt.bglm)

best.logit <- bestglm(lowbwt.bglm,

IC = "AIC",

family=binomial,

method = "exhaustive")

summary(best.logit$BestModel)

#y9_kid_rockatpeopleorcars 0.45616 0.13911 3.279 0.001041 **

#y9_kid_notdobest 0.40637 0.20346 1.997 0.045790 *

#y9_kid_notfollowthrough 0.39500 0.12190 3.240 0.001194 **

#y9_kid_hardpayattention 0.15571 0.10275 1.516 0.129636

#y9_kid_feellonely 0.41149 0.11394 3.612 0.000304 ***

#y9_kid_easilydistracted 0.33195 0.08348 3.976 7.00e-05 ***

#y9_kid_hardfinishschoolwork 0.53039 0.10978 4.831 1.36e-06 ***

#y9_kid_worrynoonetoplay 0.19029 0.09278 2.051 0.040271 *

#y9_kid_fightingothers 0.13745 0.09686 1.419 0.155895

#y9_kid_seldomseatbelt 0.50078 0.15540 3.222 0.001271 **

##### Final model

data.rose <- ROSE(adhd ~ y9_kid_neverhelphome+

y9_kid_fistfight+

y9_kid_suspendedschool+

y9_kid_grafitti+

y9_kid_rockatpeopleorcars+

y9_kid_notfollowthrough+

y9_kid_feellonely+

y9_kid_easilydistracted+

y9_kid_hardfinishschoolwork+

y9_kid_seldomseatbelt,

data = expl, seed = 1)$data

lowbwt.bglm <- data.rose[, c("y9_kid_neverhelphome",

"y9_kid_fistfight",

"y9_kid_suspendedschool",

"y9_kid_grafitti",

"y9_kid_rockatpeopleorcars",

"y9_kid_notfollowthrough",

"y9_kid_feellonely",

"y9_kid_easilydistracted",

"y9_kid_hardfinishschoolwork",

"y9_kid_seldomseatbelt", "adhd")]

names(lowbwt.bglm)[names(lowbwt.bglm) == "adhd"] <- "y"

lowbwt.bglm <- as.data.frame(lowbwt.bglm)

best.logit <- bestglm(lowbwt.bglm,

IC = "AIC",

family=binomial,

method = "exhaustive")

summary(best.logit$BestModel)

#y9_kid_neverhelphome 0.25800 0.10797 2.390 0.01687 *

#y9_kid_fistfight 0.42630 0.08180 5.211 1.88e-07 ***

#y9_kid_suspendedschool 0.40916 0.09355 4.374 1.22e-05 ***

#y9_kid_grafitti 0.37821 0.17562 2.154 0.03127 *

#y9_kid_rockatpeopleorcars 0.35601 0.15144 2.351 0.01873 *

#y9_kid_notfollowthrough 0.22885 0.13106 1.746 0.08078 .

#y9_kid_feellonely 0.32786 0.11324 2.895 0.00379 **

#y9_kid_easilydistracted 0.52070 0.08807 5.912 3.37e-09 ***

#y9_kid_hardfinishschoolwork 0.43231 0.10931 3.955 7.65e-05 ***

#y9_kid_seldomseatbelt 0.36455 0.15817 2.305 0.02117 *

# Examine accuracy in non-imputed holdout sample

glm_probs <- data.frame(probs = predict(best.logit$BestModel, newdata = holdout,type="response"))

glm_pred <- glm_probs %>% mutate(pred = ifelse(probs>.5, "1", "0"))

glm_pred <- cbind(holdout, glm_pred)

glm_pred %>% count(pred, adhd) %>% spread(adhd, n, fill = 0)

# pred 0 1

#1 0 370 52

#2 1 138 40

#3 <NA> 91 24

#Sensitivity

40/(40+52)*100 # 43.5%

#PPV

40/(138+40)*100 # 22.5%

### Test scores

# Year 3

rvocaby3 <- glm(adhd ~ male + ch3ppvtraw, data = expl, family = "binomial")

summary(rvocaby3) # * Not under .01

# Year 5

rvocaby5 <- glm(adhd ~ male + ch4ppvtraw, data = expl, family = "binomial")

rleiteratt5 <- glm(adhd ~ male + ch4lr_corscor, data = expl, family = "binomial")

rleiterimp5 <- glm(adhd ~ male + ch4lr_errscor, data = expl, family = "binomial")

summary(rvocaby5) # **

summary(rleiteratt5) # **

summary(rleiterimp5) # ***

# Year 9

rvocaby9 <- glm(adhd ~ male + ch5ppvtraw, data = expl, family = "binomial")

rwoodcock9 <- glm(adhd ~ male + ch5wj9raw, data = expl, family = "binomial")

rwoodcock10 <- glm(adhd ~ male + ch5wj10raw, data = expl, family = "binomial")

rdigitspan <- glm(adhd ~ male + ch5dsraw, data = expl, family = "binomial")

summary(rvocaby9) # ***

summary(rwoodcock9) # ***

summary(rwoodcock10) # ***

summary(rdigitspan) # ***

aggregate(ch4ppvtraw ~ adhd, expl, mean)

aggregate(ch4ppvtraw ~ adhd, expl, sd)

# Vocabulary year 5

# Mean SD

# no adhd 62.77719 19.04590

# adhd 58.58199 20.24652

aggregate(ch4lr_corscor ~ adhd, expl, mean)

aggregate(ch4lr_corscor ~ adhd, expl, sd)

# Leiter attention year 5

# Mean SD

# no adhd 12.98904 3.176504

# adhd 12.09191 3.324239

aggregate(ch4lr_errscor ~ adhd, expl, mean)

aggregate(ch4lr_errscor ~ adhd, expl, sd)

# Leiter impulsivity year 5

# Mean SD

# no adhd 10.272344 2.734841

# adhd 9.220588 3.010906

aggregate(ch5ppvtraw ~ adhd, expl, mean)

aggregate(ch5ppvtraw ~ adhd, expl, sd)

# Vocabulary year 9

# Mean SD

# no adhd 112.1135 20.32689

# adhd 107.9455 20.48460

aggregate(ch5wj9raw ~ adhd, expl, mean)

aggregate(ch5wj9raw ~ adhd, expl, sd)

# Language W-J year 9

# Mean SD

# no adhd 26.00098 5.493969

# adhd 23.58257 6.191211

aggregate(ch5wj10raw ~ adhd, expl, mean)

aggregate(ch5wj10raw ~ adhd, expl, sd)

# Math W-J year 9

# Mean SD

# no adhd 32.68238 6.150275

# adhd 30.07973 6.284032

aggregate(ch5dsraw ~ adhd, expl, mean)

aggregate(ch5dsraw ~ adhd, expl, sd)

# Working Mem WISV year 9

# Mean SD

# no adhd 14.06043 3.128681

# adhd 12.99548 3.085329

# Create evenly distributed synthetic data

data.rose <- ROSE(adhd ~ ch4ppvtraw+ch4lr_corscor+ch4lr_errscor+ch5ppvtraw+ch5wj9raw+ch5wj10raw+ch5dsraw,

data = expl, seed = 1)$data

# Run bestglm to find best subset model. Use syntehetic data to get better coefficients to find cases

lowbwt.bglm <- data.rose[, c("ch4ppvtraw","ch4lr_corscor","ch4lr_errscor",

"ch5ppvtraw","ch5wj9raw","ch5wj10raw","ch5dsraw","adhd")]

names(lowbwt.bglm)[names(lowbwt.bglm) == "adhd"] <- "y"

lowbwt.bglm <- as.data.frame(lowbwt.bglm)

best.logit <- bestglm(lowbwt.bglm,

IC = "AIC",

family=binomial,

method = "exhaustive")

summary(best.logit$BestModel)

# ch4lr_corscor -0.02880 0.01602 -1.797 0.07234 .

# ch4lr_errscor -0.06885 0.01780 -3.868 0.00011 *** impulsivity year 5

# ch5wj9raw -0.02146 0.01035 -2.074 0.03806 *

# ch5wj10raw -0.05824 0.01124 -5.180 2.22e-07 *** Math year 9

# ch5dsraw -0.05625 0.01958 -2.872 0.00407 ** Working memory year 9

# Year 5: ch4lr_errscor

# Year 9: ch5wj10raw + ch5dsraw

### Add them to year-specific models

### Year 5 Teacher

data.rose <- ROSE(adhd ~ y5_teacher_seldomworktobestability+y5_teacher_diagnoseddisability+y5_teacher_easilynameletters+y5_teacher_understconvofprint+

y5_teacher_recogndiffpeople+y5_teacher_sortscompmath+y5_teacher_relofquantities+y5_teacher_varietyinmath+y5_teacher_belowaverlanguage+

y5_teacher_belowaversocialsci+y5_teacher_belowavermath+y5_teacher_activestrucutured+y5_teacher_activeunstrucutured+y5_teacher_discussproblsparents+ch4lr_errscor,

data = expl, seed = 1)$data

lowbwt.bglm <- data.rose[, c("y5_teacher_seldomworktobestability","y5_teacher_diagnoseddisability","y5_teacher_easilynameletters","y5_teacher_understconvofprint",

"y5_teacher_recogndiffpeople","y5_teacher_sortscompmath","y5_teacher_relofquantities","y5_teacher_varietyinmath","y5_teacher_belowaverlanguage",

"y5_teacher_belowaversocialsci","y5_teacher_belowavermath","y5_teacher_activestrucutured","y5_teacher_activeunstrucutured","y5_teacher_discussproblsparents","ch4lr_errscor","adhd")]

names(lowbwt.bglm)[names(lowbwt.bglm) == "adhd"] <- "y"

lowbwt.bglm <- as.data.frame(lowbwt.bglm)

best.logit <- bestglm(lowbwt.bglm,

IC = "AIC",

family=binomial,

method = "exhaustive")

summary(best.logit$BestModel)

#y5_teacher_seldomworktobestability 0.6597 0.1900 3.472 0.000516 ***

#y5_teacher_diagnoseddisability 1.3138 0.2453 5.356 8.49e-08 ***

#y5_teacher_sortscompmath 0.4615 0.2932 1.574 0.115448

#y5_teacher_relofquantities -0.5840 0.2595 -2.251 0.024400 *

#y5_teacher_varietyinmath 0.3487 0.2314 1.507 0.131790

#y5_teacher_belowavermath 0.3742 0.1781 2.101 0.035615 *

#y5_teacher_discussproblsparents 0.3590 0.1643 2.185 0.028907 *

### Year 9 Teacher + tests

##### Final model

data.rose <- ROSE(adhd ~ y9_teacher_makesfriendseasy+

y9_teacher_responapprteasing+

y9_teacher_cleandesk+

y9_teacher_transitseasybtwactiv+

y9_teacher_anxingroupchildren+

y9_teacher_angryeasily+

y9_teacher_inattentive+

y9_teacher_leavesseat+

y9_teacher_poorreading+

y9_teacher_temperoutbursts+

y9_teacher_excitable+

ch5wj10raw+

ch5dsraw,

data = expl, seed = 1)$data

lowbwt.bglm <- data.rose[, c("y9_teacher_makesfriendseasy",

"y9_teacher_responapprteasing",

"y9_teacher_cleandesk",

"y9_teacher_transitseasybtwactiv",

"y9_teacher_anxingroupchildren",

"y9_teacher_angryeasily",

"y9_teacher_inattentive",

"y9_teacher_leavesseat",

"y9_teacher_poorreading",

"y9_teacher_temperoutbursts",

"y9_teacher_excitable",

"ch5wj10raw",

"ch5dsraw",

"adhd")]

names(lowbwt.bglm)[names(lowbwt.bglm) == "adhd"] <- "y"

lowbwt.bglm <- as.data.frame(lowbwt.bglm)

best.logit <- bestglm(lowbwt.bglm,

IC = "AIC",

family=binomial,

method = "exhaustive")

summary(best.logit$BestModel)

#y9_teacher_responapprteasing -0.21933 0.11922 -1.840 0.065795 .

#y9_teacher_cleandesk -0.49133 0.11191 -4.391 1.13e-05 ***

#y9_teacher_transitseasybtwactiv -0.21079 0.12083 -1.745 0.081071 .

#y9_teacher_anxingroupchildren 0.39936 0.11618 3.437 0.000587 ***

#y9_teacher_angryeasily 0.40820 0.11946 3.417 0.000633 ***

#y9_teacher_inattentive 0.54851 0.12373 4.433 9.28e-06 ***

#y9_teacher_leavesseat 0.64743 0.14670 4.413 1.02e-05 ***

#y9_teacher_poorreading 0.18437 0.11355 1.624 0.104435

#y9_teacher_temperoutbursts 0.42458 0.18777 2.261 0.023750 *

#y9_teacher_excitable 0.68629 0.14817 4.632 3.63e-06 ***

#ch5dsraw -0.04517 0.01774 -2.547 0.010859 *

################################################################################

##############

############## Final model

##############

################################################################################

#### NOT USED ---- SEE BELOW --- Import exploratory imputation sets --- USED to see which variables are sign associated with adhd status

#

###### Run model on full imputed dataset

#fullmodelsign <- with(data=explimplong,exp=glm(adhd~baseline_fatherinjail+

# y5_mother_dep+

# y5_speechproblem+

# y5_er_accident_yes+

# y9_phys_speechproblem+

# y3_cantconcentr+

# y3_cantsitstill+

# y3_distrbdchroutine+

# y3_tempertantrums+

# y5_disobedientschool+

# y5_impulsive+

# y5_preferolderkids+

# y5_cantsitstill+

# y5_stubborn+

# y5_wantsalotattention+

# y5_actstooyoungforage+

# y5_teacher_seldomworktobestability+

# y5_teacher_diagnoseddisability+

# y5_teacher_relofquantities+

# y5_teacher_activeunstrucutured+

# y5_teacher_discussproblsparents+

# y9_parent_failstofinish+

# y9_parent_cantconcentrate+

# y9_parent_restless+

# y9_parent_easydistracted+

# y9_parent_tempertantrums+

# y9_parent_reportsaccappropr+

# y9_teacher_makesfriendseasy+

# y9_teacher_cleandesk+

# y9_teacher_anxingroupchildren+

# y9_teacher_angryeasily+

# y9_teacher_inattentive+

# y9_teacher_leavesseat+

# y9_kid_fistfight+

# y9_kid_suspendedschool+

# y9_kid_feellonely+

# y9_kid_easilydistracted+

# y9_kid_hardfinishschoolwork, family="binomial"))

#

#resfullmodelsign <- pool(fullmodelsign)

#resfullmodelsign<-summary(resfullmodelsign, conf.int = TRUE)

#mutate_if(resfullmodelsign,is.numeric, round, 3)

#

#

##1 (Intercept) -2.917 0.327 -8.930 13.697 0.000 -3.619 -2.215

##2 baseline_fatherinjail 0.537 0.269 1.994 153.490 0.048 0.005 1.068 *

##3 y5_mother_dep -0.045 0.175 -0.258 251.902 0.797 -0.391 0.300

##4 y5_speechproblem -0.109 0.240 -0.454 46.858 0.652 -0.593 0.375

##5 y5_er_accident_yes 0.126 0.189 0.665 33.046 0.511 -0.259 0.511

##6 y9_phys_speechproblem 0.552 0.188 2.943 615.635 0.003 0.184 0.920 **

##7 y3_cantconcentr 0.185 0.224 0.828 25.422 0.415 -0.275 0.646

##8 y3_cantsitstill 0.066 0.187 0.350 19.770 0.730 -0.325 0.456

##9 y3_distrbdchroutine -0.075 0.207 -0.365 59.706 0.717 -0.489 0.338

##10 y3_tempertantrums -0.011 0.173 -0.064 79.213 0.949 -0.355 0.333

##11 y5_disobedientschool 0.201 0.245 0.819 81.886 0.415 -0.287 0.688

##12 y5_impulsive -0.480 0.235 -2.048 58.314 0.045 -0.950 -0.011 *

##13 y5_preferolderkids 0.017 0.195 0.087 14.730 0.932 -0.400 0.434

##14 y5_cantsitstill 0.290 0.163 1.784 120.828 0.077 -0.032 0.613

##15 y5_stubborn 0.367 0.214 1.716 29.356 0.097 -0.070 0.804

##16 y5_wantsalotattention 0.197 0.139 1.417 103.724 0.159 -0.079 0.474

##17 y5_actstooyoungforage -0.497 0.304 -1.637 28.122 0.113 -1.119 0.125

##18 y5_teacher_seldomworktobestability 0.118 0.223 0.526 8.549 0.612 -0.392 0.627

##19 y5_teacher_diagnoseddisability 0.170 0.177 0.960 15.543 0.352 -0.206 0.546

##20 y5_teacher_relofquantities -0.034 0.266 -0.127 6.386 0.903 -0.675 0.608

##21 y5_teacher_activeunstrucutured 0.306 0.296 1.032 6.254 0.340 -0.412 1.023

##22 y5_teacher_discussproblsparents 0.224 0.247 0.910 7.062 0.393 -0.357 0.806

##23 y9_parent_failstofinish -0.136 0.213 -0.637 1846.904 0.524 -0.553 0.282

##24 y9_parent_cantconcentrate 0.742 0.231 3.213 51.152 0.002 0.278 1.205 **

##25 y9_parent_restless 1.005 0.222 4.528 23.643 0.000 0.546 1.463 ***

##26 y9_parent_easydistracted 0.216 0.240 0.897 52.856 0.374 -0.266 0.698

##27 y9_parent_tempertantrums -0.242 0.309 -0.783 14.960 0.446 -0.900 0.417

##28 y9_parent_reportsaccappropr -0.252 0.137 -1.837 43.664 0.073 -0.528 0.024

##29 y9_teacher_makesfriendseasy -0.202 0.245 -0.823 7.459 0.436 -0.774 0.371

##30 y9_teacher_cleandesk -0.338 0.205 -1.650 10.794 0.128 -0.790 0.114

##31 y9_teacher_anxingroupchildren 0.252 0.170 1.483 17.004 0.156 -0.107 0.612

##32 y9_teacher_angryeasily 0.587 0.203 2.883 9.959 0.016 0.133 1.040 *

##33 y9_teacher_inattentive 0.473 0.186 2.547 15.120 0.022 0.077 0.869 *

##34 y9_teacher_leavesseat 0.482 0.148 3.251 50.901 0.002 0.185 0.780 **

##35 y9_kid_fistfight 0.144 0.150 0.961 43.228 0.342 -0.158 0.447

##36 y9_kid_suspendedschool -0.022 0.177 -0.127 26.267 0.900 -0.387 0.342

##37 y9_kid_feellonely -0.107 0.226 -0.473 16.249 0.642 -0.586 0.372

##38 y9_kid_easilydistracted 0.240 0.143 1.678 165.589 0.095 -0.042 0.523

##39 y9_kid_hardfinishschoolwork 0.009 0.183 0.047 80.437 0.963 -0.355 0.372

### Find significant variables across imputed datasets using synthesized data

explimp1 <- read_sav("Data/imputations adhd/explimp1.sav")

explimp2 <- read_sav("Data/imputations adhd/explimp2.sav")

explimp3 <- read_sav("Data/imputations adhd/explimp3.sav")

explimp4 <- read_sav("Data/imputations adhd/explimp4.sav")

explimp5 <- read_sav("Data/imputations adhd/explimp5.sav")

data.rose1 <- ROSE(adhd ~ male+baseline_fatherinjail+

y5_mother_dep+

y5_speechproblem+

y5_er_accident_yes+

y9_phys_speechproblem+

y3_cantconcentr+

y3_cantsitstill+

y3_distrbdchroutine+

y3_tempertantrums+

y5_disobedientschool+

y5_impulsive+

y5_preferolderkids+

y5_cantsitstill+

y5_stubborn+

y5_wantsalotattention+

y5_actstooyoungforage+

y5_teacher_seldomworktobestability+

y5_teacher_diagnoseddisability+

y5_teacher_relofquantities+

y5_teacher_activeunstrucutured+

y5_teacher_discussproblsparents+

y9_parent_failstofinish+

y9_parent_cantconcentrate+

y9_parent_restless+

y9_parent_easydistracted+

y9_parent_tempertantrums+

y9_parent_reportsaccappropr+

y9_teacher_makesfriendseasy+

y9_teacher_cleandesk+

y9_teacher_anxingroupchildren+

y9_teacher_angryeasily+

y9_teacher_inattentive+

y9_teacher_leavesseat+

y9_kid_fistfight+

y9_kid_suspendedschool+

y9_kid_feellonely+

y9_kid_easilydistracted+

y9_kid_hardfinishschoolwork,

data = explimp1, seed = 1)$data

data.rose2 <- ROSE(adhd ~ male + baseline_fatherinjail+

y5_mother_dep+

y5_speechproblem+

y5_er_accident_yes+

y9_phys_speechproblem+

y3_cantconcentr+

y3_cantsitstill+

y3_distrbdchroutine+

y3_tempertantrums+

y5_disobedientschool+

y5_impulsive+

y5_preferolderkids+

y5_cantsitstill+

y5_stubborn+

y5_wantsalotattention+

y5_actstooyoungforage+

y5_teacher_seldomworktobestability+

y5_teacher_diagnoseddisability+

y5_teacher_relofquantities+

y5_teacher_activeunstrucutured+

y5_teacher_discussproblsparents+

y9_parent_failstofinish+

y9_parent_cantconcentrate+

y9_parent_restless+

y9_parent_easydistracted+

y9_parent_tempertantrums+

y9_parent_reportsaccappropr+

y9_teacher_makesfriendseasy+

y9_teacher_cleandesk+

y9_teacher_anxingroupchildren+

y9_teacher_angryeasily+

y9_teacher_inattentive+

y9_teacher_leavesseat+

y9_kid_fistfight+

y9_kid_suspendedschool+

y9_kid_feellonely+

y9_kid_easilydistracted+

y9_kid_hardfinishschoolwork,

data = explimp2, seed = 1)$data

data.rose3 <- ROSE(adhd ~ male+baseline_fatherinjail+

y5_mother_dep+

y5_speechproblem+

y5_er_accident_yes+

y9_phys_speechproblem+

y3_cantconcentr+

y3_cantsitstill+

y3_distrbdchroutine+

y3_tempertantrums+

y5_disobedientschool+

y5_impulsive+

y5_preferolderkids+

y5_cantsitstill+

y5_stubborn+

y5_wantsalotattention+

y5_actstooyoungforage+

y5_teacher_seldomworktobestability+

y5_teacher_diagnoseddisability+

y5_teacher_relofquantities+

y5_teacher_activeunstrucutured+

y5_teacher_discussproblsparents+

y9_parent_failstofinish+

y9_parent_cantconcentrate+

y9_parent_restless+

y9_parent_easydistracted+

y9_parent_tempertantrums+

y9_parent_reportsaccappropr+

y9_teacher_makesfriendseasy+

y9_teacher_cleandesk+

y9_teacher_anxingroupchildren+

y9_teacher_angryeasily+

y9_teacher_inattentive+

y9_teacher_leavesseat+

y9_kid_fistfight+

y9_kid_suspendedschool+

y9_kid_feellonely+

y9_kid_easilydistracted+

y9_kid_hardfinishschoolwork,

data = explimp3, seed = 1)$data

data.rose4 <- ROSE(adhd ~ male+baseline_fatherinjail+

y5_mother_dep+

y5_speechproblem+

y5_er_accident_yes+

y9_phys_speechproblem+

y3_cantconcentr+

y3_cantsitstill+

y3_distrbdchroutine+

y3_tempertantrums+

y5_disobedientschool+

y5_impulsive+

y5_preferolderkids+

y5_cantsitstill+

y5_stubborn+

y5_wantsalotattention+

y5_actstooyoungforage+

y5_teacher_seldomworktobestability+

y5_teacher_diagnoseddisability+

y5_teacher_relofquantities+

y5_teacher_activeunstrucutured+

y5_teacher_discussproblsparents+

y9_parent_failstofinish+

y9_parent_cantconcentrate+

y9_parent_restless+

y9_parent_easydistracted+

y9_parent_tempertantrums+

y9_parent_reportsaccappropr+

y9_teacher_makesfriendseasy+

y9_teacher_cleandesk+

y9_teacher_anxingroupchildren+

y9_teacher_angryeasily+

y9_teacher_inattentive+

y9_teacher_leavesseat+

y9_kid_fistfight+

y9_kid_suspendedschool+

y9_kid_feellonely+

y9_kid_easilydistracted+

y9_kid_hardfinishschoolwork,

data = explimp4, seed = 1)$data

data.rose5 <- ROSE(adhd ~ male+baseline_fatherinjail+

y5_mother_dep+

y5_speechproblem+

y5_er_accident_yes+

y9_phys_speechproblem+

y3_cantconcentr+

y3_cantsitstill+

y3_distrbdchroutine+

y3_tempertantrums+

y5_disobedientschool+

y5_impulsive+

y5_preferolderkids+

y5_cantsitstill+

y5_stubborn+

y5_wantsalotattention+

y5_actstooyoungforage+

y5_teacher_seldomworktobestability+

y5_teacher_diagnoseddisability+

y5_teacher_relofquantities+

y5_teacher_activeunstrucutured+

y5_teacher_discussproblsparents+

y9_parent_failstofinish+

y9_parent_cantconcentrate+

y9_parent_restless+

y9_parent_easydistracted+

y9_parent_tempertantrums+

y9_parent_reportsaccappropr+

y9_teacher_makesfriendseasy+

y9_teacher_cleandesk+

y9_teacher_anxingroupchildren+

y9_teacher_angryeasily+

y9_teacher_inattentive+

y9_teacher_leavesseat+

y9_kid_fistfight+

y9_kid_suspendedschool+

y9_kid_feellonely+

y9_kid_easilydistracted+

y9_kid_hardfinishschoolwork,

data = explimp5, seed = 1)$data

fullmodel1 <- glm(adhd~male+baseline_fatherinjail+

y5_mother_dep+

y5_speechproblem+

y5_er_accident_yes+

y9_phys_speechproblem+

y3_cantconcentr+

y3_cantsitstill+

y3_distrbdchroutine+

y3_tempertantrums+

y5_disobedientschool+

y5_impulsive+

y5_preferolderkids+

y5_cantsitstill+

y5_stubborn+

y5_wantsalotattention+

y5_actstooyoungforage+

y5_teacher_seldomworktobestability+

y5_teacher_diagnoseddisability+

y5_teacher_relofquantities+

y5_teacher_activeunstrucutured+

y5_teacher_discussproblsparents+

y9_parent_failstofinish+

y9_parent_cantconcentrate+

y9_parent_restless+

y9_parent_easydistracted+

y9_parent_tempertantrums+

y9_parent_reportsaccappropr+

y9_teacher_makesfriendseasy+

y9_teacher_cleandesk+

y9_teacher_anxingroupchildren+

y9_teacher_angryeasily+

y9_teacher_inattentive+

y9_teacher_leavesseat+

y9_kid_fistfight+

y9_kid_suspendedschool+

y9_kid_feellonely+

y9_kid_easilydistracted+

y9_kid_hardfinishschoolwork, data=data.rose1, family="binomial")

fullmodel2 <- glm(adhd~male+baseline_fatherinjail+

y5_mother_dep+

y5_speechproblem+

y5_er_accident_yes+

y9_phys_speechproblem+

y3_cantconcentr+

y3_cantsitstill+

y3_distrbdchroutine+

y3_tempertantrums+

y5_disobedientschool+

y5_impulsive+

y5_preferolderkids+

y5_cantsitstill+

y5_stubborn+

y5_wantsalotattention+

y5_actstooyoungforage+

y5_teacher_seldomworktobestability+

y5_teacher_diagnoseddisability+

y5_teacher_relofquantities+

y5_teacher_activeunstrucutured+

y5_teacher_discussproblsparents+

y9_parent_failstofinish+

y9_parent_cantconcentrate+

y9_parent_restless+

y9_parent_easydistracted+

y9_parent_tempertantrums+

y9_parent_reportsaccappropr+

y9_teacher_makesfriendseasy+

y9_teacher_cleandesk+

y9_teacher_anxingroupchildren+

y9_teacher_angryeasily+

y9_teacher_inattentive+

y9_teacher_leavesseat+

y9_kid_fistfight+

y9_kid_suspendedschool+

y9_kid_feellonely+

y9_kid_easilydistracted+

y9_kid_hardfinishschoolwork, data=data.rose2, family="binomial")

fullmodel3 <- glm(adhd~male+baseline_fatherinjail+

y5_mother_dep+

y5_speechproblem+

y5_er_accident_yes+

y9_phys_speechproblem+

y3_cantconcentr+

y3_cantsitstill+

y3_distrbdchroutine+

y3_tempertantrums+

y5_disobedientschool+

y5_impulsive+

y5_preferolderkids+

y5_cantsitstill+

y5_stubborn+

y5_wantsalotattention+

y5_actstooyoungforage+

y5_teacher_seldomworktobestability+

y5_teacher_diagnoseddisability+

y5_teacher_relofquantities+

y5_teacher_activeunstrucutured+

y5_teacher_discussproblsparents+

y9_parent_failstofinish+

y9_parent_cantconcentrate+

y9_parent_restless+

y9_parent_easydistracted+

y9_parent_tempertantrums+

y9_parent_reportsaccappropr+

y9_teacher_makesfriendseasy+

y9_teacher_cleandesk+

y9_teacher_anxingroupchildren+

y9_teacher_angryeasily+

y9_teacher_inattentive+

y9_teacher_leavesseat+

y9_kid_fistfight+

y9_kid_suspendedschool+

y9_kid_feellonely+

y9_kid_easilydistracted+

y9_kid_hardfinishschoolwork, data=data.rose3, family="binomial")

fullmodel4 <- glm(adhd~male+baseline_fatherinjail+

y5_mother_dep+

y5_speechproblem+

y5_er_accident_yes+

y9_phys_speechproblem+

y3_cantconcentr+

y3_cantsitstill+

y3_distrbdchroutine+

y3_tempertantrums+

y5_disobedientschool+

y5_impulsive+

y5_preferolderkids+

y5_cantsitstill+

y5_stubborn+

y5_wantsalotattention+

y5_actstooyoungforage+

y5_teacher_seldomworktobestability+

y5_teacher_diagnoseddisability+

y5_teacher_relofquantities+

y5_teacher_activeunstrucutured+

y5_teacher_discussproblsparents+

y9_parent_failstofinish+

y9_parent_cantconcentrate+

y9_parent_restless+

y9_parent_easydistracted+

y9_parent_tempertantrums+

y9_parent_reportsaccappropr+

y9_teacher_makesfriendseasy+

y9_teacher_cleandesk+

y9_teacher_anxingroupchildren+

y9_teacher_angryeasily+

y9_teacher_inattentive+

y9_teacher_leavesseat+

y9_kid_fistfight+

y9_kid_suspendedschool+

y9_kid_feellonely+

y9_kid_easilydistracted+

y9_kid_hardfinishschoolwork, data=data.rose4, family="binomial")

fullmodel5 <- glm(adhd~male+baseline_fatherinjail+

y5_mother_dep+

y5_speechproblem+

y5_er_accident_yes+

y9_phys_speechproblem+

y3_cantconcentr+

y3_cantsitstill+

y3_distrbdchroutine+

y3_tempertantrums+

y5_disobedientschool+

y5_impulsive+

y5_preferolderkids+

y5_cantsitstill+

y5_stubborn+

y5_wantsalotattention+

y5_actstooyoungforage+

y5_teacher_seldomworktobestability+

y5_teacher_diagnoseddisability+

y5_teacher_relofquantities+

y5_teacher_activeunstrucutured+

y5_teacher_discussproblsparents+

y9_parent_failstofinish+

y9_parent_cantconcentrate+

y9_parent_restless+

y9_parent_easydistracted+

y9_parent_tempertantrums+

y9_parent_reportsaccappropr+

y9_teacher_makesfriendseasy+

y9_teacher_cleandesk+

y9_teacher_anxingroupchildren+

y9_teacher_angryeasily+

y9_teacher_inattentive+

y9_teacher_leavesseat+

y9_kid_fistfight+

y9_kid_suspendedschool+

y9_kid_feellonely+

y9_kid_easilydistracted+

y9_kid_hardfinishschoolwork, data=data.rose5, family="binomial")

## See which variables are sign predictive in all models

summary(fullmodel1)

summary(fullmodel2)

summary(fullmodel3)

summary(fullmodel4)

summary(fullmodel5)

###### Use only the variables selected above

data.rose1 <- ROSE(adhd ~ male+

y9_phys_speechproblem+

y5_cantsitstill+

y9_parent_cantconcentrate+

y9_parent_restless+

y9_parent_easydistracted+

y9_teacher_anxingroupchildren+

y9_teacher_angryeasily+

y9_teacher_inattentive+

y9_teacher_leavesseat,

data = explimp1, seed = 1)$data

data.rose2 <- ROSE(adhd ~ male+

y9_phys_speechproblem+

y5_cantsitstill+

y9_parent_cantconcentrate+

y9_parent_restless+

y9_parent_easydistracted+

y9_teacher_anxingroupchildren+

y9_teacher_angryeasily+

y9_teacher_inattentive+

y9_teacher_leavesseat,

data = explimp2, seed = 1)$data

data.rose3 <- ROSE(adhd ~ male+

y9_phys_speechproblem+

y5_cantsitstill+

y9_parent_cantconcentrate+

y9_parent_restless+

y9_parent_easydistracted+

y9_teacher_anxingroupchildren+

y9_teacher_angryeasily+

y9_teacher_inattentive+

y9_teacher_leavesseat,

data = explimp3, seed = 1)$data

data.rose4 <- ROSE(adhd ~ male+

y9_phys_speechproblem+

y5_cantsitstill+

y9_parent_cantconcentrate+

y9_parent_restless+

y9_parent_easydistracted+

y9_teacher_anxingroupchildren+

y9_teacher_angryeasily+

y9_teacher_inattentive+

y9_teacher_leavesseat,

data = explimp4, seed = 1)$data

data.rose5 <- ROSE(adhd ~ male+

y9_phys_speechproblem+

y5_cantsitstill+

y9_parent_cantconcentrate+

y9_parent_restless+

y9_parent_easydistracted+

y9_teacher_anxingroupchildren+

y9_teacher_angryeasily+

y9_teacher_inattentive+

y9_teacher_leavesseat,

data = explimp5, seed = 1)$data

fullmodel1 <- glm(adhd~male+

y9_phys_speechproblem+

y5_cantsitstill+

y9_parent_cantconcentrate+

y9_parent_restless+

y9_parent_easydistracted+

y9_teacher_anxingroupchildren+

y9_teacher_angryeasily+

y9_teacher_inattentive+

y9_teacher_leavesseat, data=data.rose1, family="binomial")

fullmodel2 <- glm(adhd~male+

y9_phys_speechproblem+

y5_cantsitstill+

y9_parent_cantconcentrate+

y9_parent_restless+

y9_parent_easydistracted+

y9_teacher_anxingroupchildren+

y9_teacher_angryeasily+

y9_teacher_inattentive+

y9_teacher_leavesseat, data=data.rose2, family="binomial")

fullmodel3 <- glm(adhd~male+

y9_phys_speechproblem+

y5_cantsitstill+

y9_parent_cantconcentrate+

y9_parent_restless+

y9_parent_easydistracted+

y9_teacher_anxingroupchildren+

y9_teacher_angryeasily+

y9_teacher_inattentive+

y9_teacher_leavesseat, data=data.rose3, family="binomial")

fullmodel4 <- glm(adhd~male+

y9_phys_speechproblem+

y5_cantsitstill+

y9_parent_cantconcentrate+

y9_parent_restless+

y9_parent_easydistracted+

y9_teacher_anxingroupchildren+

y9_teacher_angryeasily+

y9_teacher_inattentive+

y9_teacher_leavesseat, data=data.rose4, family="binomial")

fullmodel5 <- glm(adhd~male+

y9_phys_speechproblem+

y5_cantsitstill+

y9_parent_cantconcentrate+

y9_parent_restless+

y9_parent_easydistracted+

y9_teacher_anxingroupchildren+

y9_teacher_angryeasily+

y9_teacher_inattentive+

y9_teacher_leavesseat, data=data.rose5, family="binomial")

### Pool coefficients

c1 <- as.data.frame(fullmodel1$coefficients)

c2 <- as.data.frame(fullmodel2$coefficients)

c3 <- as.data.frame(fullmodel3$coefficients)

c4 <- as.data.frame(fullmodel4$coefficients)

c5 <- as.data.frame(fullmodel5$coefficients)

### Use fit of model 5 to use pooled coefficients

fullmodel5[["coefficients"]][["(Intercept)"]] <- -1.856772

fullmodel5[["coefficients"]][["male"]] <- 0.52284

fullmodel5[["coefficients"]][["y9_phys_speechproblem"]] <- 0.624926

fullmodel5[["coefficients"]][["y5_cantsitstill"]] <- 0.356006

fullmodel5[["coefficients"]][["y9_parent_cantconcentrate"]] <- 0.520474

fullmodel5[["coefficients"]][["y9_parent_restless"]] <- 0.866106

fullmodel5[["coefficients"]][["y9_parent_easydistracted"]] <- 0.19671

fullmodel5[["coefficients"]][["y9_teacher_anxingroupchildren"]] <- 0.485536

fullmodel5[["coefficients"]][["y9_teacher_angryeasily"]] <- 0.604378

fullmodel5[["coefficients"]][["y9_teacher_inattentive"]] <- 0.609822

fullmodel5[["coefficients"]][["y9_teacher_inattentive"]] <- 0.442572

##### Examine accuracy

holdoutimp1 <- read_sav("Data/imputations adhd/holdoutimp1.sav")

holdoutimp2 <- read_sav("Data/imputations adhd/holdoutimp2.sav")

holdoutimp3 <- read_sav("Data/imputations adhd/holdoutimp3.sav")

holdoutimp4 <- read_sav("Data/imputations adhd/holdoutimp4.sav")

holdoutimp5 <- read_sav("Data/imputations adhd/holdoutimp5.sav")

### Run model in all 5 imputed holdout dataset

glm_probs <- data.frame(probs = predict(fullmodel5, newdata = holdoutimp5,type="response"))

glm_pred <- glm_probs %>% mutate(pred = ifelse(probs>.5, "1", "0"))

glm_pred <- cbind(holdoutimp5, glm_pred)

glm_pred %>% count(pred, adhd) %>% spread(adhd, n, fill = 0)

#1

#Sensitivity

76/(76+40)*100 # 65.5%

#PPV

76/(129+76)*100 # 37.1%

#2

#Sensitivity

72/(72+44)*100 # 62.1%

#PPV

72/(120+72)*100 # 37.5%

#3

#Sensitivity

76/(76+40)*100 # 65.5%

#PPV

76/(124+76)*100 # 38.0%

#4

#Sensitivity

80/(80+36)*100 # 69.0%

#PPV

80/(122+80)*100 # 39.6%

#5

#Sensitivity

80/(80+36)*100 # 69.0%

#PPV

80/(134+80)*100 # 37.4%

### Pooled estimates

######################

### Final accuracy

######################

#Sensitivity

(65.5+62.1+65.5+69.0+69.0)/5 # 66.22%

#PPV

(37.1+37.5+38.0+39.6+37.4)/5 # 37.92%

##############################

##### Increase probability to 80%

##############################

### Run model in all 5 imputed holdout dataset

glm_probs <- data.frame(probs = predict(fullmodel5, newdata = holdoutimp5,type="response"))

glm_pred <- glm_probs %>% mutate(pred = ifelse(probs>.8, "1", "0"))

glm_pred <- cbind(holdoutimp5, glm_pred)

glm_pred %>% count(pred, adhd) %>% spread(adhd, n, fill = 0)

#1

#Sensitivity

30/(30+86)*100 # 25.9%

#PPV

30/(15+30)*100 # 66.7%

#2

#Sensitivity

24/(92+24)*100 # 20.7%

#PPV

24/(14+24)*100 # 63.2%

#3

#Sensitivity

25/(25+91)*100 # 21.6%

#PPV

25/(15+25)*100 # 62.5%

#4

#Sensitivity

27/(27+89)*100 # 23.3%

#PPV

27/(14+27)*100 # 65.9%

#5

#Sensitivity

29/(29+87)*100 # 25.0%

#PPV

29/(24+29)*100 # 54.7%

### Pooled estimates

######################

### Final accuracy

######################

#Sensitivity

(25.9+20.7+21.6+23.3+25.0)/5 # 23.3%

#PPV

(66.7+63.2+62.5+65.9+54.7)/5 # 62.6%
