# Supplementary material for: Developmental signs of ADHD and autism: a prospective investigation in 3623 children
Source: Eur Child Adolesc Psychiatry. 2022 Jun 24;32(10):1969–78. doi: 10.1007/s00787-022-02024-4 (PMC10533573; doi:10.1007/s00787-022-02024-4)
Supplement: Supplementary file 3 — Supplementary file3 (DOCX 72 KB) [file 787_2022_2024_MOESM3_ESM.docx]

library(dplyr)

library(ggplot2)

library(readxl)

library(summarytools)

library(gmodels)

library(pscl)

library(dominanceanalysis)

library(caret)

library(randomForest)

library(varImp)

library(logistf)

library(devtools)

library(VariableSelection)

library(olsrr)

library(glmnet)

library(ROSE)

library(rpart)

library(dplyr)

library(tidyr)

library(mice)

library(miceadds)

library(leaps)

library(bestglm)

setwd("/Users/matticervin/Dropbox/Artiklar/Fragile Families and Child Wellbeing Study")

options(scipen=999)

#### #### #### #### #### #### #### #### #### #### ####

#### Data for first stages of analysis

#### #### #### #### #### #### #### #### #### #### ####

neurodev_finalclear <- read_excel("Data/neurodev_finalclear.xlsx")

df <- subset(neurodev_finalclear, adhdinclude==1)

df2 <- subset(neurodev_finalclear, autisminclude==1)

### Cases of ADHD and ASD

freq(df$adhd)

# Freq % Valid % Valid Cum. % Total % Total Cum.

#----------- ------ --------- -------------- --------- --------------

# 0 2952 82.48 82.48 81.64 81.64

# 1 627 17.52 100.00 17.34 98.98

# Tot 3579 100.00 100.00 100.00 100.00

freq(df2$autism)

# Freq % Valid % Valid Cum. % Total % Total Cum.

#----------- ------ --------- -------------- --------- --------------

#0 2954 97.01 97.01 97.01 97.01

#1 91 2.99 100.00 2.99 100.00

#Tot 3045 100.00 100.00 100.00 100.00

# Create exploratory and holdout sets

## 20% vs 80% of the sample

##ADHD

smp_size <- floor(0.2 * nrow(df))

## set the seed to make partition reproducible

set.seed(1234)

twentyperc <- sample(seq_len(nrow(df)), size = smp_size)

holdout <- df[twentyperc, ]

expl <- df[-twentyperc, ]

### ASD

smp_size2 <- floor(0.2 * nrow(df2))

## set the seed to make partition reproducible

set.seed(1234)

twentyperc2 <- sample(seq_len(nrow(df2)), size = smp_size2)

holdout2 <- df2[twentyperc2, ]

expl2 <- df2[-twentyperc2, ]

freq(expl$adhd)

# Freq % Valid % Valid Cum. % Total % Total Cum.

#----------- ------ --------- -------------- --------- --------------

#0 2353 82.16 82.16 82.16 82.16

#1 511 17.84 100.00 17.84 100.00

freq(holdout$adhd)

# Freq % Valid % Valid Cum. % Total % Total Cum.

#----------- ------ --------- -------------- --------- --------------

#0 599 83.78 83.78 83.78 83.78

#1 116 16.22 100.00 16.22 100.00

freq(expl2$autism)

# Freq % Valid % Valid Cum. % Total % Total Cum.

#----------- ------ --------- -------------- --------- --------------

#0 2360 96.88 96.88 96.88 96.88

#1 76 3.12 100.00 3.12 100.00

freq(holdout2$autism)

# Freq % Valid % Valid Cum. % Total % Total Cum.

#----------- ------ --------- -------------- --------- --------------

#0 594 97.54 97.54 97.54 97.54

#1 15 2.46 100.00 2.46 100.00

####################################

######## Comparisons

####################################

CrossTable(expl2$autism, expl2$male)

### Early predictors

rpregnancy_drugs <- logistf(autism ~ male + pregnancy_drugs, data = expl2)

rpregnancy_drink <- logistf(autism ~ male + pregnancy_drink, data = expl2)

rpregnancy_smoke <- logistf(autism ~ male + pregnancy_smoke, data = expl2)

rbaseline_lowbirthweight <- logistf(autism ~ male + baseline_lowbirthweight, data = expl2)

rnotwalk14mnths <- logistf(autism ~ male + notwalk14mnths, data = expl2)

rbaseline_mothersage <- logistf(autism ~ male + baseline_mothersage, data = expl2)

rbaseline_fatherinjail <- logistf(autism ~ male + baseline_fatherinjail, data = expl2)

summary(rpregnancy_drugs)

summary(rpregnancy_drink)

summary(rpregnancy_smoke)

summary(rbaseline_lowbirthweight)

summary(rnotwalk14mnths)

summary(rbaseline_mothersage)

summary(rbaseline_fatherinjail)

### Psychiatric problems in family (grandmothers and grandfathers)

rfampsych_dep_fside <- logistf(autism ~ male + fampsych_dep_fside, data = expl2)

rfampsych_anx_fside <- logistf(autism ~ male + fampsych_anx_fside, data = expl2)

rfampsych_substance_fside <- logistf(autism ~ male + fampsych_substance_fside, data = expl2)

rfampsych_sucatt_fside <- logistf(autism ~ male + fampsych_sucatt_fside, data = expl2)

rfampsych_dep_mside <- logistf(autism ~ male + fampsych_dep_mside, data = expl2)

rfampsych_anx_mside <- logistf(autism ~ male + fampsych_anx_mside, data = expl2)

rfampsych_substance_mside <- logistf(autism ~ male + fampsych_substance_mside, data = expl2)

rfampsych_sucatt_mside <- logistf(autism ~ male + fampsych_sucatt_mside, data = expl2)

summary(rfampsych_dep_fside)#*

summary(rfampsych_anx_fside)

summary(rfampsych_substance_fside)

summary(rfampsych_sucatt_fside)

summary(rfampsych_dep_mside)#*

summary(rfampsych_anx_mside)#***

summary(rfampsych_substance_mside)

summary(rfampsych_sucatt_mside)

CrossTable(expl2$autism, expl2$fampsych_anx_mside)

### Psychiatric problems in parents over time

ry1_mother_anx <- logistf(autism ~ male + y1_mother_anx, data = expl2)

ry1_mother_dep <- logistf(autism ~ male + y1_mother_dep, data = expl2)

ry1_father_anx <- logistf(autism ~ male + y1_father_anx, data = expl2)

ry1_father_dep <- logistf(autism ~ male + y1_father_dep, data = expl2)

ry3_father_substuse <- logistf(autism ~ male + y3_father_substuse, data = expl2)

ry3_mother_substuse <- logistf(autism ~ male + y3_mother_substuse, data = expl2)

ry3_father_anx <- logistf(autism ~ male + y3_father_anx, data = expl2)

ry3_father_dep <- logistf(autism ~ male + y3_father_dep, data = expl2)

ry3_mother_anx <- logistf(autism ~ male + y3_mother_anx, data = expl2)

ry3_mother_dep <- logistf(autism ~ male + y3_mother_dep, data = expl2)

ry5_mother_dep <- logistf(autism ~ male + y5_mother_dep, data = expl2)

ry5_father_dep <- logistf(autism ~ male + y5_father_dep, data = expl2)

summary(ry1_mother_anx)#*

summary(ry1_mother_dep)#***

summary(ry1_father_anx)

summary(ry1_father_dep)

summary(ry3_father_substuse)#*

summary(ry3_mother_substuse)

summary(ry3_father_anx)

summary(ry3_father_dep)

summary(ry3_mother_anx)

summary(ry3_mother_dep)#*

summary(ry5_mother_dep)#*

summary(ry5_father_dep)

CrossTable(expl2$autism, expl2$y1_mother_dep)

### Physical health year 1

ry1_asthma <- logistf(autism ~ male + y1_asthma, data = expl2)

ry1_emergroom_accident <- logistf(autism ~ male + y1_emergroom_accident, data = expl2)

ry1_top10perchlthcvisits <- logistf(autism ~ male + y1_top10perchlthcvisits, data = expl2)

ry1_overnighthosp <- logistf(autism ~ male + y1_overnighthosp, data = expl2)

summary(ry1_asthma)

summary(ry1_emergroom_accident)

summary(ry1_top10perchlthcvisits)#*

summary(ry1_overnighthosp)#**

CrossTable(expl2$autism, expl2$y1_overnighthosp)

### Physical health year 3

ry3_healthfairpoor <- logistf(autism ~ male + y3_healthfairpoor, data = expl2)

ry3_asthma <- logistf(autism ~ male + y3_asthma, data = expl2)

ry3_hlthcarevisitstop10perc <- logistf(autism ~ male + y3_hlthcarevisitstop10perc, data = expl2)

ry3_emergroom_accident <- logistf(autism ~ male + y3_emergroom_accident, data = expl2)

ry3_overnighthosp <- logistf(autism ~ male + y3_overnighthosp, data = expl2)

ry3_emergvisittop10perc2ormore <- logistf(autism ~ male + y3_emergvisittop10perc2ormore, data = expl2)

ry3_physdisability <- logistf(autism ~ male + y3_physdisability, data = expl2)

ry3_speechprblm <- logistf(autism ~ male + y3_speechprblm, data = df)

ry5_speechproblem<-logistf(autism~male+y5_speechproblem, data=expl2, family="binomial")

summary(ry3_healthfairpoor)

summary(ry3_asthma)

summary(ry3_hlthcarevisitstop10perc)

summary(ry3_emergroom_accident)

summary(ry3_overnighthosp)

summary(ry3_emergvisittop10perc2ormore)

summary(ry3_physdisability)#***

summary(ry3_speechprblm)#***

CrossTable(expl2$autism, expl2$y3_physdisability)

CrossTable(expl2$autism, expl2$y3_speechprblm)

### Physical health year 5

ry5_hlthcarevisitstop10perc <- logistf(autism ~ male + y5_hlthcarevisitstop10perc, data = expl2)

ry5_ervisityes <- logistf(autism ~ male + y5_ervisityes, data = expl2)

ry5_overnightstayhospitalyes <- logistf(autism ~ male + y5_overnightstayhospitalyes, data = expl2)

ry5_er_accident_yes <- logistf(autism ~ male + y5_er_accident_yes, data = expl2)

ry5_over2earinf <- logistf(autism ~ male + y5_over2earinf, data = expl2)

ry5_speechproblem<-logistf(autism~male+y5_speechproblem, data=expl2, family="binomial")

summary(ry5_hlthcarevisitstop10perc)#*

summary(ry5_ervisityes)

summary(ry5_overnightstayhospitalyes)

summary(ry5_er_accident_yes)

summary(ry5_over2earinf)#***

summary(ry5_speechproblem)#***

CrossTable(expl2$autism, expl2$y5_over2earinf)

CrossTable(expl2$autism, expl2$y5_speechproblem)

### Physical health year 9

ry9_phys_poorhealth <- logistf(autism ~ male + y9_phys_poorhealth, data = expl2)

ry9_phys_wheezingchest <- logistf(autism ~ male + y9_phys_wheezingchest, data = expl2)

ry9_phys_asthma <- logistf(autism ~ male + y9_phys_asthma, data = expl2)

ry9_phys_speechproblem <- logistf(autism ~ male + y9_phys_speechproblem, data = expl2)

ry9_phys_respallergy <- logistf(autism ~ male + y9_phys_respallergy, data = expl2)

ry9_phys_foodallergy <- logistf(autism ~ male + y9_phys_foodallergy, data = expl2)

ry9_phys_eczema <- logistf(autism ~ male + y9_phys_eczema, data = expl2)

ry9_phys_diarrhea <- logistf(autism ~ male + y9_phys_diarrhea, data = expl2)

ry9_phys_anemia <- logistf(autism ~ male + y9_phys_anemia, data = expl2)

ry9_phys_freqheadaches <- logistf(autism ~ male + y9_phys_freqheadaches, data = expl2)

ry9_phys_seizures <- logistf(autism ~ male + y9_phys_seizures, data = expl2)

ry9_phys_stuttering <- logistf(autism ~ male + y9_phys_stuttering, data = expl2)

ry9_phys_diabetes <- logistf(autism ~ male + y9_phys_diabetes, data = expl2)

ry9_over2earinf <- logistf(autism ~ male + y9_over2earinf, data = expl2)

ry9_parent_overeats <- logistf(autism ~ male + y9_parent_overeats, data = expl2)

y9_parent_overtired <- logistf(autism ~ male + y9_parent_overtired, data = expl2)

ry9_parent_overweight <- logistf(autism ~ male + y9_parent_overweight, data = expl2)

ry9_parent_unknownphys_pains <- logistf(autism ~ male + y9_parent_unknownphys_pains, data = expl2)

ry9_parent_unknownphys_headaches <- logistf(autism ~ male + y9_parent_unknownphys_headaches, data = expl2)

ry9_parent_unknownphys_nausea <- logistf(autism ~ male + y9_parent_unknownphys_nausea, data = expl2)

ry9_parent_unknownphys_eyes <- logistf(autism ~ male + y9_parent_unknownphys_eyes, data = expl2)

ry9_parent_unknownphys_skin <- logistf(autism ~ male + y9_parent_unknownphys_skin, data = expl2)

ry9_parent_unknownphys_vomit <- logistf(autism ~ male + y9_parent_unknownphys_vomit, data = expl2)

ry9_parent_unknownphys_other <- logistf(autism ~ male + y9_parent_unknownphys_other, data = expl2)

ry9_parent_unknownphys_cramps <- logistf(autism ~ male + y9_parent_unknownphys_cramps, data = expl2)

ry9_weight_percentile <- logistf(autism ~ male + y9_weight_percentile, data = expl2)

ry9_height_percentile <- logistf(autism ~ male + y9_height_percentile, data = expl2)

ry9_parent_constipated <-logistf(autism~male+y9_parent_constipated,data=expl2,family="binomial")

summary(ry9_phys_poorhealth)

summary(ry9_phys_wheezingchest)

summary(ry9_phys_asthma)

summary(ry9_phys_speechproblem)#***

summary(ry9_phys_respallergy)

summary(ry9_phys_foodallergy)

summary(ry9_phys_eczema)#*

summary(ry9_phys_diarrhea)

summary(ry9_phys_anemia)

summary(ry9_phys_freqheadaches)

summary(ry9_phys_seizures)#*

summary(ry9_phys_stuttering)#**

summary(ry9_phys_diabetes)

summary(ry9_over2earinf)#**

summary(ry9_parent_overeats)

summary(y9_parent_overtired)

summary(ry9_parent_overweight)

summary(ry9_parent_unknownphys_pains)#**

summary(ry9_parent_unknownphys_headaches)

summary(ry9_parent_unknownphys_nausea)

summary(ry9_parent_unknownphys_eyes)#*

summary(ry9_parent_unknownphys_skin)#*

summary(ry9_parent_unknownphys_vomit)#*

summary(ry9_parent_unknownphys_other)

summary(ry9_parent_unknownphys_cramps)#*

summary(ry9_weight_percentile)

summary(ry9_height_percentile)#**

summary(ry9_parent_constipated)#***

CrossTable(expl2$autism, expl2$y9_phys_speechproblem)

CrossTable(expl2$autism, expl2$y9_phys_stuttering)

CrossTable(expl2$autism, expl2$y9_over2earinf)

CrossTable(expl2$autism, expl2$y9_parent_unknownphys_pains)

CrossTable(expl2$autism, expl2$y9_parent_constipated)

aggregate(y9_height_percentile ~ autism, expl, mean)

aggregate(y9_height_percentile ~ autism, expl, sd)

### Year 1 Child Factors

ry1_shy <- logistf(autism ~ male + y1_shy, data = expl2)

ry1_fusscry <- logistf(autism ~ male + y1_fusscry, data = expl2)

ry1_social <- logistf(autism ~ male + y1_social, data = expl2)

ry1_upseteasily <- logistf(autism ~ male + y1_upseteasily, data = expl2)

ry1_strongreact <- logistf(autism ~ male + y1_strongreact, data = expl2)

ry1_shystrangers <- logistf(autism ~ male + y1_shystrangers, data = expl2)

summary(ry1_shy)

summary(ry1_fusscry)

summary(ry1_social)

summary(ry1_upseteasily)

summary(ry1_strongreact)

summary(ry1_shystrangers)

### Year 3 Factors

ry3_attachment_securevsnot <- logistf(autism ~ male + y3_attachment_securevsnot, data = expl2)

ry3_symphatothers <- logistf(autism ~ male + y3_symphatothers, data = expl2)

ry3_understothersfeelings <- logistf(autism ~ male + y3_understothersfeelings, data = expl2)

ry3_openneeds <- logistf(autism ~ male + y3_openneeds, data = expl2)

ry3_joinotherchildren <- logistf(autism ~ male + y3_joinotherchildren, data = expl2)

ry3_playswithothers <- logistf(autism ~ male + y3_playswithothers, data = expl2)

ry3_interestinthings <- logistf(autism ~ male + y3_interestinthings, data = expl2)

ry3_confidentothers <- logistf(autism ~ male + y3_confidentothers, data = expl2)

ry3_proud <- logistf(autism ~ male + y3_proud, data = expl2)

ry3_manyinterests <- logistf(autism ~ male + y3_manyinterests, data = expl2)

summary(ry3_attachment_securevsnot)#**

summary(ry3_symphatothers)

summary(ry3_understothersfeelings)

summary(ry3_openneeds)

summary(ry3_joinotherchildren)#*

summary(ry3_playswithothers)#**

summary(ry3_interestinthings)

summary(ry3_confidentothers)

summary(ry3_proud)

summary(ry3_manyinterests)

CrossTable(expl2$autism, expl2$y3_attachment_securevsnot)

CrossTable(expl2$autism, expl2$y3_playswithothers)

ry3_enjpoystalkparent <- logistf(autism ~ male + y3_enjpoystalkparent, data = expl2)

ry3_tooyoung <- logistf(autism ~ male + y3_tooyoung, data = expl2)

ry3_pooreyecont <- logistf(autism ~ male + y3_pooreyecont, data = expl2)

ry3_cantconcentr <- logistf(autism ~ male + y3_cantconcentr, data = expl2)

ry3_cantsitstill <- logistf(autism ~ male + y3_cantsitstill, data = expl2)

ry3_cantwait <- logistf(autism ~ male + y3_cantwait, data = expl2)

ry3_clingadults <- logistf(autism ~ male + y3_clingadults, data = expl2)

ry3_criesalot <- logistf(autism ~ male + y3_criesalot, data = expl2)

ry3_cruelanimals <- logistf(autism ~ male + y3_cruelanimals, data = expl2)

ry3_defiant <- logistf(autism ~ male + y3_defiant, data = expl2)

summary(ry3_enjpoystalkparent)#**

summary(ry3_tooyoung)#***

summary(ry3_pooreyecont)#***

summary(ry3_cantconcentr)#***

summary(ry3_cantsitstill)#***

summary(ry3_cantwait)#***

summary(ry3_clingadults)

summary(ry3_criesalot)#*

summary(ry3_cruelanimals)

summary(ry3_defiant)#***

CrossTable(expl2$autism, expl2$y3_enjpoystalkparent)

CrossTable(expl2$autism, expl2$y3_tooyoung)

CrossTable(expl2$autism, expl2$y3_pooreyecont)

CrossTable(expl2$autism, expl2$y3_cantconcentr)

CrossTable(expl2$autism, expl2$y3_cantsitstill)

CrossTable(expl2$autism, expl2$y3_cantwait)

CrossTable(expl2$autism, expl2$y3_defiant)

ry3_demandsmetdirect <- logistf(autism ~ male + y3_demandsmetdirect, data = expl2)

ry3_destroyownthngs <- logistf(autism ~ male + y3_destroyownthngs, data = expl2)

ry3_destroyothersthngs <- logistf(autism ~ male + y3_destroyothersthngs, data = expl2)

ry3_disobed <- logistf(autism ~ male + y3_disobed, data = expl2)

ry3_distrbdchroutine <- logistf(autism ~ male + y3_distrbdchroutine, data = expl2)

ry3_dontrespondtalk <- logistf(autism ~ male + y3_dontrespondtalk, data = expl2)

ry3_notgetalongchildren <- logistf(autism ~ male + y3_notgetalongchildren, data = expl2)

ry3_actslikeadult <- logistf(autism ~ male + y3_actslikeadult, data = expl2)

ry3_noguilt <- logistf(autism ~ male + y3_noguilt, data = expl2)

ry3_easilyfrustr <- logistf(autism ~ male + y3_easilyfrustr, data = expl2)

summary(ry3_demandsmetdirect)#***

summary(ry3_destroyownthngs)#**

summary(ry3_destroyothersthngs)#*

summary(ry3_disobed)#**

summary(ry3_distrbdchroutine)#***

summary(ry3_dontrespondtalk)#**

summary(ry3_notgetalongchildren)#*

summary(ry3_actslikeadult)

summary(ry3_noguilt)#***

summary(ry3_easilyfrustr)#***

CrossTable(expl2$autism, expl2$y3_demandsmetdirect)

CrossTable(expl2$autism, expl2$y3_destroyownthngs)

CrossTable(expl2$autism, expl2$y3_destroyothersthngs)

CrossTable(expl2$autism, expl2$y3_disobed)

CrossTable(expl2$autism, expl2$y3_distrbdchroutine)

CrossTable(expl2$autism, expl2$y3_dontrespondtalk)

CrossTable(expl2$autism, expl2$y3_noguilt)

CrossTable(expl2$autism, expl2$y3_easilyfrustr)

ry3_flngseasyilyhurt <- logistf(autism ~ male + y3_flngseasyilyhurt, data = expl2)

ry3_jealous <- logistf(autism ~ male + y3_jealous, data = expl2)

ry3_accidentprone <- logistf(autism ~ male + y3_accidentprone, data = expl2)

ry3_fights <- logistf(autism ~ male + y3_fights, data = expl2)

ry3_sleepproblems <- logistf(autism ~ male + y3_sleepproblems, data = expl2)

ry3_upsetseparation <- logistf(autism ~ male + y3_upsetseparation, data = expl2)

ry3_hitsothers <- logistf(autism ~ male + y3_hitsothers, data = expl2)

ry3_hurtothersunintent <- logistf(autism ~ male + y3_hurtothersunintent, data = expl2)

ry3_unhappynoreason <- logistf(autism ~ male + y3_unhappynoreason, data = expl2)

ry3_angrymoods <- logistf(autism ~ male + y3_angrymoods, data = expl2)

summary(ry3_flngseasyilyhurt)

summary(ry3_jealous)

summary(ry3_accidentprone)#*

summary(ry3_fights)

summary(ry3_sleepproblems)

summary(ry3_upsetseparation)

summary(ry3_hitsothers)#*

summary(ry3_hurtothersunintent)

summary(ry3_unhappynoreason)#*

summary(ry3_angrymoods)#**

CrossTable(expl2$autism, expl2$y3_angrymoods)

ry3_nervous <- logistf(autism ~ male + y3_nervous, data = expl2)

ry3_overtired <- logistf(autism ~ male + y3_overtired, data = expl2)

ry3_physicallyattacks <- logistf(autism ~ male + y3_physicallyattacks, data = expl2)

ry3_notsenstowpunish <- logistf(autism ~ male + y3_notsenstowpunish, data = expl2)

ry3_shiftactivity <- logistf(autism ~ male + y3_shiftactivity, data = expl2)

ry3_refuseplaygames <- logistf(autism ~ male + y3_refuseplaygames, data = expl2)

ry3_screams <- logistf(autism ~ male + y3_screams, data = expl2)

ry3_unresponsaffection <- logistf(autism ~ male + y3_unresponsaffection, data = expl2)

ry3_easyembarassed <- logistf(autism ~ male + y3_easyembarassed, data = expl2)

ry3_selfish <- logistf(autism ~ male + y3_selfish, data = expl2)

summary(ry3_nervous)#***

summary(ry3_overtired)#***

summary(ry3_physicallyattacks)#**

summary(ry3_notsenstowpunish)#***

summary(ry3_shiftactivity)

summary(ry3_refuseplaygames)

summary(ry3_screams)#*

summary(ry3_unresponsaffection)

summary(ry3_easyembarassed)

summary(ry3_selfish)#*

CrossTable(expl2$autism, expl2$y3_nervous)

CrossTable(expl2$autism, expl2$y3_overtired)

CrossTable(expl2$autism, expl2$y3_physicallyattacks)

CrossTable(expl2$autism, expl2$y3_notsenstowpunish)

ry3_littleaffctothers <- logistf(autism ~ male + y3_littleaffctothers, data = expl2)

ry3_lttleinterestthings <- logistf(autism ~ male + y3_lttleinterestthings, data = expl2)

ry3_shy <- logistf(autism ~ male + y3_shy, data = expl2)

ry3_stubborn <- logistf(autism ~ male + y3_stubborn, data = expl2)

ry3_moodswings <- logistf(autism ~ male + y3_moodswings, data = expl2)

ry3_sulks <- logistf(autism ~ male + y3_sulks, data = expl2)

ry3_tempertantrums <- logistf(autism ~ male + y3_tempertantrums, data = expl2)

ry3_tooanxious <- logistf(autism ~ male + y3_tooanxious, data = expl2)

ry3_uncooperative <- logistf(autism ~ male + y3_uncooperative, data = expl2)

ry3_underactive <- logistf(autism ~ male + y3_underactive, data = expl2)

ry3_sad <- logistf(autism ~ male + y3_sad, data = expl2)

ry3_loud <- logistf(autism ~ male + y3_loud, data = expl2)

ry3_attentionseeking <- logistf(autism ~ male + y3_attentionseeking, data = expl2)

ry3_whiny <- logistf(autism ~ male + y3_whiny, data = expl2)

ry3_withdrawn <- logistf(autism ~ male + y3_withdrawn, data = expl2)

summary(ry3_littleaffctothers)

summary(ry3_lttleinterestthings)

summary(ry3_shy)

summary(ry3_stubborn)

summary(ry3_moodswings)

summary(ry3_sulks)#*

summary(ry3_tempertantrums)#***

summary(ry3_tooanxious)

summary(ry3_uncooperative)#*

summary(ry3_underactive)

summary(ry3_sad)

summary(ry3_loud)

summary(ry3_attentionseeking)

summary(ry3_whiny)

summary(ry3_withdrawn)#**

CrossTable(expl2$autism, expl2$y3_tempertantrums)

CrossTable(expl2$autism, expl2$y3_withdrawn)

## Early factors and family history of psych disorders

# Create evenly distributed synthetic data

data.rose <- ROSE(autism ~ fampsych_anx_mside+y1_mother_dep+y1_overnighthosp+y3_physdisability+y3_speechprblm+y5_over2earinf+y5_speechproblem+

y9_phys_speechproblem+y9_phys_stuttering+y9_over2earinf+y9_parent_unknownphys_pains+y9_parent_constipated,

data = expl2, seed = 1)$data

form <- glm(autism ~ fampsych_anx_mside+y1_mother_dep+y1_overnighthosp+y3_physdisability+y3_speechprblm+y5_over2earinf+y5_speechproblem+

y9_phys_speechproblem+y9_phys_stuttering+y9_over2earinf+y9_parent_unknownphys_pains+y9_parent_constipated, data=data.rose,

family="binomial")

# Run bestglm to find best subset model. Use syntehetic data to get better coefficients to find cases

lowbwt.bglm <- data.rose[, c("fampsych_anx_mside","y1_mother_dep","y1_overnighthosp","y3_physdisability","y3_speechprblm","y5_over2earinf","y5_speechproblem",

"y9_phys_speechproblem","y9_phys_stuttering","y9_over2earinf","y9_parent_unknownphys_pains","y9_parent_constipated","autism")]

names(lowbwt.bglm)[names(lowbwt.bglm) == "autism"] <- "y"

lowbwt.bglm <- as.data.frame(lowbwt.bglm)

best.logit <- bestglm(lowbwt.bglm,

IC = "AIC",

family=binomial,

method = "exhaustive")

summary(best.logit$BestModel)

glm_probs <- data.frame(probs = predict(form, newdata = asdimpholdout1,type="response"))

glm_pred <- glm_probs %>% mutate(pred = ifelse(probs>.5, "1", "0"))

glm_pred <- cbind(asdimpholdout1, glm_pred)

glm_pred %>% count(pred, autism) %>% spread(autism, n, fill = 0)

#pred 0 1

#1 0 2471 21

#2 1 483 70

#Sensitivity

70/(70+21)*100 # 76.9%

#PPV

70/(483+70)*100 # 12.7%

# And so on, mean Sens = 76%, mean PPV = 13%

#### Year 3 factors

# Create evenly distributed synthetic data

data.rose <- ROSE(autism ~ y3_playswithothers+y3_enjpoystalkparent+y3_tooyoung+y3_pooreyecont+y3_cantconcentr+y3_cantsitstill+y3_cantwait+y3_defiant+

y3_demandsmetdirect+y3_destroyownthngs+y3_destroyothersthngs+y3_disobed,

data = expl2, seed = 1)$data

# Run bestglm to find best subset model. Use syntehetic data to get better coefficients to find cases

lowbwt.bglm <- data.rose[, c("y3_playswithothers","y3_enjpoystalkparent","y3_tooyoung","y3_pooreyecont","y3_cantconcentr","y3_cantsitstill","y3_cantwait","y3_defiant",

"y3_demandsmetdirect","y3_destroyownthngs","y3_destroyothersthngs","y3_disobed","autism")]

names(lowbwt.bglm)[names(lowbwt.bglm) == "autism"] <- "y"

lowbwt.bglm <- as.data.frame(lowbwt.bglm)

best.logit <- bestglm(lowbwt.bglm,

IC = "AIC",

family=binomial,

method = "exhaustive")

summary(best.logit$BestModel)

#y3_playswithothers -0.458 0.112 -4.10 0.000040439 ***

#y3_enjpoystalkparent -0.333 0.139 -2.39 0.01683 *

#y3_tooyoung 1.529 0.250 6.11 0.000000001 ***

#y3_cantconcentr 1.261 0.152 8.31 < 0.0000000000000002 ***

#y3_cantsitstill 0.167 0.117 1.42 0.15468

#y3_cantwait 0.194 0.108 1.78 0.07429 .

#y3_defiant 0.429 0.129 3.32 0.00090 ***

#y3_demandsmetdirect 0.338 0.118 2.86 0.00420 **

#y3_disobed 0.594 0.180 3.29 0.00099 ***

# Create evenly distributed synthetic data

data.rose <- ROSE(autism ~ y3_distrbdchroutine+y3_dontrespondtalk+y3_noguilt+y3_easilyfrustr+

y3_angrymoods+y3_nervous+y3_overtired+y3_physicallyattacks+

y3_notsenstowpunish+y3_tempertantrums+y3_withdrawn,

data = expl2, seed = 1)$data

# Run bestglm to find best subset model. Use syntehetic data to get better coefficients to find cases

lowbwt.bglm <- data.rose[, c("y3_distrbdchroutine","y3_dontrespondtalk","y3_noguilt","y3_easilyfrustr",

"y3_angrymoods","y3_nervous","y3_overtired","y3_physicallyattacks",

"y3_notsenstowpunish","y3_tempertantrums","y3_withdrawn","autism")]

names(lowbwt.bglm)[names(lowbwt.bglm) == "autism"] <- "y"

lowbwt.bglm <- as.data.frame(lowbwt.bglm)

best.logit <- bestglm(lowbwt.bglm,

IC = "AIC",

family=binomial,

method = "exhaustive")

summary(best.logit$BestModel)

#y3_distrbdchroutine 0.8882 0.1558 5.70 0.000000012 ***

#y3_dontrespondtalk 0.3969 0.1618 2.45 0.01415 *

#y3_noguilt 0.5736 0.1250 4.59 0.000004431 ***

#y3_easilyfrustr 0.4793 0.1245 3.85 0.00012 ***

#y3_overtired 1.1799 0.2136 5.52 0.000000033 ***

#y3_notsenstowpunish 0.4110 0.1412 2.91 0.00361 **

#y3_tempertantrums 0.3161 0.1333 2.37 0.01775 *

#y3_withdrawn 0.9653 0.2352 4.10 0.000040687 ***

# Examine accuracy in imputed holdout sample (because so few cases) - run imputations across same lines, so all 5 not shown

data.rose <- ROSE(autism ~ y3_playswithothers+y3_tooyoung+y3_cantconcentr+y3_defiant+y3_demandsmetdirect+y3_disobed+

y3_distrbdchroutine+y3_noguilt+y3_easilyfrustr+y3_overtired+y3_notsenstowpunish+y3_withdrawn,

data = expl2, seed = 1)$data

# Run bestglm to find best subset model. Use syntehetic data to get better coefficients to find cases

lowbwt.bglm <- data.rose[, c("y3_playswithothers","y3_tooyoung","y3_cantconcentr","y3_defiant",

"y3_demandsmetdirect","y3_disobed","y3_distrbdchroutine","y3_noguilt",

"y3_easilyfrustr","y3_overtired","y3_notsenstowpunish","y3_withdrawn", "autism")]

names(lowbwt.bglm)[names(lowbwt.bglm) == "autism"] <- "y"

lowbwt.bglm <- as.data.frame(lowbwt.bglm)

best.logit <- bestglm(lowbwt.bglm,

IC = "AIC",

family=binomial,

method = "exhaustive")

summary(best.logit$BestModel)

#y3_playswithothers -0.604 0.113 -5.35 0.00000008672468287 ***

#y3_tooyoung 0.786 0.236 3.33 0.00088 ***

#y3_cantconcentr 1.202 0.148 8.15 0.00000000000000037 ***

#y3_defiant 0.347 0.133 2.61 0.00913 **

#y3_demandsmetdirect 0.454 0.113 4.03 0.00005553789120443 ***

#y3_distrbdchroutine 0.926 0.178 5.20 0.00000020278467963 ***

#y3_noguilt 0.512 0.136 3.77 0.00016 ***

#y3_overtired 1.264 0.234 5.40 0.00000006601533863 ***

#y3_withdrawn 0.911 0.252 3.61 0.00030 ***

library(haven)

asdimpholdout1 <- read_sav("Data/imputations asd/asdimpholdout1.sav")

asdimpholdout2 <- read_sav("Data/imputations asd/asdimpholdout2.sav")

asdimpholdout3 <- read_sav("Data/imputations asd/asdimpholdout3.sav")

asdimpholdout4 <- read_sav("Data/imputations asd/asdimpholdout4.sav")

asdimpholdout5 <- read_sav("Data/imputations asd/asdimpholdout5.sav")

glm_probs <- data.frame(probs = predict(best.logit$BestModel, newdata = asdimpholdout5,type="response"))

glm_pred <- glm_probs %>% mutate(pred = ifelse(probs>.5, "1", "0"))

glm_pred <- cbind(asdimpholdout5, glm_pred)

glm_pred %>% count(pred, autism) %>% spread(autism, n, fill = 0)

# Imp 1

# pred 0 1

#1 0 2194 28

#2 1 760 63

#Sensitivity

63/(63+28)*100 # 69.2%

#PPV

63/(760+63)*100 # 7.7%

# Imp 2

# pred 0 1

#1 0 2188 30

#2 1 766 61

#Sensitivity

61/(61+30)*100 # 67.0%

#PPV

61/(766+61)*100 # 7.4%

# Imp 3

# pred 0 1

#1 0 2203 26

#2 1 751 65

#Sensitivity

65/(65+26)*100 # 71.4%

#PPV

65/(751+65)*100 # 8.0%

# Imp 4

# pred 0 1

#1 0 2205 31

#2 1 749 60

#Sensitivity

60/(60+31)*100 # 65.9%

#PPV

60/(749+60)*100 # 7.4%

# Imp 5

# pred 0 1

#1 0 2172 29

#2 1 782 62

#Sensitivity

62/(62+29)*100 # 68.1%

#PPV

62/(782+62)*100 # 7.3%

#Pooled sensitivity

(69.2+67+71.4+65.9+68.1)/5 ## 68.3%

#Pooled PPV

(7.7+7.4+8+7.4+7.3)/5 ## 7.6%

### ### ### ### ### ### ### ###

### Year 5 factors

### ### ### ### ### ### ### ###

ry5_under7hoursofsleep <-logistf(autism~male+y5_under7hoursofsleep, data=expl2)

ry5_sleepinparentsbed<-logistf(autism~male+y5_sleepinparentsbed, data=expl2)

ry5_argues<-logistf(autism~male+y5_argues, data=expl2)

ry5_brags<-logistf(autism~male+y5_brags, data=expl2)

ry5_undstotherfeelings<-logistf(autism~male+y5_undstotherfeelings, data=expl2)

ry5_overeats<-logistf(autism~male+y5_overeats, data=expl2)

ry5_loneliness<-logistf(autism~male+y5_loneliness, data=expl2)

ry5_confused<-logistf(autism~male+y5_confused, data=expl2)

ry5_cruelothers<-logistf(autism~male+y5_cruelothers, data=expl2)

ry5_daydreams<-logistf(autism~male+y5_daydreams, data=expl2)

ry5_destroyownthings<-logistf(autism~male+y5_destroyownthings, data=expl2)

ry5_destroyotherthings<-logistf(autism~male+y5_destroyotherthings, data=expl2)

ry5_sympathyothers<-logistf(autism~male+y5_sympathyothers, data=expl2)

ry5_disobedienthome<-logistf(autism~male+y5_disobedienthome, data=expl2)

ry5_disobedientschool<-logistf(autism~male+y5_disobedientschool, data=expl2)

ry5_accidentprone<-logistf(autism~male+y5_accidentprone, data=expl2)

ry5_openwhatwant<-logistf(autism~male+y5_openwhatwant, data=expl2)

ry5_jealous<-logistf(autism~male+y5_jealous, data=expl2)

ry5_dothinkbadobsession<-logistf(autism~male+y5_dothinkbadobsession, data=expl2)

ry5_hastobeperfect<-logistf(autism~male+y5_hastobeperfect, data=expl2)

ry5_complainsnoloveshim<-logistf(autism~male+y5_complainsnoloveshim, data=expl2)

ry5_otheroutotget<-logistf(autism~male+y5_otheroutotget, data=expl2)

ry5_getintofights<-logistf(autism~male+y5_getintofights, data=expl2)

ry5_getteased<-logistf(autism~male+y5_getteased, data=expl2)

ry5_hangswotherstrouble<-logistf(autism~male+y5_hangswotherstrouble, data=expl2)

ry5_impulsive<-logistf(autism~male+y5_impulsive, data=expl2)

ry5_ratherbealone<-logistf(autism~male+y5_ratherbealone, data=expl2)

ry5_liescheats<-logistf(autism~male+y5_liescheats, data=expl2)

ry5_nervoustwitches<-logistf(autism~male+y5_nervoustwitches, data=expl2)

ry5_notlikedbyothers<-logistf(autism~male+y5_notlikedbyothers, data=expl2)

ry5_feelstooguilty<-logistf(autism~male+y5_feelstooguilty, data=expl2)

ry5_joinsothersplay<-logistf(autism~male+y5_joinsothersplay, data=expl2)

ry5_overweight<-logistf(autism~male+y5_overweight, data=expl2)

ry5_justwatchothers<-logistf(autism~male+y5_justwatchothers, data=expl2)

ry5_attackspeople<-logistf(autism~male+y5_attackspeople, data=expl2)

ry5_poorschoolwork<-logistf(autism~male+y5_poorschoolwork, data=expl2)

ry5_clumsy<-logistf(autism~male+y5_clumsy, data=expl2)

ry5_preferolderkids<-logistf(autism~male+y5_preferolderkids, data=expl2)

ry5_preferyoungerkids<-logistf(autism~male+y5_preferyoungerkids, data=expl2)

ry5_refusestotalk<-logistf(autism~male+y5_refusestotalk, data=expl2)

ry5_runsawayfromhome<-logistf(autism~male+y5_runsawayfromhome, data=expl2)

ry5_screamsalot<-logistf(autism~male+y5_screamsalot, data=expl2)

ry5_attentionfromothers<-logistf(autism~male+y5_attentionfromothers, data=expl2)

ry5_secretive<-logistf(autism~male+y5_secretive, data=expl2)

ry5_easilyembarassed<-logistf(autism~male+y5_easilyembarassed, data=expl2)

ry5_setsfires<-logistf(autism~male+y5_setsfires, data=expl2)

ry5_clownsaround<-logistf(autism~male+y5_clownsaround, data=expl2)

ry5_shy<-logistf(autism~male+y5_shy, data=expl2)

ry5_staresblankly<-logistf(autism~male+y5_staresblankly, data=expl2)

ry5_saysthanksplease<-logistf(autism~male+y5_saysthanksplease, data=expl2)

ry5_stealshome<-logistf(autism~male+y5_stealshome, data=expl2)

ry5_stealsoutsidehome<-logistf(autism~male+y5_stealsoutsidehome, data=expl2)

ry5_wanttoplayothers<-logistf(autism~male+y5_wanttoplayothers, data=expl2)

ry5_sulks<-logistf(autism~male+y5_sulks, data=expl2)

ry5_suspicious<-logistf(autism~male+y5_suspicious, data=expl2)

ry5_swears<-logistf(autism~male+y5_swears, data=expl2)

ry5_playstalkothers<-logistf(autism~male+y5_playstalkothers, data=expl2)

ry5_talkstoomuch<-logistf(autism~male+y5_talkstoomuch, data=expl2)

ry5_teases<-logistf(autism~male+y5_teases, data=expl2)

ry5_confidentwithothers<-logistf(autism~male+y5_confidentwithothers, data=expl2)

ry5_treatenspeople<-logistf(autism~male+y5_treatenspeople, data=expl2)

ry5_proud<-logistf(autism~male+y5_proud, data=expl2)

ry5_underactive<-logistf(autism~male+y5_underactive, data=expl2)

ry5_unusuallyloud<-logistf(autism~male+y5_unusuallyloud, data=expl2)

ry5_interestdiffthings<-logistf(autism~male+y5_interestdiffthings, data=expl2)

ry5_vandalizes<-logistf(autism~male+y5_vandalizes, data=expl2)

ry5_worries<-logistf(autism~male+y5_worries, data=expl2)

ry5_enjoystalktoyou<-logistf(autism~male+y5_enjoystalktoyou, data=expl2)

ry5_cantconcentrate<-logistf(autism~male+y5_cantconcentrate, data=expl2)

ry5_cantsitstill<-logistf(autism~male+y5_cantsitstill, data=expl2)

ry5_clingsadults<-logistf(autism~male+y5_clingsadults, data=expl2)

ry5_criesalot<-logistf(autism~male+y5_criesalot, data=expl2)

ry5_disobedient<-logistf(autism~male+y5_disobedient, data=expl2)

ry5_dontgetalongotherchldrn<-logistf(autism~male+y5_dontgetalongotherchldrn, data=expl2)

ry5_noguilt<-logistf(autism~male+y5_noguilt, data=expl2)

ry5_troublefallingasleep<-logistf(autism~male+y5_troublefallingasleep, data=expl2)

ry5_nervous<-logistf(autism~male+y5_nervous, data=expl2)

ry5_stubborn<-logistf(autism~male+y5_stubborn, data=expl2)

ry5_suddenmoodswings<-logistf(autism~male+y5_suddenmoodswings, data=expl2)

ry5_tempertantrums<-logistf(autism~male+y5_tempertantrums, data=expl2)

ry5_anxious<-logistf(autism~male+y5_anxious, data=expl2)

ry5_sad<-logistf(autism~male+y5_sad, data=expl2)

ry5_wantsalotattention<-logistf(autism~male+y5_wantsalotattention, data=expl2)

ry5_withdrawnotherchildren<-logistf(autism~male+y5_withdrawnotherchildren, data=expl2)

ry5_feelworthless<-logistf(autism~male+y5_feelworthless, data=expl2)

ry5_actstooyoungforage<-logistf(autism~male+y5_actstooyoungforage, data=expl2)

summary(ry5_under7hoursofsleep)

summary(ry5_sleepinparentsbed)

summary(ry5_argues)#***

summary(ry5_brags)

summary(ry5_undstotherfeelings)#*

summary(ry5_overeats)

summary(ry5_loneliness)

summary(ry5_confused)#***

summary(ry5_cruelothers)#**

summary(ry5_daydreams)#**

summary(ry5_destroyownthings)#**

summary(ry5_destroyotherthings)#**

summary(ry5_sympathyothers)#**

summary(ry5_disobedienthome)#**

summary(ry5_disobedientschool)#**

summary(ry5_accidentprone)#**

summary(ry5_openwhatwant)

summary(ry5_jealous)

summary(ry5_dothinkbadobsession)

summary(ry5_hastobeperfect)

summary(ry5_complainsnoloveshim)

summary(ry5_otheroutotget)

summary(ry5_getintofights)#**

summary(ry5_getteased)#*

summary(ry5_hangswotherstrouble)

summary(ry5_impulsive)#***

summary(ry5_ratherbealone)#***

summary(ry5_liescheats)

summary(ry5_nervoustwitches)#***

summary(ry5_notlikedbyothers)

summary(ry5_feelstooguilty)

summary(ry5_joinsothersplay)#***

summary(ry5_overweight)

summary(ry5_justwatchothers)

summary(ry5_attackspeople)#**

summary(ry5_poorschoolwork)

summary(ry5_clumsy)#***

summary(ry5_preferolderkids)

summary(ry5_preferyoungerkids)

summary(ry5_refusestotalk)

summary(ry5_runsawayfromhome)

summary(ry5_screamsalot)

summary(ry5_attentionfromothers)

summary(ry5_secretive)

summary(ry5_easilyembarassed)

summary(ry5_setsfires)

summary(ry5_clownsaround)

summary(ry5_shy)

summary(ry5_staresblankly)#*

summary(ry5_saysthanksplease)

summary(ry5_stealshome)

summary(ry5_stealsoutsidehome)

summary(ry5_wanttoplayothers)#***

summary(ry5_sulks)

summary(ry5_suspicious)

summary(ry5_swears)

summary(ry5_playstalkothers)#***

summary(ry5_talkstoomuch)

summary(ry5_teases)

summary(ry5_confidentwithothers)

summary(ry5_treatenspeople)#***

summary(ry5_proud)#*

summary(ry5_underactive)#*

summary(ry5_unusuallyloud)#**

summary(ry5_interestdiffthings)#**

summary(ry5_vandalizes)

summary(ry5_worries)

summary(ry5_enjoystalktoyou)#**

summary(ry5_cantconcentrate)#***

summary(ry5_cantsitstill)#***

summary(ry5_clingsadults)#***

summary(ry5_criesalot)#***

summary(ry5_disobedient)#***

summary(ry5_dontgetalongotherchldrn)#***

summary(ry5_noguilt)

summary(ry5_troublefallingasleep)#***

summary(ry5_nervous)#***

summary(ry5_stubborn)#***

summary(ry5_suddenmoodswings)#***

summary(ry5_tempertantrums)#***

summary(ry5_anxious)#***

summary(ry5_sad)#**

summary(ry5_wantsalotattention)#***

summary(ry5_withdrawnotherchildren)#***

summary(ry5_feelworthless)#**

summary(ry5_actstooyoungforage)#***

CrossTable(expl2$autism, expl2$y5_argues)

CrossTable(expl2$autism, expl2$y5_confused)

CrossTable(expl2$autism, expl2$y5_cruelothers)

CrossTable(expl2$autism, expl2$y5_daydreams)

CrossTable(expl2$autism, expl2$y5_destroyownthings)

CrossTable(expl2$autism, expl2$y5_destroyotherthings)

CrossTable(expl2$autism, expl2$y5_sympathyothers)

CrossTable(expl2$autism, expl2$y5_disobedienthome)

CrossTable(expl2$autism, expl2$y5_disobedientschool)

CrossTable(expl2$autism, expl2$y5_accidentprone)

CrossTable(expl2$autism, expl2$y5_getintofights)

CrossTable(expl2$autism, expl2$y5_impulsive)

CrossTable(expl2$autism, expl2$y5_ratherbealone)

CrossTable(expl2$autism, expl2$y5_nervoustwitches)

CrossTable(expl2$autism, expl2$y5_joinsothersplay)

CrossTable(expl2$autism, expl2$y5_attackspeople)

CrossTable(expl2$autism, expl2$y5_clumsy)

CrossTable(expl2$autism, expl2$y5_wanttoplayothers)

CrossTable(expl2$autism, expl2$y5_playstalkothers)

CrossTable(expl2$autism, expl2$y5_treatenspeople)

CrossTable(expl2$autism, expl2$y5_unusuallyloud)

CrossTable(expl2$autism, expl2$y5_interestdiffthings)

CrossTable(expl2$autism, expl2$y5_enjoystalktoyou)

CrossTable(expl2$autism, expl2$y5_cantconcentrate)

CrossTable(expl2$autism, expl2$y5_cantsitstill)

CrossTable(expl2$autism, expl2$y5_clingsadults)

CrossTable(expl2$autism, expl2$y5_criesalot)

CrossTable(expl2$autism, expl2$y5_disobedient)

CrossTable(expl2$autism, expl2$y5_dontgetalongotherchldrn)

CrossTable(expl2$autism, expl2$y5_troublefallingasleep)

CrossTable(expl2$autism, expl2$y5_nervous)

CrossTable(expl2$autism, expl2$y5_stubborn)

CrossTable(expl2$autism, expl2$y5_suddenmoodswings)

CrossTable(expl2$autism, expl2$y5_tempertantrums)

CrossTable(expl2$autism, expl2$y5_anxious)

CrossTable(expl2$autism, expl2$y5_sad)

CrossTable(expl2$autism, expl2$y5_wantsalotattention)

CrossTable(expl2$autism, expl2$y5_withdrawnotherchildren)

CrossTable(expl2$autism, expl2$y5_feelworthless)

CrossTable(expl2$autism, expl2$y5_actstooyoungforage)

ry5_mdoc_lowpositiveaffect<-logistf(autism~male+y5_mdoc_lowpositiveaffect, data=expl2)

ry5_mdoc_highnegativeaffect<-logistf(autism~male+y5_mdoc_highnegativeaffect, data=expl2)

ry5_mdoc_detached<-logistf(autism~male+y5_mdoc_detached, data=expl2)

ry5_mdoc_notverbal<-logistf(autism~male+y5_mdoc_notverbal, data=expl2)

summary(ry5_mdoc_lowpositiveaffect)

summary(ry5_mdoc_highnegativeaffect)

summary(ry5_mdoc_detached)

summary(ry5_mdoc_notverbal)

# Create evenly distributed synthetic data

data.rose <- ROSE(autism ~ y5_argues+

y5_confused+

y5_cruelothers+

y5_daydreams+

y5_destroyownthings+

y5_destroyotherthings+

y5_sympathyothers+

y5_disobedienthome+

y5_disobedientschool+

y5_accidentprone+

y5_getintofights+

y5_impulsive+

y5_ratherbealone,

data = expl2, seed = 1)$data

# Run bestglm to find best subset model. Use syntehetic data to get better coefficients to find cases

lowbwt.bglm <- data.rose[, c("y5_argues",

"y5_confused",

"y5_cruelothers",

"y5_daydreams",

"y5_destroyownthings",

"y5_destroyotherthings",

"y5_sympathyothers",

"y5_disobedienthome",

"y5_disobedientschool",

"y5_accidentprone",

"y5_getintofights",

"y5_impulsive",

"y5_ratherbealone","autism")]

names(lowbwt.bglm)[names(lowbwt.bglm) == "autism"] <- "y"

lowbwt.bglm <- as.data.frame(lowbwt.bglm)

best.logit <- bestglm(lowbwt.bglm,

IC = "AIC",

family=binomial,

method = "exhaustive")

summary(best.logit$BestModel)

#y5_argues 0.3867 0.1002 3.86 0.00011 ***

#y5_confused 1.3628 0.3069 4.44 0.00000895432176 ***

#y5_daydreams 0.4781 0.1858 2.57 0.01006 *

#y5_destroyownthings 0.2362 0.1645 1.44 0.15114

#y5_destroyotherthings 0.3622 0.1850 1.96 0.05028 .

#y5_sympathyothers -0.5152 0.0917 -5.62 0.00000001898847 ***

#y5_disobedienthome 0.3163 0.1513 2.09 0.03653 *

#y5_accidentprone 0.4218 0.1620 2.60 0.00924 **

#y5_getintofights -0.4570 0.2672 -1.71 0.08724 .

#y5_impulsive 0.4045 0.1618 2.50 0.01245 *

#y5_ratherbealone 1.1315 0.1567 7.22 0.00000000000051 ***

data.rose <- ROSE(autism ~ y5_nervoustwitches+

y5_joinsothersplay+

y5_attackspeople+

y5_clumsy+

y5_wanttoplayothers+

y5_playstalkothers+

y5_treatenspeople+

y5_unusuallyloud+

y5_interestdiffthings+

y5_enjoystalktoyou+

y5_cantconcentrate+

y5_cantsitstill+

y5_clingsadults,

data = expl2, seed = 1)$data

# Run bestglm to find best subset model. Use syntehetic data to get better coefficients to find cases

lowbwt.bglm <- data.rose[, c("y5_nervoustwitches",

"y5_joinsothersplay",

"y5_attackspeople",

"y5_clumsy",

"y5_wanttoplayothers",

"y5_playstalkothers",

"y5_treatenspeople",

"y5_unusuallyloud",

"y5_interestdiffthings",

"y5_enjoystalktoyou",

"y5_cantconcentrate",

"y5_cantsitstill","y5_clingsadults","autism")]

names(lowbwt.bglm)[names(lowbwt.bglm) == "autism"] <- "y"

lowbwt.bglm <- as.data.frame(lowbwt.bglm)

best.logit <- bestglm(lowbwt.bglm,

IC = "AIC",

family=binomial,

method = "exhaustive")

summary(best.logit$BestModel)

#y5_nervoustwitches 1.471 0.232 6.33 0.00000000024 ***

#y5_joinsothersplay -0.297 0.108 -2.76 0.00584 **

#y5_attackspeople 0.732 0.334 2.19 0.02827 *

#y5_clumsy 1.344 0.230 5.85 0.00000000495 ***

#y5_wanttoplayothers -0.393 0.109 -3.62 0.00030 ***

#y5_playstalkothers -0.632 0.111 -5.67 0.00000001395 ***

#y5_treatenspeople 0.791 0.288 2.75 0.00596 **

#y5_unusuallyloud 0.481 0.125 3.86 0.00011 ***

#y5_cantconcentrate 0.919 0.150 6.12 0.00000000091 ***

#y5_cantsitstill 0.481 0.131 3.68 0.00023 ***

data.rose <- ROSE(autism ~ y5_criesalot+

y5_disobedient+

y5_dontgetalongotherchldrn+

y5_troublefallingasleep+

y5_nervous+

y5_stubborn+

y5_suddenmoodswings+

y5_tempertantrums+

y5_anxious+

y5_sad+

y5_wantsalotattention+

y5_withdrawnotherchildren+

y5_feelworthless+

y5_actstooyoungforage,

data = expl2, seed = 1)$data

# Run bestglm to find best subset model. Use syntehetic data to get better coefficients to find cases

lowbwt.bglm <- data.rose[, c("y5_criesalot",

"y5_disobedient",

"y5_dontgetalongotherchldrn",

"y5_troublefallingasleep",

"y5_nervous",

"y5_stubborn",

"y5_suddenmoodswings",

"y5_tempertantrums",

"y5_anxious",

"y5_sad",

"y5_wantsalotattention",

"y5_withdrawnotherchildren",

"y5_feelworthless",

"y5_actstooyoungforage","autism")]

names(lowbwt.bglm)[names(lowbwt.bglm) == "autism"] <- "y"

lowbwt.bglm <- as.data.frame(lowbwt.bglm)

best.logit <- bestglm(lowbwt.bglm,

IC = "AIC",

family=binomial,

method = "exhaustive")

summary(best.logit$BestModel)

#y5_dontgetalongotherchldrn 0.6703 0.2228 3.01 0.0026 **

#y5_troublefallingasleep 0.8221 0.1530 5.37 0.0000000773 ***

#y5_nervous 0.8408 0.1735 4.85 0.0000012525 ***

#y5_stubborn 0.7316 0.1258 5.81 0.0000000061 ***

#y5_suddenmoodswings 0.2342 0.1416 1.65 0.0980 .

#y5_tempertantrums 0.2156 0.1342 1.61 0.1082

#y5_withdrawnotherchildren 0.6341 0.2042 3.11 0.0019 **

#y5_actstooyoungforage 1.6748 0.1655 10.12 < 0.0000000000000002 ***

data.rose <- ROSE(autism ~ y5_treatenspeople+y5_unusuallyloud+y5_cantconcentrate+y5_cantsitstill+

y5_dontgetalongotherchldrn+y5_troublefallingasleep+y5_nervous+y5_stubborn+y5_withdrawnotherchildren+y5_actstooyoungforage,

data = expl2, seed = 1)$data

lowbwt.bglm <- data.rose[, c("y5_treatenspeople","y5_unusuallyloud","y5_cantconcentrate","y5_cantsitstill",

"y5_dontgetalongotherchldrn","y5_troublefallingasleep","y5_nervous","y5_stubborn","y5_withdrawnotherchildren","y5_actstooyoungforage","autism")]

names(lowbwt.bglm)[names(lowbwt.bglm) == "autism"] <- "y"

lowbwt.bglm <- as.data.frame(lowbwt.bglm)

best.logit <- bestglm(lowbwt.bglm,

IC = "AIC",

family=binomial,

method = "exhaustive")

summary(best.logit$BestModel)

#y5_treatenspeople 1.0297 0.2856 3.61 0.00031 ***

#y5_unusuallyloud 0.2563 0.1240 2.07 0.03873 *

#y5_cantconcentrate 0.6081 0.1566 3.88 0.00010 ***

#y5_cantsitstill 0.3121 0.1354 2.30 0.02117 *

#y5_dontgetalongotherchldrn 0.4034 0.2674 1.51 0.13139

#y5_troublefallingasleep 0.8019 0.1655 4.84 0.0000012737762887 ***

#y5_nervous 0.7007 0.2046 3.42 0.00062 ***

#y5_stubborn 0.7560 0.1354 5.58 0.0000000237690366 ***

#y5_withdrawnotherchildren 1.4144 0.2971 4.76 0.0000019213583446 ***

#y5_actstooyoungforage 1.6550 0.2074 7.98 0.0000000000000015 ***

data.rose <- ROSE(autism ~ y5_argues+y5_confused+y5_sympathyothers+y5_accidentprone+y5_ratherbealone+

y5_nervoustwitches+y5_joinsothersplay+y5_clumsy+y5_wanttoplayothers+y5_playstalkothers,

data = expl2, seed = 1)$data

lowbwt.bglm <- data.rose[, c("y5_argues","y5_confused","y5_sympathyothers","y5_accidentprone","y5_ratherbealone",

"y5_nervoustwitches","y5_joinsothersplay","y5_clumsy","y5_wanttoplayothers","y5_playstalkothers","autism")]

names(lowbwt.bglm)[names(lowbwt.bglm) == "autism"] <- "y"

lowbwt.bglm <- as.data.frame(lowbwt.bglm)

best.logit <- bestglm(lowbwt.bglm,

IC = "AIC",

family=binomial,

method = "exhaustive")

summary(best.logit$BestModel)

#y5_argues 0.251 0.109 2.31 0.02066 *

#y5_confused 0.733 0.321 2.28 0.02248 *

#y5_sympathyothers -0.166 0.103 -1.61 0.10674

#y5_accidentprone 1.026 0.184 5.59 0.0000000227506 ***

#y5_ratherbealone 1.188 0.168 7.08 0.0000000000014 ***

#y5_nervoustwitches 1.226 0.230 5.33 0.0000000961648 ***

#y5_joinsothersplay -0.503 0.105 -4.82 0.0000014563656 ***

#y5_clumsy 0.711 0.237 2.99 0.00276 **

#y5_wanttoplayothers -0.408 0.109 -3.73 0.00019 ***

#y5_playstalkothers -0.581 0.113 -5.13 0.0000002866860 ***

#### FINAL MODEL

data.rose <- ROSE(autism ~ y5_treatenspeople+y5_cantconcentrate+y5_troublefallingasleep+y5_nervous+

y5_stubborn+y5_withdrawnotherchildren+y5_actstooyoungforage+

y5_accidentprone+y5_ratherbealone+y5_nervoustwitches+y5_joinsothersplay+

y5_clumsy+y5_wanttoplayothers+y5_playstalkothers,

data = expl2, seed = 1)$data

lowbwt.bglm <- data.rose[, c("y5_treatenspeople","y5_cantconcentrate","y5_troublefallingasleep","y5_nervous",

"y5_stubborn","y5_withdrawnotherchildren","y5_actstooyoungforage",

"y5_accidentprone","y5_ratherbealone","y5_nervoustwitches","y5_joinsothersplay",

"y5_clumsy","y5_wanttoplayothers","y5_playstalkothers","autism")]

names(lowbwt.bglm)[names(lowbwt.bglm) == "autism"] <- "y"

lowbwt.bglm <- as.data.frame(lowbwt.bglm)

best.logit <- bestglm(lowbwt.bglm,

IC = "AIC",

family=binomial,

method = "exhaustive")

summary(best.logit$BestModel)

#y5_treatenspeople 1.037 0.288 3.60 0.00032 ***

#y5_cantconcentrate 0.742 0.159 4.66 0.000003130661 ***

#y5_troublefallingasleep 0.847 0.161 5.26 0.000000145864 ***

#y5_nervous 0.819 0.210 3.90 0.000095643338 ***

#y5_stubborn 0.571 0.147 3.89 0.000098900704 ***

#y5_withdrawnotherchildren 0.490 0.277 1.77 0.07646 .

#y5_actstooyoungforage 0.920 0.202 4.56 0.000005213793 ***

#y5_accidentprone 0.411 0.193 2.13 0.03289 *

#y5_ratherbealone 1.126 0.171 6.58 0.000000000046 ***

#y5_nervoustwitches 0.911 0.250 3.64 0.00027 ***

#y5_clumsy 0.792 0.265 2.99 0.00280 **

#y5_wanttoplayothers -0.263 0.107 -2.46 0.01375 *

#y5_playstalkothers -0.539 0.118 -4.58 0.000004719398 ***

## Accuracy

glm_probs <- data.frame(probs = predict(best.logit$BestModel, newdata = asdimpholdout5,type="response"))

glm_pred <- glm_probs %>% mutate(pred = ifelse(probs>.5, "1", "0"))

glm_pred <- cbind(asdimpholdout5, glm_pred)

glm_pred %>% count(pred, autism) %>% spread(autism, n, fill = 0)

# Imp 1

# pred 0 1

#1 0 2279 24

#2 1 675 67

#Sensitivity

67/(67+24)*100 # 73.6%

#PPV

67/(675+67)*100 # 9.0%

# Imp 2

# pred 0 1

#1 0 2314 24

#2 1 640 67

#Sensitivity

67/(67+24)*100 #73.6%

#PPV

67/(640+67)*100 # 9.5%

# Imp 3

# pred 0 1

#1 0 2324 24

#2 1 630 67

#Sensitivity

67/(67+24)*100 # 73.6%

#PPV

67/(630+67)*100 # 9.6%

# Imp 4

# pred 0 1

#1 0 2306 24

#2 1 648 67

#Sensitivity

67/(67+24)*100 # 73.6%

#PPV

67/(648+67)*100 # 9.4%

# Imp 5

# pred 0 1

#1 0 2288 24

#2 1 666 67

#Sensitivity

67/(67+24)*100 # 73.6%

#PPV

67/(666+67)*100 # 9.1%

#Pooled sensitivity

(73.6+73.6+73.6+73.6+73.6)/5 ## 73.6%

#Pooled PPV

(9.0+9.1+9.4+9.5+9.6)/5 ## 9.3%

################################

######## Teacher-report Year 5

################################

ry5_teacher_seldomworktobestability <- logistf(autism~male+y5_teacher_seldomworktobestability, data=expl2)

ry5_teacher_diagnoseddisability <- logistf(autism~male+y5_teacher_diagnoseddisability, data=expl2)

ry5_teacher_behindinschool <- logistf(autism~male+y5_teacher_behindinschool, data=expl2)# Don't work, too few observation

ry5_teacher_understandstory <- logistf(autism~male+y5_teacher_understandstory, data=expl2)

ry5_teacher_easilynameletters <- logistf(autism~male+y5_teacher_easilynameletters, data=expl2)

ry5_teacher_readssimplebooks <- logistf(autism~male+y5_teacher_readssimplebooks, data=expl2)

ry5_teacher_understconvofprint <- logistf(autism~male+y5_teacher_understconvofprint, data=expl2)

ry5_teacher_recogndiffpeople <- logistf(autism~male+y5_teacher_recogndiffpeople, data=expl2)

ry5_teacher_expl2aneobserv <- logistf(autism~male+y5_teacher_explaneobserv, data=expl2)

ry5_teacher_sortscompmath <- logistf(autism~male+y5_teacher_sortscompmath, data=expl2)

ry5_teacher_relofquantities <- logistf(autism~male+y5_teacher_relofquantities, data=expl2)

ry5_teacher_varietyinmath <- logistf(autism~male+y5_teacher_varietyinmath, data=expl2)

ry5_teacher_belowaverlanguage <- logistf(autism~male+y5_teacher_belowaverlanguage, data=expl2)

ry5_teacher_belowaversocialsci <- logistf(autism~male+y5_teacher_belowaversocialsci, data=expl2)

ry5_teacher_belowavermath <- logistf(autism~male+y5_teacher_belowavermath, data=expl2)

ry5_teacher_activestrucutured <- logistf(autism~male+y5_teacher_activestrucutured, data=expl2)

ry5_teacher_activeunstrucutured <- logistf(autism~male+y5_teacher_activeunstrucutured, data=expl2)

ry5_teacher_discussproblsparents <- logistf(autism~male+y5_teacher_discussproblsparents, data=expl2)

summary(ry5_teacher_seldomworktobestability)#*

summary(ry5_teacher_diagnoseddisability)#***

summary(ry5_teacher_understandstory)

summary(ry5_teacher_easilynameletters)

summary(ry5_teacher_readssimplebooks)#*

summary(ry5_teacher_understconvofprint)#***

summary(ry5_teacher_recogndiffpeople)#*

summary(ry5_teacher_expl2aneobserv)#*

summary(ry5_teacher_sortscompmath)#**

summary(ry5_teacher_relofquantities)

summary(ry5_teacher_varietyinmath)#**

summary(ry5_teacher_belowaverlanguage)#***

summary(ry5_teacher_belowaversocialsci)#*

summary(ry5_teacher_belowavermath)#**

summary(ry5_teacher_activestrucutured)#*

summary(ry5_teacher_activeunstrucutured)#*

summary(ry5_teacher_discussproblsparents)#**

CrossTable(expl2$autism, expl2$y5_teacher_diagnoseddisability)

CrossTable(expl2$autism, expl2$y5_teacher_understconvofprint)

CrossTable(expl2$autism, expl2$y5_teacher_sortscompmath)

CrossTable(expl2$autism, expl2$y5_teacher_varietyinmath)

CrossTable(expl2$autism, expl2$y5_teacher_belowaverlanguage)

CrossTable(expl2$autism, expl2$y5_teacher_belowavermath)

CrossTable(expl2$autism, expl2$y5_teacher_discussproblsparents)

y5_teacher_diagnoseddisability+

y5_teacher_understconvofprint+

y5_teacher_sortscompmath+

y5_teacher_varietyinmath+

y5_teacher_belowaverlanguage+

y5_teacher_belowavermath+

y5_teacher_discussproblsparents

data.rose <- ROSE(autism ~ y5_teacher_diagnoseddisability+

y5_teacher_understconvofprint+

y5_teacher_sortscompmath+

y5_teacher_varietyinmath+

y5_teacher_belowaverlanguage+

y5_teacher_belowavermath+

y5_teacher_discussproblsparents,

data = expl2, seed = 1)$data

lowbwt.bglm <- data.rose[, c("y5_teacher_diagnoseddisability",

"y5_teacher_understconvofprint",

"y5_teacher_sortscompmath",

"y5_teacher_varietyinmath",

"y5_teacher_belowaverlanguage",

"y5_teacher_belowavermath",

"y5_teacher_discussproblsparents","autism")]

names(lowbwt.bglm)[names(lowbwt.bglm) == "autism"] <- "y"

lowbwt.bglm <- as.data.frame(lowbwt.bglm)

best.logit <- bestglm(lowbwt.bglm,

IC = "AIC",

family=binomial,

method = "exhaustive")

summary(best.logit$BestModel)

#y5_teacher_diagnoseddisability 1.863 0.241 7.73 0.000000000000011 ***

#y5_teacher_understconvofprint 1.598 0.244 6.54 0.000000000060767 ***

#y5_teacher_belowaverlanguage -0.453 0.246 -1.84 0.06515 .

#y5_teacher_discussproblsparents 0.856 0.228 3.75 0.00018 ***

## Accuracy

glm_probs <- data.frame(probs = predict(best.logit$BestModel, newdata = asdimpholdout5,type="response"))

glm_pred <- glm_probs %>% mutate(pred = ifelse(probs>.5, "1", "0"))

glm_pred <- cbind(asdimpholdout5, glm_pred)

glm_pred %>% count(pred, autism) %>% spread(autism, n, fill = 0)

# Imp 1

# pred 0 1

#1 0 1615 21

#2 1 1339 70

#Sensitivity

70/(70+21)*100 # 76.9%

#PPV

70/(1339+70)*100 # 5.0%

# Imp 2

# pred 0 1

#1 0 1742 13

#2 1 1212 78

#Sensitivity

78/(78+13)*100 #85.7%

#PPV

78/(1212+78)*100 # 6.1%

# Imp 3

# pred 0 1

#1 0 1353 24

#2 1 1601 67

#Sensitivity

67/(67+24)*100 # 73.6%

#PPV

67/(1601+67)*100 # 4.0%

# Imp 4

# pred 0 1

#1 0 1482 25

#2 1 1472 66

#Sensitivity

66/(66+25)*100 # 72.5%

#PPV

66/(1472+66)*100 # 4.3%

# Imp 5

# pred 0 1

#1 0 1421 41

#2 1 1533 50

#Sensitivity

50/(50+41)*100 # 55.0%

#PPV

50/(1533+50)*100 # 3.2%

#Pooled sensitivity

(76.9+85.7+73.6+72.5+55.0)/5 ## 72.7%

#Pooled PPV

(5.0+6.1+4.0+4.3+3.2)/5 ## 4.5%

### ### ### ### ### ### ### ###

### Year 9 factors

### ### ### ### ### ### ### ###

#### Parent

ry9_parent_easychangebetweenactivities <-logistf(autism~male+y9_parent_easychangebetweenactivities,data=expl2)

ry9_parent_cantgetmindoffthoughts <-logistf(autism~male+y9_parent_cantgetmindoffthoughts,data=expl2)

ry9_parent_cantgetalongotherkids <-logistf(autism~male+y9_parent_cantgetalongotherkids,data=expl2)

ry9_parent_ratheralonethanothers <-logistf(autism~male+y9_parent_ratheralonethanothers,data=expl2)

ry9_parent_playssexpartstoomuch <-logistf(autism~male+y9_parent_playssexpartstoomuch,data=expl2)

ry9_parent_destroysothersthings <-logistf(autism~male+y9_parent_destroysothersthings,data=expl2)

ry9_parent_talkstoomuchaboutsex <-logistf(autism~male+y9_parent_talkstoomuchaboutsex,data=expl2)

ry9_parent_sleepslessthanothers <-logistf(autism~male+y9_parent_sleepslessthanothers,data=expl2)

ry9_parent_understandotherfeelings <-logistf(autism~male+y9_parent_understandotherfeelings,data=expl2)

ry9_parent_acceptifriendsdieasinplay <-logistf(autism~male+y9_parent_acceptifriendsdieasinplay,data=expl2)

ry9_parent_actotooyoung <-logistf(autism~male+y9_parent_actotooyoung,data=expl2)

ry9_parent_drinksalcohol <-logistf(autism~male+y9_parent_drinksalcohol,data=expl2)

ry9_parent_arguesalot <-logistf(autism~male+y9_parent_arguesalot,data=expl2)

ry9_parent_failstofinish <-logistf(autism~male+y9_parent_failstofinish,data=expl2)

ry9_parent_enjoysverylittle <-logistf(autism~male+y9_parent_enjoysverylittle,data=expl2)

ry9_parent_brags <-logistf(autism~male+y9_parent_brags,data=expl2)

ry9_parent_cantconcentrate <-logistf(autism~male+y9_parent_cantconcentrate,data=expl2)

ry9_parent_restless <-logistf(autism~male+y9_parent_restless,data=expl2)

ry9_parent_clingsadults <-logistf(autism~male+y9_parent_clingsadults,data=expl2)

ry9_parent_loneliness <-logistf(autism~male+y9_parent_loneliness,data=expl2)

ry9_parent_confused <-logistf(autism~male+y9_parent_confused,data=expl2)

ry9_parent_criesalot <-logistf(autism~male+y9_parent_criesalot,data=expl2)

ry9_parent_cruelanimals <-logistf(autism~male+y9_parent_cruelanimals,data=expl2)

ry9_parent_cruelothers <-logistf(autism~male+y9_parent_cruelothers,data=expl2)

ry9_parent_daydreams <-logistf(autism~male+y9_parent_daydreams,data=expl2)

ry9_parent_harmsself <-logistf(autism~male+y9_parent_harmsself,data=expl2)

ry9_parent_demandsattention <-logistf(autism~male+y9_parent_demandsattention,data=expl2)

ry9_parent_destrousownthings <-logistf(autism~male+y9_parent_destrousownthings,data=expl2)

ry9_parent_disobed_home <-logistf(autism~male+y9_parent_disobed_home,data=expl2)

ry9_parent_disob_school <-logistf(autism~male+y9_parent_disob_school,data=expl2)

ry9_parent_guiltyaftermisbeh <-logistf(autism~male+y9_parent_guiltyaftermisbeh,data=expl2)

ry9_parent_jelaous <-logistf(autism~male+y9_parent_jelaous,data=expl2)

ry9_parent_breakrules <-logistf(autism~male+y9_parent_breakrules,data=expl2)

ry9_parent_phobias <-logistf(autism~male+y9_parent_phobias,data=expl2)

ry9_parent_feargoingschool <-logistf(autism~male+y9_parent_feargoingschool,data=expl2)

ry9_parent_feardosomthngbad <-logistf(autism~male+y9_parent_feardosomthngbad,data=expl2)

ry9_parent_feelshastobeperfect <-logistf(autism~male+y9_parent_feelshastobeperfect,data=expl2)

ry9_parent_complainsnooneloves <-logistf(autism~male+y9_parent_complainsnooneloves,data=expl2)

ry9_parent_feelsotheroutotget <-logistf(autism~male+y9_parent_feelsotheroutotget,data=expl2)

ry9_parent_feelworthless <-logistf(autism~male+y9_parent_feelworthless,data=expl2)

ry9_parent_accidentprone <-logistf(autism~male+y9_parent_accidentprone,data=expl2)

ry9_parent_getinmanyfights <-logistf(autism~male+y9_parent_getinmanyfights,data=expl2)

ry9_parent_getteased <-logistf(autism~male+y9_parent_getteased,data=expl2)

ry9_parent_hangsothertrouble <-logistf(autism~male+y9_parent_hangsothertrouble,data=expl2)

ry9_parent_hearvocies <-logistf(autism~male+y9_parent_hearvocies,data=expl2)

ry9_parent_impulsive <-logistf(autism~male+y9_parent_impulsive,data=expl2)

ry9_parent_lies <-logistf(autism~male+y9_parent_lies,data=expl2)

ry9_parent_nervous <-logistf(autism~male+y9_parent_nervous,data=expl2)

ry9_parent_nervousmovements <-logistf(autism~male+y9_parent_nervousmovements,data=expl2)

ry9_parent_nightmares <-logistf(autism~male+y9_parent_nightmares,data=expl2)

ry9_parent_notlikedotherkids <-logistf(autism~male+y9_parent_notlikedotherkids,data=expl2)

ry9_parent_anxious <-logistf(autism~male+y9_parent_anxious,data=expl2)

ry9_parent_dizzy <-logistf(autism~male+y9_parent_dizzy,data=expl2)

ry9_parent_tooguilty <-logistf(autism~male+y9_parent_tooguilty,data=expl2)

ry9_parent_attacksphysically <-logistf(autism~male+y9_parent_attacksphysically,data=expl2)

ry9_parent_picksnoseskin <-logistf(autism~male+y9_parent_picksnoseskin,data=expl2)

ry9_parent_playssexpartspublic <-logistf(autism~male+y9_parent_playssexpartspublic,data=expl2)

ry9_parent_poorinschool <-logistf(autism~male+y9_parent_poorinschool,data=expl2)

ry9_parent_clumsy <-logistf(autism~male+y9_parent_clumsy,data=expl2)

ry9_parent_prefersolderkids <-logistf(autism~male+y9_parent_prefersolderkids,data=expl2)

ry9_parent_prefersyoungerkids <-logistf(autism~male+y9_parent_prefersyoungerkids,data=expl2)

ry9_parent_refusestotalk <-logistf(autism~male+y9_parent_refusestotalk,data=expl2)

ry9_parent_compulsions <-logistf(autism~male+y9_parent_compulsions,data=expl2)

ry9_parent_runsawayfromhome <-logistf(autism~male+y9_parent_runsawayfromhome,data=expl2)

ry9_parent_screams <-logistf(autism~male+y9_parent_screams,data=expl2)

ry9_parent_secretive <-logistf(autism~male+y9_parent_secretive,data=expl2)

ry9_parent_seesthiongsnotthere <-logistf(autism~male+y9_parent_seesthiongsnotthere,data=expl2)

ry9_parent_selfembarassed <-logistf(autism~male+y9_parent_selfembarassed,data=expl2)

ry9_parent_setsfires <-logistf(autism~male+y9_parent_setsfires,data=expl2)

ry9_parent_sexualproblems <-logistf(autism~male+y9_parent_sexualproblems,data=expl2)

ry9_parent_clowns <-logistf(autism~male+y9_parent_clowns,data=expl2)

ry9_parent_shytimid <-logistf(autism~male+y9_parent_shytimid,data=expl2)

ry9_parent_easydistracted <-logistf(autism~male+y9_parent_easydistracted,data=expl2)

ry9_parent_speechproblem <-logistf(autism~male+y9_parent_speechproblem,data=expl2)

ry9_parent_staresblankly <-logistf(autism~male+y9_parent_staresblankly,data=expl2)

ry9_parent_stealshome <-logistf(autism~male+y9_parent_stealshome,data=expl2)

ry9_parent_stealsoutsidehome <-logistf(autism~male+y9_parent_stealsoutsidehome,data=expl2)

ry9_parent_hoards <-logistf(autism~male+y9_parent_hoards,data=expl2)

ry9_parent_strangebehaviors <-logistf(autism~male+y9_parent_strangebehaviors,data=expl2)

ry9_parent_strangeideas <-logistf(autism~male+y9_parent_strangeideas,data=expl2)

ry9_parent_stubborn <-logistf(autism~male+y9_parent_stubborn,data=expl2)

ry9_parent_moodswings <-logistf(autism~male+y9_parent_moodswings,data=expl2)

ry9_parent_sulks <-logistf(autism~male+y9_parent_sulks,data=expl2)

ry9_parent_supsicious <-logistf(autism~male+y9_parent_supsicious,data=expl2)

ry9_parent_obsecenlanguage <-logistf(autism~male+y9_parent_obsecenlanguage,data=expl2)

ry9_parent_talkskillingself <-logistf(autism~male+y9_parent_talkskillingself,data=expl2)

ry9_parent_walksinsleep <-logistf(autism~male+y9_parent_walksinsleep,data=expl2)

ry9_parent_talktoomuch <-logistf(autism~male+y9_parent_talktoomuch,data=expl2)

ry9_parent_teasesalot <-logistf(autism~male+y9_parent_teasesalot,data=expl2)

ry9_parent_tempertantrums <-logistf(autism~male+y9_parent_tempertantrums,data=expl2)

ry9_parent_threatenspeople <-logistf(autism~male+y9_parent_threatenspeople,data=expl2)

ry9_parent_smokes <-logistf(autism~male+y9_parent_smokes,data=expl2)

ry9_parent_troublesleeping <-logistf(autism~male+y9_parent_troublesleeping,data=expl2)

ry9_parent_skipsschool <-logistf(autism~male+y9_parent_skipsschool,data=expl2)

ry9_parent_underactive <-logistf(autism~male+y9_parent_underactive,data=expl2)

ry9_parent_sad <-logistf(autism~male+y9_parent_sad,data=expl2)

ry9_parent_unusuallyloud <-logistf(autism~male+y9_parent_unusuallyloud,data=expl2)

ry9_parent_alcohol <-logistf(autism~male+y9_parent_alcohol,data=expl2)

ry9_parent_vandalizes <-logistf(autism~male+y9_parent_vandalizes,data=expl2)

ry9_parent_whines <-logistf(autism~male+y9_parent_whines,data=expl2)

ry9_parent_withdrawn <-logistf(autism~male+y9_parent_withdrawn,data=expl2)

ry9_parent_worries <-logistf(autism~male+y9_parent_worries,data=expl2)

ry9_parent_sympatheticothers <-logistf(autism~male+y9_parent_sympatheticothers,data=expl2)

ry9_parent_openwhatshewants <-logistf(autism~male+y9_parent_openwhatshewants,data=expl2)

ry9_parent_joingroupswhentoldso <-logistf(autism~male+y9_parent_joingroupswhentoldso,data=expl2)

ry9_parent_makesfriendseasily <-logistf(autism~male+y9_parent_makesfriendseasily,data=expl2)

ry9_parent_selfconfident <-logistf(autism~male+y9_parent_selfconfident,data=expl2)

ry9_parent_interestindiffthings <-logistf(autism~male+y9_parent_interestindiffthings,data=expl2)

ry9_parent_startconversations <-logistf(autism~male+y9_parent_startconversations,data=expl2)

ry9_parent_likedbyothers <-logistf(autism~male+y9_parent_likedbyothers,data=expl2)

ry9_parent_invitesotherstohome <-logistf(autism~male+y9_parent_invitesotherstohome,data=expl2)

ry9_parent_reportsaccappropr <-logistf(autism~male+y9_parent_reportsaccappropr,data=expl2)

summary(ry9_parent_easychangebetweenactivities)#***

summary(ry9_parent_cantgetmindoffthoughts)#***

summary(ry9_parent_cantgetalongotherkids)

summary(ry9_parent_ratheralonethanothers)#***

summary(ry9_parent_playssexpartstoomuch)#***

summary(ry9_parent_destroysothersthings)

summary(ry9_parent_talkstoomuchaboutsex)#*

summary(ry9_parent_sleepslessthanothers)#***

summary(ry9_parent_understandotherfeelings)#**

summary(ry9_parent_acceptifriendsdieasinplay)#***

summary(ry9_parent_actotooyoung)#***

summary(ry9_parent_drinksalcohol)#**

summary(ry9_parent_arguesalot)#*

summary(ry9_parent_failstofinish)#**

summary(ry9_parent_enjoysverylittle)#*

summary(ry9_parent_brags)

summary(ry9_parent_cantconcentrate)#***

summary(ry9_parent_restless)#***

summary(ry9_parent_clingsadults)#***

summary(ry9_parent_loneliness)#**

summary(ry9_parent_confused)#***

summary(ry9_parent_criesalot)#**

summary(ry9_parent_cruelanimals)#**

summary(ry9_parent_cruelothers)

summary(ry9_parent_daydreams)#***

summary(ry9_parent_harmsself)#**

summary(ry9_parent_demandsattention)#***

summary(ry9_parent_destrousownthings)#**

summary(ry9_parent_disobed_home)

summary(ry9_parent_disob_school)

summary(ry9_parent_guiltyaftermisbeh)

summary(ry9_parent_jelaous)#*

summary(ry9_parent_breakrules)

summary(ry9_parent_phobias)#***

summary(ry9_parent_feargoingschool)

summary(ry9_parent_feardosomthngbad)

summary(ry9_parent_feelshastobeperfect)

summary(ry9_parent_complainsnooneloves)#*

summary(ry9_parent_feelsotheroutotget)#*

summary(ry9_parent_feelworthless)#*

summary(ry9_parent_accidentprone)#***

summary(ry9_parent_getinmanyfights)

summary(ry9_parent_getteased)#***

summary(ry9_parent_hangsothertrouble)

summary(ry9_parent_hearvocies)

summary(ry9_parent_impulsive)#***

summary(ry9_parent_lies)

summary(ry9_parent_nervous)#***

summary(ry9_parent_nervousmovements)#**

summary(ry9_parent_nightmares)#**

summary(ry9_parent_notlikedotherkids)#**

summary(ry9_parent_anxious)#***

summary(ry9_parent_dizzy)

summary(ry9_parent_tooguilty)#*

summary(ry9_parent_attacksphysically)#**

summary(ry9_parent_picksnoseskin)#**

summary(ry9_parent_playssexpartspublic)

summary(ry9_parent_poorinschool)

summary(ry9_parent_clumsy)#***

summary(ry9_parent_prefersolderkids)

summary(ry9_parent_prefersyoungerkids)#***

summary(ry9_parent_refusestotalk)#*

summary(ry9_parent_compulsions)#***

summary(ry9_parent_runsawayfromhome)#*

summary(ry9_parent_screams)#**

summary(ry9_parent_secretive)

summary(ry9_parent_seesthiongsnotthere)

summary(ry9_parent_selfembarassed)

summary(ry9_parent_setsfires)

summary(ry9_parent_sexualproblems)#*

summary(ry9_parent_clowns)

summary(ry9_parent_shytimid)

summary(ry9_parent_easydistracted)#***

summary(ry9_parent_staresblankly)#**

summary(ry9_parent_stealshome)

summary(ry9_parent_stealsoutsidehome)

summary(ry9_parent_hoards)#*

summary(ry9_parent_strangebehaviors)#***

summary(ry9_parent_strangeideas)

summary(ry9_parent_stubborn)#*

summary(ry9_parent_moodswings)#*

summary(ry9_parent_sulks)

summary(ry9_parent_supsicious)

summary(ry9_parent_obsecenlanguage)#*

summary(ry9_parent_talkskillingself)

summary(ry9_parent_walksinsleep)

summary(ry9_parent_talktoomuch)

summary(ry9_parent_teasesalot)

summary(ry9_parent_tempertantrums)#**

summary(ry9_parent_threatenspeople)#*

summary(ry9_parent_smokes)#*

summary(ry9_parent_troublesleeping)#***

summary(ry9_parent_skipsschool)#*

summary(ry9_parent_underactive)

summary(ry9_parent_sad)#*

summary(ry9_parent_unusuallyloud)#*

summary(ry9_parent_alcohol)#*

summary(ry9_parent_vandalizes)#***

summary(ry9_parent_whines)#***

summary(ry9_parent_withdrawn)#***

summary(ry9_parent_worries)#***

summary(ry9_parent_sympatheticothers)#*

summary(ry9_parent_openwhatshewants)

summary(ry9_parent_joingroupswhentoldso)#***

summary(ry9_parent_makesfriendseasily)#***

summary(ry9_parent_selfconfident)#***

summary(ry9_parent_interestindiffthings)#***

summary(ry9_parent_startconversations)

summary(ry9_parent_likedbyothers)#***

summary(ry9_parent_invitesotherstohome)#***

summary(ry9_parent_reportsaccappropr)#***

CrossTable(expl2$autism, expl2$y9_parent_easychangebetweenactivities)

CrossTable(expl2$autism, expl2$y9_parent_cantgetmindoffthoughts)

CrossTable(expl2$autism, expl2$y9_parent_ratheralonethanothers)

CrossTable(expl2$autism, expl2$y9_parent_playssexpartstoomuch)

CrossTable(expl2$autism, expl2$y9_parent_sleepslessthanothers)

CrossTable(expl2$autism, expl2$y9_parent_understandotherfeelings)

CrossTable(expl2$autism, expl2$y9_parent_acceptifriendsdieasinplay)

CrossTable(expl2$autism, expl2$y9_parent_actotooyoung)

CrossTable(expl2$autism, expl2$y9_parent_drinksalcohol)

CrossTable(expl2$autism, expl2$y9_parent_failstofinish)

CrossTable(expl2$autism, expl2$y9_parent_cantconcentrate)

CrossTable(expl2$autism, expl2$y9_parent_restless)

CrossTable(expl2$autism, expl2$y9_parent_clingsadults)

CrossTable(expl2$autism, expl2$y9_parent_loneliness)

CrossTable(expl2$autism, expl2$y9_parent_confused)

CrossTable(expl2$autism, expl2$y9_parent_criesalot)

CrossTable(expl2$autism, expl2$y9_parent_cruelanimals)

CrossTable(expl2$autism, expl2$y9_parent_daydreams)

CrossTable(expl2$autism, expl2$y9_parent_harmsself)

CrossTable(expl2$autism, expl2$y9_parent_demandsattention)

CrossTable(expl2$autism, expl2$y9_parent_destrousownthings)

CrossTable(expl2$autism, expl2$y9_parent_phobias)

CrossTable(expl2$autism, expl2$y9_parent_accidentprone)

CrossTable(expl2$autism, expl2$y9_parent_getteased)

CrossTable(expl2$autism, expl2$y9_parent_impulsive)

CrossTable(expl2$autism, expl2$y9_parent_nervous)

CrossTable(expl2$autism, expl2$y9_parent_nervousmovements)

CrossTable(expl2$autism, expl2$y9_parent_nightmares)

CrossTable(expl2$autism, expl2$y9_parent_notlikedotherkids)

CrossTable(expl2$autism, expl2$y9_parent_anxious)

CrossTable(expl2$autism, expl2$y9_parent_attacksphysically)

CrossTable(expl2$autism, expl2$y9_parent_picksnoseskin)

CrossTable(expl2$autism, expl2$y9_parent_clumsy)

CrossTable(expl2$autism, expl2$y9_parent_prefersyoungerkids)

CrossTable(expl2$autism, expl2$y9_parent_compulsions)

CrossTable(expl2$autism, expl2$y9_parent_screams)

CrossTable(expl2$autism, expl2$y9_parent_easydistracted)

CrossTable(expl2$autism, expl2$y9_parent_staresblankly)

CrossTable(expl2$autism, expl2$y9_parent_strangebehaviors)

CrossTable(expl2$autism, expl2$y9_parent_tempertantrums)

CrossTable(expl2$autism, expl2$y9_parent_troublesleeping)

CrossTable(expl2$autism, expl2$y9_parent_vandalizes)

CrossTable(expl2$autism, expl2$y9_parent_whines)

CrossTable(expl2$autism, expl2$y9_parent_withdrawn)

CrossTable(expl2$autism, expl2$y9_parent_worries)

CrossTable(expl2$autism, expl2$y9_parent_joingroupswhentoldso)

CrossTable(expl2$autism, expl2$y9_parent_makesfriendseasily)

CrossTable(expl2$autism, expl2$y9_parent_selfconfident)

CrossTable(expl2$autism, expl2$y9_parent_interestindiffthings)

CrossTable(expl2$autism, expl2$y9_parent_likedbyothers)

CrossTable(expl2$autism, expl2$y9_parent_invitesotherstohome)

CrossTable(expl2$autism, expl2$y9_parent_reportsaccappropr)

## First 13

data.rose <- ROSE(autism ~ y9_parent_easychangebetweenactivities+

y9_parent_cantgetmindoffthoughts+

y9_parent_ratheralonethanothers+

y9_parent_playssexpartstoomuch+

y9_parent_sleepslessthanothers+

y9_parent_understandotherfeelings+

y9_parent_acceptifriendsdieasinplay+

y9_parent_actotooyoung+

y9_parent_drinksalcohol+

y9_parent_failstofinish+

y9_parent_cantconcentrate+

y9_parent_restless+

y9_parent_clingsadults,

data = expl2, seed = 1)$data

lowbwt.bglm <- data.rose[, c("y9_parent_easychangebetweenactivities",

"y9_parent_cantgetmindoffthoughts","y9_parent_ratheralonethanothers",

"y9_parent_playssexpartstoomuch","y9_parent_sleepslessthanothers",

"y9_parent_understandotherfeelings","y9_parent_acceptifriendsdieasinplay",

"y9_parent_actotooyoung","y9_parent_drinksalcohol",

"y9_parent_failstofinish","y9_parent_cantconcentrate",

"y9_parent_restless","y9_parent_clingsadults","autism")]

names(lowbwt.bglm)[names(lowbwt.bglm) == "autism"] <- "y"

lowbwt.bglm <- as.data.frame(lowbwt.bglm)

best.logit <- bestglm(lowbwt.bglm,

IC = "AIC",

family=binomial,

method = "exhaustive")

summary(best.logit$BestModel)

#y9_parent_easychangebetweenactivities -0.48473 0.10633 -4.56 0.000005146371 ***

#y9_parent_cantgetmindoffthoughts 0.88900 0.16010 5.55 0.000000028111 ***

#y9_parent_ratheralonethanothers 1.20144 0.19868 6.05 0.000000001475 ***

#y9_parent_playssexpartstoomuch 1.70826 0.33023 5.17 0.000000230486 ***

#y9_parent_sleepslessthanothers 0.80592 0.22028 3.66 0.00025 ***

#y9_parent_understandotherfeelings -0.42626 0.09586 -4.45 0.000008720370 ***

#y9_parent_acceptifriendsdieasinplay -0.96685 0.10459 -9.24 < 0.0000000000000002 ***

#y9_parent_actotooyoung 1.03162 0.17909 5.76 0.000000008390 ***

#y9_parent_drinksalcohol 0.58408 0.32669 1.79 0.07380 .

#y9_parent_cantconcentrate 0.34607 0.14826 2.33 0.01958 *

#y9_parent_restless 0.95396 0.13384 7.13 0.000000000001 ***

#y9_parent_clingsadults 0.72054 0.15116 4.77 0.000001872411 ***

y9_parent_easychangebetweenactivities+y9_parent_cantgetmindoffthoughts+y9_parent_ratheralonethanothersy9_parent_playssexpartstoomuch+

y9_parent_sleepslessthanothers+y9_parent_understandotherfeelings+y9_parent_acceptifriendsdieasinplay+y9_parent_actotooyoung+

y9_parent_restless+y9_parent_clingsadults

## Second 13

data.rose <- ROSE(autism ~ y9_parent_loneliness+

y9_parent_confused+

y9_parent_criesalot+

y9_parent_cruelanimals+

y9_parent_daydreams+

y9_parent_harmsself+

y9_parent_demandsattention+

y9_parent_destrousownthings+

y9_parent_phobias+

y9_parent_accidentprone+

y9_parent_getteased+

y9_parent_impulsive+

y9_parent_nervous,

data = expl2, seed = 1)$data

lowbwt.bglm <- data.rose[, c("y9_parent_loneliness","y9_parent_confused",

"y9_parent_criesalot","y9_parent_cruelanimals",

"y9_parent_daydreams","y9_parent_harmsself",

"y9_parent_demandsattention","y9_parent_destrousownthings",

"y9_parent_phobias","y9_parent_accidentprone",

"y9_parent_getteased","y9_parent_impulsive",

"y9_parent_nervous","autism")]

names(lowbwt.bglm)[names(lowbwt.bglm) == "autism"] <- "y"

lowbwt.bglm <- as.data.frame(lowbwt.bglm)

best.logit <- bestglm(lowbwt.bglm,

IC = "AIC",

family=binomial,

method = "exhaustive")

summary(best.logit$BestModel)

#y9_parent_confused 0.3837 0.2211 1.73 0.0827 .

#y9_parent_daydreams 0.9987 0.1633 6.11 0.00000000097 ***

#y9_parent_demandsattention 0.6234 0.1335 4.67 0.00000303521 ***

#y9_parent_destrousownthings 0.3447 0.2106 1.64 0.1017

#y9_parent_phobias 0.9566 0.1822 5.25 0.00000015125 ***

#y9_parent_accidentprone 0.6063 0.2266 2.68 0.0075 **

#y9_parent_getteased 0.7478 0.1854 4.03 0.00005493126 ***

#y9_parent_impulsive 0.7838 0.1890 4.15 0.00003368861 ***

#y9_parent_nervous 1.3985 0.2330 6.00 0.00000000195 ***

y9_parent_daydreams+y9_parent_demandsattention+y9_parent_phobias+y9_parent_accidentprone+

y9_parent_getteased+y9_parent_impulsive+y9_parent_nervous

## Third 13

data.rose <- ROSE(autism ~ y9_parent_nervousmovements+

y9_parent_nightmares+

y9_parent_notlikedotherkids+

y9_parent_anxious+

y9_parent_attacksphysically+

y9_parent_picksnoseskin+

y9_parent_clumsy+

y9_parent_prefersyoungerkids+

y9_parent_compulsions+

y9_parent_screams+

y9_parent_easydistracted+

y9_parent_staresblankly+

y9_parent_strangebehaviors,

data = expl2, seed = 1)$data

lowbwt.bglm <- data.rose[, c("y9_parent_nervousmovements",

"y9_parent_nightmares",

"y9_parent_notlikedotherkids",

"y9_parent_anxious",

"y9_parent_attacksphysically",

"y9_parent_picksnoseskin",

"y9_parent_clumsy",

"y9_parent_prefersyoungerkids",

"y9_parent_compulsions",

"y9_parent_screams",

"y9_parent_easydistracted",

"y9_parent_staresblankly",

"y9_parent_strangebehaviors","autism")]

names(lowbwt.bglm)[names(lowbwt.bglm) == "autism"] <- "y"

lowbwt.bglm <- as.data.frame(lowbwt.bglm)

best.logit <- bestglm(lowbwt.bglm,

IC = "AIC",

family=binomial,

method = "exhaustive")

summary(best.logit$BestModel)

#y9_parent_nightmares 1.1959 0.3393 3.52 0.00042 ***

#y9_parent_notlikedotherkids 1.0111 0.2650 3.82 0.00014 ***

#y9_parent_anxious 1.4327 0.2121 6.75 0.00000000001432 ***

#y9_parent_picksnoseskin 0.5914 0.2638 2.24 0.02497 *

#y9_parent_clumsy 0.8268 0.2496 3.31 0.00092 ***

#y9_parent_compulsions 1.3974 0.1932 7.23 0.00000000000047 ***

#y9_parent_screams 0.4904 0.2636 1.86 0.06284 .

#y9_parent_easydistracted 0.7884 0.1303 6.05 0.00000000144747 ***

#y9_parent_staresblankly 1.1961 0.2780 4.30 0.00001686709605 ***

#y9_parent_strangebehaviors 0.6729 0.2878 2.34 0.01940 *

## Last 13

data.rose <- ROSE(autism ~ y9_parent_tempertantrums+

y9_parent_troublesleeping+

y9_parent_vandalizes+

y9_parent_whines+

y9_parent_withdrawn+

y9_parent_worries+

y9_parent_joingroupswhentoldso+

y9_parent_makesfriendseasily+

y9_parent_selfconfident+

y9_parent_interestindiffthings+

y9_parent_likedbyothers+

y9_parent_invitesotherstohome+

y9_parent_reportsaccappropr,

data = expl2, seed = 1)$data

lowbwt.bglm <- data.rose[, c("y9_parent_tempertantrums",

"y9_parent_troublesleeping",

"y9_parent_vandalizes",

"y9_parent_whines",

"y9_parent_withdrawn",

"y9_parent_worries",

"y9_parent_joingroupswhentoldso",

"y9_parent_makesfriendseasily",

"y9_parent_selfconfident",

"y9_parent_interestindiffthings",

"y9_parent_likedbyothers",

"y9_parent_invitesotherstohome",

"y9_parent_reportsaccappropr","autism")]

names(lowbwt.bglm)[names(lowbwt.bglm) == "autism"] <- "y"

lowbwt.bglm <- as.data.frame(lowbwt.bglm)

best.logit <- bestglm(lowbwt.bglm,

IC = "AIC",

family=binomial,

method = "exhaustive")

summary(best.logit$BestModel)

#y9_parent_tempertantrums 1.1613 0.1821 6.38 0.000000000181674 ***

#y9_parent_troublesleeping 0.8983 0.2108 4.26 0.000020377595223 ***

#y9_parent_whines 0.8822 0.1903 4.63 0.000003576562159 ***

#y9_parent_withdrawn 1.0712 0.3311 3.23 0.0012 **

#y9_parent_worries 0.6292 0.1571 4.01 0.000061748563341 ***

#y9_parent_joingroupswhentoldso -0.1778 0.1145 -1.55 0.1202

#y9_parent_makesfriendseasily -0.5836 0.1004 -5.82 0.000000006055918 ***

#y9_parent_selfconfident -0.9439 0.1221 -7.73 0.000000000000011 ***

#y9_parent_interestindiffthings -0.5722 0.0963 -5.94 0.000000002830770 ***

#y9_parent_invitesotherstohome -0.5425 0.1015 -5.34 0.000000091067419 ***

#y9_parent_reportsaccappropr -0.4298 0.0938 -4.58 0.000004637576370 ***

### New subsets

data.rose <- ROSE(autism ~ y9_parent_nightmares+y9_parent_notlikedotherkids+y9_parent_anxious+y9_parent_clumsy+

y9_parent_compulsions+y9_parent_easydistracted+y9_parent_staresblankly+

y9_parent_daydreams+y9_parent_demandsattention+y9_parent_phobias+y9_parent_accidentprone+

y9_parent_getteased,

data = expl2, seed = 1)$data

lowbwt.bglm <- data.rose[, c("y9_parent_nightmares","y9_parent_notlikedotherkids","y9_parent_anxious","y9_parent_clumsy",

"y9_parent_compulsions","y9_parent_easydistracted","y9_parent_staresblankly",

"y9_parent_daydreams","y9_parent_demandsattention","y9_parent_phobias","y9_parent_accidentprone",

"y9_parent_getteased","autism")]

names(lowbwt.bglm)[names(lowbwt.bglm) == "autism"] <- "y"

lowbwt.bglm <- as.data.frame(lowbwt.bglm)

best.logit <- bestglm(lowbwt.bglm,

IC = "AIC",

family=binomial,

method = "exhaustive")

summary(best.logit$BestModel)

#y9_parent_notlikedotherkids 1.4450 0.2625 5.51 0.0000000368876 ***

#y9_parent_anxious 1.7416 0.2807 6.20 0.0000000005524 ***

#y9_parent_clumsy 1.0230 0.2870 3.56 0.00036 ***

#y9_parent_compulsions 0.9936 0.1983 5.01 0.0000005436952 ***

#y9_parent_easydistracted 0.6306 0.1452 4.34 0.0000139879447 ***

#y9_parent_staresblankly 1.3229 0.3351 3.95 0.0000788292814 ***

#y9_parent_daydreams 0.8534 0.1701 5.02 0.0000005240752 ***

#y9_parent_demandsattention 0.3422 0.1303 2.63 0.00862 **

#y9_parent_phobias 1.4245 0.2040 6.98 0.0000000000029 ***

#y9_parent_accidentprone 1.0566 0.2550 4.14 0.0000343194397 ***

#y9_parent_getteased 0.6202 0.1996 3.11 0.00189 **

data.rose <- ROSE(autism ~ y9_parent_impulsive+y9_parent_nervous+

y9_parent_tempertantrums+y9_parent_troublesleeping+y9_parent_whines+y9_parent_withdrawn+

y9_parent_worries+y9_parent_makesfriendseasily+y9_parent_selfconfident+y9_parent_interestindiffthings+

y9_parent_invitesotherstohome+y9_parent_reportsaccappropr,

data = expl2, seed = 1)$data

lowbwt.bglm <- data.rose[, c("y9_parent_impulsive","y9_parent_nervous",

"y9_parent_tempertantrums","y9_parent_troublesleeping","y9_parent_whines","y9_parent_withdrawn",

"y9_parent_worries","y9_parent_makesfriendseasily","y9_parent_selfconfident","y9_parent_interestindiffthings",

"y9_parent_invitesotherstohome","y9_parent_reportsaccappropr","autism")]

names(lowbwt.bglm)[names(lowbwt.bglm) == "autism"] <- "y"

lowbwt.bglm <- as.data.frame(lowbwt.bglm)

best.logit <- bestglm(lowbwt.bglm,

IC = "AIC",

family=binomial,

method = "exhaustive")

summary(best.logit$BestModel)

#y9_parent_impulsive 1.2387 0.2068 5.99 0.000000002104 ***

#y9_parent_nervous 0.9422 0.2057 4.58 0.000004662796 ***

#y9_parent_tempertantrums 0.6620 0.1971 3.36 0.00078 ***

#y9_parent_troublesleeping 0.9326 0.2327 4.01 0.000061528108 ***

#y9_parent_whines 0.6880 0.1977 3.48 0.00050 ***

#y9_parent_withdrawn 0.7598 0.3881 1.96 0.05023 .

#y9_parent_worries 0.6003 0.1713 3.50 0.00046 ***

#y9_parent_makesfriendseasily -0.5771 0.1017 -5.67 0.000000014076 ***

#y9_parent_selfconfident -1.0368 0.1225 -8.47 < 0.0000000000000002 ***

#y9_parent_interestindiffthings -0.6882 0.1035 -6.65 0.000000000030 ***

#y9_parent_invitesotherstohome -0.7056 0.1074 -6.57 0.000000000049 ***

#y9_parent_reportsaccappropr -0.5262 0.1016 -5.18 0.000000222503 ***

### Accuracy

data.rose <- ROSE(autism ~ y9_parent_notlikedotherkids+y9_parent_anxious+y9_parent_clumsy+

y9_parent_compulsions+y9_parent_easydistracted+y9_parent_staresblankly+

y9_parent_daydreams+y9_parent_demandsattention+y9_parent_phobias+

y9_parent_accidentprone+y9_parent_getteased+y9_parent_impulsive+

y9_parent_nervous+y9_parent_tempertantrums+y9_parent_troublesleeping+

y9_parent_whines+y9_parent_worries +

y9_parent_makesfriendseasily +

y9_parent_selfconfident +

y9_parent_interestindiffthings+

y9_parent_invitesotherstohome +

y9_parent_reportsaccappropr+y9_parent_anxious+y9_parent_clumsy+y9_parent_compulsions+

y9_parent_easydistracted+y9_parent_staresblankly+y9_parent_daydreams+y9_parent_demandsattention+y9_parent_phobias+y9_parent_accidentprone+y9_parent_getteased+y9_parent_impulsive+y9_parent_nervous+

y9_parent_tempertantrums+y9_parent_troublesleeping+y9_parent_whines+y9_parent_worries+y9_parent_makesfriendseasily+y9_parent_selfconfident+y9_parent_invitesotherstohome +

y9_parent_reportsaccappropr,data = expl2, seed = 1)$data

fit <- glm(autism~y9_parent_notlikedotherkids+y9_parent_anxious+y9_parent_clumsy+

y9_parent_compulsions+y9_parent_easydistracted+y9_parent_staresblankly+

y9_parent_daydreams+y9_parent_demandsattention+y9_parent_phobias+

y9_parent_accidentprone+y9_parent_getteased+y9_parent_impulsive+

y9_parent_nervous+y9_parent_tempertantrums+y9_parent_troublesleeping+

y9_parent_whines+y9_parent_worries +

y9_parent_makesfriendseasily +

y9_parent_selfconfident +

y9_parent_interestindiffthings+

y9_parent_invitesotherstohome +

y9_parent_reportsaccappropr+y9_parent_anxious+y9_parent_clumsy+y9_parent_compulsions+

y9_parent_easydistracted+y9_parent_staresblankly+y9_parent_daydreams+y9_parent_demandsattention+y9_parent_phobias+y9_parent_accidentprone+y9_parent_getteased+y9_parent_impulsive+y9_parent_nervous+

y9_parent_tempertantrums+y9_parent_troublesleeping+y9_parent_whines+y9_parent_worries+y9_parent_makesfriendseasily+y9_parent_selfconfident+y9_parent_invitesotherstohome +

y9_parent_reportsaccappropr, data=data.rose, family=binomial)

## Accuracy

glm_probs <- data.frame(probs = predict(fit, newdata = asdimpholdout5,type="response"))

glm_pred <- glm_probs %>% mutate(pred = ifelse(probs>.5, "1", "0"))

glm_pred <- cbind(asdimpholdout5, glm_pred)

glm_pred %>% count(pred, autism) %>% spread(autism, n, fill = 0)

# Imp 1

# pred 0 1

#1 0 2206 21

#2 1 748 70

#Sensitivity

70/(70+21)*100 # 76.9%

#PPV

70/(748+70)*100 # 8.6%

# Imp 2

# pred 0 1

#1 0 2213 20

#2 1 741 71

#Sensitivity

71/(71+21)*100 # 77.2%

#PPV

71/(741+78)*100 # 8.7%

# Imp 3

# pred 0 1

#1 0 2183 21

#2 1 771 70

#Sensitivity

70/(70+21)*100 # 76.9%

#PPV

67/(771+67)*100 # 8.0%

# Imp 4

# pred 0 1

#1 0 2186 22

#2 1 768 69

#Sensitivity

69/(69+21)*100 # 76.7%

#PPV

69/(768+69)*100 # 8.2%

# Imp 5

# pred 0 1

#1 0 2205 21

#2 1 749 70

#Sensitivity

70/(70+21)*100 # 76.9%

#PPV

50/(749+50)*100 # 6.3%

#Pooled sensitivity

(76.9+77.2+76.9+76.7+76.9)/5 ## 76.9%

#Pooled PPV

(8.6+8.7+8.0+8.2+6.3)/5 ## 8.0%

##### Teacher-report

ry9_teacher_alotmoreactive_structuredplay <-logistf(autism~male+y9_teacher_alotmoreactive_structuredplay, data=expl2)

ry9_teacher_alotmoreactive_unstructuredplay <-logistf(autism~male+y9_teacher_alotmoreactive_unstructuredplay, data=expl2)

ry9_teacher_onlyattentionowninterests <-logistf(autism~male+y9_teacher_onlyattentionowninterests, data=expl2)

ry9_teacher_invitesotherinactivities <-logistf(autism~male+y9_teacher_invitesotherinactivities, data=expl2)

ry9_teacher_poorlanguage <-logistf(autism~male+y9_teacher_poorlanguage, data=expl2)

ry9_teacher_poorsocialstudies <-logistf(autism~male+y9_teacher_poorsocialstudies, data=expl2)

ry9_teacher_poormath <-logistf(autism~male+y9_teacher_poormath, data=expl2)

ry9_teacher_controltemper <-logistf(autism~male+y9_teacher_controltemper, data=expl2)

ry9_teacher_compromiseconflict <-logistf(autism~male+y9_teacher_compromiseconflict, data=expl2)

ry9_teacher_respondadeqpeerpress <-logistf(autism~male+y9_teacher_respondadeqpeerpress, data=expl2)

ry9_teacher_saynicethingsabself <-logistf(autism~male+y9_teacher_saynicethingsabself, data=expl2)

ry9_teacher_usefreetimeappropr <-logistf(autism~male+y9_teacher_usefreetimeappropr, data=expl2)

ry9_teacher_finishintime <-logistf(autism~male+y9_teacher_finishintime, data=expl2)

ry9_teacher_makesfriendseasy <-logistf(autism~male+y9_teacher_makesfriendseasy, data=expl2)

ry9_teacher_responapprteasing <-logistf(autism~male+y9_teacher_responapprteasing, data=expl2)

ry9_teacher_controlstemper <-logistf(autism~male+y9_teacher_controlstemper, data=expl2)

ry9_teacher_receivecritiquewell <-logistf(autism~male+y9_teacher_receivecritiquewell, data=expl2)

ry9_teacher_usetimeapprwaitforhelp <-logistf(autism~male+y9_teacher_usetimeapprwaitforhelp, data=expl2)

ry9_teacher_correctschoolwork <-logistf(autism~male+y9_teacher_correctschoolwork, data=expl2)

ry9_teacher_acceptpeersideas <-logistf(autism~male+y9_teacher_acceptpeersideas, data=expl2)

ry9_teacher_givescompliments <-logistf(autism~male+y9_teacher_givescompliments, data=expl2)

ry9_teacher_followsdirections <-logistf(autism~male+y9_teacher_followsdirections, data=expl2)

ry9_teacher_putawayschoolmaterial <-logistf(autism~male+y9_teacher_putawayschoolmaterial, data=expl2)

ry9_teacher_cooperatespeers <-logistf(autism~male+y9_teacher_cooperatespeers, data=expl2)

ry9_teacher_joingroupspontaneously <-logistf(autism~male+y9_teacher_joingroupspontaneously, data=expl2)

ry9_teacher_respadeqwhenpushed <-logistf(autism~male+y9_teacher_respadeqwhenpushed, data=expl2)

ry9_teacher_ignorepeerdistrinclass <-logistf(autism~male+y9_teacher_ignorepeerdistrinclass, data=expl2)

ry9_teacher_cleandesk <-logistf(autism~male+y9_teacher_cleandesk, data=expl2)

ry9_teacher_attendsinstruct <-logistf(autism~male+y9_teacher_attendsinstruct, data=expl2)

ry9_teacher_transitseasybtwactiv <-logistf(autism~male+y9_teacher_transitseasybtwactiv, data=expl2)

ry9_teacher_getalongdiffpeople <-logistf(autism~male+y9_teacher_getalongdiffpeople, data=expl2)

ry9_teacher_expressownfeelings <-logistf(autism~male+y9_teacher_expressownfeelings, data=expl2)

ry9_teacher_maintainsfriendships <-logistf(autism~male+y9_teacher_maintainsfriendships, data=expl2)

ry9_teacher_respectotherproperties <-logistf(autism~male+y9_teacher_respectotherproperties, data=expl2)

ry9_teacher_sensotherfeelings <-logistf(autism~male+y9_teacher_sensotherfeelings, data=expl2)

ry9_teacher_helpsothers <-logistf(autism~male+y9_teacher_helpsothers, data=expl2)

ry9_teacher_paysattention <-logistf(autism~male+y9_teacher_paysattention, data=expl2)

ry9_teacher_persiststasks <-logistf(autism~male+y9_teacher_persiststasks, data=expl2)

ry9_teacher_worksindependently <-logistf(autism~male+y9_teacher_worksindependently, data=expl2)

ry9_teacher_adaptschangeroutine <-logistf(autism~male+y9_teacher_adaptschangeroutine, data=expl2)

ry9_teacher_organizedbelongings <-logistf(autism~male+y9_teacher_organizedbelongings, data=expl2)

ry9_teacher_eagerlearnnewthings <-logistf(autism~male+y9_teacher_eagerlearnnewthings, data=expl2)

ry9_teacher_followclassroomrules <-logistf(autism~male+y9_teacher_followclassroomrules, data=expl2)

ry9_teacher_figthswothers <-logistf(autism~male+y9_teacher_figthswothers, data=expl2)

ry9_teacher_lowselfesteem <-logistf(autism~male+y9_teacher_lowselfesteem, data=expl2)

ry9_teacher_bulliesothers <-logistf(autism~male+y9_teacher_bulliesothers, data=expl2)

ry9_teacher_appearslonely <-logistf(autism~male+y9_teacher_appearslonely, data=expl2)

ry9_teacher_anxingroupchildren <-logistf(autism~male+y9_teacher_anxingroupchildren, data=expl2)

ry9_teacher_easyembarassed <-logistf(autism~male+y9_teacher_easyembarassed, data=expl2)

ry9_teacher_arguesothers <-logistf(autism~male+y9_teacher_arguesothers, data=expl2)

ry9_teacher_talkbackadults <-logistf(autism~male+y9_teacher_talkbackadults, data=expl2)

ry9_teacher_angryeasily <-logistf(autism~male+y9_teacher_angryeasily, data=expl2)

ry9_teacher_tempertantrums <-logistf(autism~male+y9_teacher_tempertantrums, data=expl2)

ry9_teacher_likesbeingalone <-logistf(autism~male+y9_teacher_likesbeingalone, data=expl2)

ry9_teacher_sad <-logistf(autism~male+y9_teacher_sad, data=expl2)

ry9_teacher_inattentive <-logistf(autism~male+y9_teacher_inattentive, data=expl2)

ry9_teacher_defiant <-logistf(autism~male+y9_teacher_defiant, data=expl2)

ry9_teacher_restless <-logistf(autism~male+y9_teacher_restless, data=expl2)

ry9_teacher_forgetwhatlearned <-logistf(autism~male+y9_teacher_forgetwhatlearned, data=expl2)

ry9_teacher_disturbothers <-logistf(autism~male+y9_teacher_disturbothers, data=expl2)

ry9_teacher_defiesadults <-logistf(autism~male+y9_teacher_defiesadults, data=expl2)

ry9_teacher_alwaysonthego <-logistf(autism~male+y9_teacher_alwaysonthego, data=expl2)

ry9_teacher_poorspelling <-logistf(autism~male+y9_teacher_poorspelling, data=expl2)

ry9_teacher_cannotremainstill <-logistf(autism~male+y9_teacher_cannotremainstill, data=expl2)

ry9_teacher_spiteful <-logistf(autism~male+y9_teacher_spiteful, data=expl2)

ry9_teacher_leavesseat <-logistf(autism~male+y9_teacher_leavesseat, data=expl2)

ry9_teacher_fidgethands <-logistf(autism~male+y9_teacher_fidgethands, data=expl2)

ry9_teacher_poorreading <-logistf(autism~male+y9_teacher_poorreading, data=expl2)

ry9_teacher_shortattentionspan <-logistf(autism~male+y9_teacher_shortattentionspan, data=expl2)

ry9_teacher_arguesadults <-logistf(autism~male+y9_teacher_arguesadults, data=expl2)

ry9_teacher_diffwaitturn <-logistf(autism~male+y9_teacher_diffwaitturn, data=expl2)

ry9_teacher_nointerestschool <-logistf(autism~male+y9_teacher_nointerestschool, data=expl2)

ry9_teacher_distractable <-logistf(autism~male+y9_teacher_distractable, data=expl2)

ry9_teacher_temperoutbursts <-logistf(autism~male+y9_teacher_temperoutbursts, data=expl2)

ry9_teacher_runsclimbs <-logistf(autism~male+y9_teacher_runsclimbs, data=expl2)

ry9_teacher_poorarithmetic <-logistf(autism~male+y9_teacher_poorarithmetic, data=expl2)

ry9_teacher_intrudesothers <-logistf(autism~male+y9_teacher_intrudesothers, data=expl2)

ry9_teacher_diffplayingquietly <-logistf(autism~male+y9_teacher_diffplayingquietly, data=expl2)

ry9_teacher_failstofinish <-logistf(autism~male+y9_teacher_failstofinish, data=expl2)

ry9_teacher_notfollowinstruct <-logistf(autism~male+y9_teacher_notfollowinstruct, data=expl2)

ry9_teacher_excitable <-logistf(autism~male+y9_teacher_excitable, data=expl2)

ry9_teacher_alwaysongo <-logistf(autism~male+y9_teacher_alwaysongo, data=expl2)

ry9_teacher_repeatedgrade <-logistf(autism~male+y9_teacher_repeatedgrade, data=expl2)

summary(ry9_teacher_alotmoreactive_structuredplay)#*

summary(ry9_teacher_alotmoreactive_unstructuredplay)#*

summary(ry9_teacher_onlyattentionowninterests)#***

summary(ry9_teacher_invitesotherinactivities)#***

summary(ry9_teacher_poorlanguage)#**

summary(ry9_teacher_poorsocialstudies)#***

summary(ry9_teacher_poormath)#***

summary(ry9_teacher_controltemper)#*

summary(ry9_teacher_compromiseconflict)#**

summary(ry9_teacher_respondadeqpeerpress)#**

summary(ry9_teacher_saynicethingsabself)#*

summary(ry9_teacher_usefreetimeappropr)

summary(ry9_teacher_finishintime)#*

summary(ry9_teacher_makesfriendseasy)#***

summary(ry9_teacher_responapprteasing)#***

summary(ry9_teacher_controlstemper)#***

summary(ry9_teacher_receivecritiquewell)#***

summary(ry9_teacher_usetimeapprwaitforhelp)#***

summary(ry9_teacher_correctschoolwork)

summary(ry9_teacher_acceptpeersideas)#***

summary(ry9_teacher_givescompliments)#*

summary(ry9_teacher_followsdirections)#*

summary(ry9_teacher_putawayschoolmaterial)#*

summary(ry9_teacher_cooperatespeers)#***

summary(ry9_teacher_joingroupspontaneously)#**

summary(ry9_teacher_respadeqwhenpushed)#**

summary(ry9_teacher_ignorepeerdistrinclass)#*

summary(ry9_teacher_cleandesk)#***

summary(ry9_teacher_attendsinstruct)#*

summary(ry9_teacher_transitseasybtwactiv)#**

summary(ry9_teacher_getalongdiffpeople)#***

summary(ry9_teacher_expressownfeelings)#**

summary(ry9_teacher_maintainsfriendships)#*

summary(ry9_teacher_respectotherproperties)

summary(ry9_teacher_sensotherfeelings)

summary(ry9_teacher_helpsothers)

summary(ry9_teacher_paysattention)

summary(ry9_teacher_persiststasks)

summary(ry9_teacher_worksindependently)

summary(ry9_teacher_adaptschangeroutine)#*

summary(ry9_teacher_organizedbelongings)

summary(ry9_teacher_eagerlearnnewthings)#*

summary(ry9_teacher_followclassroomrules)

summary(ry9_teacher_figthswothers)

summary(ry9_teacher_lowselfesteem)#**

summary(ry9_teacher_bulliesothers)

summary(ry9_teacher_appearslonely)#**

summary(ry9_teacher_anxingroupchildren)#***

summary(ry9_teacher_easyembarassed)

summary(ry9_teacher_arguesothers)#***

summary(ry9_teacher_talkbackadults)#***

summary(ry9_teacher_angryeasily)#***

summary(ry9_teacher_tempertantrums)#***

summary(ry9_teacher_likesbeingalone)#***

summary(ry9_teacher_sad)

summary(ry9_teacher_inattentive)#***

summary(ry9_teacher_defiant)#*

summary(ry9_teacher_restless)#**

summary(ry9_teacher_forgetwhatlearned)

summary(ry9_teacher_disturbothers)#**

summary(ry9_teacher_defiesadults)#*

summary(ry9_teacher_alwaysonthego)

summary(ry9_teacher_poorspelling)

summary(ry9_teacher_cannotremainstill)#**

summary(ry9_teacher_spiteful)

summary(ry9_teacher_leavesseat)#**

summary(ry9_teacher_fidgethands)#***

summary(ry9_teacher_poorreading)#***

summary(ry9_teacher_shortattentionspan)#***

summary(ry9_teacher_arguesadults)

summary(ry9_teacher_diffwaitturn)#***

summary(ry9_teacher_nointerestschool)

summary(ry9_teacher_distractable)#***

summary(ry9_teacher_temperoutbursts)#**

summary(ry9_teacher_runsclimbs)#*

summary(ry9_teacher_poorarithmetic)

summary(ry9_teacher_intrudesothers)#***

summary(ry9_teacher_diffplayingquietly)#***

summary(ry9_teacher_failstofinish)#**

summary(ry9_teacher_notfollowinstruct)

summary(ry9_teacher_excitable)#***

summary(ry9_teacher_alwaysongo)#**

summary(ry9_teacher_repeatedgrade)#*

CrossTable(expl2$autism, expl2$y9_teacher_onlyattentionowninterests)

CrossTable(expl2$autism, expl2$y9_teacher_invitesotherinactivities)

CrossTable(expl2$autism, expl2$y9_teacher_poorlanguage)

CrossTable(expl2$autism, expl2$y9_teacher_poorsocialstudies)

CrossTable(expl2$autism, expl2$y9_teacher_poormath)

CrossTable(expl2$autism, expl2$y9_teacher_compromiseconflict)

CrossTable(expl2$autism, expl2$y9_teacher_respondadeqpeerpress)

CrossTable(expl2$autism, expl2$y9_teacher_makesfriendseasy)

CrossTable(expl2$autism, expl2$y9_teacher_responapprteasing)

CrossTable(expl2$autism, expl2$y9_teacher_controlstemper)

CrossTable(expl2$autism, expl2$y9_teacher_receivecritiquewell)

CrossTable(expl2$autism, expl2$y9_teacher_usetimeapprwaitforhelp)

CrossTable(expl2$autism, expl2$y9_teacher_acceptpeersideas)

CrossTable(expl2$autism, expl2$y9_teacher_cooperatespeers)

CrossTable(expl2$autism, expl2$y9_teacher_joingroupspontaneously)

CrossTable(expl2$autism, expl2$y9_teacher_respadeqwhenpushed)

CrossTable(expl2$autism, expl2$y9_teacher_cleandesk)

CrossTable(expl2$autism, expl2$y9_teacher_transitseasybtwactiv)

CrossTable(expl2$autism, expl2$y9_teacher_getalongdiffpeople)

CrossTable(expl2$autism, expl2$y9_teacher_expressownfeelings)

CrossTable(expl2$autism, expl2$y9_teacher_lowselfesteem)

CrossTable(expl2$autism, expl2$y9_teacher_appearslonely)

CrossTable(expl2$autism, expl2$y9_teacher_anxingroupchildren)

CrossTable(expl2$autism, expl2$y9_teacher_arguesothers)

CrossTable(expl2$autism, expl2$y9_teacher_talkbackadults)

CrossTable(expl2$autism, expl2$y9_teacher_angryeasily)

CrossTable(expl2$autism, expl2$y9_teacher_tempertantrums)

CrossTable(expl2$autism, expl2$y9_teacher_likesbeingalone)

CrossTable(expl2$autism, expl2$y9_teacher_inattentive)

CrossTable(expl2$autism, expl2$y9_teacher_restless)

CrossTable(expl2$autism, expl2$y9_teacher_disturbothers)

CrossTable(expl2$autism, expl2$y9_teacher_cannotremainstill)

CrossTable(expl2$autism, expl2$y9_teacher_leavesseat)

CrossTable(expl2$autism, expl2$y9_teacher_fidgethands)

CrossTable(expl2$autism, expl2$y9_teacher_poorreading)

CrossTable(expl2$autism, expl2$y9_teacher_shortattentionspan)

CrossTable(expl2$autism, expl2$y9_teacher_diffwaitturn)

CrossTable(expl2$autism, expl2$y9_teacher_distractable)

CrossTable(expl2$autism, expl2$y9_teacher_temperoutbursts)

CrossTable(expl2$autism, expl2$y9_teacher_intrudesothers)

CrossTable(expl2$autism, expl2$y9_teacher_diffplayingquietly)

CrossTable(expl2$autism, expl2$y9_teacher_failstofinish)

CrossTable(expl2$autism, expl2$y9_teacher_excitable)

CrossTable(expl2$autism, expl2$y9_teacher_alwaysongo)

data.rose <- ROSE(autism ~ y9_teacher_onlyattentionowninterests+y9_teacher_invitesotherinactivities+y9_teacher_poorlanguage+y9_teacher_poorsocialstudies+

y9_teacher_poormath+y9_teacher_compromiseconflict+y9_teacher_respondadeqpeerpress+y9_teacher_makesfriendseasy+y9_teacher_responapprteasing+

y9_teacher_controlstemper+y9_teacher_receivecritiquewell+y9_teacher_usetimeapprwaitforhelp+y9_teacher_acceptpeersideas+y9_teacher_cooperatespeers+

y9_teacher_joingroupspontaneously,

data = expl2, seed = 1)$data

lowbwt.bglm <- data.rose[, c("y9_teacher_onlyattentionowninterests","y9_teacher_invitesotherinactivities","y9_teacher_poorlanguage","y9_teacher_poorsocialstudies",

"y9_teacher_poormath","y9_teacher_compromiseconflict","y9_teacher_respondadeqpeerpress","y9_teacher_makesfriendseasy","y9_teacher_responapprteasing",

"y9_teacher_controlstemper","y9_teacher_receivecritiquewell","y9_teacher_usetimeapprwaitforhelp","y9_teacher_acceptpeersideas","y9_teacher_cooperatespeers",

"y9_teacher_joingroupspontaneously","autism")]

names(lowbwt.bglm)[names(lowbwt.bglm) == "autism"] <- "y"

lowbwt.bglm <- as.data.frame(lowbwt.bglm)

best.logit <- bestglm(lowbwt.bglm,

IC = "AIC",

family=binomial,

method = "exhaustive")

summary(best.logit$BestModel)

#y9_teacher_onlyattentionowninterests 0.366 0.121 3.02 0.00253 **

#y9_teacher_poorsocialstudies 0.777 0.160 4.86 0.00000117461281479 ***

#y9_teacher_poormath 0.427 0.154 2.76 0.00570 **

#y9_teacher_makesfriendseasy -1.014 0.126 -8.06 0.00000000000000073 ***

#y9_teacher_responapprteasing -0.376 0.124 -3.04 0.00240 **

#y9_teacher_receivecritiquewell -0.788 0.124 -6.34 0.00000000022945794 ***

#y9_teacher_acceptpeersideas 0.268 0.122 2.20 0.02757 *

data.rose <- ROSE(autism ~ y9_teacher_respadeqwhenpushed+y9_teacher_cleandesk+y9_teacher_transitseasybtwactiv+y9_teacher_getalongdiffpeople+

y9_teacher_expressownfeelings+y9_teacher_lowselfesteem+y9_teacher_appearslonely+y9_teacher_anxingroupchildren+y9_teacher_arguesothers+y9_teacher_talkbackadults+

y9_teacher_angryeasily+y9_teacher_tempertantrums+y9_teacher_likesbeingalone+y9_teacher_inattentive+y9_teacher_restless,

data = expl2, seed = 1)$data

lowbwt.bglm <- data.rose[, c("y9_teacher_respadeqwhenpushed","y9_teacher_cleandesk","y9_teacher_transitseasybtwactiv","y9_teacher_getalongdiffpeople",

"y9_teacher_expressownfeelings","y9_teacher_lowselfesteem","y9_teacher_appearslonely","y9_teacher_anxingroupchildren","y9_teacher_arguesothers","y9_teacher_talkbackadults",

"y9_teacher_angryeasily","y9_teacher_tempertantrums","y9_teacher_likesbeingalone","y9_teacher_inattentive","y9_teacher_restless","autism")]

names(lowbwt.bglm)[names(lowbwt.bglm) == "autism"] <- "y"

lowbwt.bglm <- as.data.frame(lowbwt.bglm)

best.logit <- bestglm(lowbwt.bglm,

IC = "AIC",

family=binomial,

method = "exhaustive")

summary(best.logit$BestModel)

#y9_teacher_respadeqwhenpushed -0.376 0.126 -2.99 0.00277 **

#y9_teacher_cleandesk -0.742 0.124 -5.98 0.00000000218 ***

#y9_teacher_lowselfesteem 0.252 0.125 2.02 0.04354 *

#y9_teacher_appearslonely 0.213 0.127 1.68 0.09207 .

#y9_teacher_anxingroupchildren 0.476 0.131 3.64 0.00028 ***

#y9_teacher_arguesothers 0.680 0.140 4.85 0.00000126166 ***

#y9_teacher_talkbackadults 0.289 0.121 2.38 0.01727 *

#y9_teacher_angryeasily 0.463 0.126 3.69 0.00023 ***

#y9_teacher_likesbeingalone 0.558 0.125 4.47 0.00000767804 ***

#y9_teacher_inattentive 0.277 0.120 2.30 0.02150 *

data.rose <- ROSE(autism ~ y9_teacher_disturbothers+

y9_teacher_cannotremainstill+y9_teacher_leavesseat+y9_teacher_fidgethands+y9_teacher_poorreading+y9_teacher_shortattentionspan+y9_teacher_diffwaitturn+

y9_teacher_distractable+y9_teacher_temperoutbursts+y9_teacher_intrudesothers+y9_teacher_diffplayingquietly+y9_teacher_failstofinish+y9_teacher_excitable+

y9_teacher_alwaysongo,

data = expl2, seed = 1)$data

lowbwt.bglm <- data.rose[, c("y9_teacher_disturbothers","y9_teacher_cannotremainstill","y9_teacher_leavesseat","y9_teacher_fidgethands","y9_teacher_poorreading","y9_teacher_shortattentionspan","y9_teacher_diffwaitturn",

"y9_teacher_distractable","y9_teacher_temperoutbursts","y9_teacher_intrudesothers","y9_teacher_diffplayingquietly","y9_teacher_failstofinish","y9_teacher_excitable",

"y9_teacher_alwaysongo","autism")]

names(lowbwt.bglm)[names(lowbwt.bglm) == "autism"] <- "y"

lowbwt.bglm <- as.data.frame(lowbwt.bglm)

best.logit <- bestglm(lowbwt.bglm,

IC = "AIC",

family=binomial,

method = "exhaustive")

summary(best.logit$BestModel)

#y9_teacher_disturbothers -0.7520 0.1668 -4.51 0.0000065189 ***

#y9_teacher_fidgethands 0.4319 0.1419 3.04 0.00234 **

#y9_teacher_poorreading 0.5511 0.1117 4.93 0.0000008095 ***

#y9_teacher_shortattentionspan 0.4010 0.1313 3.05 0.00227 **

#y9_teacher_diffwaitturn 0.5377 0.1424 3.78 0.00016 ***

#y9_teacher_distractable 0.7326 0.1268 5.78 0.0000000075 ***

#y9_teacher_temperoutbursts 0.5426 0.1744 3.11 0.00186 **

#y9_teacher_diffplayingquietly -0.2586 0.1746 -1.48 0.13852

#y9_teacher_excitable 0.7874 0.1665 4.73 0.0000022677 ***

### Continue reducing

data.rose <- ROSE(autism ~ y9_teacher_onlyattentionowninterests+y9_teacher_poorsocialstudies+y9_teacher_poormath+y9_teacher_makesfriendseasy+y9_teacher_responapprteasing+y9_teacher_receivecritiquewell+

y9_teacher_respadeqwhenpushed+y9_teacher_cleandesk+y9_teacher_anxingroupchildren,

data = expl2, seed = 1)$data

lowbwt.bglm <- data.rose[, c("y9_teacher_onlyattentionowninterests","y9_teacher_poorsocialstudies","y9_teacher_poormath","y9_teacher_makesfriendseasy","y9_teacher_responapprteasing","y9_teacher_receivecritiquewell",

"y9_teacher_respadeqwhenpushed","y9_teacher_cleandesk","y9_teacher_anxingroupchildren","autism")]

names(lowbwt.bglm)[names(lowbwt.bglm) == "autism"] <- "y"

lowbwt.bglm <- as.data.frame(lowbwt.bglm)

best.logit <- bestglm(lowbwt.bglm,

IC = "AIC",

family=binomial,

method = "exhaustive")

summary(best.logit$BestModel)

#y9_teacher_poorsocialstudies 0.488 0.155 3.14 0.0017 **

#y9_teacher_makesfriendseasy -0.538 0.124 -4.35 0.00001368138 ***

#y9_teacher_responapprteasing -0.313 0.134 -2.33 0.0197 *

#y9_teacher_receivecritiquewell -0.777 0.124 -6.27 0.00000000037 ***

#y9_teacher_respadeqwhenpushed -0.393 0.120 -3.27 0.0011 **

#y9_teacher_cleandesk -0.661 0.120 -5.51 0.00000003560 ***

#y9_teacher_anxingroupchildren 0.644 0.121 5.30 0.00000011382 ***

data.rose <- ROSE(autism ~ y9_teacher_arguesothers+y9_teacher_angryeasily+y9_teacher_likesbeingalone+y9_teacher_disturbothers+y9_teacher_fidgethands+y9_teacher_poorreading+

y9_teacher_shortattentionspan+y9_teacher_diffwaitturn+y9_teacher_distractable+y9_teacher_temperoutbursts+y9_teacher_excitable,

data = expl2, seed = 1)$data

lowbwt.bglm <- data.rose[, c("y9_teacher_arguesothers","y9_teacher_angryeasily","y9_teacher_likesbeingalone","y9_teacher_disturbothers","y9_teacher_fidgethands","y9_teacher_poorreading",

"y9_teacher_shortattentionspan","y9_teacher_diffwaitturn","y9_teacher_distractable","y9_teacher_temperoutbursts","y9_teacher_excitable","autism")]

names(lowbwt.bglm)[names(lowbwt.bglm) == "autism"] <- "y"

lowbwt.bglm <- as.data.frame(lowbwt.bglm)

best.logit <- bestglm(lowbwt.bglm,

IC = "AIC",

family=binomial,

method = "exhaustive")

summary(best.logit$BestModel)

#y9_teacher_arguesothers 0.644 0.135 4.76 0.000001951554722 ***

#y9_teacher_angryeasily 0.764 0.123 6.19 0.000000000618825 ***

#y9_teacher_likesbeingalone 0.967 0.129 7.50 0.000000000000062 ***

#y9_teacher_disturbothers -0.738 0.186 -3.97 0.000071137239774 ***

#y9_teacher_fidgethands 0.502 0.158 3.18 0.00145 **

#y9_teacher_poorreading 0.490 0.121 4.05 0.000051723227509 ***

#y9_teacher_shortattentionspan 0.589 0.144 4.08 0.000044928070253 ***

#y9_teacher_diffwaitturn 1.085 0.169 6.44 0.000000000119962 ***

#y9_teacher_distractable 0.216 0.146 1.48 0.13905

#y9_teacher_temperoutbursts 0.905 0.238 3.81 0.00014 ***

#y9_teacher_excitable -0.269 0.187 -1.44 0.15047

### Accuracy

data.rose <- ROSE(autism ~ y9_teacher_poorsocialstudies+y9_teacher_makesfriendseasy+y9_teacher_receivecritiquewell+y9_teacher_respadeqwhenpushed+

y9_teacher_cleandesk+y9_teacher_anxingroupchildren+

y9_teacher_arguesothers+y9_teacher_angryeasily+y9_teacher_likesbeingalone+y9_teacher_disturbothers+

y9_teacher_fidgethands+y9_teacher_poorreading+y9_teacher_shortattentionspan+y9_teacher_temperoutbursts,

data = expl2, seed = 1)$data

fit <- glm(autism~y9_teacher_poorsocialstudies+y9_teacher_makesfriendseasy+y9_teacher_receivecritiquewell+y9_teacher_respadeqwhenpushed+

y9_teacher_cleandesk+y9_teacher_anxingroupchildren+

y9_teacher_arguesothers+y9_teacher_angryeasily+y9_teacher_likesbeingalone+y9_teacher_disturbothers+

y9_teacher_fidgethands+y9_teacher_poorreading+y9_teacher_shortattentionspan+y9_teacher_temperoutbursts, data=data.rose, family=binomial)

## Accuracy

glm_probs <- data.frame(probs = predict(fit, newdata = asdimpholdout4,type="response"))

glm_pred <- glm_probs %>% mutate(pred = ifelse(probs>.5, "1", "0"))

glm_pred <- cbind(asdimpholdout4, glm_pred)

glm_pred %>% count(pred, autism) %>% spread(autism, n, fill = 0)

# Imp 1

# pred 0 1

#1 0 1900 29

#2 1 1054 62

#Sensitivity

62/(62+29)*100 # 68.1%

#PPV

62/(1900+62)*100 # 3.2%

# Imp 2

# pred 0 1

#1 0 1831 28

#2 1 1123 63

#Sensitivity

63/(63+28)*100 # 69.2%

#PPV

63/(1123+63)*100 # 5.3%

# Imp 3

# pred 0 1

#0 1757 21

#2 1 1197 70

#Sensitivity

70/(70+21)*100 # 76.9%

#PPV

70/(1197+70)*100 # 5.5%

# Imp 4

# pred 0 1

#1 0 1932 29

#2 1 1022 62

#Sensitivity

62/(62+29)*100 # 68.1%

#PPV

62/(1022+62)*100 # 5.7%

# Imp 5

# pred 0 1

#1 0 2005 34

#2 1 949 57

#Sensitivity

57/(57+34)*100 # 62.6%

#PPV

57/(949+57)*100 # 5.6%

#Pooled sensitivity

(68.1+69.2+76.9+68.1+62.6)/5 ## 69.0%

#Pooled PPV

(3.2+5.3+5.5+5.7+5.6)/5 ## 5.1%

##### Year 9 - Self-Reported Factors

ry9_kid_extraschoolhelpmorethan2hours <- logistf(autism~male+y9_kid_extraschoolhelpmorethan2hours, data=expl2)

ry9_kid_notfeelclosepeopleatschool <- logistf(autism~male+y9_kid_notfeelclosepeopleatschool, data=expl2)

ry9_kid_kidstakemymoneyinschool <- logistf(autism~male+y9_kid_kidstakemymoneyinschool, data=expl2)

ry9_kid_neverstaytaskuntilsolved <- logistf(autism~male+y9_kid_neverstaytaskuntilsolved, data=expl2)

ry9_kid_neverwanttosolvehardtask <- logistf(autism~male+y9_kid_neverwanttosolvehardtask, data=expl2)

ry9_kid_angrywtroublelearning <- logistf(autism~male+y9_kid_angrywtroublelearning, data=expl2)

ry9_kid_neverhangfriends <- logistf(autism~male+y9_kid_neverhangfriends, data=expl2)

ry9_kid_neverhelphome <- logistf(autism~male+y9_kid_neverhelphome, data=expl2)

ry9_kid_sptimefamless30min <- logistf(autism~male+y9_kid_sptimefamless30min, data=expl2)

ry9_kid_gamingmorethan4hours <- logistf(autism~male+y9_kid_gamingmorethan4hours, data=expl2)

ry9_kid_tvmorethan4hours <- logistf(autism~male+y9_kid_tvmorethan4hours, data=expl2)

ry9_kid_notfeelpartofschool <- logistf(autism~male+y9_kid_notfeelpartofschool, data=expl2)

ry9_kid_nohappyatschool <- logistf(autism~male+y9_kid_nohappyatschool, data=expl2)

ry9_kid_notsafeatschool <- logistf(autism~male+y9_kid_notsafeatschool, data=expl2)

ry9_kid_pickedoninschool <- logistf(autism~male+y9_kid_pickedoninschool, data=expl2)

ry9_kid_beateninschool <- logistf(autism~male+y9_kid_beateninschool, data=expl2)

ry9_kid_leftoutinschool <- logistf(autism~male+y9_kid_leftoutinschool, data=expl2)

ry9_kid_damagedproperty <- logistf(autism~male+y9_kid_damagedproperty, data=expl2)

ry9_kid_stolensmthng <- logistf(autism~male+y9_kid_stolensmthng, data=expl2)

ry9_kid_takenmoneyhome <- logistf(autism~male+y9_kid_takenmoneyhome, data=expl2)

ry9_kid_cheatedschool <- logistf(autism~male+y9_kid_cheatedschool, data=expl2)

ry9_kid_fistfight <- logistf(autism~male+y9_kid_fistfight, data=expl2)

ry9_kid_hurtanimalpurpose <- logistf(autism~male+y9_kid_hurtanimalpurpose, data=expl2)

ry9_kid_enteredotherprpoerty <- logistf(autism~male+y9_kid_enteredotherprpoerty, data=expl2)

ry9_kid_runawayhome <- logistf(autism~male+y9_kid_runawayhome, data=expl2)

ry9_kid_skippedschool <- logistf(autism~male+y9_kid_skippedschool, data=expl2)

ry9_kid_drinking <- logistf(autism~male+y9_kid_drinking, data=expl2)

ry9_kid_weed <- logistf(autism~male+y9_kid_weed, data=expl2)

ry9_kid_cigarettes <- logistf(autism~male+y9_kid_cigarettes, data=expl2)

ry9_kid_suspendedschool <- logistf(autism~male+y9_kid_suspendedschool, data=expl2)

ry9_kid_grafitti <- logistf(autism~male+y9_kid_grafitti, data=expl2)

ry9_kid_setfire <- logistf(autism~male+y9_kid_setfire, data=expl2)

ry9_kid_freeriding <- logistf(autism~male+y9_kid_freeriding, data=expl2)

ry9_kid_rockatpeopleorcars <- logistf(autism~male+y9_kid_rockatpeopleorcars, data=expl2)

ry9_kid_neverorderly <- logistf(autism~male+y9_kid_neverorderly, data=expl2)

ry9_kid_notdobest <- logistf(autism~male+y9_kid_notdobest, data=expl2)

ry9_kid_notfollowthrough <- logistf(autism~male+y9_kid_notfollowthrough, data=expl2)

ry9_kid_arguewothers <- logistf(autism~male+y9_kid_arguewothers, data=expl2)

ry9_kid_worrytests <- logistf(autism~male+y9_kid_worrytests, data=expl2)

ry9_kid_hardpayattention <- logistf(autism~male+y9_kid_hardpayattention, data=expl2)

ry9_kid_feellonely <- logistf(autism~male+y9_kid_feellonely, data=expl2)

ry9_kid_easilydistracted <- logistf(autism~male+y9_kid_easilydistracted, data=expl2)

ry9_kid_sad <- logistf(autism~male+y9_kid_sad, data=expl2)

ry9_kid_hardfinishschoolwork <- logistf(autism~male+y9_kid_hardfinishschoolwork, data=expl2)

ry9_kid_worrydoingwellschool <- logistf(autism~male+y9_kid_worrydoingwellschool, data=expl2)

ry9_kid_worryfinishwork <- logistf(autism~male+y9_kid_worryfinishwork, data=expl2)

ry9_kid_worrynoonetoplay <- logistf(autism~male+y9_kid_worrynoonetoplay, data=expl2)

ry9_kid_smahemistakesschool <- logistf(autism~male+y9_kid_smahemistakesschool, data=expl2)

ry9_kid_distrubingothers <- logistf(autism~male+y9_kid_distrubingothers, data=expl2)

ry9_kid_fightingothers <- logistf(autism~male+y9_kid_fightingothers, data=expl2)

ry9_kid_poororfairhealth <- logistf(autism~male+y9_kid_poororfairhealth, data=expl2)

ry9_kid_seldomseatbelt <- logistf(autism~male+y9_kid_seldomseatbelt, data=expl2)

summary(ry9_kid_extraschoolhelpmorethan2hours)

summary(ry9_kid_notfeelclosepeopleatschool)

summary(ry9_kid_kidstakemymoneyinschool)#*

summary(ry9_kid_neverstaytaskuntilsolved)

summary(ry9_kid_neverwanttosolvehardtask)

summary(ry9_kid_angrywtroublelearning)#**

summary(ry9_kid_neverhangfriends)

summary(ry9_kid_neverhelphome)#**

summary(ry9_kid_sptimefamless30min)

summary(ry9_kid_gamingmorethan4hours)

summary(ry9_kid_tvmorethan4hours)#**

summary(ry9_kid_notfeelpartofschool)

summary(ry9_kid_nohappyatschool)

summary(ry9_kid_notsafeatschool)

summary(ry9_kid_pickedoninschool)#**

summary(ry9_kid_beateninschool)#*

summary(ry9_kid_leftoutinschool)

summary(ry9_kid_damagedproperty)

summary(ry9_kid_stolensmthng)

summary(ry9_kid_takenmoneyhome)#*

summary(ry9_kid_cheatedschool)

summary(ry9_kid_fistfight)

summary(ry9_kid_hurtanimalpurpose)

summary(ry9_kid_enteredotherprpoerty)

summary(ry9_kid_runawayhome)#**

summary(ry9_kid_skippedschool)#*

summary(ry9_kid_drinking)

summary(ry9_kid_weed)

summary(ry9_kid_cigarettes)#*

summary(ry9_kid_suspendedschool)

summary(ry9_kid_grafitti)

summary(ry9_kid_setfire)#**

summary(ry9_kid_freeriding)

summary(ry9_kid_rockatpeopleorcars)

summary(ry9_kid_neverorderly)#*

summary(ry9_kid_notdobest)#**

summary(ry9_kid_notfollowthrough)#*

summary(ry9_kid_arguewothers)

summary(ry9_kid_worrytests)

summary(ry9_kid_hardpayattention)#**

summary(ry9_kid_feellonely)#*

summary(ry9_kid_easilydistracted)#***

summary(ry9_kid_sad)

summary(ry9_kid_hardfinishschoolwork)

summary(ry9_kid_worrydoingwellschool)

summary(ry9_kid_worryfinishwork)

summary(ry9_kid_worrynoonetoplay)

summary(ry9_kid_smahemistakesschool)

summary(ry9_kid_distrubingothers)

summary(ry9_kid_fightingothers)

summary(ry9_kid_poororfairhealth)#*

summary(ry9_kid_seldomseatbelt)#**

CrossTable(expl2$autism, expl2$y9_kid_angrywtroublelearning)

CrossTable(expl2$autism, expl2$y9_kid_neverhelphome)

CrossTable(expl2$autism, expl2$y9_kid_tvmorethan4hours)

CrossTable(expl2$autism, expl2$y9_kid_pickedoninschool)

CrossTable(expl2$autism, expl2$y9_kid_runawayhome)

CrossTable(expl2$autism, expl2$y9_kid_setfire)

CrossTable(expl2$autism, expl2$y9_kid_notdobest)

CrossTable(expl2$autism, expl2$y9_kid_hardpayattention)

CrossTable(expl2$autism, expl2$y9_kid_easilydistracted)

CrossTable(expl2$autism, expl2$y9_kid_seldomseatbelt)

data.rose <- ROSE(autism ~ y9_kid_angrywtroublelearning+y9_kid_neverhelphome+y9_kid_tvmorethan4hours+y9_kid_pickedoninschool+

y9_kid_runawayhome+y9_kid_setfire+y9_kid_notdobest+y9_kid_hardpayattention+y9_kid_easilydistracted+y9_kid_seldomseatbelt,

data = expl2, seed = 1)$data

lowbwt.bglm <- data.rose[, c("y9_kid_angrywtroublelearning","y9_kid_neverhelphome","y9_kid_tvmorethan4hours","y9_kid_pickedoninschool",

"y9_kid_runawayhome","y9_kid_setfire","y9_kid_notdobest","y9_kid_hardpayattention","y9_kid_easilydistracted","y9_kid_seldomseatbelt","autism")]

names(lowbwt.bglm)[names(lowbwt.bglm) == "autism"] <- "y"

lowbwt.bglm <- as.data.frame(lowbwt.bglm)

best.logit <- bestglm(lowbwt.bglm,

IC = "AIC",

family=binomial,

method = "exhaustive")

summary(best.logit$BestModel)

#y9_kid_angrywtroublelearning 0.1892 0.1122 1.69 0.0919 .

#y9_kid_neverhelphome 0.7897 0.1151 6.86 0.0000000000067 ***

#y9_kid_tvmorethan4hours -0.6872 0.0991 -6.94 0.0000000000040 ***

#y9_kid_pickedoninschool 0.6184 0.0940 6.58 0.0000000000478 ***

#y9_kid_runawayhome 0.5495 0.2183 2.52 0.0118 *

#y9_kid_setfire 1.1379 0.2010 5.66 0.0000000149214 ***

#y9_kid_notdobest 0.8340 0.1977 4.22 0.0000245702585 ***

#y9_kid_hardpayattention 0.3891 0.1272 3.06 0.0022 **

#y9_kid_easilydistracted 0.3164 0.1048 3.02 0.0025 **

#y9_kid_seldomseatbelt 0.8981 0.1504 5.97 0.0000000023762 ***

## Accuracy

glm_probs <- data.frame(probs = predict(best.logit$BestModel, newdata = asdimpholdout5,type="response"))

glm_pred <- glm_probs %>% mutate(pred = ifelse(probs>.5, "1", "0"))

glm_pred <- cbind(asdimpholdout5, glm_pred)

glm_pred %>% count(pred, autism) %>% spread(autism, n, fill = 0)

# Imp 1

# pred 0 1

#1 0 2183 28

#2 1 775 63

#Sensitivity

63/(63+28)*100 # 69.2%

#PPV

63/(775+63)*100 # 7.5%

# Imp 2

# pred 0 1

#1 0 2183 28

#2 1 771 63

#Sensitivity

63/(63+28)*100 # 69.2%

#PPV

63/(771+63)*100 # 7.6%

# Imp 3

# pred 0 1

#1 0 2169 29

#2 1 785 62

#Sensitivity

62/(62+29)*100 # 68.1%

#PPV

62/(785+62)*100 # 7.3%

# Imp 4

# pred 0 1

#1 0 2169 29

#2 1 785 62

#Sensitivity

62/(62+29)*100 # 68.1%

#PPV

62/(785+62)*100 # 7.3%

# Imp 5

# pred 0 1

#1 0 2169 29

#2 1 778 62

#Sensitivity

62/(62+29)*100 # 68.1%

#PPV

62/(778+62)*100 # 7.4%

#Pooled sensitivity

(69.2+69.2+68.1+68.1+68.1)/5 ## 68.5%

#Pooled PPV

(7.5+7.6+7.3+7.3+7.4)/5 ## 7.4%

### Test scores

# Year 3

rvocaby3 <- logistf(autism ~ male + ch3ppvtraw, data = expl2)

summary(rvocaby3) # * Not under .01

# Year 5

rvocaby5 <- logistf(autism ~ male + ch4ppvtraw, data = expl2)

rleiteratt5 <- logistf(autism ~ male + ch4lr_corscor, data = expl2)

rleiterimp5 <- logistf(autism ~ male + ch4lr_errscor, data = expl2)

summary(rvocaby5) # ***

summary(rleiteratt5) # ***

summary(rleiterimp5) # ***

# Year 9

rvocaby9 <- logistf(autism ~ male + ch5ppvtraw, data = expl2)

rwoodcock9 <- logistf(autism ~ male + ch5wj9raw, data = expl2)

rwoodcock10 <- logistf(autism ~ male + ch5wj10raw, data = expl2)

rdigitspan <- logistf(autism ~ male + ch5dsraw, data = expl2)

summary(rvocaby9) # ***

summary(rwoodcock9) # ***

summary(rwoodcock10) # ***

summary(rdigitspan) # ***

aggregate(ch4ppvtraw ~ autism, expl2, mean)

aggregate(ch4ppvtraw ~ autism, expl2, sd)

# Vocabulary year 5

# Mean SD

# no autism 62.58093 19.04591

# autism 51.45946 22.16177

aggregate(ch4lr_corscor ~ autism, expl2, mean)

aggregate(ch4lr_corscor ~ autism, expl2, sd)

# Leiter attention year 5

# Mean SD

# no autism 12.95188 3.270419

# autism 10.33333 3.186510

aggregate(ch4lr_errscor ~ autism, expl2, mean)

aggregate(ch4lr_errscor ~ autism, expl2, sd)

# Leiter impulsivity year 5

# Mean SD

# no autism 10.155791 2.839265

# autism 9.481481 3.588125

aggregate(ch5ppvtraw ~ autism, expl2, mean)

aggregate(ch5ppvtraw ~ autism, expl2, sd)

# Vocabulary year 9

# Mean SD

# no autism 111.9657 20.0901

# autism 104.8980 28.9556

aggregate(ch5wj9raw ~ autism, expl2, mean)

aggregate(ch5wj9raw ~ autism, expl2, sd)

# Language W-J year 9

# Mean SD

# no autism 26.00533 5.443590

# autism 22.14000 8.131596

aggregate(ch5wj10raw ~ autism, expl2, mean)

aggregate(ch5wj10raw ~ autism, expl2, sd)

# Math W-J year 9

# Mean SD

# no autism 32.65121 5.918578

# autism 26.86000 9.396070

aggregate(ch5dsraw ~ autism, expl2, mean)

aggregate(ch5dsraw ~ autism, expl2, sd)

# Working Mem WISV year 9

# Mean SD

# no autism 14.09679 3.094737

# autism 11.32692 4.176128

#### Select best variables

data.rose <- ROSE(autism ~ ch4ppvtraw+ch4lr_corscor+ch4lr_errscor+ch5ppvtraw+ch5wj9raw+ch5wj10raw+ch5dsraw,

data = expl2, seed = 1)$data

# Run bestglm to find best subset model. Use syntehetic data to get better coefficients to find cases

lowbwt.bglm <- data.rose[, c("ch4ppvtraw","ch4lr_corscor","ch4lr_errscor",

"ch5ppvtraw","ch5wj9raw","ch5wj10raw","ch5dsraw","autism")]

names(lowbwt.bglm)[names(lowbwt.bglm) == "autism"] <- "y"

lowbwt.bglm <- as.data.frame(lowbwt.bglm)

best.logit <- bestglm(lowbwt.bglm,

IC = "AIC",

family=binomial,

method = "exhaustive")

summary(best.logit$BestModel)

# ch4ppvtraw -0.019069 0.003475 -5.487 4.08e-08 *** Vocab year 5

# ch4lr_corscor -0.157175 0.019538 -8.045 8.65e-16 *** Att year 5

# ch4lr_errscor -0.073789 0.018690 -3.948 7.88e-05 *** Imp year 5

# ch5ppvtraw 0.017438 0.003030 5.756 8.63e-09 *** Vocab year 9

# ch5wj10raw -0.084211 0.012897 -6.529 6.60e-11 *** Math year 9

# ch5dsraw -0.077519 0.023347 -3.320 0.000899 *** Working mem year 9

#### Add to tecaher year 5

data.rose <- ROSE(autism ~ y5_teacher_diagnoseddisability+

y5_teacher_understconvofprint+

y5_teacher_sortscompmath+

y5_teacher_varietyinmath+

y5_teacher_belowaverlanguage+

y5_teacher_belowavermath+

y5_teacher_discussproblsparents+

ch4ppvtraw+

ch4lr_corscor+

ch4lr_errscor,

data = expl2, seed = 1)$data

lowbwt.bglm <- data.rose[, c("y5_teacher_diagnoseddisability",

"y5_teacher_understconvofprint",

"y5_teacher_sortscompmath",

"y5_teacher_varietyinmath",

"y5_teacher_belowaverlanguage",

"y5_teacher_belowavermath",

"y5_teacher_discussproblsparents",

"ch4ppvtraw",

"ch4lr_corscor",

"ch4lr_errscor",

"autism")]

names(lowbwt.bglm)[names(lowbwt.bglm) == "autism"] <- "y"

lowbwt.bglm <- as.data.frame(lowbwt.bglm)

best.logit <- bestglm(lowbwt.bglm,

IC = "AIC",

family=binomial,

method = "exhaustive")

summary(best.logit$BestModel)

#y5_teacher_diagnoseddisability 1.487056 0.288886 5.148 2.64e-07 ***

#y5_teacher_understconvofprint 1.062628 0.225776 4.707 2.52e-06 ***

#y5_teacher_varietyinmath 1.050596 0.256060 4.103 4.08e-05 ***

#y5_teacher_discussproblsparents 0.896749 0.234119 3.830 0.000128 ***

#ch4ppvtraw -0.008607 0.006031 -1.427 0.153536

#ch4lr_corscor -0.122034 0.032776 -3.723 0.000197 ***

##### Attention problems at age 5 should be included (ch4lr_corscor)

### Teacher year 9

data.rose <- ROSE(autism ~ y9_teacher_arguesothers+y9_teacher_angryeasily+y9_teacher_likesbeingalone+y9_teacher_disturbothers+y9_teacher_fidgethands+y9_teacher_poorreading+

y9_teacher_shortattentionspan+y9_teacher_diffwaitturn+y9_teacher_distractable+y9_teacher_temperoutbursts+y9_teacher_excitable+ch5ppvtraw+ch5ppvtraw+ch5dsraw,

data = expl2, seed = 1)$data

lowbwt.bglm <- data.rose[, c("y9_teacher_arguesothers","y9_teacher_angryeasily","y9_teacher_likesbeingalone","y9_teacher_disturbothers","y9_teacher_fidgethands","y9_teacher_poorreading",

"y9_teacher_shortattentionspan","y9_teacher_diffwaitturn","y9_teacher_distractable","y9_teacher_temperoutbursts","y9_teacher_excitable",

"ch5ppvtraw","ch5ppvtraw","ch5dsraw","autism")]

names(lowbwt.bglm)[names(lowbwt.bglm) == "autism"] <- "y"

lowbwt.bglm <- as.data.frame(lowbwt.bglm)

best.logit <- bestglm(lowbwt.bglm,

IC = "AIC",

family=binomial,

method = "exhaustive")

summary(best.logit$BestModel)

# y9_teacher_angryeasily 0.58904 0.11628 5.066 4.07e-07 ***

# y9_teacher_likesbeingalone 0.84139 0.12298 6.841 7.84e-12 ***

# y9_teacher_disturbothers -0.59426 0.18101 -3.283 0.001027 **

# y9_teacher_fidgethands 0.22630 0.15597 1.451 0.146816

# y9_teacher_poorreading 0.51919 0.11518 4.508 6.56e-06 ***

# y9_teacher_diffwaitturn 0.68155 0.17024 4.004 6.24e-05 ***

# y9_teacher_distractable 0.45798 0.13693 3.345 0.000824 ***

# y9_teacher_temperoutbursts 0.50605 0.20369 2.484 0.012977 *

# y9_teacher_excitable 0.46680 0.18855 2.476 0.013298 *

# ch5dsraw -0.06668 0.01548 -4.307 1.66e-05 ***

##### Working memory at age 9 should be included (ch5dsraw)

################################################################################

##############

############## Final model

##############

################################################################################

### Find significant variables across imputed datasets using synthesized data

explimp1 <- read_sav("Data/imputations asd/asdimpexpl1.sav")

explimp2 <- read_sav("Data/imputations asd/asdimpexpl2.sav")

explimp3 <- read_sav("Data/imputations asd/asdimpexpl3.sav")

explimp4 <- read_sav("Data/imputations asd/asdimpexpl4.sav")

explimp5 <- read_sav("Data/imputations asd/asdimpexpl5.sav")

data.rose1 <- ROSE(autism ~ male+fampsych_anx_mside+y1_mother_dep+y1_overnighthosp+y3_physdisability+y3_speechprblm+y5_over2earinf+

y5_speechproblem+y9_phys_speechproblem+y9_phys_stuttering+y9_over2earinf+y9_parent_unknownphys_pains+y9_parent_constipated+

y3_playswithothers+y3_tooyoung+y3_cantconcentr+y3_defiant+y3_demandsmetdirect+y3_disobed+y3_distrbdchroutine+y3_noguilt+

y3_easilyfrustr+y3_overtired+y3_notsenstowpunish+y3_withdrawn+y5_treatenspeople+y5_cantconcentrate+y5_troublefallingasleep+

y5_nervous+y5_stubborn+y5_actstooyoungforage+y5_ratherbealone+y5_nervoustwitches+y5_clumsy+y5_playstalkothers+

y5_teacher_diagnoseddisability+y5_teacher_understconvofprint+y5_teacher_discussproblsparents+y9_parent_notlikedotherkids+

y9_parent_anxious+y9_parent_clumsy+y9_parent_compulsions+y9_parent_easydistracted+y9_parent_staresblankly+y9_parent_daydreams+

y9_parent_demandsattention+y9_parent_phobias+y9_parent_accidentprone+y9_parent_getteased+y9_parent_impulsive+y9_parent_nervous+

y9_parent_tempertantrums+y9_parent_troublesleeping+y9_parent_whines+y9_parent_worries+y9_parent_makesfriendseasily+

y9_parent_selfconfident+y9_parent_interestindiffthings+y9_parent_invitesotherstohome+y9_parent_reportsaccappropr+

y9_teacher_poorsocialstudies+y9_teacher_makesfriendseasy+y9_teacher_receivecritiquewell+y9_teacher_respadeqwhenpushed+

y9_teacher_cleandesk+y9_teacher_anxingroupchildren+y9_teacher_arguesothers+y9_teacher_angryeasily+y9_teacher_likesbeingalone+

y9_teacher_disturbothers+y9_teacher_fidgethands+y9_teacher_poorreading+y9_teacher_shortattentionspan+y9_teacher_temperoutbursts+

y9_kid_neverhelphome+y9_kid_tvmorethan4hours+y9_kid_pickedoninschool+y9_kid_setfire+y9_kid_notdobest+y9_kid_hardpayattention+

y9_kid_easilydistracted+y9_kid_seldomseatbelt,

data = explimp1, seed = 1)$data

data.rose2 <- ROSE(autism ~ male+fampsych_anx_mside+y1_mother_dep+y1_overnighthosp+y3_physdisability+y3_speechprblm+y5_over2earinf+

y5_speechproblem+y9_phys_speechproblem+y9_phys_stuttering+y9_over2earinf+y9_parent_unknownphys_pains+y9_parent_constipated+

y3_playswithothers+y3_tooyoung+y3_cantconcentr+y3_defiant+y3_demandsmetdirect+y3_disobed+y3_distrbdchroutine+y3_noguilt+

y3_easilyfrustr+y3_overtired+y3_notsenstowpunish+y3_withdrawn+y5_treatenspeople+y5_cantconcentrate+y5_troublefallingasleep+

y5_nervous+y5_stubborn+y5_actstooyoungforage+y5_ratherbealone+y5_nervoustwitches+y5_clumsy+y5_playstalkothers+

y5_teacher_diagnoseddisability+y5_teacher_understconvofprint+y5_teacher_discussproblsparents+y9_parent_notlikedotherkids+

y9_parent_anxious+y9_parent_clumsy+y9_parent_compulsions+y9_parent_easydistracted+y9_parent_staresblankly+y9_parent_daydreams+

y9_parent_demandsattention+y9_parent_phobias+y9_parent_accidentprone+y9_parent_getteased+y9_parent_impulsive+y9_parent_nervous+

y9_parent_tempertantrums+y9_parent_troublesleeping+y9_parent_whines+y9_parent_worries+y9_parent_makesfriendseasily+

y9_parent_selfconfident+y9_parent_interestindiffthings+y9_parent_invitesotherstohome+y9_parent_reportsaccappropr+

y9_teacher_poorsocialstudies+y9_teacher_makesfriendseasy+y9_teacher_receivecritiquewell+y9_teacher_respadeqwhenpushed+

y9_teacher_cleandesk+y9_teacher_anxingroupchildren+y9_teacher_arguesothers+y9_teacher_angryeasily+y9_teacher_likesbeingalone+

y9_teacher_disturbothers+y9_teacher_fidgethands+y9_teacher_poorreading+y9_teacher_shortattentionspan+y9_teacher_temperoutbursts+

y9_kid_neverhelphome+y9_kid_tvmorethan4hours+y9_kid_pickedoninschool+y9_kid_setfire+y9_kid_notdobest+y9_kid_hardpayattention+

y9_kid_easilydistracted+y9_kid_seldomseatbelt,

data = explimp2, seed = 1)$data

data.rose3 <- ROSE(autism ~ male+fampsych_anx_mside+y1_mother_dep+y1_overnighthosp+y3_physdisability+y3_speechprblm+y5_over2earinf+

y5_speechproblem+y9_phys_speechproblem+y9_phys_stuttering+y9_over2earinf+y9_parent_unknownphys_pains+y9_parent_constipated+

y3_playswithothers+y3_tooyoung+y3_cantconcentr+y3_defiant+y3_demandsmetdirect+y3_disobed+y3_distrbdchroutine+y3_noguilt+

y3_easilyfrustr+y3_overtired+y3_notsenstowpunish+y3_withdrawn+y5_treatenspeople+y5_cantconcentrate+y5_troublefallingasleep+

y5_nervous+y5_stubborn+y5_actstooyoungforage+y5_ratherbealone+y5_nervoustwitches+y5_clumsy+y5_playstalkothers+

y5_teacher_diagnoseddisability+y5_teacher_understconvofprint+y5_teacher_discussproblsparents+y9_parent_notlikedotherkids+

y9_parent_anxious+y9_parent_clumsy+y9_parent_compulsions+y9_parent_easydistracted+y9_parent_staresblankly+y9_parent_daydreams+

y9_parent_demandsattention+y9_parent_phobias+y9_parent_accidentprone+y9_parent_getteased+y9_parent_impulsive+y9_parent_nervous+

y9_parent_tempertantrums+y9_parent_troublesleeping+y9_parent_whines+y9_parent_worries+y9_parent_makesfriendseasily+

y9_parent_selfconfident+y9_parent_interestindiffthings+y9_parent_invitesotherstohome+y9_parent_reportsaccappropr+

y9_teacher_poorsocialstudies+y9_teacher_makesfriendseasy+y9_teacher_receivecritiquewell+y9_teacher_respadeqwhenpushed+

y9_teacher_cleandesk+y9_teacher_anxingroupchildren+y9_teacher_arguesothers+y9_teacher_angryeasily+y9_teacher_likesbeingalone+

y9_teacher_disturbothers+y9_teacher_fidgethands+y9_teacher_poorreading+y9_teacher_shortattentionspan+y9_teacher_temperoutbursts+

y9_kid_neverhelphome+y9_kid_tvmorethan4hours+y9_kid_pickedoninschool+y9_kid_setfire+y9_kid_notdobest+y9_kid_hardpayattention+

y9_kid_easilydistracted+y9_kid_seldomseatbelt,

data = explimp3, seed = 1)$data

data.rose4 <- ROSE(autism ~ male+fampsych_anx_mside+y1_mother_dep+y1_overnighthosp+y3_physdisability+y3_speechprblm+y5_over2earinf+

y5_speechproblem+y9_phys_speechproblem+y9_phys_stuttering+y9_over2earinf+y9_parent_unknownphys_pains+y9_parent_constipated+

y3_playswithothers+y3_tooyoung+y3_cantconcentr+y3_defiant+y3_demandsmetdirect+y3_disobed+y3_distrbdchroutine+y3_noguilt+

y3_easilyfrustr+y3_overtired+y3_notsenstowpunish+y3_withdrawn+y5_treatenspeople+y5_cantconcentrate+y5_troublefallingasleep+

y5_nervous+y5_stubborn+y5_actstooyoungforage+y5_ratherbealone+y5_nervoustwitches+y5_clumsy+y5_playstalkothers+

y5_teacher_diagnoseddisability+y5_teacher_understconvofprint+y5_teacher_discussproblsparents+y9_parent_notlikedotherkids+

y9_parent_anxious+y9_parent_clumsy+y9_parent_compulsions+y9_parent_easydistracted+y9_parent_staresblankly+y9_parent_daydreams+

y9_parent_demandsattention+y9_parent_phobias+y9_parent_accidentprone+y9_parent_getteased+y9_parent_impulsive+y9_parent_nervous+

y9_parent_tempertantrums+y9_parent_troublesleeping+y9_parent_whines+y9_parent_worries+y9_parent_makesfriendseasily+

y9_parent_selfconfident+y9_parent_interestindiffthings+y9_parent_invitesotherstohome+y9_parent_reportsaccappropr+

y9_teacher_poorsocialstudies+y9_teacher_makesfriendseasy+y9_teacher_receivecritiquewell+y9_teacher_respadeqwhenpushed+

y9_teacher_cleandesk+y9_teacher_anxingroupchildren+y9_teacher_arguesothers+y9_teacher_angryeasily+y9_teacher_likesbeingalone+

y9_teacher_disturbothers+y9_teacher_fidgethands+y9_teacher_poorreading+y9_teacher_shortattentionspan+y9_teacher_temperoutbursts+

y9_kid_neverhelphome+y9_kid_tvmorethan4hours+y9_kid_pickedoninschool+y9_kid_setfire+y9_kid_notdobest+y9_kid_hardpayattention+

y9_kid_easilydistracted+y9_kid_seldomseatbelt,

data = explimp4, seed = 1)$data

data.rose5 <- ROSE(autism ~ male+fampsych_anx_mside+y1_mother_dep+y1_overnighthosp+y3_physdisability+y3_speechprblm+y5_over2earinf+

y5_speechproblem+y9_phys_speechproblem+y9_phys_stuttering+y9_over2earinf+y9_parent_unknownphys_pains+y9_parent_constipated+

y3_playswithothers+y3_tooyoung+y3_cantconcentr+y3_defiant+y3_demandsmetdirect+y3_disobed+y3_distrbdchroutine+y3_noguilt+

y3_easilyfrustr+y3_overtired+y3_notsenstowpunish+y3_withdrawn+y5_treatenspeople+y5_cantconcentrate+y5_troublefallingasleep+

y5_nervous+y5_stubborn+y5_actstooyoungforage+y5_ratherbealone+y5_nervoustwitches+y5_clumsy+y5_playstalkothers+

y5_teacher_diagnoseddisability+y5_teacher_understconvofprint+y5_teacher_discussproblsparents+y9_parent_notlikedotherkids+

y9_parent_anxious+y9_parent_clumsy+y9_parent_compulsions+y9_parent_easydistracted+y9_parent_staresblankly+y9_parent_daydreams+

y9_parent_demandsattention+y9_parent_phobias+y9_parent_accidentprone+y9_parent_getteased+y9_parent_impulsive+y9_parent_nervous+

y9_parent_tempertantrums+y9_parent_troublesleeping+y9_parent_whines+y9_parent_worries+y9_parent_makesfriendseasily+

y9_parent_selfconfident+y9_parent_interestindiffthings+y9_parent_invitesotherstohome+y9_parent_reportsaccappropr+

y9_teacher_poorsocialstudies+y9_teacher_makesfriendseasy+y9_teacher_receivecritiquewell+y9_teacher_respadeqwhenpushed+

y9_teacher_cleandesk+y9_teacher_anxingroupchildren+y9_teacher_arguesothers+y9_teacher_angryeasily+y9_teacher_likesbeingalone+

y9_teacher_disturbothers+y9_teacher_fidgethands+y9_teacher_poorreading+y9_teacher_shortattentionspan+y9_teacher_temperoutbursts+

y9_kid_neverhelphome+y9_kid_tvmorethan4hours+y9_kid_pickedoninschool+y9_kid_setfire+y9_kid_notdobest+y9_kid_hardpayattention+

y9_kid_easilydistracted+y9_kid_seldomseatbelt,

data = explimp5, seed = 1)$data

fullmodel1 <- glm(autism~male+fampsych_anx_mside+y1_mother_dep+y1_overnighthosp+y3_physdisability+y3_speechprblm+y5_over2earinf+

y5_speechproblem+y9_phys_speechproblem+y9_phys_stuttering+y9_over2earinf+y9_parent_unknownphys_pains+y9_parent_constipated+

y3_playswithothers+y3_tooyoung+y3_cantconcentr+y3_defiant+y3_demandsmetdirect+y3_disobed+y3_distrbdchroutine+y3_noguilt+

y3_easilyfrustr+y3_overtired+y3_notsenstowpunish+y3_withdrawn+y5_treatenspeople+y5_cantconcentrate+y5_troublefallingasleep+

y5_nervous+y5_stubborn+y5_actstooyoungforage+y5_ratherbealone+y5_nervoustwitches+y5_clumsy+y5_playstalkothers+

y5_teacher_diagnoseddisability+y5_teacher_understconvofprint+y5_teacher_discussproblsparents+y9_parent_notlikedotherkids+

y9_parent_anxious+y9_parent_clumsy+y9_parent_compulsions+y9_parent_easydistracted+y9_parent_staresblankly+y9_parent_daydreams+

y9_parent_demandsattention+y9_parent_phobias+y9_parent_accidentprone+y9_parent_getteased+y9_parent_impulsive+y9_parent_nervous+

y9_parent_tempertantrums+y9_parent_troublesleeping+y9_parent_whines+y9_parent_worries+y9_parent_makesfriendseasily+

y9_parent_selfconfident+y9_parent_interestindiffthings+y9_parent_invitesotherstohome+y9_parent_reportsaccappropr+

y9_teacher_poorsocialstudies+y9_teacher_makesfriendseasy+y9_teacher_receivecritiquewell+y9_teacher_respadeqwhenpushed+

y9_teacher_cleandesk+y9_teacher_anxingroupchildren+y9_teacher_arguesothers+y9_teacher_angryeasily+y9_teacher_likesbeingalone+

y9_teacher_disturbothers+y9_teacher_fidgethands+y9_teacher_poorreading+y9_teacher_shortattentionspan+y9_teacher_temperoutbursts+

y9_kid_neverhelphome+y9_kid_tvmorethan4hours+y9_kid_pickedoninschool+y9_kid_setfire+y9_kid_notdobest+y9_kid_hardpayattention+

y9_kid_easilydistracted+y9_kid_seldomseatbelt, data=data.rose1, family="binomial")

fullmodel2 <- glm(autism~male+fampsych_anx_mside+y1_mother_dep+y1_overnighthosp+y3_physdisability+y3_speechprblm+y5_over2earinf+

y5_speechproblem+y9_phys_speechproblem+y9_phys_stuttering+y9_over2earinf+y9_parent_unknownphys_pains+y9_parent_constipated+

y3_playswithothers+y3_tooyoung+y3_cantconcentr+y3_defiant+y3_demandsmetdirect+y3_disobed+y3_distrbdchroutine+y3_noguilt+

y3_easilyfrustr+y3_overtired+y3_notsenstowpunish+y3_withdrawn+y5_treatenspeople+y5_cantconcentrate+y5_troublefallingasleep+

y5_nervous+y5_stubborn+y5_actstooyoungforage+y5_ratherbealone+y5_nervoustwitches+y5_clumsy+y5_playstalkothers+

y5_teacher_diagnoseddisability+y5_teacher_understconvofprint+y5_teacher_discussproblsparents+y9_parent_notlikedotherkids+

y9_parent_anxious+y9_parent_clumsy+y9_parent_compulsions+y9_parent_easydistracted+y9_parent_staresblankly+y9_parent_daydreams+

y9_parent_demandsattention+y9_parent_phobias+y9_parent_accidentprone+y9_parent_getteased+y9_parent_impulsive+y9_parent_nervous+

y9_parent_tempertantrums+y9_parent_troublesleeping+y9_parent_whines+y9_parent_worries+y9_parent_makesfriendseasily+

y9_parent_selfconfident+y9_parent_interestindiffthings+y9_parent_invitesotherstohome+y9_parent_reportsaccappropr+

y9_teacher_poorsocialstudies+y9_teacher_makesfriendseasy+y9_teacher_receivecritiquewell+y9_teacher_respadeqwhenpushed+

y9_teacher_cleandesk+y9_teacher_anxingroupchildren+y9_teacher_arguesothers+y9_teacher_angryeasily+y9_teacher_likesbeingalone+

y9_teacher_disturbothers+y9_teacher_fidgethands+y9_teacher_poorreading+y9_teacher_shortattentionspan+y9_teacher_temperoutbursts+

y9_kid_neverhelphome+y9_kid_tvmorethan4hours+y9_kid_pickedoninschool+y9_kid_setfire+y9_kid_notdobest+y9_kid_hardpayattention+

y9_kid_easilydistracted+y9_kid_seldomseatbelt, data=data.rose2, family="binomial")

fullmodel3 <- glm(autism~male+fampsych_anx_mside+y1_mother_dep+y1_overnighthosp+y3_physdisability+y3_speechprblm+y5_over2earinf+

y5_speechproblem+y9_phys_speechproblem+y9_phys_stuttering+y9_over2earinf+y9_parent_unknownphys_pains+y9_parent_constipated+

y3_playswithothers+y3_tooyoung+y3_cantconcentr+y3_defiant+y3_demandsmetdirect+y3_disobed+y3_distrbdchroutine+y3_noguilt+

y3_easilyfrustr+y3_overtired+y3_notsenstowpunish+y3_withdrawn+y5_treatenspeople+y5_cantconcentrate+y5_troublefallingasleep+

y5_nervous+y5_stubborn+y5_actstooyoungforage+y5_ratherbealone+y5_nervoustwitches+y5_clumsy+y5_playstalkothers+

y5_teacher_diagnoseddisability+y5_teacher_understconvofprint+y5_teacher_discussproblsparents+y9_parent_notlikedotherkids+

y9_parent_anxious+y9_parent_clumsy+y9_parent_compulsions+y9_parent_easydistracted+y9_parent_staresblankly+y9_parent_daydreams+

y9_parent_demandsattention+y9_parent_phobias+y9_parent_accidentprone+y9_parent_getteased+y9_parent_impulsive+y9_parent_nervous+

y9_parent_tempertantrums+y9_parent_troublesleeping+y9_parent_whines+y9_parent_worries+y9_parent_makesfriendseasily+

y9_parent_selfconfident+y9_parent_interestindiffthings+y9_parent_invitesotherstohome+y9_parent_reportsaccappropr+

y9_teacher_poorsocialstudies+y9_teacher_makesfriendseasy+y9_teacher_receivecritiquewell+y9_teacher_respadeqwhenpushed+

y9_teacher_cleandesk+y9_teacher_anxingroupchildren+y9_teacher_arguesothers+y9_teacher_angryeasily+y9_teacher_likesbeingalone+

y9_teacher_disturbothers+y9_teacher_fidgethands+y9_teacher_poorreading+y9_teacher_shortattentionspan+y9_teacher_temperoutbursts+

y9_kid_neverhelphome+y9_kid_tvmorethan4hours+y9_kid_pickedoninschool+y9_kid_setfire+y9_kid_notdobest+y9_kid_hardpayattention+

y9_kid_easilydistracted+y9_kid_seldomseatbelt, data=data.rose3, family="binomial")

fullmodel4 <- glm(autism~male+fampsych_anx_mside+y1_mother_dep+y1_overnighthosp+y3_physdisability+y3_speechprblm+y5_over2earinf+

y5_speechproblem+y9_phys_speechproblem+y9_phys_stuttering+y9_over2earinf+y9_parent_unknownphys_pains+y9_parent_constipated+

y3_playswithothers+y3_tooyoung+y3_cantconcentr+y3_defiant+y3_demandsmetdirect+y3_disobed+y3_distrbdchroutine+y3_noguilt+

y3_easilyfrustr+y3_overtired+y3_notsenstowpunish+y3_withdrawn+y5_treatenspeople+y5_cantconcentrate+y5_troublefallingasleep+

y5_nervous+y5_stubborn+y5_actstooyoungforage+y5_ratherbealone+y5_nervoustwitches+y5_clumsy+y5_playstalkothers+

y5_teacher_diagnoseddisability+y5_teacher_understconvofprint+y5_teacher_discussproblsparents+y9_parent_notlikedotherkids+

y9_parent_anxious+y9_parent_clumsy+y9_parent_compulsions+y9_parent_easydistracted+y9_parent_staresblankly+y9_parent_daydreams+

y9_parent_demandsattention+y9_parent_phobias+y9_parent_accidentprone+y9_parent_getteased+y9_parent_impulsive+y9_parent_nervous+

y9_parent_tempertantrums+y9_parent_troublesleeping+y9_parent_whines+y9_parent_worries+y9_parent_makesfriendseasily+

y9_parent_selfconfident+y9_parent_interestindiffthings+y9_parent_invitesotherstohome+y9_parent_reportsaccappropr+

y9_teacher_poorsocialstudies+y9_teacher_makesfriendseasy+y9_teacher_receivecritiquewell+y9_teacher_respadeqwhenpushed+

y9_teacher_cleandesk+y9_teacher_anxingroupchildren+y9_teacher_arguesothers+y9_teacher_angryeasily+y9_teacher_likesbeingalone+

y9_teacher_disturbothers+y9_teacher_fidgethands+y9_teacher_poorreading+y9_teacher_shortattentionspan+y9_teacher_temperoutbursts+

y9_kid_neverhelphome+y9_kid_tvmorethan4hours+y9_kid_pickedoninschool+y9_kid_setfire+y9_kid_notdobest+y9_kid_hardpayattention+

y9_kid_easilydistracted+y9_kid_seldomseatbelt, data=data.rose4, family="binomial")

fullmodel5 <- glm(autism~male+fampsych_anx_mside+y1_mother_dep+y1_overnighthosp+y3_physdisability+y3_speechprblm+y5_over2earinf+

y5_speechproblem+y9_phys_speechproblem+y9_phys_stuttering+y9_over2earinf+y9_parent_unknownphys_pains+y9_parent_constipated+

y3_playswithothers+y3_tooyoung+y3_cantconcentr+y3_defiant+y3_demandsmetdirect+y3_disobed+y3_distrbdchroutine+y3_noguilt+

y3_easilyfrustr+y3_overtired+y3_notsenstowpunish+y3_withdrawn+y5_treatenspeople+y5_cantconcentrate+y5_troublefallingasleep+

y5_nervous+y5_stubborn+y5_actstooyoungforage+y5_ratherbealone+y5_nervoustwitches+y5_clumsy+y5_playstalkothers+

y5_teacher_diagnoseddisability+y5_teacher_understconvofprint+y5_teacher_discussproblsparents+y9_parent_notlikedotherkids+

y9_parent_anxious+y9_parent_clumsy+y9_parent_compulsions+y9_parent_easydistracted+y9_parent_staresblankly+y9_parent_daydreams+

y9_parent_demandsattention+y9_parent_phobias+y9_parent_accidentprone+y9_parent_getteased+y9_parent_impulsive+y9_parent_nervous+

y9_parent_tempertantrums+y9_parent_troublesleeping+y9_parent_whines+y9_parent_worries+y9_parent_makesfriendseasily+

y9_parent_selfconfident+y9_parent_interestindiffthings+y9_parent_invitesotherstohome+y9_parent_reportsaccappropr+

y9_teacher_poorsocialstudies+y9_teacher_makesfriendseasy+y9_teacher_receivecritiquewell+y9_teacher_respadeqwhenpushed+

y9_teacher_cleandesk+y9_teacher_anxingroupchildren+y9_teacher_arguesothers+y9_teacher_angryeasily+y9_teacher_likesbeingalone+

y9_teacher_disturbothers+y9_teacher_fidgethands+y9_teacher_poorreading+y9_teacher_shortattentionspan+y9_teacher_temperoutbursts+

y9_kid_neverhelphome+y9_kid_tvmorethan4hours+y9_kid_pickedoninschool+y9_kid_setfire+y9_kid_notdobest+y9_kid_hardpayattention+

y9_kid_easilydistracted+y9_kid_seldomseatbelt, data=data.rose5, family="binomial")

## See which variables are sign predictive in all models

summary(fullmodel1)

summary(fullmodel2)

summary(fullmodel3)

summary(fullmodel4)

summary(fullmodel5)

###### Use only the variables selected above

data.rose1 <- ROSE(autism ~ fampsych_anx_mside+

male+

y5_stubborn+

y9_kid_neverhelphome+

y9_parent_selfconfident+

y9_parent_interestindiffthings+

y9_phys_speechproblem+

y9_teacher_arguesothers+

y9_teacher_makesfriendseasy,

data = explimp1, seed = 1)$data

data.rose2 <- ROSE(autism ~ fampsych_anx_mside+

male+

y5_stubborn+

y9_kid_neverhelphome+

y9_parent_selfconfident+

y9_parent_interestindiffthings+

y9_phys_speechproblem+

y9_teacher_arguesothers+

y9_teacher_makesfriendseasy,

data = explimp2, seed = 1)$data

data.rose3 <- ROSE(autism ~ fampsych_anx_mside+

male+

y5_stubborn+

y9_kid_neverhelphome+

y9_parent_selfconfident+

y9_parent_interestindiffthings+

y9_phys_speechproblem+

y9_teacher_arguesothers+

y9_teacher_makesfriendseasy,

data = explimp3, seed = 1)$data

data.rose4 <- ROSE(autism ~ fampsych_anx_mside+

male+

y5_stubborn+

y9_kid_neverhelphome+

y9_parent_selfconfident+

y9_parent_interestindiffthings+

y9_phys_speechproblem+

y9_teacher_arguesothers+

y9_teacher_makesfriendseasy,

data = explimp4, seed = 1)$data

data.rose5 <- ROSE(autism ~ fampsych_anx_mside+

male+

y5_stubborn+

y9_kid_neverhelphome+

y9_parent_selfconfident+

y9_parent_interestindiffthings+

y9_phys_speechproblem+

y9_teacher_arguesothers+

y9_teacher_makesfriendseasy,

data = explimp5, seed = 1)$data

fullmodel1 <- glm(autism~fampsych_anx_mside+

male+

y5_stubborn+

y9_kid_neverhelphome+

y9_parent_selfconfident+

y9_parent_interestindiffthings+

y9_phys_speechproblem+

y9_teacher_arguesothers+

y9_teacher_makesfriendseasy, data=data.rose1, family="binomial")

fullmodel2 <- glm(autism~fampsych_anx_mside+

male+

y5_stubborn+

y9_kid_neverhelphome+

y9_parent_selfconfident+

y9_parent_interestindiffthings+

y9_phys_speechproblem+

y9_teacher_arguesothers+

y9_teacher_makesfriendseasy, data=data.rose2, family="binomial")

fullmodel3 <- glm(autism~fampsych_anx_mside+

male+

y5_stubborn+

y9_kid_neverhelphome+

y9_parent_selfconfident+

y9_parent_interestindiffthings+

y9_phys_speechproblem+

y9_teacher_arguesothers+

y9_teacher_makesfriendseasy, data=data.rose3, family="binomial")

fullmodel4 <- glm(autism~fampsych_anx_mside+

male+

y5_stubborn+

y9_kid_neverhelphome+

y9_parent_selfconfident+

y9_parent_interestindiffthings+

y9_phys_speechproblem+

y9_teacher_arguesothers+

y9_teacher_makesfriendseasy, data=data.rose4, family="binomial")

fullmodel5 <- glm(autism~fampsych_anx_mside+

male+

y5_stubborn+

y9_kid_neverhelphome+

y9_parent_selfconfident+

y9_parent_interestindiffthings+

y9_phys_speechproblem+

y9_teacher_arguesothers+

y9_teacher_makesfriendseasy, data=data.rose5, family="binomial")

### Pool coefficients

c1 <- as.data.frame(fullmodel1$coefficients)

c2 <- as.data.frame(fullmodel2$coefficients)

c3 <- as.data.frame(fullmodel3$coefficients)

c4 <- as.data.frame(fullmodel4$coefficients)

c5 <- as.data.frame(fullmodel5$coefficients)

### Use fit of model 5 to use pooled coefficients

fullmodel5[["coefficients"]][["(Intercept)"]] <- -1.204917405

fullmodel5[["coefficients"]][["fampsych_anx_mside"]] <- 0.954430075

fullmodel5[["coefficients"]][["male"]] <- 0.83087033

fullmodel5[["coefficients"]][["y5_stubborn"]] <- 1.115713635

fullmodel5[["coefficients"]][["y9_kid_neverhelphome"]] <- 0.7337974

fullmodel5[["coefficients"]][["y9_parent_selfconfident"]] <- -0.85764839

fullmodel5[["coefficients"]][["y9_parent_interestindiffthings"]] <- -0.741793805

fullmodel5[["coefficients"]][["y9_phys_speechproblem"]] <- 2.08982273

fullmodel5[["coefficients"]][["y9_teacher_arguesothers"]] <- 0.55041304

fullmodel5[["coefficients"]][["y9_teacher_makesfriendseasy"]] <- -1.036422205

##### Examine accuracy

holdoutimp1 <- read_sav("Data/imputations asd/asdimpholdout1.sav")

holdoutimp2 <- read_sav("Data/imputations asd/asdimpholdout2.sav")

holdoutimp3 <- read_sav("Data/imputations asd/asdimpholdout3.sav")

holdoutimp4 <- read_sav("Data/imputations asd/asdimpholdout4.sav")

holdoutimp5 <- read_sav("Data/imputations asd/asdimpholdout5.sav")

### Run model in all 5 imputed holdout dataset

glm_probs <- data.frame(probs = predict(fullmodel5, newdata = holdoutimp5,type="response"))

glm_pred <- glm_probs %>% mutate(pred = ifelse(probs>.8, "1", "0"))

glm_pred <- cbind(holdoutimp5, glm_pred)

glm_pred %>% count(pred, autism) %>% spread(autism, n, fill = 0)

#1

#Sensitivity

76/(76+15)*100 # 83.5%

#PPV

76/(491+76)*100 # 13.4%

#2

#Sensitivity

75/(75+16)*100 # 82.4%

#PPV

75/(552+75)*100 # 11.96%

#3

#Sensitivity

75/(75+16)*100 # 82.4%

#PPV

75/(509+75)*100 # 12.84%

#4

#Sensitivity

76/(76+15)*100 # 83.5%

#PPV

76/(543+76)*100 # 12.3%

#5

#Sensitivity

67/(67+24)*100 #73.6%

#PPV

67/(494+67)*100 # 11.94%

### Pooled estimates

######################

### Final accuracy

######################

#Sensitivity

(83.5+82.4+83.5+82.4+73.6)/5 # 81.08%

#PPV

(13.4+11.96+12.84+12.3+11.94)/5 # 12.45%

##############################

##### Increase probability to 80%

##############################

### Run model in all 5 imputed holdout dataset

glm_probs <- data.frame(probs = predict(fullmodel5, newdata = holdoutimp5,type="response"))

glm_pred <- glm_probs %>% mutate(pred = ifelse(probs>.8, "1", "0"))

glm_pred <- cbind(holdoutimp5, glm_pred)

glm_pred %>% count(pred, autism) %>% spread(autism, n, fill = 0)

#1

#Sensitivity

45/(45+46)*100 # 49.5%

#PPV

45/(119+45)*100 # 27.4%

#2

#Sensitivity

49/(49+42)*100 # 53.8%

#PPV

49/(145+49)*100 # 25.3%

#3

#Sensitivity

49/(49+42)*100 # 53.8%

#PPV

49/(143+49)*100 # 25.5%

#4

#Sensitivity

49/(49+42)*100 # 53.8%

#PPV

49/(136+49)*100 # 26.5%

#5

#Sensitivity

42/(49+42)*100 # 46.2%

#PPV

42/(127+42)*100 # 24.9%

### Pooled estimates

######################

### Final accuracy

######################

#Sensitivity

(49.5+53.8+53.8+53.8+46.2)/5 # 51.4%

#PPV

(27.4+25.3+25.5+26.5+24.9)/5 # 25.9%
